# Checklist of British and Irish Hymenoptera - Chalcidoidea and Mymarommatoidea

**DOI:** 10.3897/BDJ.4.e8013

**Published:** 2016-06-06

**Authors:** Natalie Dale-Skey, Richard R. Askew, John S. Noyes, Laurence Livermore, Gavin R. Broad

**Affiliations:** ‡ The Natural History Museum, London, United Kingdom; § private address, France, France; | The Natural History Museum, London, London, United Kingdom

**Keywords:** Chalcidoidea, Mymarommatoidea, fauna.

## Abstract

**Background**

A revised checklist of the British and Irish Chalcidoidea and Mymarommatoidea substantially updates the previous comprehensive checklist, dating from 1978. Country level data (i.e. occurrence in England, Scotland, Wales, Ireland and the Isle of Man) is reported where known.

**New information**

A total of 1754 British and Irish Chalcidoidea species represents a 22% increase on the number of British species known in 1978.

## Introduction

This paper continues the series of checklists of the Hymenoptera of Britain and Ireland, starting with Broad and Livermore (2014a), Broad and Livermore (2014b) and Liston et al. (2014). The Introduction to the series (Broad 2014) sets out the background and rationale. We aim to produce an up-to-date and accurate list of all Hymenoptera species recorded reliably from Britain and Ireland.

With more than 22,000 described species worldwide, Chalcidoidea is one of the largest and most diverse Hymenoptera superfamilies. Most species are under 3mm, and the group includes the smallest known winged insect, *Kikiki huna* Huber & Beardsley (a mymarid not found in the British Isles) measuring only 0.16 mm in body length (Huber and Noyes 2013). Chalcidoidea show an amazingly diverse range of biologies; the majority of taxa are parasitoids, attacking all stages of hosts in most insect orders as well as some arachnids, but phytophagous taxa and taxa with predatory larvae are also known. Information about Chalcidoidea classification and biology can be found in John Noyes’s Universal Chalcidoidea Database (UCD), which contains original citations as well as references for all subsequent generic combinations and synonymies, and extensive lists of published host and distribution records. While the latter can be used to generate regional lists of Chalcidoidea, a British and Irish species list extracted from the UCD will not match the present checklist, from which published erroneous records have been removed and to which a large number of unpublished records based on reliably identified specimens in collections have been added.

Currently 22 (extant) Chalcidoidea families are recognized, 16 of which are represented in the British and Irish fauna. There have been some changes to the family-level classification recently (Heraty et al. 2013) and more may be anticipated. The only family-level change resulting from the Heraty et al. (2013) study that affects the British fauna is the elevation of the Azotidae to family status, from being a subfamily of the Aphelinidae. However, other major changes to the classification of Chalcidoidea since the 1978 checklist (Bouček and Graham 1978) include the removal of Mymarommatidae to its own superfamily (Gibson 1986; Noyes and Valentine 1989), the restriction of the Miscogastrinae of the Pteromalidae to just Graham’s (1969) tribe Miscogasterini (Bouček 1988), the inclusion of the family Elasmidae as a tribe of Eulophinae (Gauthier et al. 2000), and the synonymy of the Pteromalidae subfamilies Cratominae and Panstenoninae under Pteromalinae (Heraty et al. 2013).

The British and Irish Chalcidoidea list, with 1754 species, is now 22% larger than in 1978 (see Table 1). This increase results mainly from additional records (including 42 unpublished records obtained from collection surveys - see Suppl. material 3) but also to a lesser extent from a net "gain" of taxa through taxonomic changes, as summarised in Table 2. Only 4 species included in the 1978 checklist have been excluded: *Elasmus rufiventris* Ferrière, *Tetrastichus agrilorum* (Ratzeburg), *Tetrastichus tompanus* (Erdös) and *Entedon ulmi* Erdös. A detailed comparison of the 1978 checklist with the 2016 checklist can be found in Suppl. material 2.

Some of the diversity of British chalcids is illustrated in Figs 1, 2, 3, 4, 5, 6, 7, 8, 9, 10, 11, 12, 13, 14, 15, 16, 17, 18, 19, 20, 21, 22, 23, 24, 25, 26, 27, 28. Additional images of representatives of the British and Irish fauna can be found on the Flickr site of the BMNH Hymenoptera section, NHM Wasps.

## Materials and methods

The bulk of the data behind this checklist is from the UCD, with further editing and research by the authors. Nomenclature follows the UCD, and distribution data are taken from the collections of the BMNH, the UCD and from various published sources, which are cited. Nomenclatural acts can be traced through the UCD; deviations from that source are cited. A more complete methodology can be found in Broad (2014). Although we include extensive synonymy, this is a checklist and not a catalogue. Names that have been used in relevant literature can be found here but we do not document all taxonomic acts as these can be traced in readily accessible catalogues, particularly the UCD.

The following conventions and abbreviations are used here:

[*species*] taxon deleted from the British and Irish list

BMNH Natural History Museum, London

# known or suspected introductions with at least temporarily self-sustaining populations

? status (including uncertain synonymy) or identification in the British Isles uncertain

misident. has been misidentified as this name

[*species* nom. dub]. nomen dubium, a name of doubtful status

nom. ob. nomen oblitum, ‘forgotten name’, does not have priority over a younger name

nom. nov. nomen novum, a replacement name

nom. nud. nomen nudum, an unavailable name, with no type specimen

preocc. name preoccupied (junior homonym)

stat. rev. status revocatus, revived status (e.g., raised from synonymy)

unavailable name unavailable under provisions of the ICZN code

var. variety, only available as a valid name under certain provisions of the ICZN code

An alternative version of the Chalcidoidea checklist as an Excel spreadsheet is provided here in the supplementary materials (Suppl. material 1).

Future updates to the British and Irish Chalcioidea checklist will be incorporated in an online version of the checklist at Hymenoptera of the British Isles.

## Checklists

### Family Aphelinidae Thomson, 1876

#### Aphelininae

Dalman, 1820

#### Aphelinus

Dalman, 1820

MYINA Nees, 1834
ERIOPHILUS Haldeman, 1851
MESIDIA Förster, 1856
MEROLIGON Rondani, 1877
MESIDIOPSIS Nowicki, 1930
INDAPHELINUS Hayat, 1990

#### Aphelinus abdominalis

(Dalman, 1820)

Entedon abdominalis Dalman, 1820
basalis (Westwood, 1833, *Agonioneurus*)
facialis (Förster, 1841, *Myina*)
flaviceps (Förster, 1841, *Myina*)
flavipes (Förster, 1841, *Myina*)
ultor (Rondani, 1848, *Encyrtus*)
polycyclus (Förster, 1861, *Agonioneurus*)
longicornis (Ferrière, 1962, *Mesidia*)
alius Yasnosh, 1963
bicolor Yasnosh, 1963

##### Distribution

England, Wales, Ireland

#### Aphelinus annulipes

(Walker, 1851)

Myina annulipes Walker, 1851

##### Distribution

Wales, Ireland

#### Aphelinus argiope

Walker, 1839

pumila (Mayr, 1904, *Mesidia*)

##### Distribution

England

#### Aphelinus asychis

Walker, 1839

euthria Walker, 1839
affinis (Förster, 1841, *Myina*)
brevicalcar Thomson, 1876
brachyptera Kurdjumov, 1913
dubia Kurdjumov, 1913

##### Distribution

England, Wales, Ireland

#### Aphelinus chaonia

Walker, 1839

transversus Thomson, 1876
flavicornis (Förster, 1841, *Myina*)

##### Distribution

England, Ireland

#### Aphelinus daucicola

Kurdjumov, 1913

brunneus Yasnosh, 1963

##### Distribution

England

#### Aphelinus flaviventris

Kurdjumov, 1913

##### Distribution

England

#### Aphelinus fulvus

Yasnosh, 1963

##### Distribution

England

#### Aphelinus humilis

Mercet, 1927

##### Distribution

England, Ireland

#### Aphelinus mali

(Haldeman, 1851)

Eriophilus mali Haldeman, 1851
rosae (Ashmead, 1886, *Blastothrix*)
varicornis Girault, 1909

##### Distribution

England

#### Aphelinus subflavescens

(Westwood, 1837)

Agonioneurus subflavescens Westwood, 1837

##### Distribution

England, Scotland, Ireland

#### Aphelinus tetrataenion

(Erdös & Novicky, 1953)

Mesidia tetrataenion Erdös & Novicky, 1953

##### Distribution

England, Wales

#### Aphelinus thomsoni

Graham, 1976

flavus misident

##### Distribution

England, Scotland, Ireland

##### Notes

According to Graham (1976) *Eulophus flavus* Nees, 1834 is probably an encyrtid; *A. thomsoni* is Graham's nom. nov. for what Thomson called *flavus*. Walker misidentified *subflavescens* as *flavus*.

#### Aphelinus varipes

(Förster, 1841)

Myina varipes Förster, 1841
nigritus Howard, 1908

##### Distribution

England, Wales, Ireland

#### Aphytis

Howard, 1900

PROSPAPHELINUS De Gregorio, 1914
PARAPHYTIS Compere, 1925
SYEDIELLA Shafee, 1970

#### Aphytis aonidiae

(Mercet, 1911)

Aphelinus aonidiae Mercet, 1911
dubius De Santis, 1948
intermedius De Santis, 1948
citrinus Compere, 1955

##### Distribution

England

#### Aphytis diaspidis

(Howard, 1881)

Aphelinus diaspidis Howard, 1881
fuscipennis (Howard, 1881, *Aphelinus*)
ovidii (Girault, 1919, *Aphelinus*)
opuntiae Risbec, 1952
madagascariensis (Risbec, 1952, *Prosaphelinus*)
risbeci Annecke & Insley, 1971

#### Aphytis mytilaspidis

(Le Baron, 1870)

Chalcis mytilaspidis Le Baron, 1870
albidus (Westwood, 1837, *Agonioneurus*) nomen oblitum
variolosum Alam, 1956
diaspidioti Chumakova, 1957

##### Distribution

England

#### Aphytis proclia

(Walker, 1839)

Aphelinus proclia Walker, 1839
zonatus Alam, 1956
sugonjaevi Yasnosh, 1972
chowdhurii (Kaul, 1974, *Centrodora*)

##### Distribution

England

#### Centrodora

Förster, 1878

DEBACHIELLA Gordh & Rosen, 1973
MICROEUPELMUS Otten, 1941
OOLATHRON De Santis, 1981
PARAPHELINUS Perkins, 1906
PECHLANERIA Soyka, 1948
PLASTOCHARELLA Girault, 1913
TUMIDISCAPUS Girault, 1911

#### Centrodora amoena

Förster, 1878

bolivari Mercet, 1930
speciosissima misident.

##### Distribution

England

#### Centrodora livens

(Walker, 1851)

Myina livens Walker, 1851
varius (Blood, 1929, *Paraphelinus*)
danica Mercet, 1930
alpina (Soyka, 1948, *Pechlaneria*)

##### Distribution

England

#### Centrodora locustarum

(Giraud, 1863)

Agonioneurus locustarum Giraud, 1863

##### Distribution

England

#### Marietta

Motschulsky, 1863

PERISSOPTERUS Howard, 1895
PSEUDAPHELINUS Brèthes, 1918

#### Marietta picta

(André, 1878)

Agonioneurus pictus André, 1878
pantherinus (Giraud, 1878, *Coccophagus*)
zebra (Kurdjumov, 1912, *Perissopterus*)
zebra (Mercet, 1914, *Perissopterus*) preocc.
zebratus (Mercet, 1916, *Perissopterus*)
anglicus (Blood, 1929, *Perissopterus*)

##### Distribution

England

#### Coccophaginae

Förster, 1878

#### Coccobius

Ratzeburg, 1852

PHYSCUS Howard, 1895
ENCYRTOPHYSCUS Blanchard, 1948
PHYSCULUS Yasnosh, 1977

#### Coccobius annulicornis

Ratzeburg, 1852

testaceus (Masi, 1909, *Physcus*)

#### Coccophagoides

Girault, 1915

DIASPINIPHAGUS Silvestri, 1927
PRIMAPROSPALTELLA DeBach & LaSalle, 1981

#### Coccophagoides moeris

(Walker, 1839)

Aphelinus moeris Walker, 1839
janias (Walker, 1839, Pteroptrix)
similis (Masi, 1908, Prospalta)
ilicis (Mercet 1921, Prospaltella)
parvipennis Ferrière, 1955
silwoodensis (Alam, 1956, *Prospaltella*)

##### Distribution

England

#### Coccophagus

Westwood, 1833

ANERISTUS Howard, 1895
PARACHARITOPUS Brèthes, 1913
ATANEOSTIGMA Girault, 1914
EUXANTHELLUS Silvestri, 1915
PROCOCCOPHAGUS Silvestri, 1915
TANEOSTIGMOIDELLA Girault, 1915
ONOPHILUS Brèthes, 1918
PARENCARSIA Mercet, 1930
HEPTACRITUS De Santis, 1960
ACLERDAEPHAGUS Sugonjaev, 1969

#### Coccophagus gurneyi

Compere, 1929

##### Distribution

England

##### Notes

BMNH, det. Japosshvili, added here. Probably introduced from Australia via The Netherlands and may not be established.

#### Coccophagus hemera

(Walker, 1839)

Pteroptrix hemera Walker, 1839
krygeri Mercet, 1929
longifasciatus misident.

##### Distribution

England

#### Coccophagus lycimnia

(Walker, 1839)

Aphelinus lycimnia Walker, 1839
obscurus Westwood, 1833
pulchellus Westwood, 1833
scutellaris (Nees, 1834, *Eulophus*)
lecanii (Fitch, 1859, *Platygaster*)
ater Howard, 1881
cognatus Howard, 1881
vividus Howard, 1885
californicus Howard, 1889
coccidis Girault, 1917
corni Alam, 1956
taxi Alam, 1956

##### Distribution

England, Ireland

#### Coccophagus obscurus

Westwood, 1833

niger Masi, 1909
insidiator misident.

##### Distribution

England, Ireland

#### Coccophagus pulchellus

Westwood, 1833

apicalis (Förster, 1841, *Myina*)
scutellaris (Förster, 1841, *Myina*) preocc.
foersteri (Dalla Torre, 1898, *Aphelinus*)
howardi Masi, 1907

##### Distribution

England

#### Coccophagus semicircularis

(Förster, 1841)

Myina semicircularis Förster, 1841
xanthostictus (Ratzeburg, 1852, *Encyrtus*)
nigrifrons Wollaston, 1858
lunulatus Howard, 1894
scutellaris misident. (misidentification of *Coccophagus scutellaris* (Dalman, 1820, *Entedon*)).

##### Distribution

England

##### Notes

BMNH, added here

#### Encarsia

Förster, 1878

ASPIDIOTIPHAGUS Howard, 1894
PROSPALTA Howard, 1894
PROSPALTELLA Ashmead, 1904
PROSPALTOIDES Brèthes, 1914
MIMATOMUS Cockerell, 1911
DOLORESIA Mercet, 1912
PARASPIDIOTIPHAGUS Alam, 1956
ALEURODIPHILUS DeBach & Rose, 1981

#### Encarsia brittanica

(Girault, 1915)

Coccophagus brittanicus Girault, 1915

#### Encarsia citrina

(Craw, 1891)

Coccophagus citrinus Craw, 1891
australiensis (Girault, 1913, *Aspidiotiphagus*)
cyanophilli (Alam, 1956, *Aspidiotiphagus*)
schoeversi (Smits van Burgst, 1915, *Aspidiotiphagus*)
severiniellus (Ghesquière, 1933, *Aspidiotiphagus*)
silwoodensis (Alam, 1956, *Aspidiotiphagus*)
howardi (Brèthes, 1914, *Prospaltoides*)

##### Distribution

England

#### Encarsia formosa

Gahan, 1924

##### Distribution

England, Scotland, Ireland

##### Notes

Introduced into greenhouses for biological control.

#### Encarsia harrisoni

Polaszek, 2014

##### Distribution

England

##### Notes

Added by Polaszek (2014)

#### Encarsia inaron

(Walker, 1839)

Aphelinus inaron Walker, 1839
idaeus (Walker, 1839, *Aphelinus*)
borealis Hulden, 1986
brassicae Shafee & Bela, 1984
indifferentis Mercet, 1929
partenopea Masi, 1909
aleyrodis (Mercet, 1930, *Trichaporus*)

##### Distribution

England

#### Encarsia leucaspidis

(Mercet, 1912)

Prospaltella leucaspidis Mercet, 1912

##### Distribution

England

##### Notes

Specimen in Askew coll. from East Malling, Kent ex *Quadraspidiotus*, coll. 1974 by M. Copland but perhaps from a culture.

#### Encarsia lutea

(Masi, 1909)

Prospaltella lutea Masi, 1909
Encarsia lutea *sancta* (Girault, 1928, *Coccophagus*)

##### Distribution

England

##### Notes

Added by Springate and Noyes (1990)

#### Encarsia tricolor

Förster, 1878

coniugata (Masi, 1908, *Prospalta*)

##### Distribution

England

#### Pteroptrix

Westwood, 1833

ARCHENOMUS Westwood, 1833
GYROLASIA Förster, 1856
ARCHENOMUS Howard, 1898
PTEROTHRIX Dalla Torre, 1898
ARTAS Howard, 1907
CASCA Howard, 1907
HISPANIELLA Mercet, 1912
PTEROPTRICHOIDES Fullaway, 1913
APTEROPTRIX Girault, 1915
PSEUDOPTEROPTRIX Fullaway, 1918
OA Girault, 1929
APHELOSOMA Nikol'skaya, 1963
ARCHENOMISCUS Nikol'skaya & Yasnosh, 1966

#### Pteroptrix bicolor

(Howard, 1898)

Archenomus bicolor Howard, 1898
caucasica Yasnosh, 1955
callunae Alam, 1956
zonatus Alam, 1956

##### Distribution

England

#### Pteroptrix dimidiata

Westwood, 1833

britannica (Alam, 1856, *Casca*)
occidentalis (Silvestri & Mercet, 1928, *Casca*)

##### Distribution

England

#### Pteroptrix longiclava

(Girault, 1915)

Apteroptrix longiclava Girault, 1915
longicornis Nikol'skaya, 1959

##### Distribution

England

#### Eretmocerinae

Shafee & Khan, 1978

#### Eretmocerus

Haldeman, 1850

RICINUSA Risbec, 1951

#### Eretmocerus corni

Haldeman, 1850

#### Eretmocerus mundus

Mercet, 1931

masii Silvestri, 1931
aligarhensis Khan & Shafee, 1980
longipilus Khan & Shafee, 1980

### Family Azotidae Nikol'skaya & Yasnosh, 1966

#### Ablerus

Howard, 1894

AZOTUS Howard, 1898
MYOCNEMELLA Girault, 1913
DIMACROCERUS Brèthes, 1914

#### Ablerus celsus

(Brèthes, 1914)

Pteroptrix celsus Walker, 1839
brittanicus (Alam, 1956, *Azotus*)

##### Notes

England

### Family Chalcididae Latreille, 1817

#### Chalcidinae

Latreille, 1817

#### Brachymeria

Westwood, 1829

THAUMATELIA Kirby, 1883
ONCOCHALCIS Cameron, 1904
HOLOCHALCIS Kieffer, 1905
CEYXIA Girault, 1911
TUMIDICOXA Girault, 1911
THAUMATELIANA Girault, 1912
BRACHEPITELIA Girault, 1913
PSEUDEPITELIA Girault, 1913
TUMIDICOXELLA Girault, 1913
TUMIDICOXOIDES Girault, 1913
DIRRHINOMORPHA Girault & Dodd, 1915
MIROCHALCIS Girault, 1915
MEYERIELLA Krausse, 1917
AUSTRALOCHALCIS Girault, 1939

##### Notes

Species of *Brachymeria* removed from the British and Irish list:

[*femorata* (Panzer, 1801, *Chalcis*), syn. *ornatipes* (Cameron, 1906, *Chalcis*)]

Only ever regarded as a British species on the basis of two specimens without data in Manchester Museum (Ferrière and Kerrich 1958).

#### Brachymeria minuta

(Linnaeus, 1767)

Vespa minuta Linnaeus, 1767
femoralis (Geoffroy, 1785, *Sphex*)
pusilla (Fabricius, 1787, *Chalcis*)
saltatrix (Cuvier, 1833, *Evania*)
brevicornis (Klug, 1834, *Chalcis*)
scrobiculata (Förster, 1859, *Chalcis*)
tricolor (Förster, 1859, *Chalcis*)
fumata (Thomson, 1876, *Chalcis*)
paraplesia (Crawford, 1910, *Chalcis*)
jezoensis (Matsumura, 1918, *Chalcis*)
picea Nikol'skaya, 1952
putturensis Joseph, Narendran & Joy, 1971
calopeplae Joseph, Narendran & Joy, 1972

##### Distribution

England, Wales

#### Brachymeria obtusata

(Förster, 1859)

Chalcis obtusata Förster, 1859
vicina (Walker, 1834, *Chalcis*) preocc.

##### Distribution

England

##### Notes

**Figure 1.**
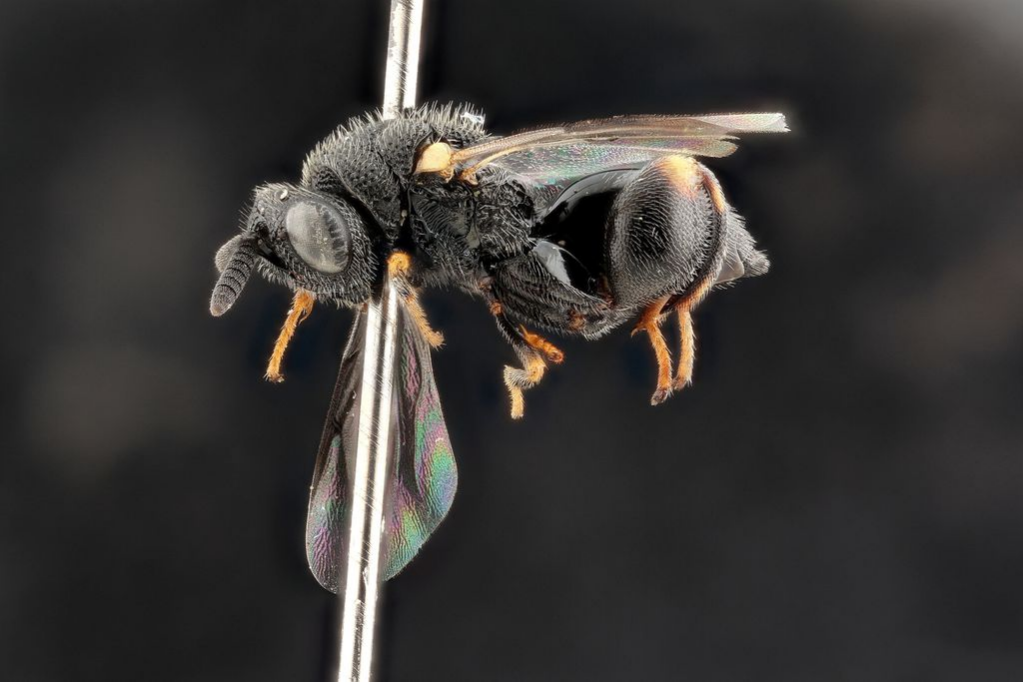
Chalcididae, Chalcidinae: *Brachymeria obtusata* (Förster) female, BMNH specimen BMNH(E)953654 [09/2006; Erith Old Borax Works; Bexley; Kent] (photo K. McCormack)

Added by Jones (2007). See Fig. 1 for habitus.

#### Brachymeria tibialis

(Walker, 1834)

Chalcis tibialis Walker, 1834
cingulata (Walker, 1834, *Chalcis*)
distinguenda (Walker, 1834, *Chalcis*)
intermedia (Nees, 1834, *Chalcis*)
scirropoda (Förster, 1859, *Chalcis*)
boops (Thomson, 1876, *Chalcis*)
rufofemorata Rosenhauer, 1856
quettaensis (Cameron, 1906, *Oncochalcis*)

##### Distribution

England

##### Notes

The origin of old English specimens was queried by Ferrière and Kerrich (1958). Its status as a British insect was confirmed by Jones (2008).

#### Chalcis

Fabricius, 1787

SMIERA Spinola, 1811
SMICRA Spinola, 1837

#### Chalcis biguttata

Spinola, 1808

melanuris Dalman, 1818
melanaris Dalman, 1820
macleanii (Curtis, 1833, *Smiera*)

##### Distribution

England

#### Chalcis myrifex

(Sulzer, 1776)

Sphex myrifex Sulzer, 1776
nigrifex (Sulzer, 1776, *Sphex*)
dearticulata (Fourcroy, 1785, *Vespa*)
petiolata (Curtis, 1833, *Smiera*)

##### Distribution

England

#### Chalcis sispes

(Linnaeus, 1761)

Sphex sispes Linnaeus, 1761
clavipes Fabricius, 1787
crassipes Desmarest, 1875
microstigma (Thomson, 1876, *Smicra*)

##### Distribution

England, Ireland

#### Conura

Spinola, 1837

SPILOCHALCIS Thomson, 1876
EPINAEUS Kirby, 1883
PROCTOCERAS Kirby, 1883
THAUMAPUS Kirby, 1883
DIPLODONTIA Ashmead, 1888
METADONTIA Ashmead, 1888
CERATOSMICRA Ashmead, 1904
ENNEASMICRA Ashmead, 1904
EUSAYIA Ashmead, 1904
EUSTYPIURA Ashmead, 1904
HEPTASMICRA Ashmead, 1904
HEXASMICRA Ashmead, 1904
MISCHOSMICRA Ashmead, 1904
OCTOSMICRA Ashmead, 1904
PENTASMICRA Ashmead, 1904
SAYIELLA Ashmead, 1904
TETRASMICRA Ashmead, 1904
TRISMICRA Ashmead, 1904
XANTHOMELANUS Ashmead, 1904
PLAGIOSMICRA Cameron, 1905
THAUMATOPUS Schulz, 1906
ARRETOCEROIDELLA Girault, 1913
MIXOCHALCIS Blanchard, 1935
PSYCHIDOSMICRA Blanchard, 1935
ETEROCHALCIS Burks, 1939
GRISSELLIELLA Narendran, 1988

#### Conura xanthostigma

(Dalman, 1820)

Chalcis xanthostigma Dalman, 1820
simlaensis (Cameron, 1902, *Spilochalcis*)
indica (Mani, 1935, *Spilochalcis*)
fletcheri (Mani, 1936, *Spilochalcis*)

##### Distribution

England

#### Haltichellinae

Ashmead, 1904

#### Haltichella

Spinola, 1811

MICROCHALCIS Kieffer, 1905

#### Haltichella rufipes

(Olivier, 1791)

Chalcis rufipes Olivier, 1791
armata (Panzer, 1801, *Chalcis*)
bispinosa (Fabricius, 1804, *Chalcis*)
bidentata (Schmitz, 1946, *Hockeria*)
quadridens (Kieffer, 1905, *Microchalcis*)

##### Distribution

England

#### Neochalcis

Kirby, 1883

ORTHOCHALCIS Kieffer, 1905
EUGASTROCHALCIS Masi, 1929

#### Neochalcis fertoni

(Kieffer, 1899)

Euchalcis fertoni Kieffer, 1899
barbara (Benoist, 1921, *Euchalcis*)

##### Distribution

England

##### Notes

Added by Askew (1992d)​

#### Proconura

Dodd, 1915

NEOCHALCIDIA Husain, Rauf & Kudeshia, 1985

##### Notes

Species of *Proconura* removed from the British and Irish list:

[*caryobori* (Hanna, 1934, *Euchalcidia*), syn. *indica* (Mani & Dubey, 1974, *Peltochalcidia*), syn. *trisulia* (Mani & Dubey, 1974, *Lasiochalcidia*), syn. *ricini* (Roy & Farooqi, 1984, *Euchalcidia*)]

Only recorded as an accidental importation in stored products.

#### Psilochalcis

Kieffer, 1905

LEPTOCHALCIS Kieffer, 1905
EUCHALCIDIA Masi, 1929
INVREIA Masi, 1929
CHALCIDIOPSIS Masi, 1933
PELTOCHALCIDIA Steffan, 1948
HYPERCHALCIDIA Steffan, 1951
PARINVREIA Steffan, 1951

#### Psilochalcis subarmata

(Förster, 1855)

Haltichella subarmata Förster, 1855
subaenea misident.
tarsalis (Förster, 1859, *Haltichella*)

##### Distribution

England

##### Notes

Omitted by Bouček and Graham (1978). Included in Ferrière and Kerrich (1958) as *Invreia subaenea* Masi, 1929; a further English record was published by.

### Family Encyrtidae Walker, 1837

#### Encyrtinae

Walker, 1837

#### Adelencyrtus

Ashmead, 1900

#### Adelencyrtus aulacaspidis

(Brèthes, 1914)

Prionomitus aulacaspidis Brèthes, 1914

##### Distribution

England

#### Ageniaspis

Dahlbom, 1857

#### Ageniaspis atricollis

(Dalman, 1820)

Encyrtus atricollis Dalman, 1820
phrosime (Walker, 1848, *Encyrtus*)
annellus (Thomson, 1876, *Litomastix*)

##### Distribution

England, Ireland

#### Ageniaspis fuscicollis

(Dalman, 1820)

Encyrtus fuscicollis Dalman, 1820
cyanocephalus (Bouché‚ 1834, *Pteromalus*)
cyanocephalus (Goureau, 1847, *Encyrtus*)
praysincola Silvestri, 1907

##### Distribution

England, Ireland

#### Ageniaspis testaceipes

(Ratzeburg, 1848)

Encyrtus testaceipes Ratzeburg, 1848
nepticulae (Mayr, 1876, *Holcothorax*)
vellutatus (Askew, 1983, *Holcothorax*)

##### Distribution

England, Wales

#### Aphycoides

Mercet, 1921

PLESIOMICROTERYS Ishii, 1928
CURBITUS Hoffer, 1957

#### Aphycoides clavellatus

(Dalman, 1820)

Encyrtus clavellatus Dalman, 1820
corybas (Walker, 1837, *Encyrtus*)
liriope (Walker, 1837, *Encyrtus*)
ilithyia (Walker, 1838, *Encyrtus*)
mysus (Walker, 1838, *Encyrtus*)
alycoeus (Walker, 1848, *Encyrtus*)
cephalotes (Ratzeburg, 1852, *Encyrtus*)
radialis (Thomson, 1876, *Microterys*)
physokermis (Girault, 1916, *Holcencyrtus*)
merceti Ferrière, 1953
viridescens (Hoffer, 1957, *Curbitus*)

##### Distribution

England

#### Aphycoides cypris

(Walker, 1838)

Encyrtus cypris Walker, 1838

##### Distribution

England, Ireland

#### Aphycus

Mayr, 1876

APHYCOIDEUS Williams, 1916
WATERSTONIA Mercet, 1917

#### Aphycus apicalis

(Dalman, 1820)

Encyrtus apicalis Dalman, 1820
albicornis Timberlake, 1916
fuliginosa (Compere & Annecke, 1961, *Waterstonia*)

##### Distribution

England, Ireland

#### Aphycus hederaceus

(Westwood, 1837)

Encyrtus hederaceus Westwood, 1837

##### Distribution

England, Ireland

#### Arrhenophagus

Aurivillius, 1888

MYMARIELLA Risbec, 1951

#### Arrhenophagus chionaspidis

Aurivillius, 1888

diaspidis (Ashmead, 1900, *Coccobius*)
parlatoreae (Risbec, 1951, *Mymariella*)
diaspidiatus Agarwal, 1963
intermedius Blanchard, 1964

##### Distribution

England

#### Aschitus

Mercet, 1921

ANICETELLUS Szelényi, 1972
VIGGIANIA Trjapitzin, 1972

#### Aschitus aeneiventris

(Walker, 1837)

Encyrtus aeneiventris Walker, 1837
micropterus (Mercet, 1921, *Encyrtus*)
usticorne (Erdös, 1955, *Metallon*)
nikolskajae (Erdös, 1955, *Microterys*)

##### Distribution

England, Scotland

#### Aschitus annulatus

Erdös, 1957

##### Distribution

England

#### Aschitus barbarus

(Dalman, 1820)

Encyrtus barbarus Dalman, 1820

##### Distribution

Ireland

#### Aschitus carpathicus

(Hoffer, 1958)

Paraphaenodiscus carpathicus Hoffer, 1958

##### Distribution

Scotland

##### Notes

Added by Jensen (1988)

#### Aschitus jalysus

(Walker, 1837)

Encyrtus jalysus Walker, 1837
distinctus (Hoffer, 1953, *Paraphaenodiscus*)

##### Distribution

England

#### Aschitus madyes

(Walker, 1837)

Encyrtus madyes Walker, 1837
mariae (Hoffer, 1953, *Paraphaenodiscus*)
javorinensis (Hoffer, 1958, *Paraphaenodiscus*)

##### Distribution

England

#### Aschitus zarina

(Walker, 1837)

Encyrtus zarina Walker, 1837
calonotus (Mercet, 1921, *Encyrtus*)
rogenhoferi (Mayr, 1876, *Encyrtus*)
rhizococci (Trjapitzin, 1978, *Trichomasthus*)

##### Distribution

England, Wales, Ireland

#### Baeocharis

Mayr, 1876

SPHAEROPISTHUS Thomson, 1876

#### Baeocharis pascuorum

Mayr, 1876

flavoscutatus (Six, 1876, *Encyrtus*)
pascuorum (Thomson, 1876, *Sphaeropisthus*)

##### Distribution

England, Scotland, Ireland

#### Blastothrix

Mayr, 1876

#### Blastothrix brittanica

Girault, 1917

anomala Sugonjaev, 1960

##### Distribution

England

#### Blastothrix erythrostetha

(Walker, 1847)

Encyrtus erythrostethus Walker, 1847
clara Nikol'skaya, 1952

##### Distribution

England

#### Blastothrix longipennis

Howard, 1881

confusa Erdös, 1959

##### Distribution

England

#### Blastothrix sericea

(Dalman, 1820)

Encyrtus sericeus Dalman, 1820
sericans (Dalman, 1820, *Encyrtus*)
saccas (Walker, 1851, *Encyrtus*)
coryli Alam, 1961

##### Distribution

England, Ireland

#### Blastothrix truncatipennis

(Ferrière, 1955)

Microterys truncatipennis Ferrière, 1955
tatricus (Erdös, 1955, *Microterys*)
pragensis Hoffer, 1963
trichomasthoides (Hoffer, 1965, *Apterencyrtus*)
minutus (Bakkendorf, 1965, *Microterys*)
trichomasthoides (Hoffer, 1965, *Zaomma*)
trjapitzini Sugonjaev, 1976

##### Distribution

England

#### Bothriothorax

Ratzeburg, 1844

TRIMORPHOCERUS Dahlbom, 1857

#### Bothriothorax altensteinii

Ratzeburg, 1844

##### Notes

Added by Trjapitzin (1989). Listed as a tentative synonym of *B. paradoxus* by Bouček and Graham (1978).

#### Bothriothorax aralius

(Walker, 1837)

Encyrtus aralius Walker, 1837
eupales (Walker, 1837, *Encyrtus*)

##### Distribution

England, Ireland

#### Bothriothorax arceanus

(Walker,1837)

Encyrtus arceanus Walker,1837

##### Distribution

England

##### Notes

Listed as a tentative synonym of *B. serratellus* by Bouček and Graham (1978).

#### Bothriothorax clavicornis

(Dalman, 1820)

Encyrtus clavicornis Dalman, 1820
conformis Thomson, 1876

##### Distribution

England, Wales, Ireland

#### Bothriothorax intermedius

Claridge, 1964

##### Distribution

England

#### Bothriothorax paradoxus

(Dalman, 1820)

Encyrtus paradoxus Dalman, 1820
nicippe (Walker, 1840, *Encyrtus*)

##### Distribution

England

#### Bothriothorax serratellus

(Dalman, 1820)

Encyrtus serratellus Dalman, 1820

##### Distribution

England

#### Bothriothorax trichops

Thomson, 1876

##### Distribution

England

#### Ceballosia

Mercet, 1921

#### Ceballosia dusmeti

Mercet, 1921

##### Distribution

England

#### Cerapterocerus

Westwood, 1833

JURINIA Costa, 1839
TELEGRAPHUS Ratzeburg, 1848

#### Cerapterocerus mirabilis

Westwood, 1833

anebus (Walker, 1837, *Encyrtus*)
platicera (Costa, 1839, *Jurinia*)
mirabilicornis (Förster, 1841, *Encyrtus*)
maculipennis (Ratzeburg, 1848, *Telegraphus*)
multiradiatus Thomson, 1876

##### Distribution

England

#### Cerchysiella

Girault, 1914

ARATUS Howard, 1897 preocc.
ERICYDNELLA Girault, 1915
MIRRENCYRTUS Girault, 1915
ZETETICONTUS Silvestri, 1915
MIMENCYRTUS Girault, 1923
ARATISCUS Ghesquière, 1946
PROLITOMASTIX Hoffer, 1954

#### Cerchysiella centennalis

(Erdös, 1955)

Zeteticontus centennalis Erdös, 1955

##### Distribution

England

#### Cerchysiella laeviscuta

(Thomson, 1876)

Cerchysiella laeviscuta *Microterys ?laeviscuta* Thomson, 1876

##### Notes

Listed as British by Bouček and Graham (1978) but this requires confirmation

#### Cerchysiella planiscutellum

(Mercet, 1921)

Zeteticontus planiscutellum Mercet, 1921
laeviscuta (Erdös, 1946, *Trichomasthus*) preocc.
vestonicensis (Hoffer, 1954, *Prolitomastix*)

##### Distribution

England

#### Cerchysius

Westwood, 1832

#### Cerchysius subplanus

(Dalman, 1820)

Encyrtus subplanus Dalman, 1820
urocerus (Dalman, 1820, *Encyrtus*)
stigmaticalis Westwood, 1832
melanopus (Walker, 1837, *Encyrtus*)
caudatus (Förster, 1841, *Encyrtus*)

##### Distribution

England, Wales, Ireland

#### Cercobelus

Walker, 1842

#### Cercobelus jugaeus

(Walker, 1837)

Encyrtus jugaeus Walker, 1837
parus (Walker, 1837, *Encyrtus*)

##### Distribution

England, Wales, Scotland, Ireland, Isle of Man

#### Cheiloneurus

Westwood, 1833

CHRYSOPOPHAGUS Ashmead, 1894
BLATTICIDA Ashmead, 1904
SARONOTUM Perkins, 1906
ECHTHROGONATOPUS Perkins, 1906
CRISTATITHORAX Girault, 1911
CHRYSOPOPHAGOIDES Girault, 1915
EPICHEILONEURUS Girault, 1915
EUSEMIONELLA Girault, 1915
EUSEMIONOPSIS Girault, 1918
PROCHEILONEURUS Girault, 1920
AULONOPS Timberlake, 1922
HYPERGONATOPUS Timberlake, 1922
RAPHAELANA Girault, 1926
BEKILYIA Risbec, 1952
METACHEILONEURUS Hoffer, 1957

#### Cheiloneurus argentifer

(Walker, 1837)

Encyrtus argentifer Walker, 1837

##### Distribution

Scotland, Ireland

#### Cheiloneurus claviger

Thomson, 1876

graeffei Ruschka, 1923

##### Distribution

England

#### Cheiloneurus elegans

(Dalman, 1820)

Encyrtus elegans Dalman, 1820

##### Distribution

England

#### Cheiloneurus glaphyra

(Walker, 1837)

Encyrtus glaphyra Walker, 1837

##### Distribution

England

##### Notes

Listed as a tentative synonym of *C. elegans* by Bouček and Graham (1978)

#### Cheiloneurus paralia

(Walker, 1837)

Encyrtus paralia Walker, 1837
eriococci Alam, 1957
formosus (Boheman, 1852, *Encyrtus*)
mongolicus Szelény, 1971

##### Distribution

England

##### Notes

**Figure 2.**
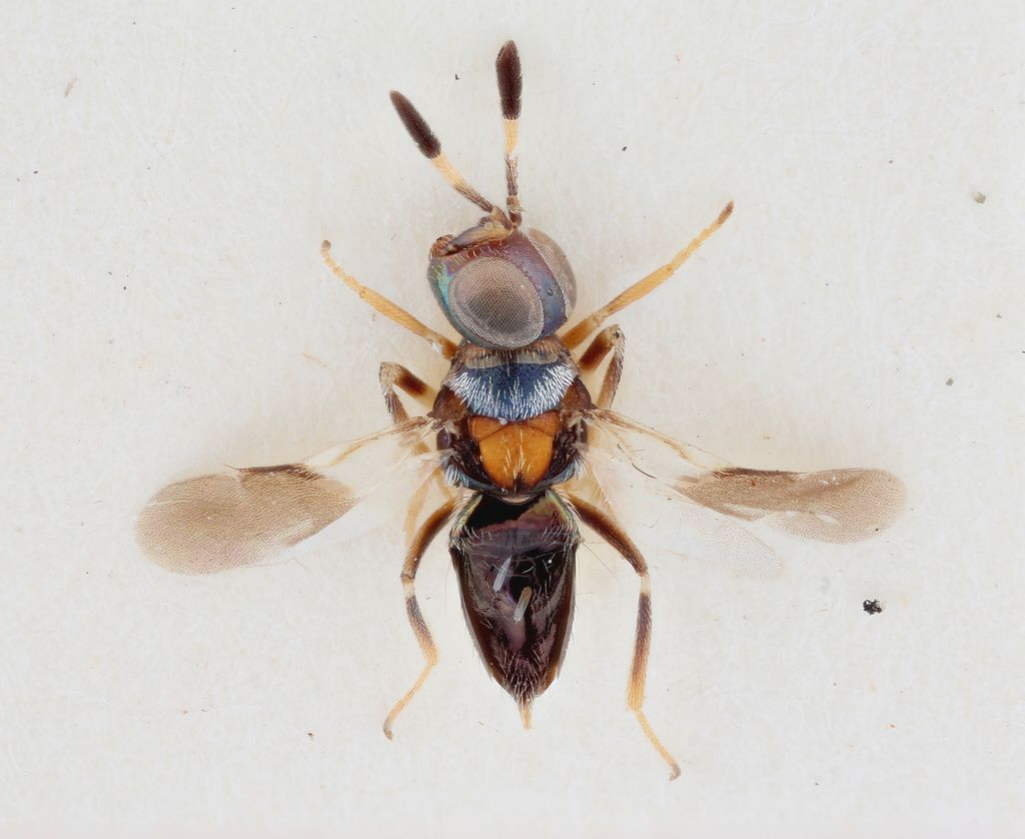
Encyrtidae, Encyrtinae: *Cheiloneurus paralia* (Walker) female, BMNH specimen BMNH(E)1414554 [06/1970; Lewknor; Oxfordshire, coll. Bouček] (photo K. McCormack)

See Fig. 2 for habitus

#### Cheiloneurus submuticus

Thomson, 1876

moestus (Hoffer, 1957, *Metacheiloneurus*)

##### Distribution

England

#### Choreia

Westwood, 1833

CHOREASPIS Hoffer, 1953

#### Choreia inepta

(Dalman, 1820)

Encyrtus ineptus Dalman, 1820
nigroaenea Westwood, 1833

##### Distribution

England, Ireland

#### Coelopencyrtus

Timberlake, 1919

NESENCYRTUS Timberlake, 1919
EPAENASOMYIA Girault, 1919
GIRAULTELLA Gahan & Fagan, 1923
BATRACHENCYRTUS Jansson, 1957
LYMANERA Szelényi, 1972

#### Coelopencyrtus arenarius

(Erdös, 1957)

Adelencyrtus arenarius Erdös, 1957
malyshevi Trjapitzin, 1960
manningeri (Szelényi, 1972, *Oobius*)

##### Distribution

England

#### Coelopencyrtus callidii

(Jansson, 1957)

Batrachencyrtus callidii Jansson, 1957
cephalotus Hedqvist, 1973

##### Distribution

England

#### Copidosoma

Ratzeburg, 1844

LITOMASTIX Thomson, 1876
BERECYNTUS Howard, 1898
PARAPSILOPHRYS Howard, 1898
PENTACNEMUS Howard, 1892
PSEUDENCYRTELLA Girault, 1913
PARACAENOCERCUS Girault, 1915
ZAOMENCYRTUS Girault, 1915
PARACOPIDOSOMOPSIS Girault, 1916
VERDUNIA Mercet, 1917
LIMASTOTIX Mercet, 1921
LITOMASTIELLUS Mercet, 1921
PARALITOMASTIX Mercet, 1921
ANGELICONANA Girault, 1922
NEOCOPIDOSOMA Ishii, 1923
PARASTEROPAEUS Girault, 1923
MESOCOPIDOSOMYIIA Girault, 1925
MESENCYRTUS Timberlake, 1941
BERECYNTISCUS Ghesquière, 1946
ARRENOCLAVUS Doutt, 1948

#### Copidosoma agrotis

(Fonscolombe, 1832)

Cynips agrotis Fonscolombe, 1832
auricollis (Thomson, 1876, *Litomastix*)
peregrinus (Mercet, 1921, *Litomastix*)

##### Distribution

England, Wales, Ireland

#### Copidosoma aithyia

(Walker, 1837)

Encyrtus aithyia Walker, 1837
hydramon (Walker, 1848, *Eupelmus*)
kriechbaumeri Mayr, 1876
phalaenarum (Thomson, 1876, *Litomastix*)
quercicola (Mercet, 1921, *Litomastix*)
salicina (Erdös, 1956, *Litomastix*)

##### Distribution

England, Wales

##### Notes

Omitted by Bouček and Graham (1978)

#### Copidosoma albipes

(Westwood, 1837)

Encyrtus albipes Westwood, 1837
citripes Ratzeburg, 1852
innocuellae Barron, 1970

##### Distribution

England, Wales

#### Copidosoma anceus

(Walker, 1837)

Encyrtus anceus Walker, 1837
caniculare Mercet, 1921

##### Distribution

England, Scotland, Ireland

##### Notes

Omitted by Bouček and Graham (1978)

#### Copidosoma ancharus

(Walker, 1837)

Encyrtus ancharus Walker, 1837
vulso (Walker, 1846, *Cerchysius*)
nanellae Silvestri, 1923
globiceps Erdös, 1955
lembolovicum Trjapitzin, 1994
tortricis Waterston, 1920

##### Distribution

England

#### Copidosoma aretas

(Walker, 1838)

Encyrtus aretas Walker, 1838
telesto (Walker, 1838, *Encyrtus*)
suspectus (Bakkendorf, 1965, *Litomastix*)

##### Distribution

England, Scotland, Wales, Ireland

#### Copidosoma babas

(Walker, 1837)

Encyrtus babas Walker, 1837
machaeras (Walker, 1837, *Encyrtus*)
boreale Hoffer, 1970

##### Distribution

England, Wales, Ireland

#### Copidosoma boucheanum

Ratzeburg, 1844

hilaris (Ratzeburg, 1852, *Encyrtus*)
cultriformis (Mayr, 1876, *Encyrtus*)
cultriforme Hoffer, 1957

##### Distribution

England

##### Notes

Added by Springate and Noyes (1990)

#### Copidosoma cervius

(Walker, 1846)

Encyrtus cervius Walker, 1846
truncatulus (Thomson, 1876, *Litomastix*)
moldavica (Hoffer, 1957, *Litomastix*)
tvediensis (Bakkendorf, 1965, *Litomastix*)

##### Distribution

England, Scotland, Wales, Ireland

#### Copidosoma chalconotum

(Dalman, 1820)

Encyrtus chalconotus Dalman, 1820
mitreus (Walker, 1837, *Encyrtus*)
phithra (Walker, 1837, *Encyrtus*)

##### Distribution

England, Scotland, Ireland

#### Copidosoma cuproviride

Springate & Noyes, 1990

##### Distribution

Wales

##### Notes

Added by Springate and Noyes (1990)

#### Copidosoma cyaneum

Hoffer, 1970

##### Distribution

England, Wales

##### Notes

Added by Guerrieri and Noyes (2005)

#### Copidosoma dius

(Walker, 1837)

Encyrtus dius Walker, 1837
gellius (Walker, 1837, *Encyrtus*)
fundulus (Walker, 1846, *Encyrtus*)
proecia (Walker, 1846, *Encyrtus*)
castellanum Mercet, 1921
matritense Mercet, 1921
igneum Bakkendorf, 1965
laevigatum Erdös, 1957

##### Distribution

England, Scotland, Wales, Ireland

#### Copidosoma fadus

(Walker, 1838)

Encyrtus fadus Walker, 1838
pragense Novicky, 1925

##### Distribution

England

##### Notes

Omitted by Bouček and Graham (1978)

#### Copidosoma filicorne

(Dalman, 1820)

Encyrtus filicornis Dalman, 1820
geniculatus (Dalman, 1820, *Encyrtus*)
didius (Walker, 1837, *Encyrtus*)
montanum Mercet, 1921
glandiferellae Barron & Bisdee, 1984

##### Distribution

England, Scotland, Wales, Ireland

#### Copidosoma flagellare

(Dalman, 1820)

Encyrtus flagellaris Dalman, 1820
tegularis (Ratzeburg, 1852, *Encyrtus*)

##### Distribution

England, Scotland, Wales, Ireland

#### Copidosoma floridanum

(Ashmead, 1900)

Berecyntus floridanus Ashmead, 1900
japonicum Ashmead, 1904
argentinus (Brèthes, 1913, *Litomastix*)
calypso (Crawford, 1914, *Holcencyrtus*)
javae (Girault, 1917, *Paracopidosomopsis*)
brasiliensis (Brèthes, 1920, *Prionomitus*)
intermedia (Mercet, 1921, *Litomastix*)
walshi (Mercet, 1922, *Litomastix*)
maculata (Ishii, 1928, *Litomastix*)
brethesi (Blanchard, 1936, *Litomastix*)
daccaensis (Mani, 1941, *Litomastix*)
phytometrae (Risbec, 1951, *Paralitomastix*)

##### Distribution

England, Scotland, Wales

##### Notes

Added by Springate and Noyes (1990)​

#### Copidosoma fuscisquama

(Thomson, 1876)

Litomastix fuscisquama Thomson, 1876

##### Distribution

England, Ireland

#### Copidosoma genale

(Thomson, 1876)

Litomastix genalis Thomson, 1876
peninsulare Mercet, 1921

##### Distribution

England, Scotland, Wales, Ireland,

#### Copidosoma iracundum

Erdös, 1957

savsdargi Trjapitzin, 1968

##### Distribution

England, Scotland, Wales

#### Copidosoma peticus

(Walker, 1846)

Encyrtus peticus Walker, 1846
flavomaculatus (Ratzeburg, 1848, *Encyrtus*)
coleophorae Mayr, 1876
triangularis (Thomson, 1876, *Litomastix*)
buyssoni Mayr, 1902
incertum Mercet, 1921
brevicaudae Mercet, 1923
woronieckae Nowicki, 1925
jamansaiense Myartseva, 1983
kisilkumense Myartseva, 1983

##### Distribution

England

#### Copidosoma radnense

Erdös, 1957

crassicorne Hoffer, 1970

##### Distribution

England, Wales, Ireland

##### Notes

Added by Guerrieri and Noyes (2005)

#### Copidosoma ratzeburgi

Mercet, 1921

##### Distribution

England

##### Notes

Added by Guerrieri and Noyes (2005)

#### Copidosoma serricorne

(Dalman, 1820)

Encyrtus serricornis Dalman, 1820
spherus (Walker, 1837, *Encyrtus*)
cidariae Mayr, 1876

##### Distribution

England, Scotland

#### Copidosoma sosares

(Walker, 1837)

Encyrtus sosares Walker, 1837
molos (Walker, 1848, *Encyrtus*)
hartmanni Mayr, 1876
latifrons (Thomson, 1876, *Litomastix*)
pinicola (Mercet, 1921, *Litomastix*)
alexandri (Myartseva, 1979, *Litomastix*)
pastinacella (Logvinovskaya, 1983, *Litomastix*)

##### Distribution

England, Scotland, Wales

#### Copidosoma subalbicorne

(Hoffer, 1960)

Paralitomastix subalbicorne Hoffer, 1960

##### Distribution

England, Wales

#### Copidosoma terebrator

Mayr, 1876

giganteum Hoffer, 1957
bohemicum Hoffer, 1969

##### Distribution

England,

##### Notes

Added by Springate and Noyes (1990)​

#### Copidosoma thebe

(Walker, 1838)

Encyrtus thebe Walker, 1838
camirus (Walker, 1838, *Encyrtus*)
claviger (Mercet, 1921, *Litomastix*)
pulchellus (Mercet, 1921, *Litomastix*)
fulvipes (Erdös, 1960, *Litomastix*)
tibiale Hoffer, 1970

##### Distribution

England, Wales, Ireland

#### Copidosoma truncatellum

(Dalman,1820)

Encyrtus truncatellus Dalman,1820
atheas (Walker, 1837, *Encyrtus*)
aestivalis (Mercet, 1921, *Litomastix*)

##### Distribution

England, Scotland, Wales, Ireland

#### Copidosoma varicorne

(Nees, 1834)

Encyrtus varicornis Nees, 1834
annulata Nikol'skaya, 1952
batorligetensis (Erdös, 1960, *Paralitomastix*)
clavellatus (Erdös, 1960, *Paralitomastix*)
gallaephila (Risbec, 1951, *Paralitomastix*)
sylleptae (Risbec, 1951, *Paralitomastix*)

##### Distribution

England

##### Notes

Omitted by Bouček and Graham (1978) but recorded as British by Chater (1931), latterly by Guerrieri and Noyes (2005)

#### Discodes

Förster, 1856

PHAENODISCUS Förster, 1856

#### Discodes aeneus

(Dalman, 1820)

Encyrtus aeneus Dalman, 1820
melanopterus (Nees, 1834, *Encyrtus*)
statius (Walker, 1850, *Encyrtus*)

##### Distribution

England

#### Discodes anthores

(Walker, 1848)

Encyrtus anthores Walker, 1848
Discodes anthores ?*iophon* (Walker, 1848, *Encyrtus*)

##### Distribution

England, Wales, Ireland

#### Discodes encopiformis

(Walker, 1847)

Encyrtus encopiformis Walker, 1847
cercopiformis (Mayr, 1876, *Phaenodiscus*)

##### Distribution

England

#### Echthroplexiella

Mercet, 1921

#### Echthroplexiella obscura

(Hoffer,1954)

Waterstonia obscura Hoffer,1954

##### Distribution

England, Wales

#### Echthroplexiella tertia

(Hoffer,1954)

Waterstonia tertia Hoffer,1954

##### Distribution

England

#### Echthroplexis

Förster, 1856

CAENOCERCUS Thomson, 1876
BOTHRIENCYRTUS Timberlake, 1919
EUCANTABRIA Mercet, 1921

#### Echthroplexis puncticollis

(Thomson, 1876)

Caenocercus puncticollis Thomson, 1876
azurea (Mercet, 1921, *Eucantabria*)

##### Distribution

England

#### Ectroma

Westwood, 1833

METALLON Walker, 1848
PEZOBIUS Förster, 1860
CONCENTROLINEA Bakkendorf, 1965
IDIOCOCCOPHILUS Tachikawa & Gordh, 1987

#### Ectroma fulvescens

Westwood, 1833

acacallis (Walker, 1848, *Metallon*)
polychromus (Förster, 1860, *Pezobius*)
desertorum (Hoffer, 1953, *Mayridia*)

##### Distribution

England, Wales

#### Ectroma reinhardi

(Mayr, 1876)

Ericydnus reinhardi Mayr, 1876

##### Distribution

England, Scotland

#### Encyrtus

Latreille, 1809

COMYS Förster, 1856
EUCOMYS Förster, 1856
HOWARDIA Dalla Torre, 1897
HOWARDIELLA Dalla Torre, 1898
ALLORHOPOIDEUS Brèthes, 1916
PRORHOPOIDEUS Brèthes, 1921

##### Notes

Doubtfully placed species of *Encyrtus*:

[*antistius* Walker, 1851] - England

[*chaerilus* Walker, 1837, syn. *choerilus* misspelling] - Wales

[*meon* Walker, 1838] - England

[*metharma* Walker, 1846] - England

[*philiscus* Walker, 1851]

[*proculus* Walker, 1846] - England

[*pyttalus* Walker, 1844] (included as a tentative synonym of *Choreia inepta* by Bouček & Graham (1978))

[*teuteus* Walker, 1837] - England

[*tylissos* Walker, 1848] - England]

#### Encyrtus albitarsis

Zetterstedt, 1838

niveitarsis Thomson, 1876

##### Distribution

England

#### Encyrtus infelix

(Embleton, 1902)

Comys infelix Embleton, 1902
hortensis (Girault, 1915, *Eucomys*)
proserpinensis (Girault, 1915, *Eucomys*)
tananarivensis (Risbec, 1952, *Eucomys*)

##### Distribution

England, Scotland

#### Encyrtus infidus

(Rossi, 1790)

Chrysis infidus Rossi, 1790
scutellatus (Swederus, 1795, *Pteromalus*)
obscurus Dalman, 1820
scutellaris Dalman, 1820
scutellaris Fonscolombe, 1832 preocc.
incerta (Nikol'skaya, 1952, *Eucomys*)
hokkaidonis Tachikawa, 1963

##### Distribution

England, Ireland

#### Encyrtus swederi

Dalman, 1820

vitis Curtis, 1832

##### Distribution

England

#### Encyrtus antistius

Walker, 1851

##### Distribution

England

#### Encyrtus chaerilus

Walker, 1837

##### Distribution

Wales

#### Encyrtus meon

Walker, 1838

##### Distribution

England

#### Epitetracnemus

Girault, 1915

ANABROLEPIS Timberlake, 1920

#### Epitetracnemus intersectus

(Fonscolombe, 1832)

Encyrtus intersectus Fonscolombe, 1832
zetterstedtii (Westwood, 1837, *Encyrtus*)
dendripennis (Ratzeburg, 1852, *Encyrtus*)
pictipennis (Six, 1867, *Eupelmus*)
extranea (Timberlake, 1920, *Anabrolepis*)

##### Distribution

England

#### Eusemion

Dahlbom, 1857

#### Eusemion cornigerum

(Walker, 1838)

Encyrtus corniger Walker, 1838
tsukumiense Tachikawa, 1957

##### Distribution

England, Scotland, Wales

#### Ginsiana

Erdös & Novicky, 1955

TATRANUS Hoffer, 1963
POGLOTHYREA Szelényi, 1972

#### Ginsiana carpetana

(Mercet, 1921)

Microterys carpetanus Mercet, 1921
matranum (Erdös, 1957, *Copidosoma*)
brevicauda (Hoffer, 1963, *Ooencyrtus*)

##### Distribution

England

#### Ginsiana praepannonica

(Erdös, 1957)

Copidosoma praepannonicum Erdös, 1957
terebrator (Hoffer, 1963, *Ooencyrtus*)

##### Distribution

England

#### Habrolepis

Förster, 1856

GYMNONEURA Risbec, 1951

#### Habrolepis dalmanni

(Westwood, 1837)

Encyrtus dalmanni Westwood, 1837
nubilipennis (Walker, 1838, *Encyrtus*)
pulchris Botoc, 1962

##### Distribution

England

#### Helegonatopus

Perkins, 1906

CHALCERINYS Perkins, 1906
SCHEDIOIDES Mercet, 1919
EUCHALCERINYS Timberlake, 1922
HAZMBURKIA Hoffer, 1954
MASENCYRTUS Hoffer, 1960
PALUDENCYRTUS Hoffer, 1965

#### Helegonatopus citripes

(Erdös, 1957)

Ginsiana citripes Erdös, 1957
concupiens (Hoffer, 1960, *Masencyrtus*)

##### Distribution

England

#### Helegonatopus dimorphus

(Hoffer, 1954)

Hazmburkia dimorpha Hoffer, 1954

##### Distribution

Scotland

#### Heterococcidoxenus

Ishii, 1940

MICROSPHENUS Kerrich, 1963

#### Heterococcidoxenus schlechtendali

(Mayr, 1876)

Bothriothorax schlechtendali Mayr, 1876

##### Distribution

England

#### Homalotyloidea

Mercet, 1921

APHYCASPIS Hoffer, 1954

#### Homalotyloidea dahlbomii

(Westwood, 1837)

Encyrtus dahlbomii Westwood, 1837
latiscapus (Masi, 1919, *Homalotylus*)

##### Distribution

England, Ireland

#### Homalotyloidea erginus

(Walker, 1837)

Encyrtus erginus Walker, 1837
hispanicus (Mercet, 1921, *Homalotylus*)

##### Distribution

England

#### Homalotyloidea nowickyi

Hoffer, 1957

##### Distribution

England

#### Homalotylus

Mayr, 1876

NOBRIMUS Thomson, 1876
MENDOZANIELLA Brèthes, 1913
HEMAENASIOIDEA Girault, 1916
ANISOTYLUS Timberlake, 1919
LEPIDAPHYCUS Blanchard, 1936
NEOAENASIOIDEA Agarwal, 1966

#### Homalotylus flaminius

(Dalman, 1820)

Encyrtus flaminius Dalman, 1820

##### Distribution

England

#### Homalotylus hemipterinus

(De Stefani, 1898)

Phaenodiscus hemipterinus De Stefani, 1898
microgaster Girault, 1917
orci Girault, 1917

##### Notes

BMNH, det. Noyes, added here

#### Isodromus

Howard, 1887

PARATANEOSTIGMA Girault, 1915

#### Isodromus flaviceps

(Dalman, 1820)

Encyrtus flaviceps Dalman, 1820

##### Distribution

England, Wales

##### Notes

Added by Askew and Shaw (1979b)

#### Isodromus vinulus

(Dalman, 1820)

Encyrtus vinulus Dalman, 1820
intermedius (Boheman, 1852, *Encyrtus*)

##### Distribution

England,

#### Ixodiphagus

Howard, 1907

HUNTERELLUS Howard, 1908
AUSTRALZAOMMA Girault, 1925

#### Ixodiphagus hookeri

(Howard, 1908)

Hunterellus hookeri Howard, 1908
caniphila (Risbec, 1951, *Habrolepis*)
caucurtei Buysson, 1912

##### Distribution

England

#### Lakshaphagus

Mahdihassan, 1931

CHEILONICETUS Shafee, Alam & Agarwal, 1975

#### Lakshaphagus laevis

(Erdös, 1957)

Mayrencyrtus laevis Erdös, 1957

##### Distribution

England

#### Lamennaisia

Girault, 1922

MERCETENCYRTUS Trjapitzin, 1963
SABIRELLA Agarwal, Agarwal & Khan, 1980
NEGENIASPIDIUS Trjapitzin, 1982

#### Lamennaisia ambigua

(Nees, 1834)

Encyrtus ambiguus Nees, 1834
nasidius (Walker, 1846, *Encyrtus*)
acratos (Walker, 1848, *Encyrtus*)
dubius (Howard, 1889, *Encyrtus*)
dubiosus (Dalla Torre, 1898, *Encyrtus*)
tarsalis (Girault, 1916, *Habrolepoidea*)
indica (Agarwal, Agarwal & Khan, 1980, *Sabirella*)
longiscapus (Fatima & Shafee, 1994, *Adelencyrtus*)

##### Distribution

England

#### Lamennaisia nobilis

(Nees, 1834)

Encyrtus nobilis Nees, 1834
pretiosus (Mercet, 1921, *Coccidencyrtus*)

##### Distribution

England

#### Leiocyrtus

Erdös & Novicky, 1955

#### Leiocyrtus clavatus

Erdös & Novicky, 1955

##### Distribution

England, Wales

#### Mahencyrtus

Masi, 1917

TYNDARICHOIDES Mercet, 1921
PROTYNDARICHUS Mercet, 1923

#### Mahencyrtus comara

(Walker, 1837)

Encyrtus comara Walker, 1837
metallicus (Mercet, 1921, *Tyndarichoides*)
balatonicus (Erdös, 1957, *Protyndarichus*)
britannicus (Alam, 1957, *Protyndarichus*)
graminum (Erdös, 1957, *Protyndarichus*)

##### Distribution

England, Wales, Ireland

#### Mayrencyrtus

Hincks, 1944

LIOTHORAX Mayr, 1876 preocc.

#### Mayrencyrtus imandes

(Walker, 1837)

Encyrtus imandes Walker, 1837
maja (Hoffer, 1957, *Lyka*)

##### Distribution

England

#### Mayridia

Mercet, 1921

SUPERPRIONOMITUS Mercet, 1921
INDOENCYRTUS Hayat & Verma, 1978

#### Mayridia alcmon

(Walker,1848)

Encyrtus alcmon Walker,1848
subfuscipennis Erdös, 1957

##### Distribution

England

#### Mayridia myrlea

(Walker,1838)

Encyrtus myrlea Walker,1838
bifasciatellus (Mayr, 1876, *Encyrtus*)

##### Distribution

England

#### Mayridia procera

(Mercet, 1921)

Superprionomitus procerus Mercet, 1921

##### Distribution

England, Isle of Man

#### Metaphycus

Mercet, 1917

AENASIOIDEA Girault, 1911 suppressed
TYNDARICHOIDES Girault, 1920
EUAPHYCUS Mercet, 1921
MERCETIELLA Dozier, 1926
OAPHYCUS Girault, 1932
ERYTHRAPHYCUS Compere, 1947
MELANAPHYCUS Compere, 1947
ANAPHYCUS Sugonjaev, 1960
MESAPHYCUS Sugonjaev, 1960
NOTOENCYRTUS De Santis, 1964
XENAPHYCUS Trjapitzin, 1978
AENIGMAPHYCUS Sharkov & Voynovich, 1988

#### Metaphycus alami

Tachikawa, 1968

eriococci Alam, 1957

##### Distribution

England

#### Metaphycus annasor

Guerrieri & Noyes, 2000

##### Distribution

England

##### Notes

Added by Guerrieri and Noyes (2000)

#### Metaphycus artinix

Guerrieri & Noyes, 2000

##### Distribution

Scotland

##### Notes

Added by Guerrieri and Noyes (2000)

#### Metaphycus asterolecanii

(Mercet, 1923)

Aphycus asterolecanii Mercet, 1923
variolosus (Alam, 1957, *Euaphycus*)

##### Distribution

England

#### Metaphycus ater

(Mercet, 1925)

Euaphycus ater Mercet, 1925
nigritus (Mercet, 1921, *Aphycus*)

##### Distribution

England, Scotland, Wales

#### Metaphycus bulgariensis

Sugonjaev, 1976

##### Distribution

Wales

##### Notes

Added by Guerrieri and Noyes (2000)

#### Metaphycus chermis

(Fonscolombe, 1832)

Cinips chermis Fonscolombe, 1832
mayri (Timberlake, 1916, *Aphycus*)
fulvifrons (Walker, 1838, *Encyrtus*)

##### Distribution

England, Scotland, Wales, Ireland

#### Metaphycus ecares

Guerrieri & Noyes, 2000

##### Distribution

England

##### Notes

Added by Guerrieri and Noyes (2000)

#### Metaphycus flavovarius

(Mercet, 1921)

Paraphycus flavovarius Mercet, 1921
vigil (Erdös, 1957, *Paraphycus*)

##### Distribution

England, Wales, Ireland

#### Metaphycus hanstediensis

Bakkendorf, 1965

##### Distribution

Scotland

##### Notes

Added by Guerrieri and Noyes (2000)

#### Metaphycus helvolus

(Compere, 1926)

Aphycus helvolus Compere, 1926

##### Notes

Introduced into greenhouses for biological control

#### Metaphycus insidiosus

(Mercet, 1921)

Aphycus insidiosus Mercet, 1921
taxi Alam, 1957

##### Distribution

England, Wales

#### Metaphycus melanostomatus

(Timberlake, 1916)

Aphycus melanostomatus Timberlake, 1916

##### Distribution

England, Scotland, Wales, Ireland

#### Metaphycus nadius

(Walker, 1838)

Encyrtus nadius Walker, 1838
syllaeus (Walker, 1838, *Encyrtus*)
pinicola (Mercet, 1917, *Aphycus*)
intermedius (Mercet, 1925, *Euaphycus*)
callunae (Alam, 1957, *Euaphycus*)
duplus (Chumakova, 1961, *Euaphycus*)

##### Distribution

England, Wales

#### Metaphycus pappus

(Walker, 1838)

Encyrtus pappus Walker, 1838
notatus Hoffer, 1954

##### Distribution

England, Wales, Ireland

#### Metaphycus petitus

(Walker, 1851)

Encyrtus petitus Walker, 1851
erythraeus Hoffer, 1954

##### Distribution

England, Wales, Ireland

#### Metaphycus piceus

Hoffer, 1954

##### Distribution

England

##### Notes

Added by Guerrieri and Noyes (2000)

#### Metaphycus punctipes

(Dalman, 1820)

Encyrtus punctipes Dalman, 1820
phaeus (Erdös, 1955, *Aphycus*)
salicis Erdös, 1956

##### Distribution

England, Wales, Ireland

##### Notes

Added by Guerrieri and Noyes (2000)

#### Metaphycus stagnarum

Hoffer, 1954

melanus Sugonjaev, 1960

##### Distribution

England, Wales, Isle of Man

##### Notes

Added by Guerrieri and Noyes (2000)

#### Metaphycus zebratus

(Mercet, 1917)

Aphycus zebratus Mercet, 1917
parvus (Mercet, 1921, *Aphycus*)

##### Distribution

England, Wales

#### Microterys

Thomson, 1876

SCEPTROPHORUS Förster, 1856
APENTELICUS Fullaway, 1913
PARAPHAENODISCOIDES Mercet, 1921
BIROUS Erdös & Novicky, 1955

##### Notes

Species of *Microterys* removed from the British and Irish list:

[*hortulanus* Erdös, 1956]

Recorded as new to Britain by Japoshvili and Noyes (2006) on the basis of a Welsh specimen but this was probably a misidentification.

#### Microterys anomalus

(Erdös & Novicky, 1955)

Birous anomalus Erdös & Novicky, 1955

#### Microterys apicipennis

Bakkendorf, 1965

##### Distribution

England

##### Notes

Added by Springate and Noyes (1990)

#### Microterys bellae

Trjapitsin, 1968

##### Distribution

England

##### Notes

BMNH, det. Noyes, added here

#### Microterys chalcostomus

(Dalman, 1820)

Encyrtus chalcostomus Dalman, 1820

##### Distribution

England

#### Microterys ferrugineus

(Nees, 1834)

Encyrtus ferrugineus Nees, 1834

##### Distribution

England

#### Microterys interpunctus

(Dalman, 1820)

Encyrtus interpunctus Dalman, 1820

##### Distribution

England

##### Notes

Added by Springate and Noyes (1990)

#### Microterys lunatus

(Dalman, 1820)

Encyrtus lunatus Dalman, 1820

##### Distribution

England

#### Microterys masii

Silvestri, 1919

##### Distribution

England

#### Microterys nietneri

(Motschulsky, 1859)

Encyrtus nietneri Motschulsky, 1859

##### Distribution

England

##### Notes

BMNH, det. Noyes, added here - only found in greenhouses

#### Microterys polylaus

(Walker, 1846)

Encyrtus polylaus Walker, 1846

##### Distribution

England

#### Microterys seyon

Guerrieri, 1996

##### Distribution

England, Wales

##### Notes

Added by Guerrieri (1996)

#### Microterys subcupratus

(Dalman, 1820)

Encyrtus subcupratus Dalman, 1820

##### Distribution

Ireland

##### Notes

Omitted by Bouček and Graham (1978)as only recorded from Ireland (O'Connor et al. 2000), not Britain

#### Microterys sylvius

(Dalman, 1820)

Encyrtus sylvius Dalman, 1820
zephyrinus (Dalman, 1820, *Encyrtus*)
titiani Girault, 1917

##### Distribution

England, Ireland, Isle of Man

#### Microterys tessellatus

(Dalman, 1820)

Encyrtus tessellatus Dalman, 1820

##### Distribution

England, Scotland, Wales, Ireland

#### Ooencyrtus

Ashmead, 1900

ECHTHRODRYINUS Perkins, 1906
ECTOPIOGNATHA Perkins, 1906
SCHEDIUS Howard, 1910
TETRACNEMELLA Girault, 1915
XESMATIA Timberlake, 1920
PSEUDOLITOMASTIX Risbec, 1954
OOENCYRTELLUS Hoffer, 1963

#### Ooencyrtus brunneipes

Noyes, 1978

##### Distribution

England

#### Parablastothrix

Mercet, 1917

CALOMETOPIA Mercet, 1921

#### Parablastothrix vespertina

Mercet, 1917

##### Distribution

England, Wales

#### Parablatticida

Girault, 1915

HOLANUSIA Girault, 1915
GENIASPIDIUS Masi, 1917
SYMPHYCUS Masi, 1917
AMAURILYMA Graham, 1958
DESOBIUS Noyes, 1980

#### Parablatticida brevicornis

(Dalman, 1820)

Encyrtus brevicornis Dalman, 1820
gabestus (Walker, 1838, *Encyrtus*)

##### Distribution

England, Ireland

#### Parechthrodryinus

Girault, 1916

#### Parechthrodryinus paralourgos

Springate & Noyes, 1990

##### Distribution

England

##### Notes

Added by Springate and Noyes (1990)

#### Prionomastix

Mayr, 1876

APRIONOMASTIX Girault, 1913
CHESTOMORPHA Ashmead, 1900
LIOCARUS Thomson, 1876

#### Prionomastix morio

(Dalman, 1820)

Encyrtus morio Dalman, 1820

##### Distribution

England

#### Prionomitus

Mayr, 1876

#### Prionomitus mitratus

(Dalman, 1820)

Encyrtus mitratus Dalman, 1820
chlorinus (Dalman, 1820, *Encyrtus*)
coniferae (Walker, 1837, *Encyrtus*)

##### Distribution

England, Scotland, Ireland

#### Prionomitus tiliaris

(Dalman, 1820)

Encyrtus tiliaris Dalman, 1820

##### Distribution

England, Ireland

#### Prochiloneurus

Silvestri, 1915

ACHRYSOPOPHAGUS Girault, 1915
PARACHRYSOPOPHAGUS Agarwal, 1965
NEOPROCHILONEURUS Viggiani, 1966
PROCHILONEUROIDES Hayat, Alam & Agarwal, 1975

#### Prochiloneurus bolivari

Mercet, 1919

stylatus (Ruschka, 1923, *Chiloneurus*)
fukudai (Tachikawa, 1971, *Neoprochiloneurus*)

##### Distribution

England, Wales

#### Protyndarichoides

Noyes, 1980

#### Protyndarichoides aligarhensis

(Fatma & Shafee, 1985)

Parasyrpophagus aligarhense Fatma & Shafee, 1985

##### Distribution

England

##### Notes

Added by Springate and Noyes (1990)

#### Pseudencyrtus

Ashmead, 1900

#### Pseudencyrtus idmon

(Walker, 1848)

Encyrtus idmon Walker, 1848
idya (Walker, 1848, *Encyrtus*)
claviger (Thomson, 1876, *Microterys*)

##### Distribution

England, Ireland

#### Pseudencyrtus misellus

(Dalman, 1820)

Encyrtus misellus Dalman, 1820
tennes (Walker, 1837, *Encyrtus*)
dubius Erdös, 1957

##### Distribution

England

#### Pseudencyrtus salicisstrobili

(Linnaeus, 1758)

Cynips salicisstrobili Linnaeus, 1758
sitalces (Walker, 1837, *Encyrtus*)

##### Distribution

England

#### Pseudococcobius

Timberlake, 1916

AUSTRALRHOPOIDEUS Girault, 1926
PEZAPHYCUS Novicky, 1926

#### Pseudococcobius obenbergeri

(Novicky, 1926)

Pezaphycus obenbergeri Novicky, 1926
pannonicus (Erdös, 1946, *Aphycus*)
antennalis (Alam, 1957, *Aphycus*)
brachypterus (Alam, 1957, *Aphycus*)

##### Distribution

England

#### Tachinaephagus

Ashmead, 1904

PHAENODISCOIDES Girault, 1915
TACHINACPHAGUS Girault, 1917
AUSTRALENCYRTUS Johnston & Tiegs, 1921
AUSTRALOMALOTYLUS Risbec, 1956

#### Tachinaephagus zealandicus

Ashmead, 1904

australiensis Girault, 1917
giraulti (Johnston & Tiegs, 1921, *Australencyrtus*)
fulvoventralis (Dodd, 1921, *Stenoterys*)
rageaui (Risbec, 1956, *Australomalotylus*)

##### Distribution

England

##### Notes

BMNH, det. Noyes, added here. Originally recorded as new to Wales by Japoshvili and Noyes (2006) but this was based on G. Japoshvili confusing ‘New South Wales’ with ‘Wales’ on label data. Strangely, this species has subsequently been found by J. Noyes in England.

#### Pseudorhopus

Timberlake, 1926

AMERICENCYRTUS Sugonjaev, 1989

#### Pseudorhopus testaceus

(Ratzeburg, 1848)

Encyrtus testaceus Ratzeburg, 1848
britannicus Alam, 1957

##### Distribution

England

#### Psyllaephagus

Ashmead, 1900

MIROCERUS Ashmead, 1904
CALOCERINELOIDES Girault, 1913
EPANAGYRUS Girault, 1915
NEANAGYRUS Girault, 1915
ANAGYROPSIS Girault, 1917
METAPRIONOMITUS Mercet, 1921
SHAKESPEARIA Girault, 1928
PSYLLENCYRTUS Tachikawa, 1955
CALLUNIPHILUS Erdös, 1961
ANISODROMUS Riek, 1962
OOENCYRTOIDES Hoffer, 1963
PROPSYLLAEPHAGUS Blanchard, 1964
MERCETIA Bakkendorf, 1965
KASZABICYRTUS Szelényi, 1971

#### Psyllaephagus lusitanicus

(Mercet, 1921)

Copidosoma lusitanicum Mercet, 1921
cocci Alam, 1957
vendicus (Erdös, 1961, *Calluniphilus*)
albopilosus (Hoffer, 1963, *Ooencyrtus*)

##### Distribution

England, Wales

#### Psyllaephagus pilosus

Noyes, 1988

##### Distribution

England, Wales, Ireland, Isle of Man

##### Notes

Introduced for biological control

#### Sectiliclava

Hoffer, 1957

PARAPSYLLAEPHAGUS Robinson, 1961

#### Sectiliclava cleone

(Walker, 1844)

Encyrtus cleone Walker, 1844
ungularis (Thomson, 1876, *Litomastix*)
adulticollis (Robinson, 1961, *Parapsyllaephagus*)
paliuri Hoffer, 1957

##### Distribution

England

#### Stemmatosteres

Timberlake, 1918

#### Stemmatosteres bohemicus

Hoffer, 1954

#### Subprionomitus

Mercet, 1921

KAKAOBURRA Girault, 1922

#### Subprionomitus festucae

(Mayr, 1876)

Encyrtus festucae Mayr, 1876
cantabricus Mercet, 1921

##### Distribution

England

#### Syrphophagus

Ashmead, 1900

APHIDENCYRTUS Ashmead, 1900
ECHTHROBACCHA Perkins, 1906
NESYRPHOPHAGUS Girault, 1915
NESYRPOPHAGUS Girault, 1915
HEXANUSIA Girault, 1922
SYRPHIDENCYRTUS Blanchard, 1940

#### Syrphophagus aeruginosus

(Dalman, 1820)

Encyrtus aeruginosus Dalman, 1820
dercilus (Walker, 1837, *Encyrtus*)
thinaeus (Walker, 1837, *Encyrtus*)
aenescens (Zetterstedt, 1838, *Encyrtus*)
meges (Walker, 1846, *Encyrtus*)
syrphi (Ratzeburg, 1852, *Encyrtus*)
congruus (Walker, 1872, *Encyrtus*)

##### Distribution

Ireland

#### Syrphophagus aphidivorus

(Mayr, 1876)

Encyrtus aphidivorus Mayr, 1876
schizoneurae (Ashmead, 1885, *Eupelmus*)
aphidiphagus (Ashmead, 1887, *Encyrtus*)
megourae (Ashmead, 1887, *Encyrtus*)
websteri (Howard, 1890, Encyrtus)
submetallicus (Mercet, 1921, *Microterys*)
silvestrinus Ghesquière, 1956
merceti Erdös, 1957
psyllae (Kaul & Agarwal, 1986, *Aphidencyrtus*)
kerrichi (Fatima & Shafee, 1994, *Adelencyrtus*)

##### Distribution

England, Scotland

#### Syrphophagus ariantes

(Walker, 1837)

Encyrtus ariantes Walker, 1837
elbasus (Walker, 1837, *Encyrtus*)
scythis (Walker, 1838, *Encyrtus*)

##### Distribution

England, Ireland

#### Syrphophagus fuscipes

(Dalman, 1820)

Encyrtus fuscipes Dalman, 1820

##### Distribution

England

#### Syrphophagus herbidus

(Dalman,1820)

Encyrtus herbidus Dalman,1820
batillus (Walker, 1837, *Encyrtus*)
tegularis Hoffer, 1970

##### Distribution

England, Ireland

#### Syrphophagus hyalipennis

(Mayr, 1876)

Encyrtus hyalipennis Mayr, 1876

##### Distribution

Ireland

##### Notes

Added by O'Connor et al. (2000). Listed by Bouček and Graham (1978) as a junior synonym of *taeniatus*.

#### Syrphophagus mamitus

(Walker, 1837)

Encyrtus mamitus Walker, 1837
erylus (Walker, 1838, *Encyrtus*)
cantabricus (Mercet, 1921, *Microterys*)

##### Distribution

England, Wales

#### Syrphophagus pertiades

(Walker, 1837)

Encyrtus pertiades Walker, 1837
magnus Hoffer, 1965

##### Distribution

England

#### Syrphophagus philotis

(Walker, 1848)

Encyrtus philotis Walker, 1848

##### Distribution

England

#### Syrphophagus qadrii

(Alam, 1961)

Aphidencyrtus qadrii Alam, 1961

##### Distribution

England

#### Syrphophagus quercicola

(Hoffer, 1970)

Aphidencyrtus quercicola Hoffer, 1970

##### Distribution

England

#### Syrphophagus sosius

(Walker, 1837)

Encyrtus sosius Walker, 1837

##### Distribution

England

#### Syrphophagus taeniatus

(Förster, 1861)

Encyrtus taeniatus Förster, 1861

##### Distribution

England

#### Thomsonisca

Ghesquière, 1946

THOMSONIELLA Mercet, 1921 preocc.
HETERENCYRTUS Hoffer, 1953
ATHESMUS Erdös & Novicky, 1955
EUUSSURIA Chumakova, 1957
KOSZTARABIA Erdös, 1957
PAKENCYRTUS Ahmad, 1970

#### Thomsonisca amathus

(Walker, 1838)

Encyrtus amathus Walker, 1838
typica (Mercet, 1921, *Thomsoniella*)
sumavicus (Hoffer, 1953, *Heterencyrtus*)
luctuosus (Erdös & Novicky, 1955, *Athesmus*)
chionaspidis (Erdös, 1957, *Kosztarabia*)
britannica Alam, 1957
chionaspidis Hedqvist, 1958

##### Distribution

England, Ireland

#### Trechnites

Thomson, 1876

PSYLLEDONTUS Crawford, 1910
METALLONELLA Girault, 1915

#### Trechnites alni

Erdös, 1957

crassus Erdös, 1957

##### Distribution

England, Wales, Ireland

#### Trechnites fuscitarsis

(Thomson, 1876)

Metallon fuscitarsis Thomson, 1876

##### Distribution

England, Scotland, Wales

#### Trechnites psyllae

(Ruschka, 1923)

Metallon psyllae Ruschka, 1923

##### Distribution

England,

#### Trichomasthus

Thomson, 1876

COCCIDOXENUS Crawford, 1913

#### Trichomasthus albimanus

Thomson, 1876

##### Distribution

England, Scotland

##### Notes

BMNH, det. Bonde-Jensen, added here

#### Trichomasthus cyaneus

(Dalman, 1820)

Encyrtus cyaneus Dalman, 1820
cyanellus (Dalman, 1820, *Encyrtus*)

##### Distribution

England, Wales, Ireland

#### Trichomasthus cyanifrons

(Dalman, 1820)

Encyrtus cyanifrons Dalman, 1820

##### Distribution

England

#### Trichomasthus danzigae

Trjapitzin, 1978

##### Distribution

Scotland

##### Notes

Added by Jensen and Sharkov (1989)

#### Trichomasthus frontalis

Alam, 1957

solitocornis (Kaul & Agarwal, 1986, *Ginsiana*)

##### Distribution

England

#### Trichomasthus gabinius

(Walker, 1837)

Encyrtus gabinius Walker, 1837
gabinus misspelling

##### Distribution

England, Ireland

#### Trichomasthus genutius

(Walker, 1846)

Encyrtus genutius Walker, 1846

##### Distribution

England,

#### Trichomasthus marsus

(Walker, 1837)

Encyrtus marsus Walker, 1837
Trichomasthus marsus ? *mattinus* (Walker, 1837, *Encyrtus*)

##### Distribution

England, Scotland, Wales

#### Tyndarichus

Howard, 1910

#### Tyndarichus melanacis

(Dalman, 1820)

Encyrtus melanacis Dalman, 1820
jancirus (Walker, 1837, *Encyrtus*)
ignotus Mercet, 1947

##### Distribution

England, Scotland

#### Tyndarichus scaurus

(Walker, 1837)

Encyrtus scaurus Walker, 1837
genetyllis (Walker, 1848, *Encyrtus*)

##### Distribution

England

#### Zaomma

Ashmead, 1900

APTERENCYRTUS Ashmead, 1905
METALLONOIDEA Girault, 1915
CHILONEURINUS Mercet, 1921
RICHARDSIUS Alam, 1957
METAPTERENCYRTUS Tachikawa, 1963

#### Zaomma lambinus

(Walker, 1838)

Encyrtus lambinus Walker, 1838
microphagus (Mayr, 1876, *Chiloneurus*)
euryclea (Walker, 1844, *Encyrtus*)
diaspidinarum (Howard, 1894, *Chiloneurus*)
pulchricornis (Ashmead, 1905, *Apterencyrtus*)
aspidioti (Girault, 1915, *Aphidencyrtus*)
brittanica (Girault, 1915, *Metallonoidea*)
mayri (Ruschka, 1915, *Habrolepis*)
thomsoniscae (Alam, 1957, *Apterencyrtus*)
zonatus (Alam, 1957, *Apterencyrtus*)
adeli (Traboulsi, 1968, *Apterencyrtus*)

##### Distribution

England, Ireland

#### Tetracneminae

Howard, 1892

#### Aglyptus

Förster, 1856

#### Aglyptus rufus

(Dalman, 1820)

Eupelmus rufus Dalman, 1820
rufescens (Nees, 1834, *Eupelmus*)
lindus (Walker, 1837, *Encyrtus*)
rufus (Walker, 1837, *Encyrtus*) preocc.

##### Distribution

England

#### Anagyrus

Howard, 1896

HETERARTHRELLUS Howard 1898
EPIDINOCARSIS Girault, 1913
PARANUSIA Brèthes, 1913
PHILOPONECTROMA Brèthes, 1913
DOLIPHOCERAS Mercet, 1921
GYRANUSA Mercet, 1921
GYRANUSIA Brèthes, 1921
PROTANAGYRUS Blanchard, 1940
APOANAGYRUS Compere, 1947
ANATHRIX Burks, 1952
RHOPOMORPHUS Ghesquière, 1958
NESOANAGYRUS Beardsley, 1969
XIPHOMASTIX De Santis, 1972
CREMESINA Noyes & Hayat, 1984
TONGYUS Noyes & Hayat, 1984

#### Anagyrus aligarhensis

Agarwal & Alam, 1959

diversicornis Mercet, 1921 preocc.
Anagyrus aligarhensis ? *opacum* (Mercet, 1921, *Philoponectroma*)
punctulatus Agarwal & Alam, 1959
punctulatus Agarwal, 1965 preocc.
micans Noyes, 2000

##### Distribution

England

##### Notes

Added by Springate and Noyes (1990)

#### Anagyrus belibus

(Walker, 1837)

Encyrtus belibus Walker, 1837
scyles (Walker, 1837, *Encyrtus*)
arene (Walker, 1838, *Encyrtus*)
barca (Walker, 1838, *Encyrtus*)
dores (Walker, 1838, *Encyrtus*)
elpis (Walker, 1838, *Encyrtus*)
mamertus (Walker, 1846, *Encyrtus*)
integralis (Mercet, 1919, *Pholidoceras*)
pseudococci (Alam, 1957, *Doliphoceras*) preocc.
varleyellus (Ghesquière, 1958, *Rhopomorphus*)

##### Distribution

England, Wales, Ireland

#### Anagyrus bohemanni

(Westwood, 1837)

Encyrtus bohemanni Westwood, 1837
quercicola Mercet, 1921
singularis Hoffer, 1953
mayri (Ruschka, 1923, *Blastothrix*)

##### Distribution

England, Scotland

#### Anagyrus bouceki

Hoffer, 1953

##### Distribution

England, Wales

##### Notes

Added by Springate and Noyes (1990)​

#### Anagyrus novickyi

Hoffer, 1953

##### Distribution

England, Wales,

##### Notes

Added by Springate and Noyes (1990)​

#### Anagyrus pseudococci

(Girault, 1915)

Epidinocarsis pseudococci Girault, 1915

##### Notes

Introduced into greenhouses for biological control

#### Anagyrus schoenherri

(Westwood, 1837)

Encyrtus schoenherri Westwood, 1837
alboclavatus Ishii, 1928
flavus Ishii, 1928

##### Distribution

England, Isle of Man

#### Anagyrus securicornis

Domenichini, 1953

bohemicus Hoffer, 1953
sabulicola (Hoffer, 1953, *Gyranusa*)

##### Distribution

England, Wales

##### Notes

Added by Springate and Noyes (1990)​

#### Anomalicornia

Mercet, 1921

#### Anomalicornia tenuicornis

Mercet, 1921

ruschkai Mercet, 1922

##### Distribution

England, Wales

#### Anusia

Förster, 1856

#### Anusia nasicornis

Förster, 1860

austriaca Förster, 1860
laevis (Mercet, 1921, *Doliphoceras*)

##### Distribution

England, Ireland

#### Charitopus

Förster, 1856

LEPTORHOPALA Motschulsky, 1863
EUPELMOMORPHA Girault, 1915
DIVERSICORNIA Mercet, 1916

#### Charitopus fulviventris

Förster, 1860

pinicola (Mercet, 1916, *Diversicornia*)

#### Coccidoxenoides

Girault, 1915

PAURIDIA Timberlake, 1919

#### Coccidoxenoides perminutus

Girault, 1915

peregrina (Timberlake, 1919, *Pauridia*)
babindae (Girault, 1922, *Fulgoridicida*)
ivorensis (Risbec, 1955, *Protyndarichus*)

##### Notes

Introduced into greenhouses for biological control

#### Dinocarsis

Förster, 1856

EUSCAPUS Dahlbom, 1857

#### Dinocarsis hemiptera

(Dalman, 1820)

Encyrtus hemipterus Dalman, 1820
submontana Hoffer, 1952

##### Distribution

England, Wales, Ireland

#### Dusmetia

Mercet, 1921

BACALUSA Noyes & Hayat, 1984

##### Notes

Species of *Dusmetia* removed from the British and Irish list:

[*ceballosi* Mercet, 1921]

Recorded as British by Bouček (1977) without comment; no British or Irish specimens have come to light and this record was almost certainly an error.

#### Dusmetia pulex

(Ruschka, 1923)

Blastothrix pulex Ruschka, 1923

##### Distribution

England

#### Ericydnus

Haliday, 1832

GRANDORIELLA Domenichini, 1952

#### Ericydnus apterogenes

Mayr, 1876

latiusculus Thomson, 1876

##### Notes

Added by Graham (1991b)

#### Ericydnus baleus

(Walker, 1838)

Encyrtus baleus Walker, 1838

##### Distribution

England, Scotland, Wales

#### Ericydnus sipylus

(Walker, 1837)

Encyrtus sipylus Walker, 1837
aemnestus (Walker, 1850, *Encyrtus*)
bicolor Nikol'skaya, 1952
basalis (Förster, 1861, *Encyrtus*)
atriceps (Walker, 1872, *Metallon*)

##### Distribution

England, Ireland

#### Ericydnus strigosus

(Nees, 1834)

Encyrtus strigosus Nees, 1834

##### Distribution

England, Ireland

#### Ericydnus ventralis

(Dalman, 1820)

Encyrtus ventralis Dalman, 1820
paludatus Haliday, 1837
dichrous Mercet, 1921

##### Distribution

England, Ireland

#### Gyranusoidea

Compere, 1947

LEPTANUSIA De Santis, 1964
NEURANAGYRUS Bouček, 1977
THERENCYRTUS Trjapitzin, 1977

#### Gyranusoidea aphycoides

(Mercet, 1921)

Heterarthrellus aphycoides Mercet, 1921

##### Distribution

England

#### Leptomastidea

Mercet, 1916

TANAOMASTIX Timberlake, 1918

#### Leptomastidea abnormis

(Girault, 1915)

Paraleptomastix abnormis Girault, 1915
aurantiaca Mercet, 1916

##### Distribution

England

##### Notes

Introduced into greenhouses for biological control

#### Leptomastidea bifasciata

(Mayr, 1876)

Blastothrix bifasciata Mayr, 1876

##### Distribution

England

#### Leptomastix

Förster, 1856

STERRHOCOMA Förster, 1856
STENOTERYS Thomson, 1876

#### Leptomastix dactylopii

Howard, 1885

superba Silvestri, 1915
longipennis Mercet, 1927
bifasciata Compere, 1938
tambourissae Risbec, 1952

##### Distribution

England

##### Notes

Introduced into greenhouses for biological control

#### Leptomastix epona

(Walker, 1844)

Encyrtus epona Walker, 1844
orbitalis (Thomson, 1876, *Stenoterys*)

##### Distribution

England

#### Mira

Schellenberg, 1803

DICELLOCERAS Menzel, 1855
EURYSCAPUS Förster, 1856
LONCHOCERUS Dahlbom, 1857
EUZKADIA Mercet, 1921

#### Mira mucora

Schellenberg, 1803

macrocera Schellenberg, 1803
platycerus (Dalman, 1820, *Encyrtus*)
vibrans (Menzel, 1855, *Dicelloceras*)

##### Distribution

England

#### Rhopus

Förster, 1856

XANTHOENCYRTUS Ashmead, 1902
MIRASTYMACHUS Girault, 1915
SCELIOENCYRTUS Girault, 1915
PHOLIDOCERAS Mercet, 1918
PHOLIDOCERODES Ferrière, 1955
PLATYRHOPUS Erdös, 1955
HAMUSENCYRTUS Subba Rao & Hayat, 1979
NEOXANTHOENCYRTUS Avasthi & Shafee, 1980

#### Rhopus acaetes

(Walker, 1844)

Aphelinus acaetes Walker, 1844
jarli (Kryger, 1943, *Pholidoceras*)

##### Distribution

England, Wales

#### Rhopus caris

(Walker, 1838)

Encyrtus caris Walker, 1838

##### Distribution

England

#### Rhopus piso

(Walker, 1838)

Encyrtus piso Walker, 1838
donostiarrense (Mercet, 1921, *Doliphoceras*)

##### Distribution

England, Ireland

#### Rhopus semiapterus

(Mercet, 1921)

Pholidoceras semiaptera Mercet, 1921

##### Distribution

England

##### Notes

Recorded as British by Herting (1972) and subsequently other authors; omitted by Bouček and Graham (1978)

#### Rhopus sulphureus

(Westwood, 1837)

Encyrtus sulphureus Westwood, 1837
europaeus (Girault, 1915, *Mirastymachus*)

##### Distribution

England

#### Tetracnemoidea

Howard, 1898

TETRACNEMOPSIS Ashmead, 1900
ARHOPOIDEUS Girault, 1915
ECTROMELLA Girault, 1915
ANARHOPUS Timberlake, 1929
HUNGARIELLA Erdös, 1946
ANTIPODENCYRTUS Kerrich, 1964
ZEALANDENCYRTUS Tachikawa & Valentine, 1971

#### Tetracnemoidea brevicornis

(Girault, 1915)

Arhopoideus brevicornis Girault, 1915
pretiosa (Timberlake, 1929, *Tetracnemus*)

##### Distribution

England

##### Notes

Added by Japoshvili and Noyes (2006). Probably introduced from Australia via The Netherlands and may not be established

#### Tetracnemus

Westwood, 1837

CALOCERINUS Howard, 1892
TETRACLADIA Howard, 1892
HENICOPYGUS Ashmead, 1900
TETRALOPHIDEA Ashmead, 1900
TETRALOPHIELLUS Ashmead, 1900
PARACALOCERINUS Girault, 1915
NEBAOCHARIS Girault, 1916
MASIA Mercet, 1919
ANUSIELLA Mercet, 1923
PLACOCERAS Erdös, 1946
COMPERENCYRTUS De Santis, 1964

#### Tetracnemus diversicornis

Westwood, 1837

pulchripennis (Mercet, 1923 *Masia*)

##### Distribution

England

### Family Eucharitidae Walker, 1846

#### Eucharitinae

Walker, 1846

#### Eucharis

Latreille, 1804

PSILOGASTER Blanchard, 1840
PSILOGASTRELLUS Ghesquière, 1946
EUCHARISCA Bouček, 1956

#### Eucharis adscendens

(Fabricius, 1787)

Cynips adscendens Fabricius, 1787
kollari Förster, 1859

##### Distribution

England, Wales

##### Notes

Not found in Britain since 1907 (Ferrière and Kerrich 1958) and probably extinct in this country.

### Family Eulophidae Westwood, 1829

#### Eulophidae

Westwood, 1829

##### Notes

Graham (1963) gives some distribution data for a number of species.

#### Entedoninae

Förster, 1856

#### Achrysocharoides

Girault, 1913

ENAYSMA Delucchi, 1954

#### Achrysocharoides acerianus

(Askew, 1974)

Enaysma aceriana Askew, 1974

#### Achrysocharoides atys

(Walker, 1839)

Entedon atys Walker, 1839
aenea (Delucchi, 1956, *Enaysma*)

##### Distribution

England, Scotland

#### Achrysocharoides buekkensis

(Erdös, 1958)

Chrysocharis buekkensis Erdös, 1958

##### Distribution

England

##### Notes

Askew and Ruse (1974) note British specimens that may be *A. buekkensis* - record needs confirmation.

#### Achrysocharoides butus

(Walker, 1839)

Entedon butus Walker, 1839
septentrionalis (Delucchi, 1957, *Enaysma*)

##### Distribution

England, Wales

#### Achrysocharoides carpini

Bryan, 1980

##### Notes

Added by Bryan (1980)

#### Achrysocharoides cilla

(Walker, 1839)

Entedon cilla Walker, 1839
leucobates (Ratzeburg, 1848, *Elachestus*)
chrysostomus (Thomson, 1878, *Derostenus*)

##### Distribution

England

#### Achrysocharoides insignitellae

(Erdös, 1966)

Chrysocharis insignitellae Erdös, 1966

##### Notes

Added in

#### Achrysocharoides latreillii

(Curtis, 1826)

Eulophus latreillii Curtis, 1826

##### Distribution

England, Scotland

#### Achrysocharoides niveipes

(Thomson, 1878)

Derostenus niveipes Thomson, 1878

##### Distribution

England

#### Achrysocharoides platanoidae

Hansson & Shevtsova, 2010

##### Distribution

England

##### Notes

Added by Hansson and Shevtsova (2010)

#### Achrysocharoides splendens

(Delucchi, 1954)

Enaysma splendens Delucchi, 1954

##### Distribution

England

#### Achrysocharoides suprafolius

(Askew, 1974)

Enaysma suprafolia Askew, 1974

#### Achrysocharoides zwoelferi

(Delucchi, 1954)

Enaysma zwoelferi Delucchi, 1954

##### Distribution

England, Scotland

#### Asecodes

Förster, 1856

GANAHLIA Dalla Torre, 1897
TELEOPTERUS Silvestri, 1914
METASECODES Erdös, 1955
DESMATOCHARIS Graham, 1959

#### Asecodes congruens

(Nees, 1834)

Eulophus congruens Nees, 1834
coronis (Walker, 1838, *Cirrospilus*)
eudora (Walker, 1838, *Cirrospilus*)
lycomedes (Walker, 1838, *Cirrospilus*)
orelia (Walker, 1838, *Cirrospilus*)
procles (Walker, 1838, *Cirrospilus*)
thione (Walker, 1839, *Pteroptrix*)
fuscipes Förster, 1861
nitens Förster, 1861
orbatum (Szelényi, 1978, *Eugerium*)

##### Distribution

England, Scotland, Ireland

#### Asecodes delucchii

(Bouček, 1971)

Teleopterus delucchii Bouček, 1971

##### Distribution

England

#### Asecodes erxias

(Walker, 1848)

Entedon erxias Walker, 1848
scutellatus (Ferrière, 1952, *Omphale*)
bicolor (Erdös, 1955, *Metasecodes*)

##### Distribution

England, Wales, Ireland

#### Asecodes hyperion

Graham, 1963

##### Distribution

England, Wales

#### Asecodes lagus

(Walker, 1838)

Cirrospilus lagus Walker, 1838
agamedes (Walker, 1839, *Entedon*)

##### Distribution

England

#### Asecodes lucens

(Nees, 1834)

Eulophus lucens Nees, 1834
chthonia (Walker, 1839, *Entedon*)
mento (Walker, 1839, *Entedon*)
metagenes (Walker, 1848, *Entedon*)
parviclava (Thomson. 1878, *Derostenus*)

##### Distribution

England, Scotland, Ireland

#### Ceranisus

Walker, 1842

THRIPOCTENUS Crawford, 1911
EPOMPHALE Girault, 1915
URFACUS Doganlar, 2003
GAZIANTEPUS Doganlar & Doganlar, 2013
GUELSENIA Doganlar & Doganlar, 2013
SERGUEICUS Doganlar & Doganlar, 2013

#### Ceranisus lepidotus

Graham, 1963

##### Distribution

England

#### Ceranisus menes

(Walker, 1839)

Pteroptrix menes Walker, 1839
brui (Vuillet, 1914, *Thripoctenus*)
vinctus (Gahan, 1932, *Thripoctenus*)
rosilloi De Santis, 1961

#### Ceranisus pacuvius

(Walker, 1838)

Cirrospilus pacuvius Walker, 1838
acestor (Walker, 1839, *Entedon*)
aculeo (Walker, 1848, *Diglyphus*)
clavicornis (Thomson, 1878, *Derostenus*)
kutteri (Ferrière, 1936, *Thripoctenus*)

##### Distribution

England

#### Ceranisus planitianus

Erdös, 1966

#### Chrysocharis

Förster, 1856

EUOPHTHALMOMYIA Ashmead, 1904
NESOMYIA Ashmead, 1904
ZAOMMOMYIA Ashmead, 1904
OMPHALCHRYSOCHARIS Girault, 1917
RHICNOPELTOIDEA Girault, 1917
EUPARACRIAS Brèthes, 1923
PHYTOMYZOPHAGA Brèthes, 1923
KRATOCHVILIANA Malac, 1943
EPILAMPSIS Delucchi, 1954

#### Chrysocharis acoris

(Walker, 1839)

Entedon acoris Walker, 1839
tamus (Walker, 1839, *Entedon*)
incerta Yoshimoto, 1973

##### Distribution

England, Scotland

#### Chrysocharis acutigaster

Hansson, 1985

##### Notes

Added by Hansson (1985)

#### Chrysocharis albicoxis

Erdös, 1958

##### Distribution

England

#### Chrysocharis amanus

(Walker, 1839)

Entedon amanus Walker, 1839
varus (Walker, 1839, *Entedon*)
nepticularum Erdös, 1954
sanguisorbae Erdös, 1961
aceris Erdös, 1966

#### Chrysocharis amasis

(Walker, 1839)

Entedon amasis Walker, 1839
aurifrons (Thomson, 1878, *Derostenus*)
icetas (Walker, 1848, *Entedon*)
larina (Walker, 1839, *Entedon*)

##### Distribution

England

#### Chrysocharis amyite

(Walker, 1839)

Entedon amyite Walker, 1839
filicornis (Thomson, 1878, *Derostenus*)
brevis Delucchi, 1954
seiuncta Delucchi, 1954
bellincus Yoshimoto, 1973

##### Distribution

England

#### Chrysocharis antoni

Hansson, 1985

##### Notes

BMNH, det. Hansson, added here

#### Chrysocharis argyropezae

Graham, 1963

##### Distribution

England, Ireland

#### Chrysocharis assis

(Walker, 1839)

Entedon assis Walker, 1839
orientalis (Girault, 1917, *Omphalchrysocharis*)

##### Distribution

England

#### Chrysocharis budensis

Erdös, 1954

##### Notes

Added by Askew (1984a)

#### Chrysocharis chlorus

Graham, 1963

graminearum (Szelényi & Szöcs, 1976, *Neochrysocharis*)

##### Distribution

England

#### Chrysocharis collaris

Graham, 1963

##### Distribution

England, Scotland

#### Chrysocharis crassiscapus

(Thomson, 1878)

Derostenus crassiscapus Thomson, 1878
mallochi Gahan, 1917
sulcatus (Erdös, 1954, *Derostenus*)

##### Distribution

England

#### Chrysocharis elongata

(Thomson, 1878)

Derostenus elongatus Thomson, 1878
pontaniae Askew & Kopelke, 1989

##### Distribution

England

#### Chrysocharis entedonoides

(Walker, 1872)

Eulophus entedonoides Walker, 1872
albicans Delucchi, 1954

##### Distribution

England

##### Notes

Added by Hansson (1985)

#### Chrysocharis eurynota

Graham, 1963

##### Distribution

England

#### Chrysocharis gemma

(Walker, 1839)

Entedon gemma Walker, 1839
proclea (Walker, 1839, *Entedon*)
centralis (Walker, 1872, *Eulophus*)

##### Distribution

England

#### Chrysocharis idyia

(Walker, 1839)

Entedon idyia Walker, 1839
pontinus (Walker, 1839, *Entedon*)
iriarte (Walker, 1848, *Gastrancistrus*)
heterotomus (Thomson, 1878, *Derostenus*)

##### Distribution

England

#### Chrysocharis illustris

Graham, 1963

##### Distribution

England, Scotland

#### Chrysocharis laomedon

(Walker, 1839)

Entedon laomedon Walker, 1839
parsodes (Walker, 1839, *Entedon*)
sartamus (Walker, 1839, *Entedon*)
albiceps (Delucchi, 1954, *Epilampsis*)
coxalis (Delucchi, 1956, *Epilampsis*)
hirsutiventris Yoshimoto, 1973
yoshimotoi Doganlar, 1980

##### Distribution

England, Scotland, Wales

#### Chrysocharis laricinellae

(Ratzeburg, 1848)

Entedon laricinellae Ratzeburg, 1848
Chrysocharis laricinellae ?*ocyalus* (Walker, 1839, *Entedon*)

##### Distribution

England, Wales

#### Chrysocharis liriomyzae

Delucchi, 1954

punctifacies Delucchi, 1954
foveata Szelényi, 1981

##### Distribution

England, Scotland

#### Chrysocharis mediana

Förster, 1861

subpolita Erdös, 1958
levipectus Yoshimoto, 1973

##### Distribution

England

#### Chrysocharis moravica

(Malac, 1943)

Kratochviliana moravica Malac, 1943

#### Chrysocharis nautius

(Walker, 1846)

Entedon nautius Walker, 1846
deciduae (Delucchi, 1954, *Epilampsis*)

##### Distribution

England

#### Chrysocharis nephereus

(Walker, 1839)

Entedon nephereus Walker, 1839
erigone (Walker, 1839, *Entedon*)
inarus (Walker, 1839, *Entedon*)
matho (Walker, 1839, *Entedon*)
metella (Walker, 1839, *Entedon*)
nautes (Walker, 1839, *Entedon*)
orchestis (Ratzeburg, 1844, *Eulophus*)
laetus (Ratzeburg, 1848, *Entedon*)
sauros (Walker, 1848, *Entedon*)
auronitens (Ratzeburg, 1852, *Entedon*)
obscurinervis Bukovskii, 1938
orchestidis Bukovskii, 1938
smirnovi Bukovskii, 1938
gunholdi (Delucchi, 1954, *Epilampsis*)
laevigata (Delucchi, 1954, *Epilampsis*)
tadici (Delucchi, 1954, *Epilampsis*)
cuspidigaster Yoshimoto, 1973
truncatipennis Yoshimoto, 1973
elmaellae Doganlar, 1980

##### Distribution

England, Scotland

#### Chrysocharis nigricrus

(Thomson, 1878)

Derostenus nigricrus Thomson, 1878

#### Chrysocharis nitetis

(Walker, 1839)

Entedon nitetis Walker, 1839
boops (Thomson, 1878, *Derostenus*)
novellus (Walker, 1839, *Entedon*)
elegantissima (Girault, 1917, *Chrysocharomyia*)
milleri Yoshimoto, 1973

##### Distribution

England

#### Chrysocharis nitidifrons

Graham, 1963

##### Distribution

England, Ireland

#### Chrysocharis orbicularis

(Nees, 1834)

Elachestus orbicularis Nees, 1834
abrota (Walker, 1839, *Entedon*)
altadas (Walker, 1839, *Entedon*)
charaxus (Walker, 1839, *Entedon*)
eutropius (Walker, 1839, *Entedon*)
lycoris (Walker, 1839, *Entedon*)
naenia (Walker, 1839, *Entedon*)
nurscia (Walker, 1848, *Entedon*)
facialis Förster, 1861
punctifrons (Thomson, 1878, *Derostenus*)

##### Distribution

England, Scotland

#### Chrysocharis pallipes

(Nees, 1834)

Elachestus pallipes Nees, 1834
alphenus (Walker, 1839, *Entedon*)
chilo (Walker, 1839, *Entedon*)
lycambes (Walker, 1839, *Entedon*)
parmys (Walker, 1839, *Entedon*)
petiolata Förster, 1861
petiolatus (Thomson, 1878, *Derostenus*)
pallidipes (Dalla Torre, 1898, *Elachistus*)

##### Distribution

England, Scotland, Wales

#### Chrysocharis pentheus

(Walker, 1839)

Entedon pentheus Walker, 1839
ergetelis (Walker, 1848, *Entedon*)
pallipes (Gahan, 1917, *Derostenus*) preocc.
mirabilis (Sundby, 1957, *Epilampsis*)
aquilegiae Erdös, 1961

##### Distribution

England, Wales

#### Chrysocharis phryne

(Walker, 1839)

Entedon phryne Walker, 1839
scutellaris (Thomson, 1878, *Derostenus*)

##### Distribution

England

#### Chrysocharis pilicoxa

(Thomson, 1878)

Derostenus pilicoxa Thomson, 1878

##### Distribution

England, Scotland

#### Chrysocharis pilosa

Delucchi, 1954

##### Notes

Added by Godfray and Askew (2013)

#### Chrysocharis polyzo

(Walker, 1839)

Entedon polyzo Walker, 1839
acerbas (Walker, 1839, *Entedon*)
enephes (Walker, 1839, *Entedon*)
leucippus (Walker, 1839, *Entedon*)
palustris (Goureau, 1851, *Omphale*)
thomsoni (Crawford, 1913, *Entedon*)
depressa Delucchi, 1954
plana Delucchi, 1954

##### Distribution

England, Scotland

#### Chrysocharis prodice

(Walker, 1839)

Entedon prodice Walker, 1839
daunus (Walker, 1839, *Entedon*)
thoe (Walker, 1839, *Entedon*)
coedicius (Walker, 1846, *Entedon*)
temena (Walker, 1848, *Entedon*)
latipennis (Thomson, 1878, *Derostenus*)
salutaris (Crosby, 1911, *Derostenus*)
stipitis Yoshimoto, 1973
duriceps Szelényi, 1979

##### Distribution

England, Scotland, Wales

#### Chrysocharis pubens

Delucchi, 1954

latifrons Gijswijt, 1965

##### Distribution

England

#### Chrysocharis pubicornis

(Zetterstedt, 1838)

Entedon pubicornis Zetterstedt, 1838
punctellus (Zetterstedt, 1838, *Entedon*)
aesopus (Walker, 1839, *Entedon*)
amyrtaeus (Walker, 1839, *Entedon*)
cydon (Walker, 1839, *Entedon*)
eropus (Walker, 1839, *Entedon*)
syma (Walker, 1839, *Entedon*)
hersilia (Walker, 1840, *Entedon*)
adreus (Walker, 1848, *Entedon*)
femoralis Förster, 1861
aeneiscapus (Thomson, 1878, *Derostenus*)
avellanae Erdös, 1961
bipicturata Szelényi, 1977
asclepiadeae Szelényi, 1979
tranquilla Szelényi, 1981

##### Distribution

England, Wales

#### Chrysocharis purpurea

Bukowski, 1938

phyllotomae (Delucchi, 1954, *Epilampsis*)
kumatai (Kamijo, 1960, *Epilampsis*)

##### Distribution

England

#### Chrysocharis submutica

Graham, 1963

##### Distribution

England

#### Chrysocharis truncatula

Graham, 1963

##### Distribution

England

#### Chrysocharis viridis

(Nees, 1834)

Elachestus viridis Nees, 1834
subauratus (Nees, 1834, *Elachestus*)
afranius (Walker, 1839, *Entedon*)
aso (Walker, 1839, *Entedon*)
bibulus (Walker, 1839, *Entedon*)
calitor (Walker, 1839, *Entedon*)
melaenis (Walker, 1839, *Entedon*)
tanis (Walker, 1839, *Entedon*)
thersamon (Walker, 1839, *Entedon*)
viridicoxis Förster, 1861
punctiscapus (Thomson, 1878, *Derostenus*)
pallidipes (Ashmead, 1904, *Euophthalmomyia*)
appendigaster (Masi, 1952, *Derostenus*)
albula Delucchi, 1954

##### Distribution

England, Scotland

#### Chrysonotomyia

Ashmead, 1904

NEOCHRYSOCHARELLA Dodd, 1915
RUBENSTEINA Girault, 1934
MOSERINA Delucchi, 1962
LADNA Bouček, 1988
CALLIFRONS Schauff, Yoshimoto & Hansson, 1994

#### Chrysonotomyia germanicus

(Erdös, 1956)

Halochariessa germanica Erdös, 1956

##### Distribution

England

#### Closterocerus

Westwood, 1833

ACHRYSOCHARELLA Girault, 1913
ACHRYSOCHARIS Girault, 1913
CHRYSOCHARELLA Girault, 1913
PSEUDOCHRYSOCHARIS Girault, 1913
WOLFFIELLA Krausse, 1917
HALOCHARIS Erdös, 1951
CHRYSOCHARIDIA Erdös, 1956
HALOCHARIESSA Erdös, 1956
CECIDIOPHAGA Erdös, 1966
MANGOCHARIS Bouček, 1986
HISPINOCHARIS Bouček, 1988

#### Closterocerus lanassa

(Walker, 1839)

Entedon lanassa Walker, 1839
debilis (Förster, 1841, *Eulophus*)

##### Distribution

England

#### Closterocerus lyonetiae

(Ferrière, 1952)

Achrysocharis lyonetiae Ferrière, 1952

##### Notes

BMNH, det. Hansson, added here

#### Closterocerus smaragdulus

(Graham, 1963)

Achrysocharis smaragdula Graham, 1963

##### Distribution

England

#### Closterocerus trifasciatus

Westwood, 1833

winnemanae Crawford, 1912
sesquifasciatus (Ratzeburg, 1844, *Eulophus*)
tricincta (Ashmead, 1888, *Pleurotropis*)

##### Distribution

England

#### Closterocerus turcicus

(Nees, 1834)

Eulophus turcicus Nees, 1834

#### Derostenus

Westwood, 1833

#### Derostenus gemmeus

Westwood, 1833

albipes (Nees, 1834, *Elachestus*)
albiscapus (Nees, 1834, *Elachestus*)
amyclas (Walker, 1839, *Entedon*)
caesius (Walker, 1839, *Entedon*)
rutilans (Walker, 1840, *Entedon*)
cupreus (Förster, 1841, *Elachestus*)
conformis Thomson, 1878
laevifrons Thomson, 1878
levifrons Dalla Torre, 1898

##### Distribution

England, Scotland

#### Derostenus punctiscuta

Thomson, 1878

##### Distribution

England

#### Entedon

Dalman, 1820

TRANOCERA Curtis, 1829
PLEUROPACHUS Westwood, 1837
PLEUROPACHYS Förster, 1856
ERIGLYPTUS Crawford, 1907
ENTEDONELLA Girault, 1913
METACRIAS Girault, 1913
PELOROTELOPSELLA Girault, 1913
URACRIAS Girault, 1913
METRIOCHARIS Silvestri, 1914
ACANTHENTEDON Dodd, 1917
METACRIASINUS Ghesquière, 1946

##### Notes

Some distribution data from Askew (1991b), Askew (1992b) and Graham (1971).

Doubtfully placed species of *Entedon* (see Bouček and Askew 1968):

[*amadocus* Walker, 1848 nom. dub.]

[*axia* Walker, 1848 nom. dub.]

[*charino* Walker, 1839 nom. dub.]

[*glabrio* Walker, 1846 nom. dub.]

[*mera* Walker, 1839 nom. dub.]

[*stennos* Walker, 1848 nom. dub.]

[*thonis* Walker, 1839 nom. dub.]

Species of *Entedon* removed from the British and Irish list (see GRAHAM 1971):

[*cioni* Thomson, 1878] [*ulmi* Erdös, 1954]

#### Entedon abdera

Walker, 1839

nigritarsis Erdös, 1944
punctatus Thomson, 1878

#### Entedon armigerae

Graham, 1971

##### Distribution

England

#### Entedon calcicola

Graham, 1971

##### Distribution

England

#### Entedon cionobius

Thomson, 1878

cinereae Erdös, 1961
fructicola Gumovsky, 1995

##### Distribution

England

#### Entedon diotimus

Walker, 1839

loti Erdös, 1944
transversalis Erdös, 1944

##### Distribution

England, Scotland, Wales

#### Entedon ergias

Walker, 1839

busiris Walker, 1839
merion Walker, 1839
annulatus (Förster, 1841, *Elachestus*)
albipes (Ratzeburg, 1844, *Eulophus*)
leucogramma (Ratzeburg, 1844, *Ichneumon*)

##### Distribution

England

#### Entedon fufius

Walker, 1846

montanus Erdös, 1951

##### Distribution

England

#### Entedon gracilior

Graham, 1971

##### Distribution

England

#### Entedon hercyna

Walker, 1839

aselli Erdös, 1954
elongatus Thomson, 1878

##### Distribution

England, Scotland, Wales

#### Entedon incultus

Askew, 1991

##### Distribution

England

##### Notes

Added by Askew (1991a)

#### Entedon insignis

Erdös, 1944

lixi Erdös, 1951

##### Distribution

England

#### Entedon longus

Bouček, 1968

longulus Erdös, 1944 preocc.

##### Notes

Only noted as probably British in Bouček and Askew (1968)​ and record requires confirmation.

#### Entedon marci

Askew, 1992

##### Notes

Added by Askew (1992b)

#### Entedon meliloti

Askew, 1992

##### Distribution

England, Scotland

##### Notes

Added by Askew (1992b)

#### Entedon metatarsalis

Thomson, 1878

erdoesi Delucchi, 1954

##### Distribution

England, Scotland

#### Entedon methion

Walker, 1839

gyoerfii Erdös, 1954

##### Distribution

England

#### Entedon molybdaenus

Erdös, 1944

##### Distribution

England

#### Entedon pallicrus

Erdös, 1944

##### Distribution

England

#### Entedon parvicalcar

Thomson, 1878

##### Distribution

England

#### Entedon pharnus

Walker, 1839

#### Entedon philiscus

Walker, 1851

##### Distribution

England, Wales

#### Entedon procioni

Erdös, 1944

urticarii Erdös, 1951

##### Distribution

England, Wales

#### Entedon punctiscapus

Thomson, 1878

##### Distribution

England, Wales

#### Entedon rumicis

Graham, 1971

##### Distribution

England, Wales

#### Entedon setifrons

Askew, 1991

##### Distribution

England

##### Notes

Added by Askew (1991b)

#### Entedon sparetus

Walker, 1839

longiventris Thomson, 1878
longiventrosus Dalla Torre, 1898
mecini Askew, 1992

##### Distribution

England, Wales, Ireland

##### Notes

**Figure 3.**
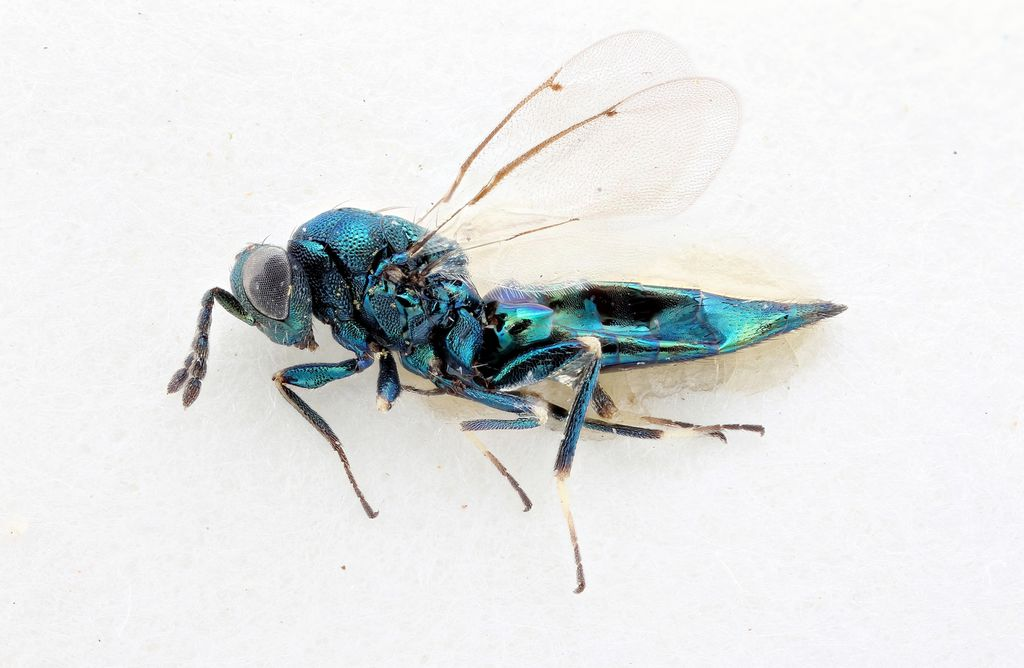
Eulophidae, Entedoninae: *Entedon sparetus* Walker female, BMNH specimen BMNH(E)953650 [/05/1976, Colnbrook, Berkshire, England, coll. Bouček] (photo K. McCormack)

See Fig. 3 for habitus

#### Entedon subovatus

Thomson, 1878

##### Distribution

England

#### Entedon sylvestris

Szelényi, 1981

##### Distribution

England

##### Notes

Added by Askew (1992b)

#### Entedon tibialis

(Nees, 1834)

Eulophus tibialis Nees, 1834
euphorion Walker, 1839
longicornis Erdös, 1944

##### Distribution

England

#### Entedon ulicis

(Perris, 1840)

Eulophus ulicis Perris, 1840

##### Distribution

England

#### Entedon zanara

Walker, 1839

albicrus Thomson, 1878

##### Distribution

England

#### Euderomphale

Girault, 1916

ALEURODIPHAGUS Nowicki, 1929

#### Euderomphale cerris

Erdös, 1961

##### Distribution

England

#### Euderomphale chelidonii

Erdös, 1966

##### Distribution

England

##### Notes

Added by Williams (1989)

#### Grahamia

Erdös, 1966

#### Grahamia clinius

(Walker, 1839)

Entedon clinius Walker, 1839
cleopater (Walker, 1839, *Eulophus*)

##### Distribution

England, Scotland

#### Ionympha

Graham, 1959

#### Ionympha carne

(Walker, 1839)

Entedon carne Walker, 1839
pedicellaris (Jansson, 1955, *Asecodes*)

##### Distribution

England, Scotland, Ireland

#### Ionympha ochus

(Walker, 1839)

Entedon ochus Walker, 1839
natras (Walker, 1839, *Cirrospilus*)
tegar (Walker, 1839, *Entedon*)
vagellius (Walker, 1839, *Entedon*)
antaradus (Walker, 1848, *Entedon*)

##### Distribution

England, Scotland

#### Mestocharis

Förster, 1878

#### Mestocharis bimacularis

(Dalman, 1820)

Entedon bimacularis Dalman, 1820
arisba (Walker, 1839, *Entedon*)
cyclospila Förster, 1878
militaris Rimsky-Korsakov, 1933
nearctica Yoshimoto, 1976

##### Distribution

England, Scotland, Wales

#### Neochrysocharis

Kurdjumov, 1912

RHICNOPELTOMYIA Girault, 1913
HETEROCHARIS Erdös, 1954
PHOLEMA Graham, 1963
FERMECERANISUS Szelényi, 1977

#### Neochrysocharis albiscapus

Erdös, 1954

##### Notes

Askew coll., det. Askew, added here

#### Neochrysocharis aratus

(Walker, 1838)

Cirrospilus aratus Walker, 1838
abastor (Walker, 1838, *Cirrospilus*)
abruptus (Thomson, 1878, *Derostenus*)
immaculata Kurdjumov, 1912

##### Distribution

England

#### Neochrysocharis arvensis

Graham, 1963

##### Distribution

England

#### Neochrysocharis chlorogaster

(Erdös, 1966)

Achrysocharella chlorogaster Erdös, 1966

#### Neochrysocharis clinias

(Walker, 1838)

Cirrospilus clinias Walker, 1838
aeneicrus Erdös, 1954

##### Distribution

England

#### Neochrysocharis cuprifrons

Erdös, 1954

##### Distribution

England

#### Neochrysocharis dimas

(Walker, 1839)

Entedon dimas Walker, 1839

##### Distribution

England

#### Neochrysocharis formosus

Westwood, 1833

phaenna (Walker, 1839, *Entedon*)
lunatus (Ratzeburg, 1848, *Entedon*)
ovulorum (Ratzeburg, 1848, *Entedon*)
obscuripes (Förster, 1861, *Chrysocharis*)
fullowayi (Crawford, 1913, *Derostenus*)
variipes (Crawford, 1913, *Derostenus*)
silvia (Girault, 1917, *Achrysocharella*)
camilli (Girault, 1917, *Achrysocharis*)

##### Distribution

England, Ireland

##### Notes

**Figure 4.**
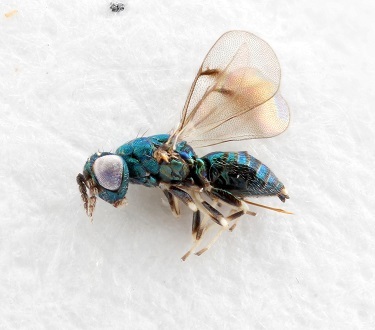
Eulophidae, Entedoninae: *Neochrysocharis formosus* (Westwood) female, BMNH specimen BMNH(E) 1414560 [/08/2013, Scilly Isles, Cornwall, England coll. Dale-Skey] (photo K. McCormack)

See Fig. 4 for habitus

#### Neochrysocharis longiventris

(Askew, 1979)

Chrysonotomyia longiventris Askew, 1979

##### Distribution

England

##### Notes

Added by Askew (1979)

#### Neochrysocharis microstoma

(Graham, 1963)

Pholema microstoma Graham, 1963

##### Distribution

England

#### Neochrysocharis trifolii

Erdös, 1961

##### Notes

Added by Askew et al. (2001)

#### Omphale

Haliday, 1833

SMARAGDITES Westwood, 1833
HOLCOPELTE Förster, 1856
SECODES Förster, 1856
CHRYSOCHAROIDEUS Ashmead, 1904
HOLCOPELTA Schulz, 1906
EUDEROMYIA Girault, 1913
CHRYSOCHAROMYIA Dodd, 1915
PAROMPHALE Girault & Dodd, 1915
RAPHAELONIA Girault, 1924
EUGERIUM Graham, 1959
EXODONTOMPHALE Bouček, 1984

##### Notes

Taxonomy and distribution from Hansson and Shevtsova (2012)

#### Omphale acamas

(Walker, 1839)

Entedon acamas Walker, 1839
laelius (Walker, 1839, *Entedon*)

##### Distribution

England

#### Omphale acuminata

Gijswijt, 1976

##### Distribution

England

##### Notes

Added by Askew (2003)

#### Omphale admirabilis

(Westwood, 1833)

Smaragdites admirabilis Westwood, 1833

##### Distribution

England

#### Omphale aethiops

Graham, 1963

##### Distribution

England, Scotland, Wales, Ireland

#### Omphale aetius

(Walker, 1839)

Entedon aetius Walker, 1839
Omphale aetius ?*marica* (Walker, 1839, *Entedon*)
metius (Walker, 1839, *Entedon*)

##### Distribution

England, Ireland

#### Omphale betulicola

Graham, 1963

##### Distribution

England, Scotland, Ireland

#### Omphale brevibuccata

Szelényi, 1978

##### Notes

Added by Askew (2003)

#### Omphale brevis

Graham, 1963

##### Distribution

England

#### Omphale breviventris

Graham, 1970

##### Distribution

England

#### Omphale chryseis

Graham, 1963

##### Distribution

England, Ireland

#### Omphale clymene

(Walker, 1839)

Entedon clymene Walker, 1839

##### Distribution

England

#### Omphale clypealis

(Thomson, 1878)

Derostenus clypealis Thomson, 1878

##### Distribution

England

#### Omphale coilus

(Walker, 1839)

Entedon coilus Walker, 1839
lyaeus (Walker, 1839, *Entedon*)
montana Erdös, 1951

#### Omphale connectens

Graham, 1963

##### Distribution

England, Scotland, Ireland

#### Omphale cornula

Hansson & Shevtsova, 2012

##### Distribution

England

##### Notes

Added by Hansson and Shevtsova (2012)

#### Omphale epaphus

(Walker, 1839)

Entedon epaphus Walker, 1839
Omphale epaphus ?*varipes* (Thomson, 1878, *Derostenus*)

##### Distribution

England, Scotland, Ireland

#### Omphale erginnus

(Walker, 1839)

Entedon erginnus Walker, 1839

##### Distribution

England

#### Omphale erugata

Hansson & Shevtsova, 2012

##### Distribution

England

##### Notes

Added by Hansson and Shevtsova (2012)

#### Omphale euphorbiae

Hansson & Shevtsova, 2012

##### Distribution

England

##### Notes

Added by Hansson and Shevtsova (2012)

#### Omphale grahami

Gijswijt, 1976

##### Distribution

England

##### Notes

Recorded by Graham (1963) as an indet. species and subsequently described by M.J. Gijswijt but not including the English specimens.

#### Omphale incognita

Hansson & Shevtsova, 2012

##### Distribution

England

##### Notes

Added by Hansson and Shevtsova (2012)

#### Omphale isander

(Walker, 1839)

Cirrospilus isander Walker, 1839
isander (Walker, 1848, *Entedon*) preocc.
fimbriatus (Jansson, 1955, *Asecodes*)

##### Notes

*O. isander* (Walker, 1848, *Entedon*): see Bouček and Askew (1968); currently listed as a valid species of *Entedon* in the UCD.

#### Omphale lugens

(Nees, 1834)

Eulophus lugens Nees, 1834
navius (Walker, 1839, *Entedon*)
coactus (Ratzeburg, 1848, *Entedon*)
fagi (Förster, 1856, *Secodes*)

##### Distribution

England, Scotland, Wales, Ireland

#### Omphale lugubris

Askew, 2003

##### Distribution

England, Ireland

#### Omphale matrana

Erdös, 1954

##### Distribution

England

##### Notes

Added by Askew (2003)

#### Omphale nitens

Graham, 1963

##### Distribution

England

#### Omphale obscura

(Förster, 1841)

Elachestus obscurus Förster, 1841
fulvipes (Förster, 1861, *Holcopelte*)

##### Distribution

England, Wales

#### Omphale phaola

(Walker, 1839)

Entedon phaola Walker, 1839

##### Distribution

England, Ireland

#### Omphale phruron

(Walker, 1839)

Entedon phruron Walker, 1839
teresis Askew, 2003

##### Distribution

England, Scotland, Wales, Ireland

#### Omphale rubigus

(Walker, 1839)

Entedon rubigus Walker, 1839
Omphale rubigus ?*rhesus* (Walker, 1839, *Entedon*)

##### Distribution

England, Scotland

##### Notes

*O. rhesus* is not mentioned by Hansson and Shevtsova (2012)

#### Omphale salicis

(Haliday, 1833)

Entedon salicis Haliday, 1833
subulatus (Nees, 1834, *Eulophus*)
terebrator (Förster, 1841, *Eulophus*)

##### Distribution

England, Scotland, Wales

#### Omphale sulciscuta

(Thomson, 1878)

Derostenus sulciscuta Thomson, 1878

##### Distribution

England

#### Omphale telephe

(Walker, 1839)

Entedon telephe Walker, 1839

##### Distribution

England

#### Omphale theana

(Walker, 1839)

Entedon theana Walker, 1839
ithonus (Walker, 1839, *Entedon*)
radialis (Thomson, 1878, *Derostenus*)
americana (Girault, 1916, *Achrysocharella*)

##### Distribution

England, Scotland, Wales

#### Omphale versicolor

(Nees, 1834)

Eulophus versicolor Nees, 1834
anthylla (Walker, 1839, *Entedon*)

##### Distribution

England, Scotland

#### Pediobius

Walker, 1846

MICROTERUS Spinola, 1811
RHOPALOTUS Förster, 1856
PLEUROTROPIS Förster, 1856
SPARTIOPHILUS Rondani, 1872
HEPTOMERUS Rondani, 1874
CLUTHAIRA Cameron, 1912
AMESTOCHARIS Girault, 1913
CHRYSOATOMOIDES Girault, 1913
EPACRIAS Girault, 1913
MESTOCHAROIDEUS Girault, 1913
PSEUDACRIAS Girault, 1913
ENTEDONOMYIA Girault, 1915
ENTEDONOPSEUS Girault, 1915
HORISMENOPSIS Girault, 1915
MESTOCHAROMYIA Girault, 1915
NEOPSEUDACRIAS Girault, 1915
EPIPLEUROTROPIS Girault, 1917
PSEUDACRIASOIDES Girault, 1917
MESENTEDON Girault, 1920

#### Pediobius alaspharus

(Walker, 1839)

Entedon alaspharus Walker, 1839

#### Pediobius alcaeus

(Walker, 1839)

Entedon alcaeus Walker, 1839
beon (Walker, 1839, *Entedon*)
politus (Ratzeburg, 1848, *Elachestus*)
longfellowi (Girault, 1917, *Epipleurotropis*)

##### Distribution

England, Scotland, Wales

#### Pediobius brachycerus

(Thomson, 1878)

Pleurotropis brachycerus Thomson, 1878
wilderi (Howard, 1892, *Mestocharis*)
aquatica (Erdös, 1954, *Pleurotropis*)

##### Distribution

England

#### Pediobius calamagrostidis

Dawah, 1988

##### Notes

Added by Dawah (1988)

#### Pediobius cassidae

Erdös, 1958

##### Distribution

England

#### Pediobius claridgei

Dawah, 1988

##### Notes

Added by Dawah (1988)

#### Pediobius claviger

(Thomson, 1878)

Pleurotropis claviger Thomson, 1878

##### Distribution

England

#### Pediobius clita

(Walker, 1839)

Entedon clita Walker, 1839

##### Distribution

England

#### Pediobius coxalis

Bouček, 1965

##### Notes

BMNH, det. Hansson, added here

#### Pediobius crassicornis

(Thomson, 1878)

Pleurotropis crassicornis Thomson, 1878
albitarsis (Ashmead, 1888, *Asecodes*)
tarsalis (Ashmead, 1894, *Holcopelte*)
howardi (Crawford, 1910, *Pleurotropis*)
ashmeadi (Crawford, 1912, *Pleurotropis*)

##### Distribution

England

#### Pediobius dactylicola

Dawah, 1988

##### Notes

Added by Dawah (1988)

#### Pediobius deschampsiae

Dawah, 1988

##### Notes

Added by Dawah (1988)

#### Pediobius dorycniellae

Erdös, 1961

#### Pediobius epeus

(Walker, 1839)

Entedon epeus Walker, 1839
ulmi (Erdös, 1954, *Pleurotropis*)

##### Distribution

England

#### Pediobius epigonus

(Walker, 1839)

Entedon epigonus Walker, 1839
isomerus (Förster, 1861, *Pleurotropis*)
nigripes (Lindeman, 1887, *Semiotellus*)

##### Distribution

England, Scotland

#### Pediobius eubius

(Walker, 1839)

Entedon eubius Walker, 1839
angularis (Förster, 1841, *Elachestus*)
nitifrons (Thomson, 1878, *Pleurotropis*)
utahensis (Crawford, 1913, *Pleurotropis*)
longus (Girault, 1916, *Pleurotropis*)
perdubius (Girault, 1917, *Amestocharis*)

##### Distribution

England, Scotland

#### Pediobius facialis

(Giraud, 1863)

Pleurotropis facialis Giraud, 1863
albitarsis (Ashmead, 1888, *Entedon*)
sexdentatus (Girault, 1916, *Pseudacrias*)
olethreutidis (Gahan, 1932, *Pleurotropis*)
albae Erdös, 1961

##### Distribution

England, Wales

#### Pediobius festucae

Dawah, 1988

##### Notes

Added by Dawah (1988)

#### Pediobius foliorum

(Geoffroy, 1785)

Cynips foliorum Geoffroy, 1785
cothurnatus (Nees, 1834, *Elachestus*)
gradualis (Nees, 1834, *Elachestus*)
argon (Walker, 1839, *Entedon*)
splendens (Cook & Davis, 1891, *Derostenus*)
kraussei (Wolff, 1916, *Chrysocharis*)

##### Distribution

England

#### Pediobius longicornis

(Erdös, 1954)

Pleurotropis longicornis Erdös, 1954

##### Distribution

England

##### Notes

Added by Jennings (2007b)

#### Pediobius lysis

(Walker, 1839)

Entedon lysis Walker, 1839
corytus (Walker, 1839, *Entedon*)
sosarmus (Walker, 1839, *Entedon*)
albitarsis (Fonscolombe, 1840, *Cynips*)
cyniphidum (Ratzeburg, 1848, *Elachestus*)
cribrifrons (Thomson, 1878, *Pleurotropis*)
naso (Erdös, 1951, *Pleurotropis*)

##### Distribution

England, Scotland

#### Pediobius metallicus

(Nees, 1834)

Eulophus metallicus Nees, 1834
acantha (Walker, 1839, *Entedon*)
amyntas (Walker, 1839, *Entedon*)
caenus (Walker, 1839, *Entedon*)
caeruleonitens (Rondani, 1874, *Heptomerus*)
brevicornis (Thomson, 1878, *Pleurotropis*)
rugosithorax (Crawford, 1912, *Pleurotropis*)
kansensis (Girault, 1918, *Pleurotropis*)
helianthemellae Erdös, 1961

##### Distribution

England, Scotland, Wales

#### Pediobius nigritarsis

(Thomson, 1878)

Pleurotropis nigritarsis Thomson, 1878
benefica (Gahan, 1921, *Pleurotropis*)

##### Distribution

England, Scotland

#### Pediobius obscurus

Dawah & Al-Haddad, 2002

##### Notes

Added by Dawah et al. (2002)

#### Pediobius phalaridis

Dawah, 1988

##### Notes

Added by Dawah (1988)

#### Pediobius phyllotretae

(Riley, 1884)

Pleurotropis phyllotretae Riley, 1884
glabratus Bouček, 1965

#### Pediobius planiventris

(Thomson, 1878)

Pleurotropis planiventris Thomson, 1878

##### Distribution

England, Ireland

#### Pediobius pyrgo

(Walker, 1839)

Entedon pyrgo Walker, 1839
pyralidum (Audouin, 1842, *Eulophus*)
complaniusculus (Ratzeburg, 1852, *Elachestus*)
substrigosa (Thomson, 1878, *Pleurotropis*)
nawai (Ashmead, 1904, *Derostenus*)
chalcidiphagus (Szelényi, 1957, *Rhopalotus*)

#### Pediobius saulius

(Walker, 1839)

Entedon saulius Walker, 1839
linus (Walker, 1839, *Entedon*)
obscuripes (Ratzeburg, 1844, *Eulophus*)
strigiscuta (Thomson, 1878, *Pleurotropis*)
grandii Ferrière, 1954

##### Distribution

England

#### Pediobius termerus

(Walker, 1839)

Entedon termerus Walker, 1839
nephthe (Walker, 1839, *Entedon*)
clinus (Walker, 1844, *Horismenus*)

##### Distribution

England, Scotland

#### Pediobius tetratomus

(Thomson, 1878)

Pleurotropis tetratomus Thomson, 1878

##### Distribution

England, Scotland

#### Entiinae

Hedqvist, 1974

EUDERINAE Förster, 1856 preocc.

#### Astichus

Förster, 1856

CLOSTEROCEROIDES Girault, 1913
CLOSTEROMPHALE Girault & Dodd, 1915
CLOSTEROMYIIA Girault, 1920

#### Astichus arithmeticus

(Förster, 1851)

Euderus arithmeticus Förster, 1851

#### Astichus maculatus

Hedqvist, 1969

##### Distribution

England, Scotland

##### Notes

**Figure 5.**
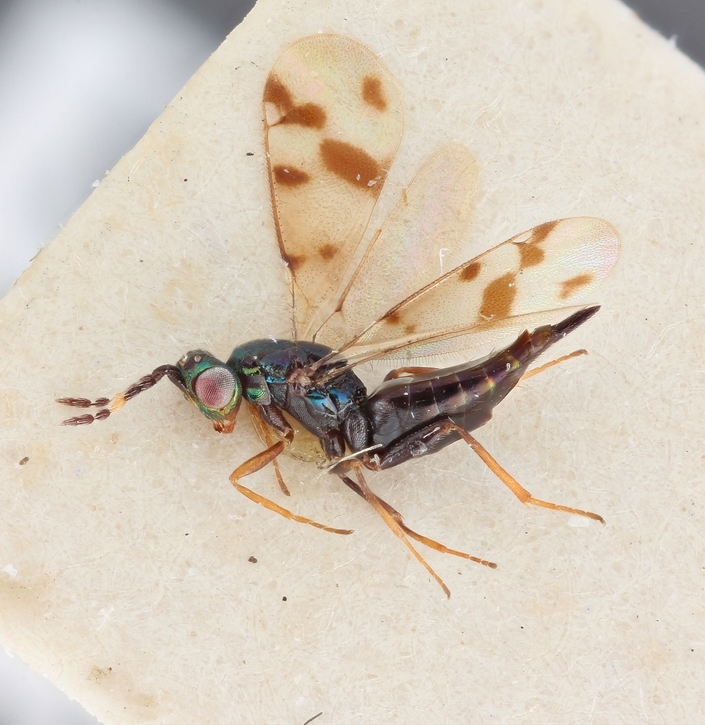
Eulophidae, Entiinae: *Astichus maculatus* Hedqvist female, BMNH specimen BMNH(E) 1414499 [/06/1965, Nethy Bridge, Inverness, Scotland coll. Graham] (photo K. McCormack)

BMNH, det. Straka, added here. See Fig. 5 for habitus

#### Astichus solutus

Förster, 1856

#### Euderus

Haliday, 1844

OMPHALOMORPHA Girault, 1913
SECODELLA Girault, 1913
SECODES Girault, 1913
ALLOMPHALE Silvestri, 1914
SECODELLOIDEA Girault, 1917
SECODOIDEA Gahan & Fagan, 1923
PAREUDERUS Ferrière, 1931

#### Euderus albitarsis

(Zetterstedt, 1838)

Entedon albitarsis Zetterstedt, 1838
amphis (Walker, 1839, *Entedon*)
mithras (Walker, 1839, *Entedon*)

#### Euderus viridis

Thomson, 1878

#### Parasecodella

Girault, 1915

EUDERASTICHUS Bouček, 1963

#### Parasecodella obscura

(Thomson, 1878)

Euderus obscurus Thomson, 1878
almus (Erdös, 1951, *Allocerastichus*)

##### Distribution

England, Wales

#### Eulophinae

Westwood, 1829

ELASMIDAE Förster, 1856
ELASMINAE Förster, 1856

#### Aulogymnus

Förster, 1851

OLYNX Förster, 1856
CYNIPHOCTONUS Reinhard, 1858
OLINX Reinhard, 1858 unjustified emendation
OPHELINOIDEUS Ashmead, 1904
SCOTOLINX Ashmead, 1904
PSEUDIGLYPHELLA Girault, 1913
MIROLYNX Girault, 1916
PSEUDOLYNX Girault, 1916

#### Aulogymnus arsames

(Walker, 1838)

Cirrospilus arsames Walker, 1838
lineaticeps (Mayr, 1877, *Olynx*)

#### Aulogymnus euedoreschus

(Walker, 1839)

Eulophus euedoreschus Walker, 1839
fulvicrus (Thomson, 1878, *Olinx*)

#### Aulogymnus gallarum

(Linnaeus, 1761)

Ichneumon gallarum Linnaeus, 1761
rotundiventris (Thomson, 1878, *Olinx*)
pulchra (Mayr, 1877, *Olynx*)

##### Distribution

England

#### Aulogymnus skianeuros

(Ratzeburg, 1844)

Eulophus skianeuros Ratzeburg, 1844

#### Aulogymnus trilineatus

(Mayr, 1877)

Olinx trilineata Mayr, 1877

#### Cirrospilus

Westwood, 1832

ATOPOSOMA Masi, 1907
ATOPOSOMOIDEA Howard, 1910
WINNEMANA Crawford, 1911
GYROLASELLA Girault, 1913
ACHRYSOCHARELLOIDEA Girault, 1913
CIRROSPILOMELLA Girault, 1913
PSEUDIGLYPHOMYIA Girault, 1913
CIRROSPILOPSIS Girault, 1915
PARZAGRAMMOSOMA Girault, 1916
GIRAULTIA Gahan & Fagan, 1923
AUSTROLYNX Girault, 1929
OOTETRASTICHOIDES Ii, 1936
PLESIOSPILUS Ferrière, 1954

#### Cirrospilus argei

(Crawford, 1911)

Winnemana argei Crawford, 1911

#### Cirrospilus curvineurus

Askew, 1965

#### Cirrospilus diallus

Walker, 1838

quadrimaculatus (Förster, 1841, *Eulophus*)
flavomaculatus (Ratzeburg, 1844, *Eulophus*)
walkeri Stephens, 1846
punctatus (Ratzeburg, 1848, *Entedon*)

##### Distribution

England

#### Cirrospilus elegantissimus

Westwood, 1832

unistriatus (Förster, 1841, *Eulophus*)

#### Cirrospilus elongatus

Bouček, 1959

##### Distribution

England

#### Cirrospilus lyncus

Walker, 1838

unifasciatus (Förster, 1841, *Eulophus*)
caudatulus Thomson, 1878

##### Distribution

England

#### Cirrospilus pictus

(Nees, 1834)

Eulophus pictus Nees, 1834
thasus Walker, 1838
arcuatus (Förster, 1841, *Eulophus*)
bifasciatus Walker, 1872
ogimae (Howard, 1910, *Atoposomoidea*)
nigriscutellaris Sheng & Wang, 1992
huangyaensis Sheng, 1994

#### Cirrospilus pinicolus

Askew, 1984

##### Distribution

England

##### Notes

Added by Askew (1984b)

#### Cirrospilus salatis

Walker, 1838

immaculatus Thomson, 1878

##### Distribution

England

#### Cirrospilus singa

Walker, 1838

##### Distribution

Scotland

#### Cirrospilus viticola

(Rondani, 1877)

Omphale viticola Rondani, 1877
subviolaceus Thomson, 1878
simulator Masi, 1933
luteus Bukovskii, 1938
setulosus Graham, 1959

##### Distribution

England

#### Cirrospilus vittatus

Walker, 1838

phorbas Walker, 1838
lineatus (Förster, 1841, *Eulophus*)
nigrolineata (Crawford, 1913, *Zagrammosoma*)
nigrolineatum (Crawford, 1913, *Zagrammosoma*)
pulcherrima (Mercet, 1916, *Atoposomoidea*)
sanguinea (Girault, 1916, *Zagrammosoma*)
sanguineum (Girault, 1916, *Zagrammosoma*)
donatellae Mariani, 1942
hytomyzae (Ishii, 1953, *Atoposoma*)

##### Distribution

England, Scotland

#### Colpoclypeus

Lucchese, 1941

#### Colpoclypeus florus

(Walker, 1839)

Eulophus florus Walker, 1839
silvestrii Lucchese, 1941

##### Distribution

England, Scotland, Wales

#### Dahlbominus

Hincks, 1945

MICROPLECTRON Dahlbom, 1857 preocc.

#### Dahlbominus fuscipennis

(Zetterstedt, 1838)

Entedon fuscipennis Zetterstedt, 1838

#### Diaulinopsis

Crawford, 1912

#### Diaulinopsis arenaria

(Erdös, 1951)

Cycloscapus arenarius Erdös, 1951

##### Distribution

England

##### Notes

BMNH, det. Hansson, added here

#### Dicladocerus

Westwood, 1832

SOLENOTUS Förster, 1856
DIGLYPHIS Thomson, 1878
SOLENONOTUS Schulz, 1906

#### Dicladocerus breviramulus

Bouček, 1959

#### Dicladocerus euryalus

(Haliday, 1844)

Eulophus euryalus Haliday, 1844
aeneiscapus (Thomson, 1878, *Diglyphis*)

##### Distribution

Scotland

#### Dicladocerus westwoodii

Westwood, 1832

aepytus (Walker, 1839, *Eulophus*)
battis (Walker, 1839, *Eulophus*)
viridis (Förster, 1856, *Solenotus*)
rugifrons (Thomson, 1878, *Diglyphus*)

##### Distribution

England

#### Diglyphus

Walker, 1844

DIAULUS Ashmead, 1904
DIAULINUS Schulz, 1906
CYCLOSCAPUS Erdös & Novicky, 1951

#### Diglyphus chabrias

(Walker, 1838)

Cirrospilus chabrias Walker, 1838

##### Distribution

England, Wales

#### Diglyphus crassinervis

Erdös, 1958

##### Distribution

England

#### Diglyphus isaea

(Walker, 1838)

Cirrospilus isaea Walker, 1838
lycophron (Walker, 1838, *Cirrospilus*)
medidas (Walker, 1838, *Cirrospilus*)
gracilis (Goureau, 1851, *Entedon*)
bisannulatus Förster, 1861
ornatus Förster, 1861
clavicornis Walker, 1872
phytomyzae (Rondani, 1877, *Elachistus*)
viridis (Thomson, 1878, *Solenotus*)

##### Distribution

England, Scotland, Wales, Ireland

#### Diglyphus minoeus

(Walker, 1838)

Cirrospilus minoeus Walker, 1838
abron (Walker, 1838, *Cirrospilus*)
amelon (Walker, 1839, *Eulophus*)
deldon (Walker, 1839, *Cirrospilus*)
myron (Walker, 1839, *Cirrospilus*)
smilis (Walker, 1839, *Cirrospilus*)

##### Distribution

England, Wales

#### Diglyphus pachyneurus

Graham, 1963

##### Distribution

England

#### Diglyphus poppoea

Walker, 1848

##### Distribution

England, Scotland, Wales, Ireland

#### Diglyphus pusztensis

(Erdös & Novicky, 1951)

Cycloscapus pusztensis Erdös & Novicky, 1951
tibiscanus Erdös, 1958
fulvipes Erdös, 1961

##### Distribution

Wales

#### Dimmockia

Ashmead, 1904

ENCOPA Graham, 1959

#### Dimmockia brevicornis

(Erdös, 1954)

Eulophus brevicornis Erdös, 1954

#### Elachertus

Spinola, 1811

ELACHISTUS Förster, 1856
ARDALUS Howard, 1897
CIRROSPILOIDEUS Ashmead, 1904
DIGLYPHOMORPHELLA Girault, 1913
PARENTEDON Girault, 1913
PSEUDELACHERTEUS Girault, 1913
SYMPIESOMORPHELLEUS Girault, 1913
ARDALOIDES Girault, 1915
EUPLECTROMORPHELLA Girault, 1915
PROARDALUS Girault & Dodd, 1915
EPARDALUS Girault, 1917
PETEENUS Erdös, 1961

##### Notes

Doubtfully placed species of *Elachertus* (see Bouček and Askew 1968):

[*erse* (Walker, 1839, *Eulophus*) nom. dub.]

[*suada* (Walker, 1839, *Eulophus*) nom. dub.]

#### Elachertus anthophilae

Bouček, 2002

##### Notes

Added by Boucek (2002)

#### Elachertus artaeus

(Walker, 1839)

Eulophus artaeus Walker, 1839

#### Elachertus bisurmanus

Erdös, 1966

##### Notes

Added by Askew et al. (2001)

#### Elachertus charondas

(Walker, 1839)

Eulophus charondas Walker, 1839
orsus (Walker, 1839, *Eulophus*)
punctiscuta (Thomson, 1878, *Elachistus*)
monachae (Ruschka & Fulmek, 1915, *Elachistus*)

##### Distribution

England

#### Elachertus fenestratus

Nees, 1834

argissa (Walker, 1839, *Eulophus*)
eurybates (Walker, 1839, *Eulophus*)
saon (Walker, 1839, *Eulophus*)
opaculus (Thomson, 1878, *Elachistus*)
proteoteratis (Howard, 1885, *Elachistus*)
coxalis (Howard, 1885, *Elachistus*)
veridoeneus (Provancher, 1887, *Euplectrus*)
pini Gahan, 1927

##### Distribution

England

#### Elachertus gallicus

Erdös, 1958

#### Elachertus inunctus

Nees, 1834

eucrate (Walker, 1839, *Eulophus*)
florianus (Walker, 1839, *Eulophus*)
neleus (Walker, 1839, *Eulophus*)
sublaevis (Thomson, 1878, *Elachistus*)

##### Distribution

England

#### Elachertus isadas

(Walker, 1839)

Eulophus isadas Walker, 1839
scyllis (Walker, 1848, *Eulophus*)
ticida (Walker, 1839, *Eulophus*)
splendens (Förster, 1841, *Elachestus*)
viridulus (Thomson, 1878, *Elachistus*)

##### Distribution

England, Scotland, Wales

#### Elachertus lateralis

(Spinola, 1808)

Diplolepis lateralis Spinola, 1808
carinatus (Ratzeburg, 1848, *Elachestus*)
aeneiscapus (Thomson, 1878, *Elachistus*)
petiolatus (Thomson, 1878, *Elachistus*)
clavatus Erdös, 1966

##### Distribution

England, Scotland, Wales

#### Elachertus pilosiscuta

Bouček, 1971

##### Distribution

Scotland

#### Elasmus

Westwood, 1833

ANEURE Nees, 1834
HEPTOCONDYLA Rondani, 1877
CYCLOPLEURA Cameron, 1913
AUSTELASMUS Riek, 1967

##### Notes

Species of *Elasmus* removed from the British and Irish list:

[*rufiventris* Ferrière, 1947] - listed by Bouček and Graham (1978)​ in error

#### Elasmus anius

Walker, 1846

#### Elasmus flabellatus

(Fonscolombe, 1832)

Eulophus flabellatus Fonscolombe, 1832
scutellaris (Nees, 1834, *Aneure*)
rhipicerus (Förster, 1841, *Aneure*)
giraudi Ferrière, 1947

#### Elasmus nudus

(Nees, 1834)

Aneure nuda Nees, 1834
albipennis Thomson, 1878

##### Notes

Omitted by Bouček and Graham (1978)​

#### Eulophus

Geoffroy, 1762

COMEDO Schrank, 1802
CRATOTECHUS Thomson, 1878

##### Notes

Doubtfully placed species of Eulophus (see Bouček and Askew 1968​):

[*aepulo* Walker, 1839 nom. dub.]

[*agathyllus* Walker, 1846 nom. dub.]

[*boeotus* Walker, 1839 nom. dub.]

[*callidius* Walker, 1839 nom. dub.]

[*carbo* Walker, 1839 nom. dub.]

[*eucritus* Walker, 1839 nom. dub.]

[*iapetus* Walker, 1839 nom. dub.]

[*kirbii* Curtis & Westwood, 1826 nom. dub.]

[*orsinus* Walker, 1839 nom. dub.]

[*pythodorus* Walker, 1848 nom. dub.]

[*sancus* Walker, 1839 nom. dub.]

[*trachalus* Walker, 1839 nom. dub.]

​[*veturius* Walker, 1848 nom. dub.]

#### Eulophus abdominalis

Nees, 1834

anatole Walker, 1839
longicornis (Thomson, 1878, *Cratotechus*)

##### Distribution

England

#### Eulophus larvarum

(Linnaeus, 1758)

Ichneumon larvarum Linnaeus, 1758
aeneicoxa (Thomson, 1878, *Cratotechus*)

##### Distribution

England

#### Eulophus pennicornis

Nees, 1834

plumicornis (Dalman, 1820, *Entedon*)
fuliginosus Nees, 1834
drupes Walker, 1839
opaculus (Thomson, 1878, *Cratotechus*)

##### Distribution

England

#### Eulophus ramicornis

(Fabricius, 1781)

Ichneumon ramicornis Fabricius, 1781
circularis (Geoffroy, 1785, *Cynips*)
eulophus (Geoffroy, 1785, *Cynips*)
damicornis Kirby, 1825
dimidiatus Nees, 1834
bombycicornis Ratzeburg, 1844
phalaenarum Ratzeburg, 1844
fumatus Ratzeburg, 1848
mulierosus Karsch, 1879
hoplitis (Crawford, 1911, *Cratotechus*)
nigribasis Gradwell, 1957

#### Eulophus rhamnius

Walker, 1848

#### Eulophus smerinthicida

Bouček, 1959

#### Eulophus thespius

Walker, 1839

ungularis (Thomson, 1878, *Cratotechus*)

#### Euplectrus

Westwood, 1832

DIPLECTRON Dahlbom, 1857
PACHYSCAPHA Howard, 1897
REKABIA Cameron, 1905
HETEROSCAPUS Brèthes, 1918 preocc.
HETEROSCAPISCUS Ghesquière, 1946

#### Euplectrus bicolor

(Swederus, 1795)

Pteromalus bicolor Swederus, 1795
albiventris (Spinola, 1811, *Elachertus*)
Euplectrus bicolor ?*maculiventris* Westwood, 1832
intactus Walker, 1872

##### Distribution

England

#### Euplectrus platyhypenae

Howard, 1885

nigriceps Ferrière, 1941

##### Notes

Only questionably noted as British, as *E. nigriceps*, by Graham (1963)

#### Hemiptarsenus

Westwood, 1833

NOTANISOMORPHA Ashmead, 1904
ERIGLYPTOIDEUS Girault, 1913
HEMIPTARSENOIDEUS Girault, 1916
NEODIMMOCKIA Dodd, 1917

#### Hemiptarsenus fulvicollis

Westwood, 1833

anementus (Walker, 1839, *Eulophus*)
catreus (Walker, 1839, *Eulophus*)
dercynus (Walker, 1839, *Eulophus*)
faula (Walker, 1839, *Eulophus*)
pulcherrimus (Förster, 1841, *Elachestus*)
tarandus (Förster, 1841, *Eulophus*)
albicoxa Thomson, 1878

##### Distribution

England

#### Hemiptarsenus ornatus

(Nees, 1834)

Encyrtus ornatus Nees, 1834
dropion (Walker, 1839, *Eulophus*)
gratus (Goureau, 1851, *Entedon*)
lepidus (Goureau, 1851, *Entedon*)
opicornis (Förster, 1861, *Eulophus*)

#### Hemiptarsenus unguicellus

(Zetterstedt, 1838)

Entedon unguicellus Zetterstedt, 1838
hedila (Walker, 1839, *Eulophus*)
hegemon (Walker, 1839, *Eulophus*)
ianthea (Walker, 1839, *Eulophus*)
laogonus (Walker, 1839, *Eulophus*)
myodes (Walker, 1839, *Eulophus*)
nonus (Walker, 1839, *Eulophus*)
nycteus (Walker, 1839, *Eulophus*)
piscus (Walker, 1839, *Eulophus*)
villius (Walker, 1839, *Eulophus*)
alce (Walker, 1840, *Eulophus*)
alcicornis (Förster, 1841, *Eulophus*)
antilope (Förster, 1841, *Eulophus*)
harmocerus (Förster, 1841, *Eulophus*)
opicornis (Förster, 1841, *Eulophus*)
pellucens (Förster, 1841, *Elachestus*)
sexradiatus (Förster, 1841, *Eulophus*)
cinctipes (Stephens, 1846, *Eulophus*)
divisus (Walker, 1872, *Eulophus*)
drusilla (Walker, 1839, *Eulophus*)
gonippus (Walker, 1839, *Eulophus*)

#### Hemiptarsenus waterhousii

Westwood, 1833

arenarius Erdös, 1951

#### Hyssopus

Girault, 1916

HYSSOPISCUS Ghesquière, 1946
CRATAEPOIDES Masi, 1955

#### Hyssopus geniculatus

(Hartig, 1838)

Eulophus geniculatus Hartig, 1838
russoi (Zinna, 1955, *Crataepoides*)

#### Hyssopus nigritulus

(Zetterstedt, 1838)

Entedon nigritulus Zetterstedt, 1838
aphaca (Walker, 1839, *Cirrospilus*)

##### Distribution

England, Scotland

#### Hyssopus olivaceus

(Thomson, 1878)

Elachistus olivaceus Thomson, 1878

##### Distribution

England, Wales

#### Microlycus

Thomson, 1878

NEOLACHERTUS Szelényi, 1976

#### Microlycus erdoesi

Bouček, 1959

##### Distribution

England

##### Notes

Added by Askew (1992d)

#### Microlycus harcalo

(Walker, 1852)

Eulophus harcalo Walker, 1852

##### Distribution

England

##### Notes

Askew (1992a) considers this name to be a nomen dubium.

#### Microlycus heterocerus

Thomson, 1878

##### Distribution

England

##### Notes

Added by Askew et al. (2001)

#### Miotropis

Thomson, 1878

STENOMESIOIDEUS Ashmead, 1904
MIONOTROPIS Sculz, 1906
STENOMESIOIDEA Girault, 1916

#### Miotropis unipuncta

(Nees, 1834)

Eulophus unipunctus Nees, 1834
articas (Walker, 1839, *Cirrospilus*)
quadrifasciatus (Förster, 1841, *Eulophus*)
simplex Thomson, 1878
sulcicrista Thomson, 1878

##### Distribution

England, Wales

#### Necremnus

Thomson, 1878

##### Notes

Some distribution data from Gebiola et al. (2015)​

#### Necremnus aenigmaticus

Gibson, 2015

##### Distribution

England

##### Notes

Added by Gebiola et al. (2015)​

#### Necremnus artynes

(Walker, 1839)

Eulophus artynes Walker, 1839
subcontiguus (Thomson, 1878, *Eulophus*)

##### Distribution

England, Wales

#### Necremnus capitatus

Bouček, 1959

#### Necremnus cosconius

(Walker, 1839)

Eulophus cosconius Walker, 1839
amempsimus (Walker, 1839, *Eulophus*)
punctifrons Thomson, 1878

##### Distribution

England, Scotland, Wales

#### Necremnus croton

(Walker, 1839)

Eulophus croton Walker, 1839

##### Distribution

England

#### Necremnus flagellaris

Askew, 1992

##### Distribution

Scotland

##### Notes

Added by Askew (1992a)

#### Necremnus folia

(Walker, 1839)

Eulophus folia Walker, 1839
diyllus (Walker, 1839, *Eulophus*)

##### Distribution

Ireland

#### Necremnus leucarthros

(Nees, 1834)

Eulophus leucarthros Nees, 1834
anaxippus (Walker, 1846, *Eulophus*)
cornucopiae (Förster, 1841, *Eulophus*)
teratocerus (Förster, 1861, *Eulophus*)

##### Distribution

England, Wales

#### Necremnus metalarus

(Walker, 1839)

Eulophus metalarus Walker, 1839

##### Distribution

England, Ireland

#### Necremnus rhaecus

(Walker, 1839)

Eulophus rhaecus Walker, 1839

##### Distribution

England

##### Notes

Removed from synonymy under *N. folia* in Gebiola et al. (2015)​

#### Necremnus tidius

(Walker, 1839)

Eulophus tidius Walker, 1839
metanira (Walker, 1839, *Eulophus*)
zeugma (Walker, 1839, *Eulophus*)
mamurius (Walker, 1848, *Eulophus*)
duplicatus Gahan, 1941

##### Distribution

England

##### Notes

*N. hippia* (Walker, 1839, *Eulophus*) was removed from synonymy under *N. tidius* by Gebiola et al. (2015)​; there is no evidence that *N. hippia* has been recorded from Britain as Walker did not specify a type locality and only a ‘var.’ was described as having being collected from near London, which is not equivalent to *N. hippia* sensu Gebiola et al. (2015)​.

#### Platyplectrus

Ferrière, 1941

AUTOPLECTRUS Gadd, 1945

#### Platyplectrus laeviscuta

(Thomson, 1878)

Euplectrus laeviscuta Thomson, 1878

#### Pnigalio

Schrank, 1802

TINEOPHAGA Rondani, 1868
NOTANISOMORPHOMYIA Girault, 1913

##### Notes

Doubtfully placed species of *Pnigalio* (see Bouček and Askew (1968)):

[*cruciatus* (Ratzeburg, 1848, *Pteromalus*) nom. dub.]

#### Pnigalio agraules

(Walker, 1839)

Eulophus agraules Walker, 1839
barbarus (Förster, 1841, *Eulophus*)
tischeriae (Rondani, 1868, *Tineophaga*)
orchesticida (Rondani, 1877, *Spartiophilus*)
populifoliellae (Erdös, 1954, *Eulophus*)
mediterraneus Ferrière & Delucchi, 1957

#### Pnigalio attis

(Walker, 1839)

Eulophus attis Walker, 1839

#### Pnigalio epilobii

Bouček, 1966

#### Pnigalio longulus

(Zetterstedt, 1838)

Entedon longulus Zetterstedt, 1838
pisidice (Walker, 1839, *Eulophus*)
arcticus (Thomson, 1878, *Teleogmus*)

##### Distribution

England, Scotland

#### Pnigalio nemati

(Westwood, 1838)

Eulophus nemati Westwood, 1838
tischbeinii (Ratzeburg, 1848, *Eulophus*)

##### Distribution

England

#### Pnigalio pectinicornis

(Linnaeus, 1758)

Ichneumon pectinicornis Linnaeus, 1758
ramicornis (Retzius, 1783, *Ichneumon*)
fusciventris (Nees, 1834, *Elachestus*)
medius (Nees, 1834, *Eulophus*)
coecilius (Walker, 1839, *Eulophus*)
cromus (Walker, 1839, *Eulophus*)
faustitas (Walker, 1839, *Eulophus*)
lucumo (Walker, 1839, *Eulophus*)
mandron (Walker, 1839, *Eulophus*)
mania (Walker, 1839, *Eulophus*)
menyllus (Walker, 1839, *Eulophus*)
fissicornis (Förster, 1841, *Eulophus*)
fuscicornis (Förster, 1841, *Eulophus*)
plumicornis (Förster, 1841, *Eulophus*)
tarandicornis (Förster, 1841, *Eulophus*)
dendricornis (Ratzeburg, 1844, *Eulophus*)
pilicornis (Ratzeburg, 1844, *Eulophus*)
viduus (Ratzeburg, 1844, *Eulophus*)
subcutaneus (Ratzeburg, 1852, *Eulophus*)
habrocerus Förster, 1861, Eulophus)
megalocerus (Förster, 1861, *Eulophus*)

##### Distribution

England

#### Pnigalio phragmitis

(Erdös, 1954)

Eulophus phragmitis Erdös, 1954

##### Distribution

England

#### Pnigalio pristiphorae

Askew, 1965

#### Pnigalio soemius

(Walker, 1839)

Eulophus soemius Walker, 1839
meriones (Walker, 1839, *Eulophus*)
prothenor (Walker, 1839, *Eulophus*)
punctiscuta (Thomson, 1878, *Eulophus*)
nigroaeneus (Erdös, 1954, *Eulophus*)
flavipes (Erdös, 1954, *Eulophus*)

##### Distribution

England

#### Pnigalio ternatus

Askew, 1984

##### Notes

Added by Askew (1984a)

#### Pnigalio tricuspis

(Erdös, 1954)

Eulophus tricuspis Erdös, 1954

##### Distribution

England

##### Notes

Added by Compton and Askew (2007)

#### Pnigalio tyrrhenus

(Walker, 1839)

Eulophus tyrrhenus Walker, 1839

##### Distribution

England

##### Notes

Transferred from nom. dub. under *Eulophus* Gebiola et al. (2015)​

#### Ratzeburgiola

Erdös, 1958

#### Ratzeburgiola incompleta

Bouček, 1971

cristata misident.

#### Stenomesius

Westwood, 1833

EURYSCOTOLINX Girault, 1913
STENELACHISTUS Masi, 1917
NIORO Risbec, 1951

#### Stenomesius rufescens

(Retzius, 1783)

Ichneumon rufescens Retzius, 1783
rufescens (Rossi, 1794, *Ichneumon*) preocc.
maculatus Westwood, 1833
pulchellus Westwood, 1833
acesius (Walker, 1839, *Cirrospilus*)
nemoranae (Rondani, 1870, *Misina*)

#### Sympiesis

Förster, 1856

TELEOGMUS Förster, 1856
SYMPIEZUS Thomson, 1878
ASYMPIESIELLA Girault, 1913
DIAULOMELLA Girault, 1913
NECREMNOMYIA Girault, 1913
OPHELIMINUS Girault, 1913
PSEUDOPHELIMINUS Girault, 1913
SYMPIESONECREMNUS Girault, 1913
DIAULOMORPHELLA Girault, 1915
PARDIAULOMELLA Girault, 1915
PARDIAULOMYIA Girault & Dodd, 1915
PRONECREMNUS Girault & Dodd, 1915
MOROCERAS Erdös, 1954

#### Sympiesis acalle

(Walker, 1848)

Eulophus acalle Walker, 1848
nubeculatus (Ratzeburg, 1848, *Entedon*)
bifasciatus (Thomson, 1878, *Eulophus*)
bimaculatipennis (Girault, 1912, *Astichus*)
bimaculata Crawford, 1913
meteori Girault, 1916

#### Sympiesis dolichogaster

Ashmead, 1888

mikado Ashmead, 1904
nelsonensis (Girault, 1913, *Asympiesiella*)
nelsonensis Girault, 1914 preocc.
india (Girault, 1916, *Asympiesiella*)
nowickii Szelényi, 1941

#### Sympiesis gordius

(Walker, 1839)

Eulophus gordius Walker, 1839
alaparus (Walker, 1839, *Eulophus*)
pisenor (Walker, 1839, *Eulophus*)
cervicornis (Förster, 1841, *Eulophus*)
padellae (Ratzeburg, 1844, *Eulophus*)
bulmerincqii (Ratzeburg, 1848, *Eulophus*)
laevissimus (Ratzeburg, 1848, *Eulophus*)
stramineipes (Thomson, 1878, *Eulophus*)
lexingtonensis Girault, 1917
marylandensis Girault, 1917
miltoni Girault, 1917
rex Girault, 1917

##### Distribution

England, Scotland

#### Sympiesis grahami

Erdös, 1966

#### Sympiesis gregori

Bouček, 1959

linifoliellae Delucchi, 1962

##### Distribution

England, Scotland

#### Sympiesis notata

(Zetterstedt, 1838)

Pteromalus notatus Zetterstedt, 1838
laodochus (Walker, 1839, *Eulophus*)
pronoe (Walker, 1839, *Eulophus*)
sandanis (Walker, 1839, *Eulophus*)
damicornis (Förster, 1841, *Eulophus*)
superior (Förster, 1841, *Eulophus*)
atmopterus (Ratzeburg, 1852, *Entedon*)

#### Sympiesis sericeicornis

(Nees, 1834)

Eulophus sericeicornis Nees, 1834
upupaenellae (Bouché, 1834, *Eulophus*)
docilis (Walker, 1839, *Eulophus*)
eneugamus (Walker, 1839, *Eulophus*)
sithon (Walker, 1839, *Eulophus*)
laticornis (Ratzeburg, 1848, *Entedon*)
punctipleura (Thomson, 1878, *Sympiezus*)
compressicornis (Provancher, 1887, *Coccophagus*)
conicus (Provancher, 1887, *Metacolus*)
nigrifemora Ashmead, 1888
nigripes Ashmead, 1888
massassoit Crawford, 1913

##### Distribution

England

#### Sympiesis viridula

(Thomson, 1878)

Eulophus viridulus Thomson, 1878

##### Distribution

England

#### Sympiesis xanthostoma

(Nees, 1834)

Eulophus xanthostomus Nees, 1834
leodamas (Walker, 1839, *Eulophus*)
orbitalis (Förster, 1856, *Teleogmus*)
szelenyii Györfi, 1941

##### Distribution

England

#### Xanthellum

Erdös & Novicky, 1951

#### Xanthellum transsylvanicum

Erdös, 1951

#### Opheliminae

Ashmead, 1904

##### Notes

Subfamily erected by Burks et al. (2011)

#### Ophelimus

Haliday, 1844

#### Ophelimus maskelli

(Ashmead, 1900)

Pteroptrix maskelli Ashmead, 1900

##### Distribution

England

##### Notes

Added by Tilbury and Jukes (2006): an introduced gall-former on planted *Eucalpytus*, only tentatively identified; see also Badmin (2008)

#### Tetrastichinae

Förster, 1856

##### Notes

Much distribution data from Graham (1987), Graham (1991a)

#### Aceratoneuromyia

Girault, 1917

#### Aceratoneuromyia claridgei

Graham, 1991

##### Distribution

England

##### Notes

Added by Graham (1991a)

#### Aceratoneuromyia granularis

Domenichini, 1967

##### Distribution

England, Wales

#### Aceratoneuromyia indica

(Silvestri, 1910)

Syntomosphyrum indicum Silvestri, 1910
australia Girault, 1917

##### Distribution

England

##### Notes

Graham (1991a) records an English specimen that he supposed represented an accidental introduction.

#### Anaprostocetus

Graham, 1987

#### Anaprostocetus acuminatus

(Ratzeburg, 1848)

Entedon acuminatus Ratzeburg, 1848

##### Distribution

England

#### Aprostocetus

Westwood, 1833

#### Aprostocetus

Westwood, 1833

TETRASTICHUS misident.
TRICHOCERAS Ratzeburg, 1844
GENIOCERUS Ratzeburg, 1848
LONCHENTEDON Ratzeburg, 1852
HYPERTELES Förster, 1856
OXYMORPHA Förster, 1856
MYIOMISA Rondani, 1877
SYNTOMOSPHYRUM Förster, 1878
HADROTHRIX Cameron, 1913

##### Notes

Species of Aprostocetus (Aprostocetus) removed from the British and Irish list as not listed as such by Graham (1987):

[*tompanus* (Erdös, 1954, *Geniocerus*)]

#### Aprostocetus (Aprostocetus) aega

(Walker, 1839)

Cirrospilus aega Walker, 1839

##### Distribution

England

#### Aprostocetus (Aprostocetus) aethiops

(Zetterstedt, 1838)

Entedon aethiops Zetterstedt, 1838
nerio (Walker, 1839, *Cirrospilus*)
Aprostocetus (Aprostocetus) aethiops ?*prosymna* (Walker, 1839, *Cirrospilus*)
vicellius (Walker, 1839, *Cirrospilus*)
teridae (Walker, 1840, *Cirrospilus*)
seminarius (Ratzeburg, 1852, *Entedon*)
spartii (Ratzeburg, 1852, *Entedon*)

##### Distribution

England, Scotland

#### Aprostocetus (Aprostocetus) agrus

(Walker, 1839)

Cirrospilus agrus Walker, 1839
amynus (Walker, 1839, *Cirrospilus*)
conii (Erdös, 1954, *Geniocerus*)
rugosus (Erdös, 1954, *Geniocerus*)

##### Distribution

England, Ireland

#### Aprostocetus (Aprostocetus) alveatus

Graham, 1961

##### Distribution

England

#### Aprostocetus (Aprostocetus) amenon

(Walker, 1839)

Cirrospilus amenon Walker, 1839

##### Distribution

England

#### Aprostocetus (Aprostocetus) annulatus

(Förster, 1861)

Tetrastichus annulatus Förster, 1861

##### Distribution

England

##### Notes

Added by Graham (1987)

#### Aprostocetus (Aprostocetus) anodaphus

(Walker, 1839)

Cirrospilus anodaphus Walker, 1839

##### Distribution

England, Ireland

#### Aprostocetus (Aprostocetus) apama

(Walker, 1839)

Cirrospilus apama Walker, 1839
facialis (Thomson, 1878, *Tetrastichus*)

##### Distribution

England

#### Aprostocetus (Aprostocetus) aquilus

Graham, 1987

##### Distribution

England

##### Notes

Added by Graham (1987)

#### Aprostocetus (Aprostocetus) arathis

(Walker, 1839)

Cirrospilus arathis Walker, 1839

##### Distribution

England

##### Notes

Included as a species inquirenda by Graham (1987)

#### Aprostocetus (Aprostocetus) arenarius

(Erdös, 1954)

Geniocerus arenarius Erdös, 1954

##### Distribution

England

#### Aprostocetus (Aprostocetus) aristaeus

(Walker, 1839)

Cirrospilus aristaeus Walker, 1839
confusus (Förster, 1861, *Tetrastichus*)
seticollis (Thomson, 1878, *Tetrastichus*)

##### Distribution

England, Ireland

#### Aprostocetus (Aprostocetus) arrabonicus

(Erdös, 1954)

Baryscapus arrabonicus Erdös, 1954

##### Distribution

England

##### Notes

Added by Graham (1987)

#### Aprostocetus (Aprostocetus) artemisiae

(Erdös, 1954)

Geniocerus artemisiae Erdös, 1954

##### Distribution

England

##### Notes

Added by Graham (1987)

#### Aprostocetus (Aprostocetus) artemisicola

Graham, 1987

##### Distribution

England

##### Notes

Added by Graham (1987)

#### Aprostocetus (Aprostocetus) aurantiacus

(Ratzeburg, 1852)

Entedon aurantiacus Ratzeburg, 1852
Aprostocetus (Aprostocetus) aurantiacus ?*cyniphidum* (Ratzeburg, 1848, *Geniocerus*)
rosarum (Erdös, 1971, *Tetrastichus*)

##### Distribution

England

##### Notes

Added by Graham (1987)

#### Aprostocetus (Aprostocetus) beroe

(Walker, 1839)

Cirrospilus beroe Walker, 1839

##### Distribution

England

#### Aprostocetus (Aprostocetus) boreus

(Delucchi, 1954)

Tetrastichus boreus Delucchi, 1954

##### Distribution

England

#### Aprostocetus (Aprostocetus) brachycerus

(Thomson, 1878)

Tetrastichus brachycerus Thomson, 1878

##### Distribution

England, Ireland

#### Aprostocetus (Aprostocetus) bruzzonis

(Masi, 1930)

Tetrastichus bruzzonis Masi, 1930

##### Distribution

England

##### Notes

Added by Graham (1987)

#### Aprostocetus (Aprostocetus) calamarius

Graham, 1961

##### Distribution

England, Ireland

##### Notes

Added by Jennings (2003)

#### Aprostocetus (Aprostocetus) catius

(Walker, 1839)

Cirrospilus catius Walker, 1839
Aprostocetus (Aprostocetus) catius ?*vaccus* (Walker, 1839, *Cirrospilus*)

##### Distribution

England, Ireland

#### Aprostocetus (Aprostocetus) caudatus

Westwood, 1833

tristis (Nees, 1834, *Eulophus*)
mutilia (Walker, 1839, *Cirrospilus*)
phalis (Walker, 1839, *Cirrospilus*)
trabea (Walker, 1839, *Cirrospilus*)
crassicauda (Thomson, 1878, *Tetrastichus*)

##### Distribution

England, Ireland

#### Aprostocetus (Aprostocetus) ciliatus

(Nees, 1834)

Eulophus ciliatus Nees, 1834

##### Distribution

England

##### Notes

Added by Graham (1987)

#### Aprostocetus (Aprostocetus) citrinus

(Förster, 1841)

Eulophus citrinus Förster, 1841
varius (Thomson, 1878, *Tetrastichus*)

##### Distribution

England, Ireland

#### Aprostocetus (Aprostocetus) clavicornis

(Zetterstedt, 1838)

Entedon clavicornis Zetterstedt, 1838
euedochus (Walker, 1839, *Cirrospilus*)
lamius (Walker, 1839, *Cirrospilus*)

##### Distribution

England

#### Aprostocetus (Aprostocetus) coccidiphagus

Graham, 1987

##### Distribution

England

##### Notes

Added by Graham (1987)

#### Aprostocetus (Aprostocetus) collega

(Ratzeburg, 1844)

Eulophus collega Ratzeburg, 1844
fageti Graham, 1961

##### Distribution

England

##### Notes

Added by Graham (1987)

#### Aprostocetus (Aprostocetus) constrictus

Graham, 1987

##### Distribution

England, Ireland

##### Notes

Added by Graham (1987)

#### Aprostocetus (Aprostocetus) cultratus

Graham, 1987

##### Distribution

England

##### Notes

Added by Graham (1987)

#### Aprostocetus (Aprostocetus) diversus

(Förster, 1841)

Eulophus diversus Förster, 1841
abydenus (Walker, 1848, *Tetrastichus*)

##### Distribution

England

#### Aprostocetus (Aprostocetus) eleuchia

(Walker, 1839)

Cirrospilus eleuchia Walker, 1839

##### Distribution

England

#### Aprostocetus (Aprostocetus) elongatus

(Förster, 1841)

Eulophus elongatus Förster, 1841
signaticollis (Walker, 1847, *Eulophus*)
macroneurus (Ratzeburg, 1852, *Entedon*)
intermedius (Thomson, 1878, *Tetrastichus*)

##### Distribution

England

#### Aprostocetus (Aprostocetus) emesa

(Walker, 1839)

Cirrospilus emesa Walker, 1839
anteius (Walker, 1839, *Cirrospilus*)
deipyrus (Walker, 1839, *Cirrospilus*)
rabirius (Walker, 1839, *Cirrospilus*)

##### Distribution

England, Ireland

#### Aprostocetus (Aprostocetus) epicharmus

(Walker, 1839)

Cirrospilus epicharmus Walker, 1839
Aprostocetus (Aprostocetus) epicharmus ?*ione* (Walker, 1839, *Cirrospilus*)
Aprostocetus (Aprostocetus) epicharmus ?*rhode* (Walker, 1840, *Cirrospilus*)
vincius (Walker, 1839, *Cirrospilus*)
variegatus (Szelényi, 1941, *Tetrastichus*)

##### Distribution

England, Ireland

#### Aprostocetus (Aprostocetus) eriophyes

(Taylor, 1909)

Tetrastichus eriophyes Taylor, 1909

#### Aprostocetus (Aprostocetus) esherensis

Graham, 1987

##### Distribution

England

##### Notes

Added by Graham (1987)

#### Aprostocetus (Aprostocetus) euagoras

(Walker, 1839)

Cirrospilus euagoras Walker, 1839

##### Distribution

England

#### Aprostocetus (Aprostocetus) eupolis

(Walker, 1839)

Cirrospilus eupolis Walker, 1839

##### Distribution

England

#### Aprostocetus (Aprostocetus) eurytomae

(Nees, 1834)

Eulophus eurytomae Nees, 1834

##### Distribution

England

##### Notes

Added by Askew (1997)

#### Aprostocetus (Aprostocetus) fabicola

(Rondani, 1877)

Entedon fabicola Rondani, 1877
lasiopterinus (Rondani, 1877, *Entedon*)

##### Distribution

England

#### Aprostocetus (Aprostocetus) femoralis

(Sundby, 1957)

Tetrastichus femoralis Sundby, 1957

##### Distribution

England

##### Notes

Added by Graham (1987)

#### Aprostocetus (Aprostocetus) forsteri

(Walker, 1847)

Eulophus forsteri Walker, 1847

##### Notes

Added by Jennings (2009b)

#### Aprostocetus (Aprostocetus) fulvipes

(Förster, 1878)

Syntomosphyrum fulvipes Förster, 1878
astichus (Thomson, 1878, *Tetrastichus*)

##### Distribution

England

#### Aprostocetus (Aprostocetus) gaus

(Walker, 1839)

Cirrospilus gaus Walker, 1839
asopus (Walker, 1839, *Cirrospilus*)
orsedice (Walker, 1839, *Cirrospilus*)
tenerus (Walker, 1839, *Cirrospilus*)
deplanatus (Thomson, 1878, *Tetrastichus*)

##### Distribution

England, Ireland

#### Aprostocetus (Aprostocetus) gratus

(Giraud, 1863)

Tetrastichus gratus Giraud, 1863
thomsonii (Dalla Torre, 1898, *Tetrastichus*)
badulini (Kostjukov, 1977, *Tetrastichus*)

##### Distribution

England, Ireland

#### Aprostocetus (Aprostocetus) humilis

Graham, 1961

##### Distribution

England, Scotland

#### Aprostocetus (Aprostocetus) incrassatus

Graham, 1961

##### Distribution

England

#### Aprostocetus (Aprostocetus) lacaena

(Walker, 1839)

Cirrospilus lacaena Walker, 1839

##### Distribution

England

#### Aprostocetus (Aprostocetus) lachares

(Walker, 1839)

Cirrospilus lachares Walker, 1839

##### Distribution

England

#### Aprostocetus (Aprostocetus) lacunatus

Graham, 1987

##### Distribution

England

##### Notes

Added by Graham (1987)

#### Aprostocetus (Aprostocetus) leucone

(Walker, 1839)

Cirrospilus leucone Walker, 1839
longicaudatus (Förster, 1841, *Eulophus*)
dolichurus (Thomson, 1878, *Tetrastichus*)

##### Distribution

England, Ireland

#### Aprostocetus (Aprostocetus) ligus

(Walker, 1839)

Cirrospilus ligus Walker, 1839
oxathres (Walker, 1839, *Cirrospilus*)

##### Distribution

England

#### Aprostocetus (Aprostocetus) longicauda

(Thomson, 1878)

Tetrastichus longicauda Thomson, 1878

##### Distribution

England

#### Aprostocetus (Aprostocetus) longiscapus

(Thomson, 1878)

Tetrastichus longiscapus Thomson, 1878

##### Distribution

England

##### Notes

Askew coll., det. Askew, added here

#### Aprostocetus (Aprostocetus) luteus

(Ratzeburg, 1848)

Entedon luteus Ratzeburg, 1848

##### Distribution

England

#### Aprostocetus (Aprostocetus) lycidas

(Walker, 1839)

Cirrospilus lycidas Walker, 1839

##### Distribution

England

#### Aprostocetus (Aprostocetus) lysippe

(Walker, 1839)

Cirrospilus lysippe Walker, 1839
achaemenes (Walker, 1839, *Cirrospilus*)

##### Distribution

England

#### Aprostocetus (Aprostocetus) menius

(Walker, 1839)

Cirrospilus menius Walker, 1839

##### Distribution

England

#### Aprostocetus (Aprostocetus) meroe

Graham, 1987

##### Distribution

England

##### Notes

Added by Graham (1987)

#### Aprostocetus (Aprostocetus) metra

(Walker, 1839)

Cirrospilus metra Walker, 1839

##### Distribution

England

#### Aprostocetus (Aprostocetus) micantulus

(Thomson, 1878)

Tetrastichus micantulus Thomson, 1878

##### Distribution

England

##### Notes

Added by Graham (1987)

#### Aprostocetus (Aprostocetus) myrsus

(Walker, 1839)

Cirrospilus myrsus Walker, 1839

##### Distribution

England

#### Aprostocetus (Aprostocetus) neglectus

(Domenichini, 1957)

Tetrastichus neglectus Domenichini, 1957

##### Distribution

England

##### Notes

Added by Mabbott and Mabbott (2003)

#### Aprostocetus (Aprostocetus) novatus

(Walker, 1839)

Cirrospilus novatus Walker, 1839

##### Distribution

England

#### Aprostocetus (Aprostocetus) nymphis

(Walker, 1839)

Cirrospilus nymphis Walker, 1839

##### Distribution

England

#### Aprostocetus (Aprostocetus) obliquus

Graham, 1987

##### Distribution

England

##### Notes

Added by Graham (1987)

#### Aprostocetus (Aprostocetus) orithyia

(Walker, 1839)

Cirrospilus orithyia Walker, 1839
arundinis (Giraud, 1863, *Tetrastichus*)

##### Distribution

England, Ireland

#### Aprostocetus (Aprostocetus) oropus

(Walker, 1839)

Cirrospilus oropus Walker, 1839

##### Distribution

England

#### Aprostocetus (Aprostocetus) pachyneuros

(Ratzeburg, 1844)

Eulophus pachyneuros Ratzeburg, 1844

##### Distribution

England

##### Notes

Added by Graham (1987)

#### Aprostocetus (Aprostocetus) pallipes

(Dalman, 1820)

Entedon pallipes Dalman, 1820
faucula (Walker, 1839, *Cirrospilus*)
orodes (Walker, 1839, *Cirrospilus*)
sucro (Walker, 1839, *Cirrospilus*)
Aprostocetus (Aprostocetus) pallipes ?*voranus* (Walker, 1839, *Cirrospilus*)
pallidipes (Dalla Torre, 1898, *Entedon*)

##### Distribution

England, Scotland

#### Aprostocetus (Aprostocetus) palustris

Graham, 1987

##### Distribution

England

##### Notes

Added by Graham (1987)

#### Aprostocetus (Aprostocetus) pausiris

(Walker, 1839)

Cirrospilus pausiris Walker, 1839
anticlea (Walker, 1839, *Cirrospilus*)
cyrrhus (Walker, 1839, *Cirrospilus*)

##### Distribution

England, Ireland

#### Aprostocetus (Aprostocetus) perone

Graham, 1987

##### Distribution

England

##### Notes

Added by Graham (1987)

#### Aprostocetus (Aprostocetus) phineus

(Walker, 1839)

Cirrospilus phineus Walker, 1839

##### Distribution

England

#### Aprostocetus (Aprostocetus) phloeophthori

Graham, 1983

##### Distribution

England

##### Notes

Added by Graham (1987)

#### Aprostocetus (Aprostocetus) phragmiticola

Graham, 1987

##### Distribution

England

##### Notes

Added by Graham (1987)

#### Aprostocetus (Aprostocetus) phragmitinus

(Erdös, 1954)

Geniocerus phragmitinus Erdös, 1954

##### Distribution

England

##### Notes

Added by Graham (1987)

#### Aprostocetus (Aprostocetus) planiusculus

(Thomson, 1878)

Tetrastichus planiusculus Thomson, 1878

##### Distribution

England

#### Aprostocetus (Aprostocetus) ptarmicae

Graham, 1987

##### Distribution

England

##### Notes

Added by Graham (1987)

#### Aprostocetus (Aprostocetus) pygmaeus

(Zetterstedt, 1838)

Entedon pygmaeus Zetterstedt, 1838
conon (Walker, 1839, *Cirrospilus*)
deioces (Walker, 1839, *Cirrospilus*)
Aprostocetus (Aprostocetus) pygmaeus ?*plangon* (Walker, 1839, *Cirrospilus*)
sandace (Walker, 1839, *Cirrospilus*)
xixuthrus (Walker, 1839, *Cirrospilus*)
zenocia (Walker, 1839, *Cirrospilus*)
triarius (Walker, 1848, *Tetrastichus*)
obscuripes (Thomson, 1878, *Tetrastichus*)

##### Distribution

England, Ireland

#### Aprostocetus (Aprostocetus) rhacius

(Walker, 1839)

Cirrospilus rhacius Walker, 1839
dotus misident.
Aprostocetus (Aprostocetus) rhacius ?*mazaeus* (Walker, 1839, *Cirrospilus*)

##### Distribution

England

#### Aprostocetus (Aprostocetus) rhipheus

(Walker, 1839)

Cirrospilus rhipheus Walker, 1839
anyta (Walker, 1839, *Cirrospilus*)
Aprostocetus (Aprostocetus) rhipheus ?*eratus* (Walker, 1839, *Cirrospilus*)

##### Distribution

England

#### Aprostocetus (Aprostocetus) roesellae

(Nees, 1834)

Eulophus roesellae Nees, 1834
deplanatus Walker, 1874 (*Tetrastichus)*

##### Distribution

England

#### Aprostocetus (Aprostocetus) rubi

Graham, 1987

##### Notes

Added by Jennings (2005a)

#### Aprostocetus (Aprostocetus) rufescens

Graham, 1987

##### Distribution

England

##### Notes

Added by Graham (1987)

#### Aprostocetus (Aprostocetus) rufiscapus

Graham, 1987

##### Distribution

England

##### Notes

Added by Graham (1987)

#### Aprostocetus (Aprostocetus) rumicis

Graham, 1987

##### Distribution

England

##### Notes

Added by Graham (1987)

#### Aprostocetus (Aprostocetus) salictorum

Graham, 1987

##### Distribution

Scotland

##### Notes

Jennings coll., det. Askew, added here

#### Aprostocetus (Aprostocetus) scoticus

Graham, 1987

##### Distribution

Scotland

##### Notes

Added by Graham (1987)

#### Aprostocetus (Aprostocetus) serratularum

Graham, 1987

##### Distribution

England

##### Notes

Added by Graham (1987)

#### Aprostocetus (Aprostocetus) stenus

Graham, 1987

##### Distribution

England

##### Notes

Added by Graham (1987)

#### Aprostocetus (Aprostocetus) stigmaticalis

Graham, 1987

##### Distribution

England

##### Notes

Added by Graham (1987)

#### Aprostocetus (Aprostocetus) strobilanae

(Ratzeburg, 1844)

Eulophus strobilanae Ratzeburg, 1844
erythrophthalmus (Ratzeburg, 1844, *Trichoceras*)

##### Distribution

England

#### Aprostocetus (Aprostocetus) subanellatus

Graham, 1961

##### Distribution

England, Ireland

##### Notes

Added by Graham (1987)

#### Aprostocetus (Aprostocetus) taxi

Graham, 1987

##### Distribution

England

##### Notes

Added by Graham (1987)

#### Aprostocetus (Aprostocetus) tanaceticola

Graham, 1987

##### Notes

Added by Jennings (2009d)

#### Aprostocetus (Aprostocetus) tenuiradialis

Graham, 1987

##### Distribution

England

##### Notes

Added by Graham (1987)

#### Aprostocetus (Aprostocetus) terebrans

Erdös, 1954

##### Distribution

England, Ireland

#### Aprostocetus (Aprostocetus) tilicola

Graham, 1987

##### Distribution

England

##### Notes

Added by Graham (1987)

#### Aprostocetus (Aprostocetus) totis

(Walker, 1839)

Cirrospilus totis Walker, 1839

##### Distribution

England

##### Notes

Included as a species inquirenda by Graham (1987)

#### Aprostocetus (Aprostocetus) trjapitzini

(Kostjukov, 1976)

Tetrastichus trjapitzini Kostjukov, 1976

##### Distribution

England

##### Notes

Added by Graham (1987)

#### Aprostocetus (Aprostocetus) tymber

(Walker, 1839)

Cirrospilus tymber Walker, 1839

##### Distribution

England, Ireland

#### Aprostocetus (Aprostocetus) veronicae

Graham, 1987

##### Distribution

England

##### Notes

Added by Graham (1987)

#### Aprostocetus (Aprostocetus) verticalis

Graham, 1987

##### Distribution

England

##### Notes

Added by Graham (1987)

#### Aprostocetus (Aprostocetus) verutus

Graham, 1961

##### Distribution

England

#### Aprostocetus (Aprostocetus) viridinitens

Graham, 1987

##### Distribution

England

##### Notes

Added by Graham (1987)

#### Aprostocetus (Aprostocetus) xeuxes

(Walker, 1839)

Cirrospilus xeuxes Walker, 1839

##### Distribution

England

##### Notes

Included as a species inquirenda by Graham (1987)

#### Aprostocetus (Aprostocetus) zoilus

(Walker, 1839)

Cirrospilus zoilus Walker, 1839

##### Distribution

England, Ireland

#### Aprostocetus (Aprostocetus) zosimus

(Walker, 1839)

Cirrospilus zosimus Walker, 1839
abantidas (Walker, 1839, *Cirrospilus*)
athyrte (Walker, 1839, *Cirrospilus*)
bunus (Walker, 1839, *Cirrospilus*)
chares (Walker, 1839, *Cirrospilus*)
hypsistus (Walker, 1839, *Cirrospilus*)
molo (Walker, 1839, *Cirrospilus*)
Aprostocetus (Aprostocetus) zosimus ?*paralus* (Walker, 1839, *Cirrospilus*)
simo (Walker, 1839, *Cirrospilus*)
charoba (Walker, 1840, *Cirrospilus*)
zopyrus (Walker, 1840, *Cirrospilus*)
flavimanus (Thomson, 1878, *Tetrastichus*)
punctiscuta (Thomson, 1878, *Tetrastichus*)
carinatus (Forbes, 1885, *Tetrastichus*)
rileyi (Lindeman, 1887, *Tetrastichus*)
tenuis (Erdös, 1954, *Geniocerus*)

##### Distribution

England, Ireland

#### Chrysotetrastichus

Kostjukov, 1977

#### Aprostocetus (Chrysotetrastichus) celtidis

(Erdös, 1954)

Geniocerus celtidis Erdös, 1954

##### Distribution

England, Ireland

#### Aprostocetus (Chrysotetrastichus) distichus

Graham, 1961

##### Distribution

England

##### Notes

Omitted by Bouček and Graham (1978)

#### Aprostocetus (Chrysotetrastichus) oreophilus

(Förster, 1861)

Tetrastichus oreophilus Förster, 1861

##### Distribution

England

#### Aprostocetus (Chrysotetrastichus) suevius

(Walker, 1839)

Cirrospilus suevius Walker, 1839
salicis Erdös, 1961

##### Distribution

England, Ireland

#### Coriophagus

Graham,1987

#### Aprostocetus (Coriophagus) eurytus

(Walker, 1839)

Cirrospilus eurytus Walker, 1839
elegans (Erdös, 1951, *Geniocerus*)

##### Distribution

England

#### Ootetrastichus

Perkins, 1906

Ootetrastichus ?*NEOMPHALOIDOMYIA* Girault, 1917
ANELLARIA Bakkendorf, 1934
GYROLACHNUS Erdös, 1954
PACHYSCAPUS Erdös, 1954
TEREBRATELLA Shafee & Rizvi, 1985

#### Aprostocetus (Ootetrastichus) crino

(Walker, 1838)

Cirrospilus crino Walker, 1838
dispar (Silvestri, 1920, *Tetrastichus*)
oecanthivorus (Gahan, 1932, *Tetrastichus*)
dubius (Bakkendorf, 1955, *Tetrastichus*)

##### Distribution

England, Ireland

#### Aprostocetus (Ootetrastichus) longulus

(Erdös, 1954)

Gyrolachnus longulus Erdös, 1954

##### Distribution

Ireland

##### Notes

Added by Graham (1987)

#### Aprostocetus (Ootetrastichus) mandanis

(Walker, 1839)

Cirrospilus mandanis Walker, 1839
conomeli (Bakkendorf, 1934, *Anellaria*)

##### Distribution

England

#### Aprostocetus (Ootetrastichus) mycerinus

(Walker, 1839)

Cirrospilus mycerinus Walker, 1839
quadriannulatus Kurdjumov, 1913
acuminatellus (Erdös, 1969, *Tetrastichus*)

##### Distribution

England, Ireland

#### Aprostocetus (Ootetrastichus) rufus

(Bakkendorf, 1953)

Tetrastichus rufus Bakkendorf, 1953
cupratus Erdös, 1958

##### Distribution

England

#### Baryscapus

Förster, 1856

EUTETRASTICHUS Kostjukov, 1977
TETRASTICHOPSIS Girault, 1916
THRIPOSOMA Crawford, 1913

##### Notes

Species of *Baryscapus* removed from the British and Irish list:

[*agrilorum* (Ratzeburg, 1844, *Eulophus*)] - listed as British by Bouček and Graham (1978) but apparently in error

#### Baryscapus adalia

(Walker, 1839)

Cirrospilus adalia Walker, 1839
crassinervis (Thomson, 1878, *Tetrastichus*)

##### Distribution

England

#### Baryscapus cirsiicola

Graham, 1991

##### Distribution

England

##### Notes

Added by Graham (1991a)

#### Baryscapus conwentziae

(Ferrière, 1959)

Tetrastichus conwentziae Ferrière, 1959

##### Distribution

England

##### Notes

Added by Graham (1991)

#### Baryscapus daira

(Walker, 1839)

Cirrospilus daira Walker, 1839
canadensis Ashmead, 1888
cirsii Kurdjumov, 1913

##### Distribution

England

#### Baryscapus diaphantus

(Walker, 1839)

Cirrospilus diaphantus Walker, 1839
terminalis (Thomson, 1878, *Tetrastichus*)

##### Distribution

England

#### Baryscapus endemus

(Walker, 1839)

Cirrospilus endemus Walker, 1839
decisus (Walker, 1863, *Tetrastichus*)
tibialis (Kurdjumov, 1913, *Geniocerus*)
encyrti (Ferrière, 1926, *Tetrastichus*)
orchestidis (Bukovskii, 1938, *Tetrastichus*)
cioni (Erdös, 1971, *Tetrastichus*)
femoralis (Erdös, 1971, *Tetrastichus*)

##### Distribution

England, Ireland

#### Baryscapus evonymellae

(Bouché, 1834)

Eulophus evonymellae Bouché, 1834
cribrellae (Rondani, 1877, *Entedon*)

##### Distribution

England

#### Baryscapus fossarum

Graham, 1991

##### Distribution

England

##### Notes

Added by Graham (1991)

#### Baryscapus galactopus

(Ratzeburg, 1844)

Eulophus galactopus Ratzeburg, 1844
vinulae (Ratzeburg, 1844, *Eulophus*)
lissonotus (Möller, 1886, *Tetrastichus*)

##### Distribution

England, Ireland

#### Baryscapus gradwelli

Graham, 1991

##### Distribution

England

##### Notes

Added by Graham (1991)

#### Baryscapus hylesini

Graham, 1991

##### Distribution

England

##### Notes

Added by Graham (1991)

#### Baryscapus nigroviolaceus

(Nees, 1834)

Eulophus nigroviolaceus Nees, 1834
amethystinus (Ratzeburg, 1848, *Entedon*)
antispilae (Rondani, 1877, *Entedon*)

##### Distribution

England

##### Notes

Added by Graham (1991)

#### Baryscapus pallidae

Graham, 1991

##### Distribution

England

##### Notes

Added by Graham (1991)

#### Baryscapus pilicornis

Graham, 1991

##### Distribution

England

##### Notes

Added by Graham (1991)

#### Baryscapus pospelovi

(Kurdjumov, 1912)

Tetrastichus pospelovi Kurdjumov, 1912

##### Distribution

England

##### Notes

Added by Graham (1991)

#### Baryscapus spartifoliellae

Graham, 1991

##### Distribution

England

##### Notes

Added by Graham (1991)

#### Baryscapus spenceri

Graham, 1991

##### Distribution

England

##### Notes

Added by Graham (1991)

#### Baryscapus szöcsi

(Erdös, 1958)

Geniocerus Erdös, 1958

##### Distribution

England

##### Notes

Added by Graham (1991)

#### Baryscapus tineivorus

(Ferrière, 1941)

Tetrastichus tineivorus Ferrière, 1941
carpatus (Burks, 1943, *Tetrastichus*)

##### Distribution

England

##### Notes

Added by Graham (1991)

#### Baryscapus turionum

(Hartig, 1838)

Eulophus turionum Hartig, 1838

##### Distribution

England

#### Chaenotetrastichus

Graham, 1987

#### Chaenotetrastichus semiflavus

(Girault, 1917)

Parachrysocharis semiflava Girault, 1917

##### Notes

Askew coll., added here

#### Crataepus

Förster, 1878

#### Crataepus marbis

(Walker, 1839)

Cirrospilus marbis Walker, 1839
aquisgranensis Förster, 1878
fletcherii Ashmead, 1892

##### Distribution

England

#### Holcotetrastichus

Graham, 1987

#### Holcotetrastichus rhosaces

(Walker, 1839)

Cirrospilus rhosaces Walker, 1839
racilla (Walker, 1839, *Cirrospilus*)

##### Distribution

England, Ireland

#### Kocourekia

Bouček, 1966

#### Kocourekia debilis

(Ratzeburg, 1852)

Entedon debilis Ratzeburg, 1852
hirtula Bouček, 1966

##### Distribution

England

##### Notes

Added by Askew (1997)

#### Melittobia

Westwood, 1848

ANTHOPHORABIA Newport, 1849
PHILOPISON Cameron, 1908
SPHECOPHAGUS Brèthes, 1910
SPHECOPHILUS Brèthes, 1910

#### Melittobia acasta

(Walker, 1839)

Cirrospilus acasta Walker, 1839
audouinii Westwood, 1848
retusa (Newport, 1849, *Anthophorabia*)
fasciata (Newport, 1852, *Anthophorabia*)
osmiae Thomson, 1878
strandi Wolff & Krausse, 1921
melittobius (Thomson, 1878, *Tetrastichus*)

##### Distribution

England

#### Minotetrastichus

Kostjukov, 1977

#### Minotetrastichus frontalis

(Nees, 1834)

Eulophus frontalis Nees, 1834
ecus (Walker, 1839, *Cirrospilus*)
cyclogaster (Ratzeburg, 1844, *Eulophus*)
xanthops (Ratzeburg, 1844, *Eulophus*)
rivillellae (Rondani, 1877, *Entedon*)
budensis (Erdös, 1954, *Geniocerus*)
cimbicis (Kostjukov, 1976, *Tetrastichus*)

##### Distribution

England, Ireland

#### Minotetrastichus loxotoma

(Graham, 1961)

Aprostocetus loxotoma Graham, 1961

##### Distribution

England

#### Minotetrastichus platanellus

(Mercet, 1922)

Tetrastichodes platanellus Mercet, 1922
populi (Erdös, 1958, *Tetrastichodes*)
populifoliella (Erdös, 1969, *Tetrastichus*)

##### Distribution

England

##### Notes

Added by Agassiz and LaSalle (1996)

#### Neotrichoporoides

Girault, 1913

APROSTOCEROLOIDES Girault, 1913
TETRASTICHOMORPHA Girault, 1913
TRICHAPOROIDELLA Girault, 1913
EPIQUADRASTICHUS Girault, 1915
PARAPROSTOCETUS Girault, 1915
BURKSIA Fullaway, 1955

#### Neotrichoporoides gordensis

Graham, 1987

##### Notes

Added by Graham (1987)

#### Oomyzus

Rondani, 1870

#### Oomyzus anomalus

Graham, 1991

##### Distribution

England

##### Notes

Added by Graham (1991)

#### Oomyzus galerucivorus

(Hedqvist, 1959)

Tetrastichus galerucivorus Hedqvist, 1959

#### Oomyzus incertus

(Ratzeburg, 1844)

Eulophus incertus Ratzeburg, 1844
matranus (Erdös, 1954, *Baryscapus*)
fumatus (Erdös, 1954, *Tetrastichus*)
erdoesi (Domenichini, 1965, *Tetrastichus*)
pannonicus (Erdös, 1969, *Tetrastichus*)

##### Distribution

England, Ireland

##### Notes

Added by Graham (1991)

#### Oomyzus scaposus

(Thomson, 1878)

Tetrastichus scaposus Thomson, 1878
coccinellae (Kurdjumov, 1912, *Tetrastichus*)
taprobanes (Waterston, 1915, *Syntomosphyrum*)
melanis (Burks, 1943, *Tetrastichus*)
sexmaculatus (Chandy Kurian, 1953, *Tetrastichus*)

##### Distribution

England

##### Notes

Added by Graham (1991)

#### Oomyzus tanaceti

(Graham, 1985)

Tetrastichus tanaceti Graham, 1985

##### Distribution

England

##### Notes

Added by Graham (1985)

#### Peckelachertus

Yoshimoto, 1970

#### Peckelachertus anglicanus

Graham, 1977

##### Distribution

England

#### Phymastichus

LaSalle, 1990

#### Phymastichus coffea

LaSalle, 1990

#### Pronotalia

Gradwell, 1957

CRATAEPIELLA Domenichini, 1958

#### Pronotalia trypetae

Gradwell, 1957

##### Distribution

England

#### Quadrastichus

Girault, 1913

CECIDOTETRASTICHUS Kostjukov, 1977

#### Quadrastichus anysis

(Walker, 1839)

Cirrospilus anysis Walker, 1839

##### Distribution

England

#### Quadrastichus artemisiphilus

Graham, 1991

##### Distribution

England

##### Notes

Added by Graham (1991)

#### Quadrastichus brevinervis

(Zetterstedt, 1838)

Entedon brevinervis Zetterstedt, 1838
subdepressus (Thomson, 1878, *Tetrastichus*)

##### Distribution

England

##### Notes

Added by Graham (1991)

#### Quadrastichus centor

(Graham, 1961)

Aprostocetus centor Graham, 1961

##### Distribution

England, Ireland

#### Quadrastichus citrinus

(Thomson, 1878)

Tetrastichus citrinus Thomson, 1878
citrinellus (Graham, 1961, *Aprostocetus*)

##### Distribution

England, Scotland, Wales

#### Quadrastichus fungicola

Graham, 1991

##### Distribution

England

##### Notes

Added by Graham (1991)

#### Quadrastichus lasiocerus

(Graham, 1961)

Aprostocetus lasiocera Graham, 1961

##### Distribution

England, Ireland

#### Quadrastichus malhamensis

(Graham, 1961)

Aprostocetus malhamensis Graham, 1961

##### Distribution

England

#### Quadrastichus pedicellaris

(Thomson, 1878)

Tetrastichus pedicellaris Thomson, 1878
flavicornis (Erdös, 1954, *Tetrastichus*)

##### Distribution

England, Ireland

#### Quadrastichus perissiae

(Janata, 1912)

Tetrastichus perissiae Janata, 1912

##### Distribution

England

##### Notes

Added by Graham (1991a)

#### Quadrastichus praecox

(Graham, 1961)

Aprostocetus praecox Graham, 1961

##### Distribution

England

#### Quadrastichus pteridis

Graham, 1991

##### Distribution

Ireland

##### Notes

Added by Graham (1991a)

#### Quadrastichus sajoi

(Szelényi, 1941)

Myiomisa sajoi Szelényi, 1941
scabricollis (Graham, 1961, *Aprostocetus*)

##### Distribution

England

#### Quadrastichus stenocranus

Graham, 1991

##### Distribution

England

##### Notes

Added by Graham (1991)

#### Quadrastichus thysanotus

(Förster, 1861)

Tetrastichus thysanotus Förster, 1861
pumilio (Graham, 1961, *Aprostocetus*)

##### Distribution

England, Ireland

#### Quadrastichus vacuna

(Walker, 1839)

Cirrospilus vacuna Walker, 1839
alcithoe (Walker, 1839, *Cirrospilus*)
numeria (Walker, 1839, *Cirrospilus*)
quercens (Walker, 1839, *Cirrospilus*)
rhoesus (Walker, 1839, *Cirrospilus*)
sotades (Walker, 1839, *Cirrospilus*)
brunchus (Walker, 1840, *Cirrospilus*)
compressiventris (Thomson, 1878, *Tetrastichus*)
migrator (Förster, 1861, *Tetrastichus*)
penetrans (Förster, 1861, *Tetrastichus*)

##### Distribution

England, Scotland, Wales, Ireland

#### Quadrastichus ventricosus

(Graham, 1961)

Aprostocetus ventricosus Graham, 1961

##### Distribution

England

#### Quadrastichus xanthosoma

(Graham, 1974)

Tetrastichus xanthosoma Graham, 1974

##### Distribution

England, Scotland

#### Sigmophora

Rondani, 1867

LOPODYTES Rondani, 1867
EULOPHOTETRASTICHUS Girault, 1913
EUPLECTROTETRASTICHUS Girault, 1915
LOPODYTISCUS Ghesquière, 1946

#### Sigmophora brevicornis

(Panzer, 1804)

Cynips brevicornis Panzer, 1804
verbasci (Dufour, 1837, *Eulophus*)
armaeus (Walker, 1839, *Cirrospilus*)
zeuxo (Walker, 1839, *Cirrospilus*)
setiseries (Förster, 1841, *Eulophus*)
asphondyliae (Rondani, 1867, *Lopodytes*)
prunicola (Rondani, 1867, *Lopodytes*)
scrophulariella Rondani, 1867
tricolor (Ashmead, 1904, *Tetrastichus*)
isaaci (Rohwer, 1921, *Tetrastichus*)
sayatamabae (Ishii, 1950, *Tetrastichus*)

##### Distribution

England, Ireland

#### Tamarixia

Mercet, 1924

#### Tamarixia actis

(Walker, 1839)

Pteroptrix actis Walker, 1839
callunae (Erdös, 1969, *Tetrastichus*)

##### Distribution

England, Ireland

#### Tamarixia leptothrix

Graham, 1991

##### Distribution

England, Ireland

##### Notes

Added by Graham (1991)

#### Tamarixia monesus

(Walker, 1839)

Cirrospilus monesus Walker, 1839
pallicornis (Thomson, 1878, *Tetrastichus*)
pallidicornis (Dalla Torre, 1898, *Tetrastichus*)

##### Distribution

England

#### Tamarixia pronomus

(Walker, 1839)

Cirrospilus pronomus Walker, 1839
pamyles (Walker, 1839, *Cirrospilus*)
obscuratus (André‚ 1878, *Tetrastichus*)

##### Distribution

England

#### Tamarixia pubescens

(Nees, 1834)

Eulophus pubescens Nees, 1834

##### Distribution

England

#### Tamarixia tremblayi

(Domenichini, 1965)

Tetrastichus tremblayi Domenichini, 1965

##### Distribution

England

##### Notes

Added by Graham (1991)

#### Tamarixia upis

(Walker, 1839)

Cirrospilus upis Walker, 1839
orsillus (Walker, 1839, *Cirrospilus*)
bermius (Walker, 1848, *Tetrastichus*)

##### Distribution

England

#### Tetrastichus

Haliday, 1844

ENNETOMA Dahlbom, 1857
SOLENODERUS Motschulsky, 1863
LYGELLUS Giard, 1896
NEOTETRASTICHUS Perkins, 1912
CERATONEURONOMYIA Girault, 1913
PSEUDOMPHALOIDES Girault, 1915
REDINIA Girault, 1936
NEPARAPROSTOCETUS Mani, 1939

#### Tetrastichus acutiusculus

Graham, 1991

##### Distribution

England

#### Tetrastichus atratulus

(Nees, 1834)

Eulophus atratulus Nees, 1834
puncticoxae Kurdjumov, 1913

##### Distribution

England

##### Notes

Added by Graham (1991)

#### Tetrastichus axia

Walker, 1848

#### Tetrastichus brachyopae

Graham, 1991

##### Distribution

England

##### Notes

Askew coll., det. Askew, added here

#### Tetrastichus brevicalcar

Graham, 1991

##### Distribution

England

##### Notes

Added by Graham (1991)

#### Tetrastichus clito

(Walker, 1840)

Cirrospilus clito Walker, 1840
cassidae (Dufour, 1846, *Eulophus*)
cassidarum (Ratzeburg, 1852, *Entedon*)

##### Distribution

England

#### Tetrastichus coelarchus

Graham, 1991

##### Distribution

England, Ireland

##### Notes

Added by Graham (1991)

#### Tetrastichus coeruleus

(Nees, 1834)

Eulophus coeruleus Nees, 1834
asparagi Crawford, 1909

##### Distribution

England

#### Tetrastichus dasyops

Graham, 1991

##### Distribution

England

##### Notes

Added by Graham (1991)

#### Tetrastichus decrescens

Graham, 1991

##### Distribution

England

##### Notes

Added by Graham (1991)

#### Tetrastichus halidayi

(Graham, 1961)

Aprostocetus halidayi Graham, 1961

##### Distribution

England, Scotland, Ireland

#### Tetrastichus helviscapus

Graham, 1991

##### Distribution

England

##### Notes

Added by Graham (1991)

#### Tetrastichus hylotomarum

(Bouché, 1834)

Eulophus hylotomarum Bouché, 1834

##### Distribution

England

#### Tetrastichus ilithyia

(Walker, 1839)

Cirrospilus ilithyia Walker, 1839

##### Distribution

Scotland, Ireland

#### Tetrastichus inaequalis

Graham, 1991

##### Distribution

England

##### Notes

Added by Graham (1991)

#### Tetrastichus inunctus

(Nees, 1834)

Eulophus inunctus Nees, 1834
oleinus (Ratzeburg, 1848, *Entedon*)

##### Distribution

England

##### Notes

This name was overlooked by Graham (1987) and is therefore of uncertain generic placement; in the BMNH collections, *inunctus* has been included under *Aprostocetus*. English specimens were listed by Thompson (1955) but omitted by Bouček and Graham (1978). There are specimens in BMNH det. Bouček and Ferrière.

#### Tetrastichus julis

(Walker, 1839)

Cirrospilus julis Walker, 1839
maderae Walker, 1872

##### Distribution

England

#### Tetrastichus legionarius

Giraud, 1863

##### Distribution

England

##### Notes

Added by Plant (1997)

#### Tetrastichus leocrates

(Walker, 1839)

Cirrospilus leocrates Walker, 1839

##### Distribution

England

#### Tetrastichus lyridice

(Walker, 1839)

Cirrospilus lyridice Walker, 1839

##### Distribution

England

#### Tetrastichus macrops

(Graham, 1961)

Aprostocetus macrops Graham, 1961

##### Distribution

England

#### Tetrastichus miser

(Nees, 1834)

Eulophus miser Nees, 1834
attalus (Walker, 1839, *Cirrospilus*)
medianus (Ratzeburg, 1848, *Entedon*)

##### Distribution

England, Ireland

#### Tetrastichus murcia

(Walker, 1839)

Cirrospilus murcia Walker, 1839
trichops Thomson, 1878

##### Distribution

England

#### Tetrastichus pachycerus

Graham, 1991

##### Distribution

England

##### Notes

Added by Graham (1991)

#### Tetrastichus paululus

Graham, 1991

##### Distribution

England, Wales

##### Notes

Added by Graham (1991)

#### Tetrastichus pilemostomae

Graham, 1991

##### Distribution

England

##### Notes

Added by Graham (1991)

#### Tetrastichus setifer

Thomson, 1878

##### Distribution

England

##### Notes

Added by Salisbury (2003)

#### Tetrastichus sinope

(Walker, 1839)

Cirrospilus sinope Walker, 1839
agathocles (Walker, 1839, *Cirrospilus*)
hippis (Walker, 1839, *Cirrospilus*)
rapo (Walker, 1839, *Cirrospilus*)

##### Distribution

England, Ireland

#### Tetrastichus tachos

(Walker, 1839)

Cirrospilus tachos Walker, 1839

##### Distribution

England

#### Tetrastichus telon

(Graham, 1961)

Aprostocetus telon Graham, 1961

##### Distribution

England

#### Tetrastichus temporalis

(Graham, 1961)

Aprostocetus temporalis Graham, 1961

##### Distribution

England

#### Tetrastichus tyrtaeus

(Walker, 1839)

Cirrospilus tyrtaeus Walker, 1839

##### Distribution

England

##### Notes

Included as a species inquirenda by Graham (1987)

#### Tetrastichus ulmi

Erdös, 1954

##### Distribution

England

### Family Eupelmidae Walker, 1833

#### Calosotinae

Curtis, 1836

#### Calosota

Curtis, 1836

CALOSOTER Walker, 1837
METACALOSOTER Masi, 1917
HYLEPHILA Masi, 1927 preocc.
HYLEPHILISCA Ghesquière, 1946
MINAIA Pagliano & Scaramozzino, 1990

#### Calosota acron

(Walker, 1848)

Eupelmus acron Walker, 1848
contractus (Walker, 1872, *Trigonoderus*)
anguinalis Ruschka, 1921

##### Distribution

England

#### Calosota aestivalis

Curtis, 1836

vernalis (Walker, 1837, *Calosoter*) preocc.
fumipennis Bolivar & Pieltain, 1923

##### Distribution

England, Ireland

#### Calosota vernalis

Curtis, 1836

aestivalis (Walker, 1837, *Calosoter*) preocc.

##### Distribution

England

#### Eusandalum

Ratzeburg, 1852

STENOCERA Curtis, 1836 preocc.
POLYMORIA Förster, 1856
RATZEBURGIA Förster, 1856
STENOCEROIDES Dalla Torre, 1897
MESEUSANDALUM Girault, 1915
POLYMORIOIDES Masi, 1941
NOTOSANDALUM De Santis, 1968
EXOSANDALUM Bouček, 1988

#### Eusandalum walkeri

(Curtis, 1836)

Stenocera walkeri Curtis, 1836

##### Distribution

England

#### Eupelminae

Walker, 1833

#### Anastatus

Motschulsky, 1859

CACOTROPIA Motschulsky, 1863
ANTIGASTER Walsh & Riley, 1869
MISOCHORIS Rondani, 1877
MISOCORIS Rondani, 1877
SOLINDENIA Cameron, 1883
PARAGUAYA Girault, 1911
PARASOLINDENIA Girault, 1913
PAROODERELLA Girault, 1913
PSEUDANASTATUS Girault, 1913
PSEUDOODERELLA Brèthes, 1922
CERYCIUM Erdös, 1946
PARAVIGNALIA Risbec, 1951
VIGNALIA Risbec, 1951
PROANASTATUS De Santis, 1952
DESCAMPSIA De Santis, 1952
ANASTATIMORPHA Erdös, 1957
CLADANASTATUS Bouček, 1979

#### Anastatus catalonicus

Bolivar & Pieltain, 1935

##### Distribution

England

##### Notes

**Figure 6.**
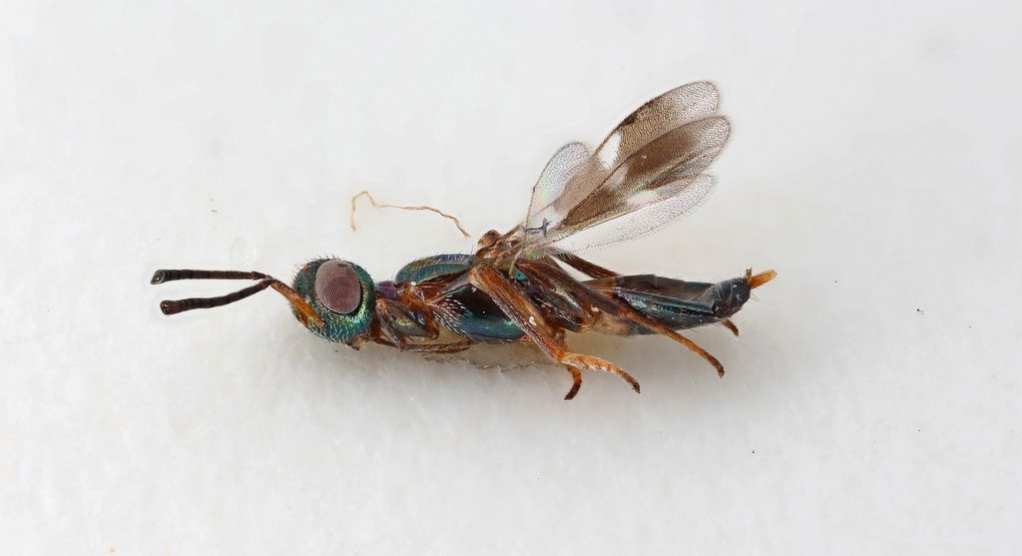
Eupelmidae, Eupelminae: Anastatus ? catalonicus Bolívar y Pieltain female, BMNH specimen BMNH(E)1414497 [/07/1994, Box Hill, Dorking, Surrey, England coll. Noyes] (photo K. McCormack)

BMNH det. Noyes, added here. This species, if it is the same as that found frequently in SW France, is probably unnamed (G. Delvare pers. comm. to R.R. Askew). See Fig. 6 for habitus.

#### Anastatus ruficaudus

Ferrière, 1954

##### Distribution

Wales

##### Notes

Added by Askew (1987)

#### Eupelmus

Dalman, 1820

MACRONEURA Walker, 1837
HOLCEUPELMUS Cameron, 1905
CHARITOPELLA Crosby, 1909
BRUCHOCIDA Crawford, 1913
EPISOLINDELIA Girault, 1914
LINDESONIUS Brèthes, 1916
RAFA Brèthes, 1916
CHARITOPODINUS Bridwell, 1918
EUPELMELLA Masi, 1919
LEPIDEUPELMUS Timberlake, 1926
NEOSOLINDENIA Gourlay, 1928
FANAMOKALA Risbec, 1960
EURONMACRA Kalina, 1981
COCCEUPELMUS Kalina, 1984

##### Notes

*Fanamokala* Risbec, 1960 was originally described in Pteromalidae and synonymised under *Eupelmus* in Fusu et al. (2015).

Species of *Eupelmus* removed from the British and Irish list:

[*karschii* Lindemann, 1887]

Only recorded as British in a catalogue of parasitoids and predators of insect pests (Thompson 1955).

#### Eupelmus annulatus

Nees, 1834

albicauda (Spinola, 1811, *Diplolepis*) nom. nud.
annulata (Spinola, 1811, *Diplolepis*) nom. nud.
nubilipennis Förster, 1860

##### Distribution

England

##### Notes

Taxonomy follows Gibson (2011); published records of *E. annulatus* reared from cynipid galls actually refer to *E. spongipartus* but *E. annulatus* is also present in Britain (Gibson 2011; R.R.Askew coll.)

#### Eupelmus atropurpureus

Dalman, 1820

hemipterus (Fonscolombe, 1832, *Cleonymus*)
atrocoeruleus Thomson, 1878

##### Distribution

England, Wales

#### Eupelmus azureus

Ratzeburg, 1844

cordairii (Ratzeburg, 1844, *Pteromalus*)
spongipartus Förster, 1860

##### Distribution

England

##### Notes

Taxonomy follows Al khatib et al. (2014); specimens in Askew coll.

#### Eupelmus confusus

Al khatib, 2014

##### Distribution

England

##### Notes

Added by Gibson and Fusu (2016)

#### Eupelmus hartigi

Förster, 1841

#### Eupelmus kiefferi

De Stefani, 1898

##### Distribution

England

##### Notes

Added by Gibson and Fusu (2016)

#### Eupelmus martelli

Masi, 1941

##### Distribution

England

##### Notes

BMNH det. Fusu, added here

#### Eupelmus memnonius

Dalman, 1820

##### Notes

Only tentatively included on the British list by Bouček and Graham (1978) on the basis of Curtis (1829) record; here carried over from that list.

#### Eupelmus microzonus

Förster, 1860

##### Notes

Askew coll., det Askew, added here

#### Eupelmus opacus

Delvare, 2015

##### Distribution

England

##### Notes

Added by Gibson and Fusu (2016)

#### Eupelmus pini

Taylor, 1927

aloysii Russo, 1938
sculpturatus Nikol’skaya, 1952
suecicus Hedqvist, 1963
carinifrons Yang, 1996

##### Distribution

England

##### Notes

Taxonomy follows Gibson (2011)

#### Eupelmus pullus

Ruschka, 1921

##### Distribution

England, Scotland

#### Eupelmus spongipartus

Förster, 1860

##### Distribution

England

##### Notes

See comment under *E. annulatus*

#### Eupelmus urozonus

Dalman, 1820

zonurus Dalman, 1820
orthia (Walker, 1839, *Pteromalus*)
audouinii (Ratzeburg, 1844, Pteromalus)
dufourii (Ratzeburg, 1848, *Pteromalus*)

##### Distribution

England, Wales, Scotland, Ireland

#### Eupelmus vesicularis

(Retzius, 1783)

Ichneumon vesicularis Retzius, 1783
hemipterus (Spinola, 1783, *Cleonymus*)
degeeri Dalman, 1820
geeri Nees, 1834
maculipes (Walker, 1837, *Macroneura*)
canadensis (Provancher, 1883, *Theocolax*)
saltator (Lindeman, 1887, *Euryscapus*)
albitarsis Costa, 1888
coleopterophagus (Girault, 1916, *Eupelminus*)

##### Distribution

England, Wales, Ireland

##### Notes

See Fig. 7 for habitus

#### Merostenus

Walker, 1837

EUPELMINUS Dalla Torre, 1897
UROCRYPTUS Westwood, 1839

#### Merostenus excavatus

(Dalman, 1820)

Eupelmus excavatus Dalman, 1820
phedyma Walker, 1837

##### Distribution

England

### Family Eurytomidae Walker, 1832

#### Eurytominae

Walker, 1832

#### Aximopsis

Ashmead, 1904

AXIMOGASTRA Ashmead, 1904
AXIMOPSIS Ashmead, 1904
MESOEURYTOMA Cameron, 1911
STIREURYTOMA Cameron, 1911
CONOAXIMA Brues, 1922
EURYTOMARIA Masi, 1943
AXIMOGASTROMA Narendran, 1994

#### Aximopsis nodularis

Boheman, 1836

argele Walker, 1844
rubicola Giraud, 1866
aequalis Walker, 1873
petiolata Thomson, 1876
petiolulata Dalla Torre, 1898

##### Distribution

England

#### Bruchophagus

Ashmead, 1888

SYSTOLODES Ashmead, 1888
EURYSYSTOLE Girault, 1913
PHYLLOXEROXENOIDES Girault, 1913

#### Bruchophagus atra

(Walker, 1832)

Isosoma atrum Walker, 1832
globiceps Bouček, 1954

##### Distribution

England, Wales

##### Notes

Transferred to *Bruchophagus* by Lotfalizadeh et al. (2007)

#### Bruchophagus gibbus

(Boheman, 1836)

Eurytoma gibba Boheman, 1836
brachycera (Boheman, 1836, *Eurytoma*)
mucianus (Walker, 1848, *Eurytoma*)
funebris (Howard, 1880, *Eurytoma*)
brevicornis (Ashmead, 1894, *Systolodes*)

##### Distribution

England

#### Bruchophagus intermedius

(Thomson, 1876)

Eurytoma intermedia Thomson, 1876

#### Bruchophagus platypterus

(Walker, 1834)

Systole platyptera Walker, 1834
kolobovae Fedoseeva, 1956

##### Distribution

England

#### Eurytoma

Illiger, 1807

DECATOMA Spinola, 1811
ENNETOMA Dahlbom, 1857
EVOXYSOMA Ashmead, 1888
EUOXYSOMA Dalla Torre, 1898
BEPHRATELLA Girault, 1913
IPIDEURYTOMA Bouček & Novicky, 1954
AHTOLA Claridge, 1961
DESANTISCA Burks, 1971

#### Eurytoma aciculata

Ratzeburg, 1848

##### Distribution

England

#### Eurytoma aethiops

Boheman, 1836

sittace Walker, 1844

#### Eurytoma afra

Boheman, 1836

albimana Boheman, 1836
saliciperdae Mayr, 1878
polygraphi (Ashmead, 1894, *Decatomidea*)
spessivtsevi (Bouček & Novicky, 1954, *Ipideurtyoma*)

##### Distribution

England

#### Eurytoma apicalis

Walker, 1832

Eurytoma apicalis ?*annulipes* Walker, 1832
Eurytoma apicalis ?*gracilis* Walker, 1832
Eurytoma apicalis ?*minor* Boheman, 1836

#### Eurytoma appendigaster

(Swederus, 1795)

Pteromalus appendigaster Swederus, 1795

##### Distribution

England, Wales

#### Eurytoma arctica

Thomson, 1876

##### Distribution

England, Scotland

#### Eurytoma aspila

(Walker, 1836)

Decatoma aspilus Walker, 1836
nicaeae (Walker, 1844, *Decatoma*)
phanacidis Mayr, 1878

##### Distribution

England

#### Eurytoma brunniventris

Ratzeburg, 1852

##### Distribution

England, Scotland

#### Eurytoma castor

Claridge, 1959

##### Distribution

England

#### Eurytoma centaureae

Claridge, 1960

##### Distribution

England

#### Eurytoma collaris

Walker, 1832

minuta Walker, 1832
micipsa Walker, 1844
intermissa Walker, 1871
arrhenatheri Erdös, 1969
brachypodii Erdös, 1969
bromi Erdös, 1969

##### Distribution

England

#### Eurytoma compressa

(Fabricius, 1794)

Cynips compressa Fabricius, 1794
tibialis Boheman, 1836
angulata Thomson, 1876
claripennis Thomson, 1876
dilatata Thomson, 1876

##### Distribution

England, Scotland

#### Eurytoma crassinervis

Thomson, 1876

##### Distribution

Wales

#### Eurytoma curculionum

Mayr, 1878

##### Distribution

England

#### Eurytoma curta

Walker, 1832

##### Distribution

England

#### Eurytoma cynipsea

Boheman, 1836

nasalis Thomson, 1876

##### Distribution

England

##### Notes

Omitted by Bouček and Graham (1978); first recorded as British by Bouček (1965)

#### Eurytoma danuvica

Erdös, 1955

Eurytoma danuvica ?*insignis* Walker, 1871

##### Distribution

England

#### Eurytoma dentata

Mayr, 1878

fulvipes Crawford, 1910
nesiotes Crawford, 1911
denticoxa Gahan, 1919
dentipecta Gahan, 1919

##### Distribution

England

#### Eurytoma erdoesi

Szelényi, 1974

fumipennis Erdös, 1969 preocc.

##### Distribution

Wales

##### Notes

Added by Dawah et al. (1995)

#### Eurytoma exempta

Walker, 1871

#### Eurytoma flavimana

Boheman, 1836

cestius Walker, 1848
nobbei Mayr, 1878
inquilinum (Rimsky-Korsakov, 1914, *Isosoma*)

##### Distribution

England, Wales

#### Eurytoma fumipennis

Walker, 1836

brevicollis Walker, 1846
euphorbiae Zerova, 1971

##### Distribution

England

#### Eurytoma hypochoeridis

Claridge, 1960

culmicola Zerova, 1986

##### Distribution

England

#### Eurytoma laserpitii

Mayr, 1878

##### Notes

Added by Jennings (2011)

#### Eurytoma longipennis

Walker, 1832

#### Eurytoma maura

Boheman, 1836

auricoma Mayr, 1878

#### Eurytoma mayri

Ashmead, 1887

diastrophi Mayr, 1878

##### Distribution

England, Scotland

#### Eurytoma melanoneura

Walker, 1871

##### Distribution

England

##### Notes

Removed from synonymy with *E. morio* by Delvare et al. (2014)

#### Eurytoma morio

Boheman, 1836

acuminata Walker, 1834
scultenna Walker, 1844
eccoptogastri Ratzeburg, 1844
flavoscapularis Ratzeburg, 1844
flavovaria Ratzeburg, 1844
ischioxanthos Ratzeburg, 1844
umbilicata Thomson, 1876
bargaglii Rondani, 1877
fraxincola Hedqvist, 1963

##### Distribution

England

#### Eurytoma paraliae

Graham, 1984

##### Distribution

England, Wales

##### Notes

Added by Graham (1984)

#### Eurytoma phalaridis

Graham, 1974

##### Distribution

England

#### Eurytoma pistaciae

Rondani, 1877

pistacina Rondani, 1877
setigera Mayr, 1878

##### Distribution

England

#### Eurytoma pollux

Claridge, 1959

##### Distribution

England

#### Eurytoma punctatella

Zerova, 1978

##### Notes

Added by Jennings (2005b)

#### Eurytoma robusta

Mayr, 1878

##### Distribution

England

#### Eurytoma rosae

Nees, 1834

pubicornis Boheman, 1836

##### Distribution

England

#### Eurytoma roseni

Claridge, 1959

##### Distribution

England, Wales

#### Eurytoma rufipes

Walker, 1832

#### Eurytoma serratulae

(Fabricius, 1798)

Cynips serratulae Fabricius, 1798
tristis Mayr, 1878

##### Distribution

England

#### Eurytoma strigifrons

Thomson, 1876

aylaxioides Andriescu, 1971

#### Eurytoma tapio

Claridge, 1959

##### Distribution

England

#### Eurytoma tumida

Walker, 1844

grahami Zerova, 1994

##### Distribution

England

#### Eurytoma verticillata

(Fabricius, 1798)

Ichneumon verticillatum Fabricius, 1798
longulum (Walker, 1832, *Isosoma*)
simile (Walker, 1832, *Isosoma*)
costata Ratzeburg, 1848
hyponomeutae Erdös, 1957

#### Mangoma

Subba Rao, 1986

#### Mangoma salicis

Walker, 1834

humeralis Förster, 1841
salicis Thomson, 1876 preocc.

##### Distribution

England, Scotland

#### Sycophila

Walker, 1871

TINEOMYZA Rondani, 1872
ISANISA Walker, 1875
PSEUDISA Walker, 1875
DECATOMIDEA Ashmead, 1888
EUDECATOMA Ashmead, 1888

#### Sycophila biguttata

(Swederus, 1795)

Pteromalus biguttatus Swederus, 1795
fasciata (Fonscolombe, 1832, *Cinips*)
cooperi (Curtis, 1831, *Decatoma*)
obscura (Curtis, 1831, *Decatoma*)
immaculata (Walker, 1832, *Decatoma*)
Sycophila biguttata ?*plana* (Walker, 1832, *Decatoma*)
signata (Nees, 1834, *Eurytoma*)
semifasciata (Walker, 1834, *Decatoma*)
Sycophila biguttata ?*flavicornis* (Walker, 1836, *Eurytoma*)
pistacina (Rondani, 1872, *Chalcis*)
inaequalis (Thomson, 1876, *Decatoma*)
incrassata (Thomson, 1876, *Decatoma*)
strigifrons (Thomson, 1876, *Decatoma*)
mallorcae (Hedqvist, 1962, *Eudecatoma*)

##### Distribution

England

##### Notes

Claridge (1959) recognised *S. plana* and *S. flavicornis* as possible synonyms of *S. biguttata*

#### Sycophila binotata

(Fonscolombe , 1832)

Cynips binotata Fonscolombe, 1832
plagiotrochi (Mayr, 1905, *Decatoma*)

##### Distribution

England

##### Notes

Added by Notton et al. (2014); thought to have been introduced from Italy with saplings

#### Sycophila concinna

(Boheman, 1836)

Eurytoma concinna Boheman, 1836
mesomelas (Walker, 1836, *Decatoma*)

##### Distribution

England, Scotland

#### Sycophila fasciata

(Thomson, 1876)

Decatoma fasciata Thomson, 1876
stagnalis (Erdös, 1947, *Decatoma*)

##### Distribution

England

#### Sycophila flavicollis

(Walker, 1834)

Decatoma flavicollis Walker, 1834
xanthomelas (Boheman, 1836, *Eurytoma*)
neesii (Förster, 1841, *Eurytoma*)

##### Distribution

England

#### Sycophila mayri

(Erdös, 1959)

Eudecatoma mayri Erdös, 1959

##### Notes

Added by Jennings (2009a)

#### Sycophila mellea

(Curtis, 1831)

Decatoma mellea Curtis, 1831
amsterdamensis (Girault, 1917, *Decatoma*)
rimskykorsakovi (Erdös, 1952, *Decatoma*)

##### Distribution

England, Wales

#### Sycophila submutica

(Thomson, 1876)

Decatoma submutica Thomson, 1876
caudata (Thomson, 1876, *Decatoma*)
emarginata Abdul-Rassoul, 1980

##### Distribution

England

##### Notes

Omitted by Bouček and Graham (1978); first recorded as British by Claridge (1959).

#### Sycophila variegata

(Curtis, 1831)

Decatoma variegata Curtis, 1831
minuta (Curtis, 1831, *Decatoma*)
unicolor (Curtis, 1831, *Decatoma*)
rufa (Fonscolombe, 1832, *Cinips*)
tenuicornis (Walker, 1832, *Decatoma*)
gilva Abdul-Rassoul, 1980

##### Distribution

England

##### Notes

**Figure 8.**
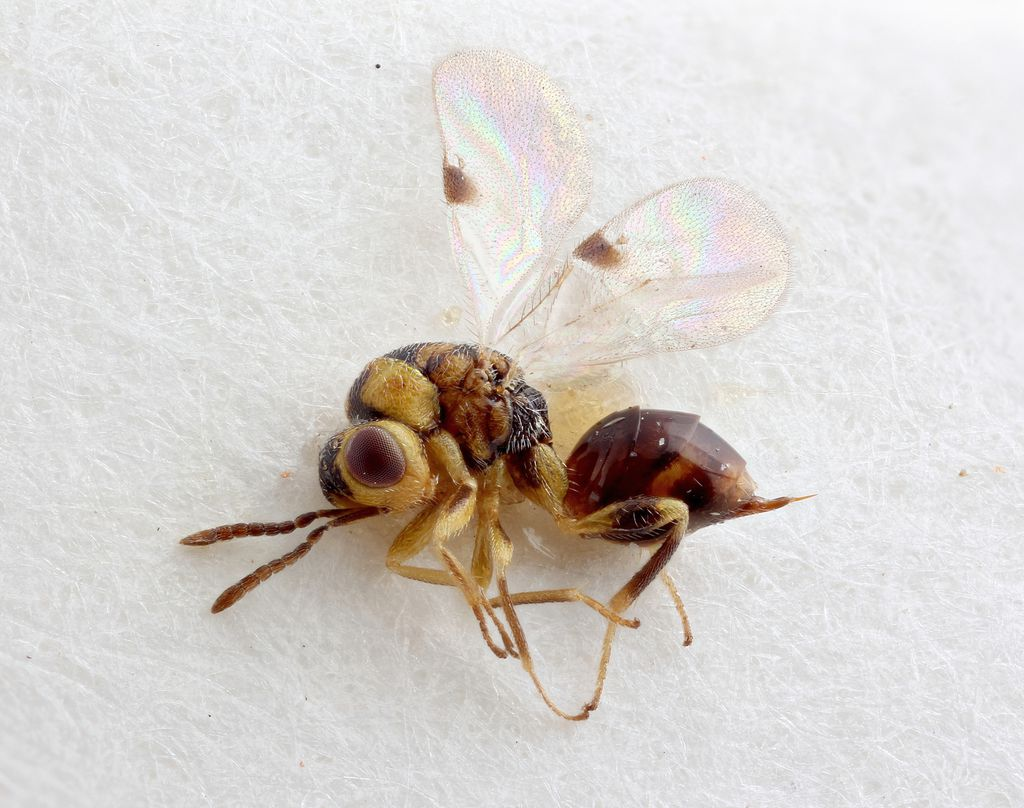
Eurytomidae, Eurytominae: *Sycophila variegata* (Curtis) female, BMNH specimen BMNH(E)1414552 [/08/1975, Thames Ditton, Surrey, England, coll. Bouček] (photo K. McCormack)

See Fig. 8 for habitus

#### Systole

Walker, 1832

#### Systole albipennis

Walker, 1832

nitida (Walker, 1832, *Eurytoma*)
brevicornis (Boheman, 1836, *Eurytoma*)

##### Distribution

England

#### Systole conspicua

Erdős, 1951

##### Distribution

England

##### Notes

**Figure 9.**
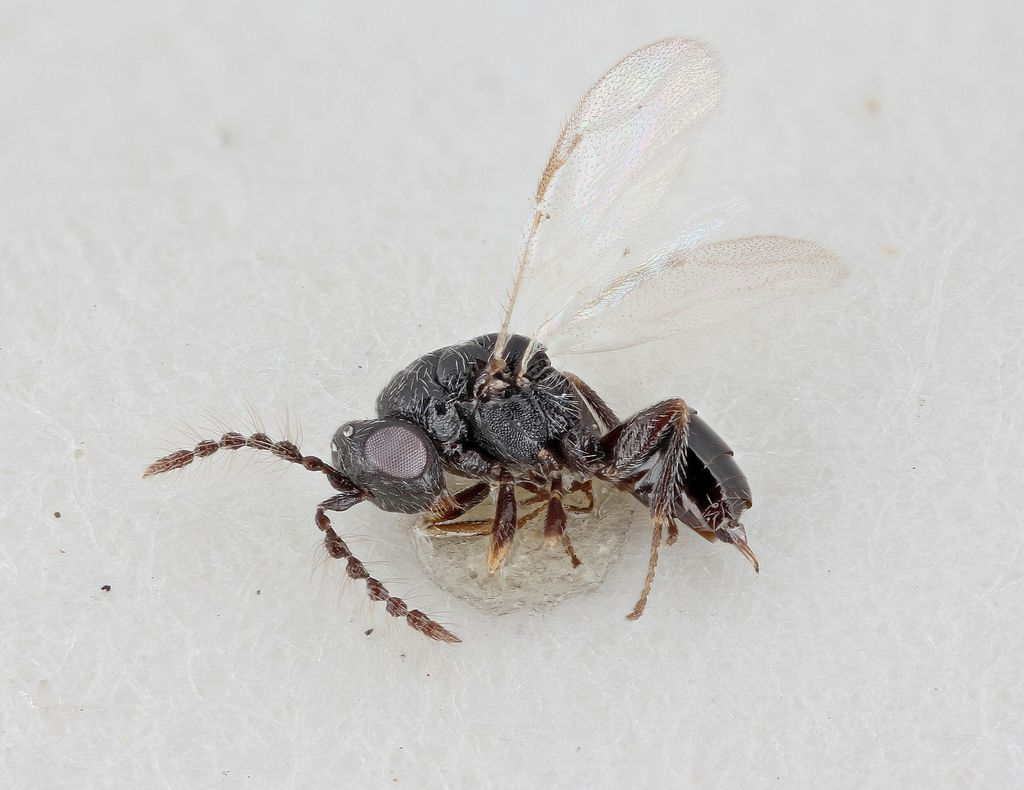
Eurytomidae, Eurytominae: *Systole conspicua* Erdös male, BMNH specimen BMNH(E)1414495 [/09/1976, Sussex, England, coll. Silvertown]] (photo K. McCormack)

BMNH, det. Noyes, added here. See Fig. 9 for habitus.

#### Systole tuonela

Claridge, 1959

##### Distribution

England

#### Tetramesa

Walker, 1848

ISOSOMA Walker, 1832
HARMOLITA Motschulsky, 1863
PHILACHYRA Walker, 1871
ISOSOMOCHARIS Ashmead, 1888
XANTHOSOMA Ashmead, 1888
HARMOLYTA Dalla Torre, 1898
URIOS Girault, 1911
EXANTHOSOMA Girault, 1915
ISTHMOSOMA Hedicke, 1921
GAHANIOLA Erdös, 1952

#### Tetramesa aequalis

(Walker, 1871)

Isosoma aequalis Walker, 1871

##### Distribution

England

#### Tetramesa airae

(Schlechtendal, 1891)

Isosoma airae Schlechtendal, 1891
ruebsaameni (Hedicke, 1921, *Isosoma*)

##### Distribution

England, Wales

#### Tetramesa albomaculatum

(Ashmead, 1894)

Isosoma albomaculatum Ashmead, 1894

##### Distribution

England, Wales

#### Tetramesa angustatum

(Walker, 1832)

Isosoma angustatum Walker, 1832
guttula (Boheman, 1836, *Eurytoma*)

##### Distribution

England

#### Tetramesa angustipenne

(Walker, 1832)

Isosoma angustipenne Walker, 1832
melanomera (Walker, 1871, *Isosoma*)
opaca (Thomson, 1876, *Isosoma*)
longicolle (Hedicke, 1921, *Isosoma*)

##### Distribution

England

#### Tetramesa brevicollis

(Walker, 1836)

Isosoma brevicolle Walker, 1836
hieronymi (Hedicke, 1921, *Isosoma*) preocc.
hieronymi (Schlechtendal, 1891, *Isosoma*)

#### Tetramesa brevicornis

(Walker, 1832)

Isosoma brevicorne Walker, 1832
clavicornis (Walker, 1832, *Isosoma*)
clavicorne (Walker, 1871, *Isosoma*) preocc.
tibiale (Walker, 1871, *Isosoma*)
ruschkai (Hedicke, 1921, *Isosoma*)

##### Distribution

England

#### Tetramesa brevipennis

(Walker, 1836)

Isosoma brevipenne Walker, 1836
filicorne (Hedicke, 1921, *Isosoma*)

##### Distribution

England

#### Tetramesa brevis

(Walker, 1832)

Isosoma brevis Walker, 1832

#### Tetramesa breviventris

(Walker, 1832)

Isosoma breviventre Walker, 1832

#### Tetramesa calamagrostidis

(Schlechtendal, 1891)

Eurytoma calamagrostidis Schlechtendal, 1891
calamagrostidis (Hedicke, 1921, *Isosoma*) preocc.

##### Distribution

England, Wales

#### Tetramesa cornuta

(Walker, 1832)

Isosoma cornutum Walker, 1832
dissimile (Walker, 1832, *Isosoma*)
agropyrophila (Phillips & Emery, 1918, *Harmolita*)

##### Distribution

England

#### Tetramesa crassicornis

(Walker, 1832)

Isosoma crassicorne Walker, 1832
iarbas Walker, 1848
jarbas Dalla Torre, 1898

#### Tetramesa eximia

(Giraud, 1863)

Isosoma eximium Giraud, 1863
giganteum (Hedicke, 1921, *Isosoma*)

##### Distribution

England, Wales

#### Tetramesa fulvicollis

(Walker, 1832)

Isosoma fulvicolle Walker, 1832
flavicolle (Walker, 1834, *Isosoma*)
tenuipes (Walker, 1871, *Isosoma*)

##### Distribution

England

#### Tetramesa fumipennis

(Walker, 1832)

Isosoma fumipenne Walker, 1832
claripenne (Walker, 1871, *Isosoma*)

##### Distribution

England

#### Tetramesa hyalipennis

(Walker, 1832)

Isosoma hyalipenne Walker, 1832
pilicornis (Boheman, 1836, *Eurytoma*)
graminicola (Giraud, 1863, *Isosoma*)

##### Distribution

England, Wales, Ireland

##### Notes

**Figure 10.**
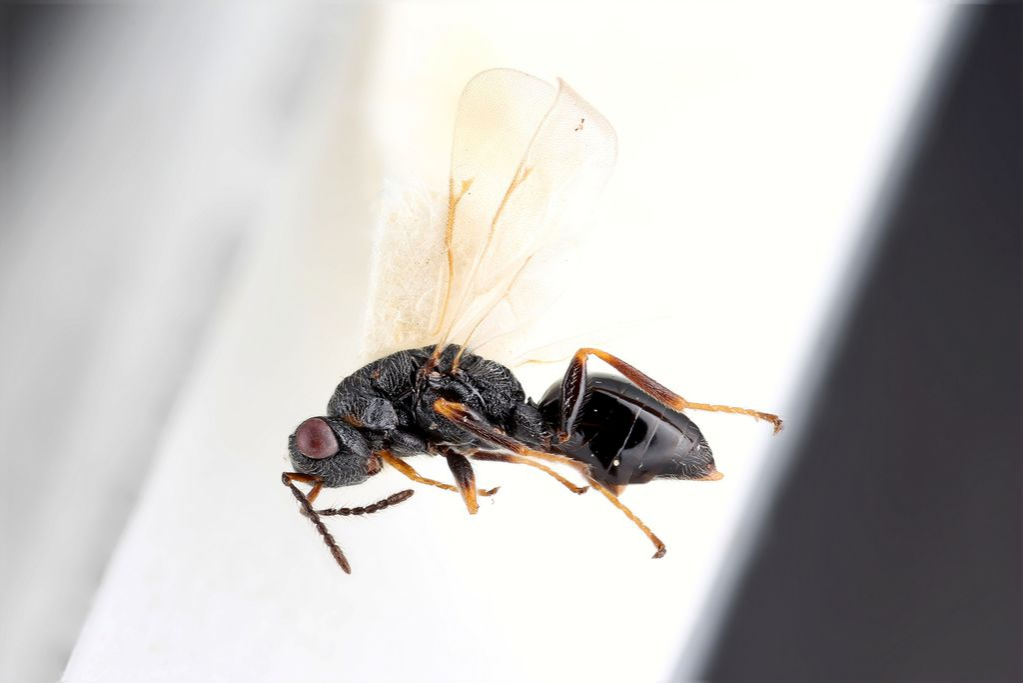
Eurytomidae, Eurytominae: *Tetramesa hyalipennis* (Walker) female, BMNH specimen BMNH(E)1414547 [Merthyr Mawr, Bridgend, Wales, coll. Dawah] (photo K. McCormack)

See Fig. 10 for habitus

#### Tetramesa inaequalis

(Thomson, 1876)

Isosoma inaequalis Thomson, 1876

#### Tetramesa juncea

(Walker, 1871)

Isosoma juncea Walker, 1871

#### Tetramesa laothoe

(Walker, 1843)

Isosoma laothoe Walker, 1843

#### Tetramesa linearis

(Walker, 1832)

Isosoma lineare Walker, 1832
nigra (Fonscolombe, 1832, *Cinips*)
attenuatum (Walker, 1832, *Isosoma*)
canaliculata (Walker, 1871, *Isosoma*)
agropyri (Schlechtendal, 1891, *Isosoma*)
atlantica (Phillips & Emery, 1918, *Harmolita*)
dimidiatum (Hedicke, 1921, *Isosoma*)

##### Distribution

England, Wales

#### Tetramesa longicornis

(Walker, 1832)

Isosoma lingicorne Walker, 1832
depressum (Walker, 1832, *Isosoma*)
phalaridis (Phillips & Poos, 1922, *Harmolita*)

##### Distribution

England, Wales

#### Tetramesa longula

(Dalman, 1820)

Eurytoma longula Dalman, 1820
dactylicola (Phillips & Emery, 1918, *Harmolita*)

##### Distribution

England

#### Tetramesa maculata

(Howard, 1896)

Isosoma maculatum Howard, 1896

#### Tetramesa maderae

(Walker, 1849)

Isosoma maderae Walker, 1849
ips (Walker, 1871, *Philachyra*)
apterum (Portschinsky, 1881, *Isosoma*)
tritici (Riley, 1882, *Isosoma*)
grande (Riley, 1884, *Isosoma*)
vestali (Girault, 1911, *Urios*)

#### Tetramesa maritima

(Hedicke, 1921)

Isosoma maritimum Hedicke, 1921

##### Distribution

England, Wales, Scotland

##### Notes

Omitted by Bouček and Graham (1978); Hedicke (1921) recorded this species (as a subspecies of *T. hyalipennis*) from England and Scotland on the basis of records of its galls by earlier authors; *T. maritima* was distinguished from *T. hyalipennis* by Bailey (1967).

#### Tetramesa minor

(Walker, 1832)

Isosoma minor Walker, 1832
elongatum (Walker, 1832, *Isosoma*)

#### Tetramesa nepe

(Walker, 1844)

Isosoma nepe Walker, 1844

#### Tetramesa paluda

Graham, 1974

#### Tetramesa petiolata

(Walker, 1832)

Isosoma petiolata Walker, 1832

##### Distribution

England, Wales

#### Tetramesa phleicola

(Hedicke, 1921)

Isosoma phleicola Hedicke, 1921

##### Distribution

England

#### Tetramesa pusilla

(Walker, 1832)

Isosoma pusillum Walker, 1832

#### Tetramesa robusta

(Walker, 1871)

Isosoma robusta Walker, 1871

#### Tetramesa subfumata

(Walker, 1871)

Isosoma subfumata Walker, 1871

#### Tetramesa szelenyii

Graham, 1974

#### Tetramesa tenuicornis

(Walker, 1832)

Isosoma tenuicorne Walker, 1832

#### Tetramesa vacillans

(Walker, 1836)

Isosoma vacillans Walker, 1836

### Family Mymaridae Haliday, 1833

#### Alaptus

Westwood, 1839

PARVULINUS Mercet, 1912
METALAPTUS Malenotti, 1917

#### Alaptus antennatus

Kryger, 1950

##### Distribution

England

#### Alaptus extremus

Soyka, 1939

terebrans Kryger, 1950

##### Distribution

England

#### Alaptus fusculus

Walker, 1846

foersteri Soyka, 1939

##### Distribution

England, Ireland

#### Alaptus magnus

Cheke & Turner, 1974

##### Distribution

England

##### Notes

Listed as a tentative junior synonym of *A. fusculus* by Bouček and Graham (1978)

#### Alaptus minimus

Westwood, 1839

crassus Kryger, 1950
uncinatus Kryger, 1950

##### Distribution

England

#### Alaptus pallidornis

Förster, 1856

excisus Westwood, 1879

##### Distribution

England

#### Alaptus richardsi

Hincks, 1960

##### Distribution

England

#### Anagrus

Haliday, 1833

PTERATOMUS Packard, 1864
PACKARDIELLA Ashmead, 1904
PARANAGRUS Perkins, 1905
ANAGRELLA Bakkendorf, 1962

#### Anagrus atomus

(Linnaeus, 1767)

Ichneumon atomus Linnaeus, 1767
bartheli Tullgren, 1916
minimus Menozzi, 1942
tullgreni Hedqvist, 1954
devius Soyka, 1956
gabitzi Soyka, 1956
hundsheimensis Soyka, 1956
kressbachi Soyka, 1956
lemonicolor Soyka, 1956
levis Soyka, 1956
stammeri Soyka, 1956
varius Soyka, 1956

##### Distribution

England, Scotland, Wales

#### Anagrus avalae

Soyka, 1956

nigriceps Girault, 1915 preocc.
arcuatus Soyka, 1956
diversicornis Soyka, 1956
valkenburgensis Soyka, 1956
oregonensis Triapitsyn, 1996

##### Distribution

England

##### Notes

Added by Chiappini and Triapitsyn (1999)

#### Anagrus bakkendorfi

Soyka, 1946

latipennis Soyka, 1956

##### Distribution

England

##### Notes

Added by Triapitsyn and Berezovskiy (2004)

#### Anagrus breviphragma

Soyka, 1956

longigaster Soyka, 1956
ovipositor Soyka, 1956
supremus Soyka, 1956
vacuipennis Soyka, 1956
silwoodensis Walker, 1979

##### Distribution

England, Wales

##### Notes

Added by Walker (1979)

#### Anagrus ensifer

Debauche, 1948

##### Distribution

England, Wales

#### Anagrus fennicus

Soyka, 1956

##### Distribution

England, Wales

##### Notes

Added by Triapitsyn and Berezovskiy (2004)

#### Anagrus incarnatus

Haliday, 1833

debilis Förster, 1847
flavus Förster, 1847
pallidus Förster, 1847
pallipes Förster, 1861
pallidipes Dalla Torre, 1898
hydrophilus Ashmead, 1905
danicus Soyka, 1956
incarnatosimilis Soyka, 1956
neopallidus Soyka, 1956
pallidior Soyka, 1956
pulcher Soyka, 1956
pulcherrimus Soyka, 1956
varicolor Soyka, 1956
stenocrani Walker, 1979
mutans Walker, 1979

##### Distribution

England, Wales, Ireland

#### Anagrus nigriceps

(Smits van Burgst, 1914)

Litus nigriceps Smits van Burgst, 1914

##### Distribution

England

##### Notes

Added by Triapitsyn and Berezovskiy (2004)

#### Anagrus obscurus

Förster, 1861

##### Distribution

England

##### Notes

Added by Triapitsyn and Berezovskiy (2004)

#### Anagrus similis

Soyka, 1956

holci Walker, 1979

##### Distribution

England, Ireland

##### Notes

Added by Walker (1979)

#### Anagrus subfuscus

Förster, 1847

##### Distribution

England

#### Anagrus ustulatus

Haliday, 1833

parvus Soyka, 1956

##### Distribution

England, Scotland, Wales, Ireland

#### Anaphes

Haliday, 1833

PATASSON Walker, 1846
PANTHUS Walker, 1846
FLABRINUS Rondani, 1877
ANAPHOIDEA Girault, 1909
CLINOMYMAR Kieffer, 1913
YUNGABURRA Girault, 1933
FERRIERELLA Soyka, 1946
SYNANAPHES Soyka, 1946
FERRIERELLA Soyka, 1946
FULMEKIELLA Soyka, 1946
HOFENEDERIA Soyka, 1946
SYNANAPHES Soyka, 1946
ANTONIELLA Soyka, 1950
MARIELLA Soyka, 1950
STAMMERIELLA Soyka, 1950
AUSTRANAPHES Ogloblin, 1962

#### Anaphes aries

Debauche, 1948

#### Anaphes auripes

Walker, 1846

##### Distribution

Ireland

#### Anaphes collinus

Walker, 1846

#### Anaphes crassicornis

(Walker, 1846)

Panthus crassicornis Walker, 1846

#### Anaphes diana

(Girault, 1911)

Anaphoidea diana Girault, 1911
lameerei (Debauche, 1948, *Patasson*)

##### Distribution

England

#### Anaphes dorcas

(Debauche, 1948)

Patasson dorcas Debauche, 1948

#### Anaphes fuscipennis

Haliday, 1833

pratensis Förster, 1847
capitulata (Soyka, 1949, *Ferrierella*)
filicornis (Soyka, 1949, *Ferrierella*)
maculata (Soyka, 1949, *Ferrierella*)
neopratensis (Soyka, 1949, *Ferrierella*)
stammeri (Soyka, 1949, *Ferrierella*)

##### Distribution

Ireland

#### Anaphes globosicornis

(Soyka, 1949)

Mymar globosicornis Soyka, 1949

##### Distribution

England

##### Notes

BMNH, det. Thuroczy, added here. The specimen was collected in Kew Gardens and may be an introduction (the species is known from Northern Europe).

#### Anaphes latipennis

Walker, 1846

#### Anaphes leptoceras

(Debauche, 1948)

Patasson leptoceras Debauche, 1948

#### Anaphes linearis

(Soyka, 1949)

Fulmekiella linearis Soyka, 1949

##### Distribution

England

##### Notes

BMNH, det. Thuroczy, added here

#### Anaphes lineipennis

(Soyka, 1949)

Anaphoidea lineipennis Soyka, 1949

##### Distribution

England

##### Notes

BMNH, det. Thuroczy, added here

#### Anaphes longicornis

Walker, 1846

##### Distribution

Ireland

#### Anaphes maialis

(Debauche, 1948)

Patasson maialis Debauche, 1948

##### Distribution

England

##### Notes

BMNH, det. Thuroczy, added here

#### Anaphes medius

Soyka, 1946

lacensis (Soyka, 1949, *Synanaphes*)
ranalteri (Soyka, 1949, *Synanaphes*)

##### Distribution

England

##### Notes

BMNH, det. Thuroczy, added here

#### Anaphes nilaparvatae

Pang & Wang, 1985

##### Distribution

England

##### Notes

BMNH, det. Triapitsyn, added here

#### Anaphes pectoralis

(Soyka, 1946)

Hofenederia pectoralis Soyka, 1946

##### Distribution

England

##### Notes

BMNH, det. Thuroczy, added here

#### Anaphes regulus

Walker, 1846

autumnalis Förster, 1847

##### Distribution

Ireland

#### Anaphes silesicus

(Soyka, 1946)

Anaphoidea silesica Soyka, 1946

##### Distribution

Ireland

##### Notes

Added by Thuróczy and O'Connor (2015)

#### Arescon

Walker, 1846

LEIMACIS Walker, 1846
LIMACIS Förster, 1856
XENOMYMAR Crawford, 1913
NEUROTES Enock, 1914

#### Arescon dimidiata

(Curtis, 1832)

Mymar dimidiatus Curtis, 1832
rufula (Förster, 1847, *Leimacis*)
flaviventris (Ryland, 1922, *Neurotes*)

##### Distribution

England, Wales

#### Arescon iridescens

(Enock, 1914)

Neurotes iridescens Enock, 1914

##### Distribution

England

#### Camptoptera

Förster, 1856

PTEROCLISIS Förster, 1856
STICHOTHRIX Förster, 1856
EOMYMAR Perkins, 1912
CONGOLIA Ghesquière, 1942
SPHEGILLA Debauche, 1948
WERTANEKIELLA Soyka, 1961
STANERIA Mathot, 1966

#### Camptoptera cardui

(Förster, 1856)

Stichothrix cardui Förster, 1856

##### Distribution

England

##### Notes

BMNH, det. Triapitsyn, added here

#### Camptoptera elongatula

Kryger, 1950

##### Distribution

England

##### Notes

Omitted by Bouček and Graham (1978)

#### Camptoptera foersteri

Girault, 1917

aula Debauche, 1948

##### Distribution

England

##### Notes

Added by Huber (2011)

#### Camptoptera papaveris

Förster, 1856

##### Notes

Girault (1915) described *C. saintpierrei* for the species which he had previously misidentified as *C. papaveris*. The identities and occurrence in Britain of both species need to be checked.

#### Camptoptera punctum

(Shaw, 1798)

Ichneumon punctum Shaw, 1798

##### Distribution

England

##### Notes

Transferred from *Anaphes* by Huber (2011)

#### Camptoptera saintpierrei

Girault, 1915

##### Distribution

England

##### Notes

See remark under *C. papaveris*

#### Camptoptera tarsalis

Kryger, 1950

##### Notes

Omitted by Bouček and Graham (1978)

#### Caraphractus

Walker, 1846

VALKERELLA Westwood, 1879

#### Caraphractus cinctus

Walker, 1846

reductus Rimsky-Korsakov, 1925
natans (Lubbock, 1864, *Polynema*)

##### Distribution

England, Scotland

#### Cleruchus

Enock, 1909

STENOPTEROMYMAR Ferrière, 1952
DOUTTIELLA Annecke, 1961
PARACLERUCHUS Yoshimoto, 1971

#### Cleruchus bakkendorfi

Debauche, 1948

##### Distribution

England

##### Notes

Added by Trjapitzin (1978)

#### Cleruchus pluteus

Enock, 1909

##### Distribution

England

#### Cleruchus taktochno

Triapitsyn, 2014

##### Distribution

England

##### Notes

BMNH, det. Triapitzyn, added here

#### Cosmocomoidea

Howard, 1908

#### Cosmocomoidea atra

(Förster, 1841)

Gonatocerus ater Förster, 1841
pannonica (Soyka, 1946, *Gonatocerus*)
schmitzi (Debauche, 1948, *Lymaenon*)
indica (Subba Rao & Kaur, 1959, *Lymaenon*)
nigroides (Narayanan & Subba Rao, 1961, *Lymaenon*)
intermedia (Botoc, 1962, *Lymaenon*)
empoascae (Subba Rao, 1966, *Lymaenon*)
populi (Viggiani, 1969, *Lymaenon*)

##### Distribution

England, Wales, Ireland

#### Cosmocomoidea latipennis

(Girault, 1911)

Gonatocerus latipennis Girault, 1911
maxima (Girault, 1911, *Gonatocerus*)

##### Distribution

England

##### Notes

Added by Triapitsyn (2013)

#### Cosmocomoidea oxypygus

(Förster, 1856)

Gonatocerus oxypygus Förster, 1856
ovicenatus (Leonard & Crosby, 1915, *Gonatocerus*)
megalura (Mathot, 1969, *Lymaenon*)

##### Distribution

England

#### Cosmocomoidea tremulae

(Bakkendorf, 1934)

Lymaenon tremulae Bakkendorf, 1934

##### Notes

Added by Triapitsyn (2013)

#### Dicopus

Enock, 1909

#### Dicopus cervus

Morley, 1931

##### Distribution

England

#### Dicopus minutissimus

Enock, 1909

##### Distribution

England

#### Erythmelus

Enock, 1909

ENAESIUS Enock, 1909
PARALLELAPTERA Enock, 1909
ANTHEMIELLA Girault, 1911

#### Erythmelus agilis

(Enock, 1909)

Enaesius agilis Enock, 1909
laticeps (Enock, 1909, *Enaesius*)
limburgensis (Soyka, 1932, *Enaesius*)

##### Distribution

England, Ireland

#### Erythmelus flavovarius

(Walker, 1846)

Panthus flavovarius Walker, 1846
goochi (Walker, 1846, *Panthus*)
parvus (Soyka, 1932, *Enaesius*)
dichromocnemus Nowicky, 1953
spinosus Mathot, 1969

##### Distribution

England, Ireland

#### Erythmelus panis

(Enock, 1909)

Parallelaptera panis Enock, 1909
foucarti (Demair, 1973, *Parallelaptera*)
panchama (Subba Rao, 1989, *Parallelaptera*)

##### Distribution

England

#### Erythmelus rex

(Girault, 1911)

Anthemiella rex Girault, 1911
margianus Trjapitzin, 1993

##### Distribution

Wales

##### Notes

Added by Triapitsyn (2003)

#### Eustochus

Haliday, 1833

#### Eustochus atripennis

(Curtis, 1832)

Mymar atripennis Curtis, 1832

##### Distribution

England

#### Gonatocerus

Nees, 1834

##### Notes

Doubtfully placed species of *Gonatocerus*:

[*flavocinctus* (Walker, 1846, *Lymaenon*) nom. nud.].

#### Gonatocerus aegyptiacus

Soyka, 1950

saipanensis (Doutt, 1955, *Lymaenon*)
tarae (Narayanan & Subba Rao, 1961, *Lymaenon*)
alami Shamim & Shafee, 1984
minor Matthews, 1986

##### Distribution

England

##### Notes

Added by Matthews (1986)

#### Gonatocerus fuscicornis

(Walker, 1846)

Lymaenon fuscicornis Walker, 1846
sulphuripes (Förster, 1847, *Rachistus*)
pictosimilis Soyka, 1946
alecto (Debauche, 1948, *Lymaenon*)
crassipes (Debauche, 1948, *Lymaenon*)
synaptus (Debauche, 1948, *Lymaenon*)

##### Distribution

England, Scotland, Wales, Ireland

#### Gonatocerus longicornis

Nees, 1834

terebrator (Förster, 1847, *Rachistus*)
cicadellae Nikol'skaya, 1951
shasthryi (Subba Rao & Kaur, 1959, *Lymaenon*)
britteni (Hincks, 1960, *Lymaenon*)
longiventris (Botoc, 1963, *Lymaenon*)
uttarodeccanus Mani & Saraswat, 1973

##### Distribution

England, Wales

#### Gonatocerus pictus

(Haliday, 1833)

Ooctonus pictus Haliday, 1833
flavus Förster, 1841

##### Distribution

England, Wales, Ireland

#### Gonatocerus rogersi

Matthews, 1986

##### Distribution

England

##### Notes

Added by Matthews (1986)

#### Litus

Haliday, 1833

MALFATTIA Meunier, 1901
NEOLITUS Ogloblin, 1935
NEOLITISCUS Ghesquière, 1946

#### Litus cynipseus

Haliday, 1833

krygeri Kieffer, 1913

##### Distribution

England, Wales

#### Lymaenon

Walker, 1846

RACHISTUS Förster, 1847
RHACHISTUS Dalla Torre, 1898
OOPHILUS Enock, 1909
AGONATOCERUS Girault, 1913
DECARTHRIUS Debauche, 1949

#### Lymaenon acuminatus

Walker, 1846

Gonatocerus acuminatus Walker, 1846
longicauda (Enock, 1909, *Oophilus*)

##### Distribution

England

#### Lymaenon aureus

(Girault, 1911)

Gonatocerus aureus Girault, 1911
chrysis Debauche, 1948, *Lymaenon*
flavus (Soyka, 1950, *Gonatocerus*) preocc.
pahlgamensis Narayanan, 1961

##### Distribution

England, Scotland, Wales

#### Lymaenon litoralis

(Haliday, 1833)

Ooctonus litoralis Haliday, 1833
radiculatus (Ahlberg, 1925, *Gonatocerus*)
effusi Bakkendorf, 1934
paludis Debauche, 1948
rhacodes Debauche, 1948
arduennae Mathot, 1969
pulchellus (Hellén, 1974, *Gonatocerus*)

##### Distribution

England, Scotland, Wales, Ireland

##### Notes

*L. cunctator* Mathot, 1969 was removed from synonymy under *L. littoralis* by Triapitsyn (2013); there is no evidence that *L. cunctator* has been recorded from Britain.

#### Lymaenon longior

(Soyka, 1946)

Gonatocerus longior Soyka, 1946

##### Distribution

England, Wales

##### Notes

Added by Matthews (1986)

#### Lymaenon novickyi

(Soyka, 1946)

Gonatocerus novickyi Soyka, 1946
fossarum Hincks, 1952

##### Distribution

England

#### Lymaenon thyrides

Debauche, 1948

##### Distribution

England

#### Mymar

Curtis, 1829

PTEROLINONONYKETRA Maláč, 1943
OGLOBLINIELLA Soyka, 1946

#### Mymar pulchellum

Curtis, 1832

spectabilis Förster, 1856
venustum Girault, 1911
obenbergeri (Maláč, 1943, *Pterolinononyketra*)

##### Distribution

England

##### Notes

**Figure 11.**
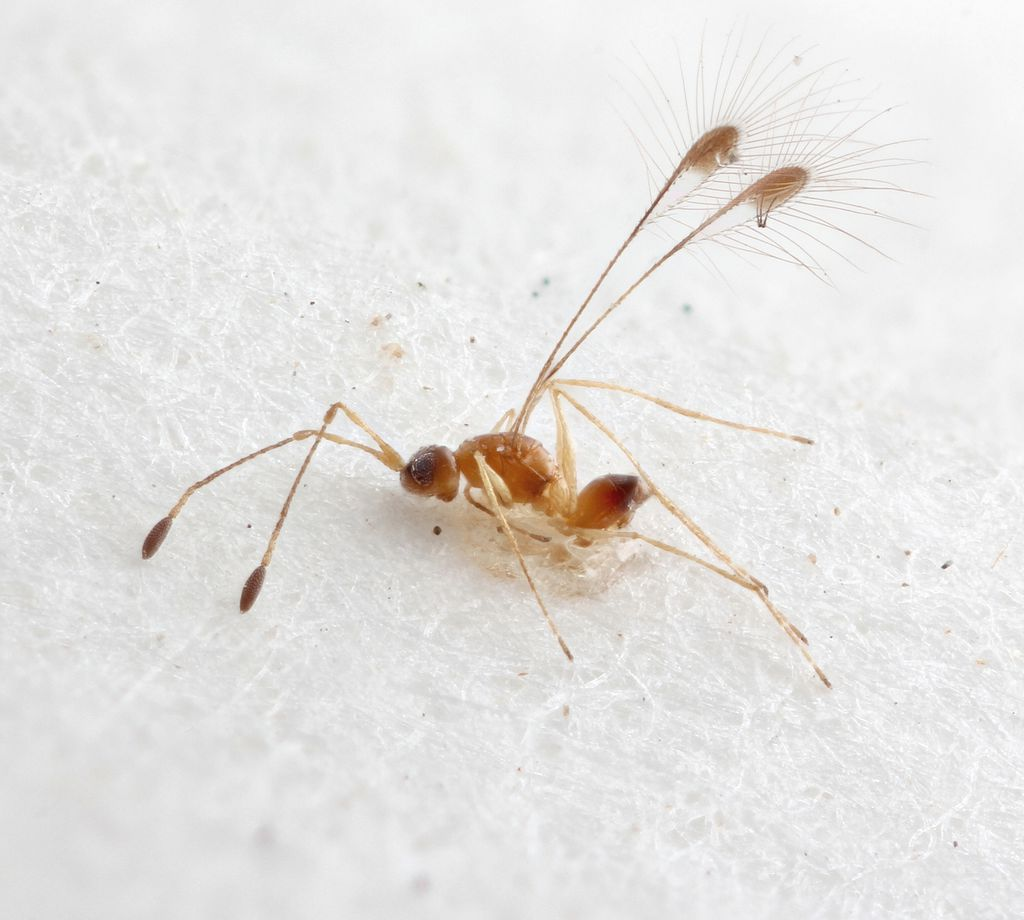
Mymaridae: *Mymar pulchellum* Curtis female, BMNH specimen BMNH(E)1414558 [/07/1979, Happy Valley, Surrey, England, coll. Noyes] (photo K. McCormack)

See Fig. 11 for habitus

#### Mymar regale

Enock, 1912

##### Distribution

England

#### Ooctonus

Haliday, 1833

SPHECOMICRUS Haliday, 1846

#### Ooctonus hemipterus

Haliday, 1833

amoenus (Förster, 1841, *Eutriche*)
atroclavatus Kieffer, 1913
foersteri Soyka, 1941
pechlaneri Soyka, 1941
wagneri Soyka, 1941
soykai Hincks, 1952

##### Distribution

England, Scotland, Wales, Ireland

#### Ooctonus insignis

Haliday, 1833

major Förster, 1847
austriacus Soyka, 1949
elegantissimus Soyka, 1949
silvestris Soyka, 1949
isotomus Mathot, 1969

##### Distribution

England, Wales, Ireland

#### Ooctonus notatus

Walker, 1846

heterotomus Förster, 1847
auripes Whittaker, 1931
atroflavus Soyka, 1949
diversicornis Soyka, 1949

##### Distribution

England, Wales, Ireland

#### Ooctonus sublaevis

Förster, 1847

polonicus Soyka, 1949
montanus Soyka, 1950
remonti Mathot, 1969
dovrensis Solem & Sveum, 1980

##### Distribution

England

##### Notes

Added by Triapitsyn (2010)

#### Ooctonus vulgatus

Haliday, 1833

americanus Girault, 1913
wesmaeli Debauche, 1948
acutiventris Soyka, 1949
collinus Soyka, 1949
stammeri Soyka, 1949
viennensis Soyka, 1949
niger Soyka, 1950
askhamensis Hincks, 1952

##### Distribution

England, Wales, Ireland

#### Polynema

Haliday, 1833

EUTRICHE Nees, 1834
DORICLYTUS Förster, 1847
CALLITRICHE Agassiz, 1848
COSMOCOMA Förster, 1856
BARYPOLYNEMA Ogloblin, 1946
MAIDLIELLA Soyka, 1946
NOVICKYELLA Soyka, 1946
XENOPOLYNEMA Ogloblin, 1960

#### Polynema albitarse

Kieffer, 1913

lygaeum Hincks, 1960

##### Distribution

England

#### Polynema atratum

Haliday, 1833

##### Distribution

England

#### Polynema bakkendorfi

Hincks, 1950

##### Distribution

England

#### Polynema brittanum

Girault, 1911

##### Distribution

England

#### Polynema euchariforme

Haliday, 1833

gracilis (Förster, 1841, *Eutriche*)

##### Distribution

England, Wales

#### Polynema flavipes

Walker, 1846

ovulorum misident.
longula Förster, 1847

##### Distribution

England

#### Polynema fumipenne

Walker, 1846

##### Distribution

England, Ireland

#### Polynema fuscipes

Haliday, 1833

elegans (Förster, 1841, *Eutriche*)
pulla Förster, 1847

##### Distribution

England, Ireland

#### Polynema gracile

(Nees, 1834)

Eutriche gracile Nees, 1834
britteni Hincks, 1950

##### Distribution

England

#### Polynema halidayi

Debauche, 1948

##### Distribution

Ireland

##### Notes

Added by Thuróczy and O'Connor (2015)

#### Polynema longicauda

Kieffer, 1913

#### Polynema microptera

Bakkendorf, 1934

#### Polynema permagnum

Soyka, 1956

#### Polynema pusillum

Haliday, 1833

##### Distribution

England, Ireland

#### Polynema reticulatum

Hincks, 1950

##### Distribution

England

#### Polynema richmondense

Hincks, 1960

##### Distribution

England

#### Polynema ruficolle

Kieffer, 1913

#### Polynema valkenburgense

Soyka, 1931

#### Polynema vitripenne

(Förster, 1847)

Doriclytus vitripenne Förster, 1847

#### Polynema waterhousei

Hincks, 1950

##### Distribution

England

#### Polynema woodi

Hincks, 1950

##### Distribution

England

#### Stephanodes

Enock, 1909

EUSTEPHANODES Ogloblin, 1967
MASONANA Yoshimoto, 1990

#### Stephanodes similis

(Förster, 1847)

Polynema similis Förster, 1847
elegans Enock, 1909 preocc.
enockii (Girault, 1911, *Polynema*)
psecas (Girault, 1911, *Polynema*)
psecas Girault, 1912 preocc.
isotoma (Debauche, 1949, *Polynema*)

##### Distribution

England, Scotland, Wales, Ireland

#### Stethynium

Enock, 1909

#### Stethynium triclavatum

Enock, 1909

faunum Girault, 1911

##### Distribution

England

### Family Ormyridae Förster, 1856

#### Ormyrus

Westwood, 1832

PERIGLYPHUS Boheman, 1834
SIPHONURA Nees, 1834
CYRTOSOMA Perris, 1840
MONOBAEUS Förster, 1860
TRIBAEUS Förster, 1860
CHRYSOIDEUS De Stefani, 1898
WANIA Risbec, 1951
AVRASYAMYRUS Doganlar, 1991

#### Ormyrus gratiosus

(Förster, 1860)

Monobaeus gratiosus Förster, 1860

##### Distribution

England

##### Notes

**Figure 12.**
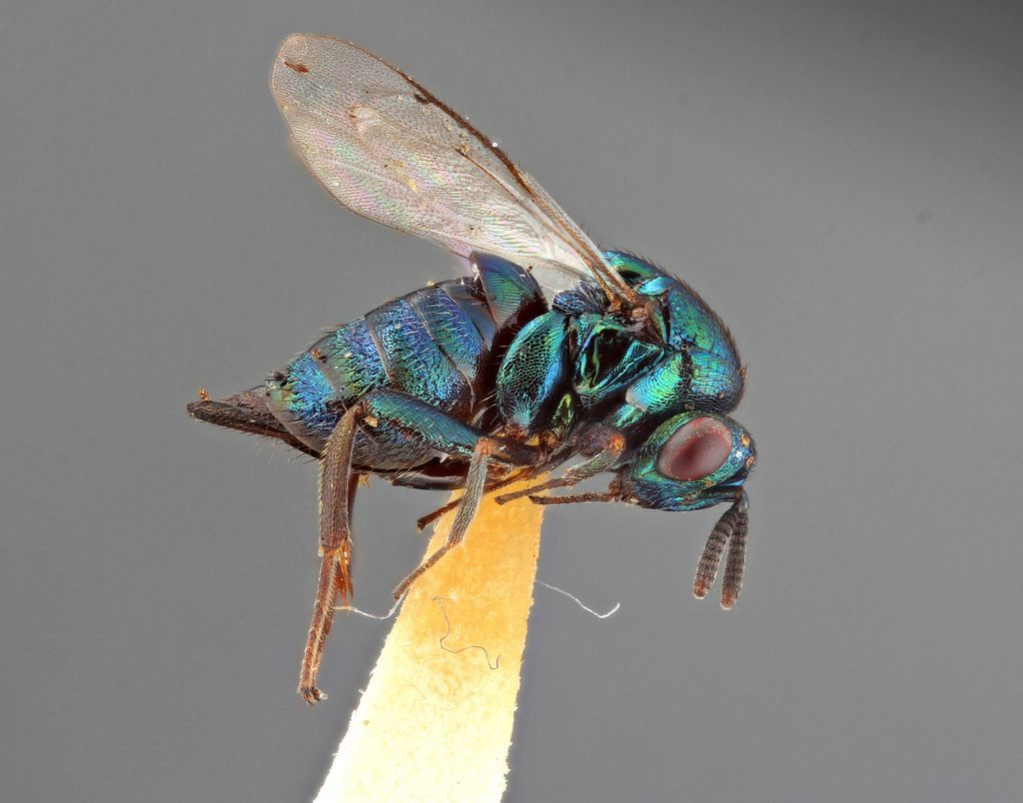
Ormyidae: *Ormyrus gratiosus* (Förster) female, BMNH specimen BMNH(E)1414486 [/10/1961, Portland, Dorset, England, coll. Clark] (photo K. McCormack)

See Fig. 12 for habitus

#### Ormyrus nitidulus

(Fabricius, 1804)

Chalcis nitidula Fabricius, 1804
tubulosa (Fonscolombe, 1832, *Cinips*)
cyanosthetus (Walker, 1847, *Siphonura*)
cyanosthetus Schmidt, 1851
gallaequercus (Dufour, 1864, *Siphonura*)
chrysidiformis (De Stefani, 1898, *Torymus*)

##### Distribution

England

#### Ormyrus papaveris

(Perris, 1840)

Cyrtosoma papaveris Perris, 1840

##### Distribution

England

##### Notes

Added by Jennings (2001)

#### Ormyrus pomaceus

(Geoffroy, 1785)

Cynips pomaceus Geoffroy, 1785
aeneicinctus Rondani, 1877
aerosus Förster, 1860
blandus Förster, 1860
nigrocyaneus Walker, 1833
placidus Förster, 1860
prodigus Förster, 1860
punctiger Westwood, 1832
viridanus Förster, 1860
gastris (Boheman, 1834, *Periglyphus*)
brevicauda (Nees, 1834, *Siphonura*)
sericea (Nees, 1834, *Siphonura*)
variolosa (Nees, 1834, *Siphonura*)
viridiaenea (Ratzeburg, 1844, *Siphonura*)
fere-niger (De Stefani, 1898, *Torymus*)

##### Distribution

England, Ireland

##### Notes

According to Graham Stone (pers. comm.), DNA analysis suggests that *O. pomaceus* is an aggregate species, but it has not yet proved possible to satisfactorily distinguish morphological segregates.

### Family Perilampidae Förster, 1856

#### Chrysolampinae

Dalla Torre, 1898

#### Chrysolampus

Spinola, 1811

Elatus Walker, 1848
Lamprostylus Förster, 1856
Toxeumoides Girault, 1915
Paratoximopsis Girault, 1922

#### Chrysolampus rufitarsis

(Förster, 1859)

Elatus rufitarsis Förster, 1859

##### Distribution

England

#### Chrysolampus thenae

(Walker, 1848)

Elatus thenae Walker, 1848
obscurus (Walker, 1874, *Perilampus*)

##### Distribution

England, Wales

#### Perilampinae

Förster, 1856

#### Perilampus

Latreille, 1809

Perilampus Latreille, 1809
Cinipsillum Lamarck, 1817
Cynipsillum Agassiz, 1845
Afroperilampus Risbec, 1957
Bagdasar Argaman, 1990
Balintos Argaman, 1990
Bukbakas Argaman, 1990
Dekterek Argaman, 1990
Durgadas Argaman, 1990
Ecalibur Argaman, 1990
Fifirtiz Argaman, 1990
Fulaytar Argaman, 1990
Goyurfis Argaman, 1990
Ihrambek Argaman, 1990
Itonayis Argaman, 1990
Kekender Argaman, 1990
Lufarfar Argaman, 1990
Mivarhis Argaman, 1990
Naspoyar Argaman, 1990
Nilgator Argaman, 1990
Orarlar Argaman, 1990
Pondoros Argaman, 1990
Sicatang Argaman, 1990
Taltonos Argaman, 1990
Tiboras Argaman, 1990
Tondolos Argaman, 1990
Vadramas Argaman, 1990
Vaktaris Argaman, 1990
Yertatop Argaman, 1990
Zuglavas Argaman, 1990

#### Perilampus aeneus

(Rossius, 1790)

Chalcis aenea Rossius, 1790
italica (Fabricius, 1793, *Cynips*)

##### Distribution

England

##### Notes

**Figure 13.**
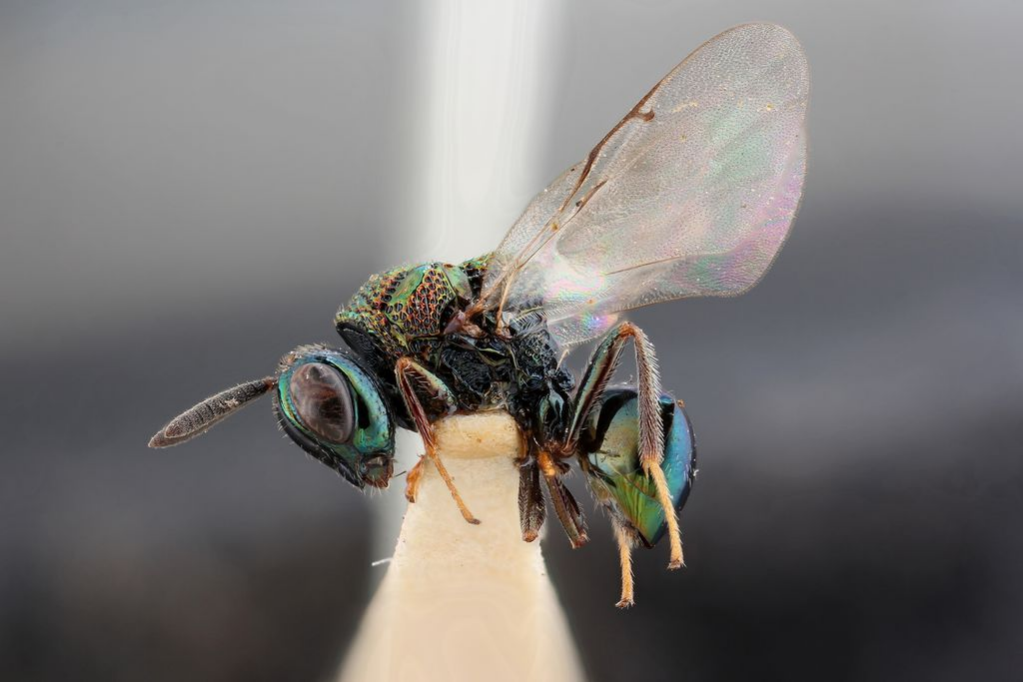
Perilampidae, Perilampinae: *Perilampus aeneus* (Rossius) female, BMNH specimen BMNH(E)953652 [/07/1931, Windsor Forest, Berkshire, England, coll. Donisthorpe] (photo K. McCormack)

See Fig. 13 for habitus

#### Perilampus aureoviridis

Walker, 1833

emarginatus Thomson, 1876
lacunosus Nikol'skaya, 1952

##### Distribution

England, Scotland, Wales

##### Notes

See Askew et al. (2006)

#### Perilampus cyaneus

(Fabricius, 1798)

Ichneumon cyaneus Fabricius, 1798

##### Notes

Recorded as British by Curtis (1829) but subsequently synonymised under *ruficornis*. Argaman (1991) resurrected *cyaneus* as a valid species but its presence on the British list requires confirmation.

#### Perilampus laevifrons

Dalman, 1822

inaequalis Förster, 1859
nigriventris Förster, 1859

##### Distribution

England

#### Perilampus micans

Dalman, 1820

auriceps Walker, 1833
femoralis Walker, 1833
lycti (Crawford, 1914, *Chrysolampus*)

##### Distribution

England

#### Perilampus polypori

Bouček, 1971

##### Distribution

England

#### Perilampus ruficornis

(Fabricius, 1793)

Cynips ruficornis Fabricius, 1793
violacea (Fabricius, 1804, *Diplolepis*)
pallipes Curtis, 1827
nigricornis Walker, 1833
scaber Nikol'skaya, 1952

##### Distribution

England

#### Perilampus tristis

Mayr, 1905

batavus Smits van Burgst, 1919
capitatus Smulyan, 1936
orcula Nikol'skaya, 1952

### Family Pteromalidae Dalman, 1820: Pteromalinae

#### Pteromalinae

Dalman, 1820

##### Notes

The family has been split into two groups (Pteromalinae and all other subfamilies) due to restrictions on the number of names that could be included as one group

#### Ablaxia

Delucchi 1957

#### Ablaxia anaxenor

(Walker, 1845)

Pteromalus anaxenor Walker, 1845

#### Ablaxia megachlora

(Walker, 1835)

Pteromalus megachlorus Walker, 1835

#### Ablaxia parviclava

(Thomson, 1878)

Etroxys parviclava Thomson, 1878

#### Ablaxia squamifera

(Thomson, 1878)

Etroxys squamifera Thomson, 1878

#### Ablaxia temporalis

Graham, 1969

##### Distribution

England

#### Acrocormus

Förster, 1856

#### Acrocormus semifasciatus

Thomson, 1878

#### Aggelma

Delucchi, 1956

#### Aggelma spiracularis

(Thomson, 1878)

Etroxys spiracularis Thomson, 1878

#### Anisopteromalus

Ruschka, 1912

APLASTOMORPHA Crawford, 1913

#### Anisopteromalus calandrae

(Howard, 1881)

Pteromalus calandrae Howard, 1881
oryzae (Cameron, 1891, *Pteromalus*)
vandinei (Tucker, 1910, *Meraporus*)
mollis Ruschka, 1912
australiensis (Girault, 1913, *Neocatolaccus*)
pratti (Crawford, 1913, *Aplastomorpha*)
medius (Masi, 1917, *Bruchobius*)
indicus (Ayyar & Mani, 1937, *Neocatolaccus*)
mamezophagus (Ishii & Nagasawa, 1942, *Neocatolaccus*)

#### Anogmoides

Askew, 1970

#### Anogmoides fumipennis

Askew, 1970

#### Anogmus

Förster, 1856

PLATYTHORAX Erdös, 1948

#### Anogmus strobilorum

(Thomson, 1878)

Roptrocerus strobilorum Thomson, 1878

#### Anogmus vala

(Walker, 1839)

Pteromalus vala Walker, 1839
specularis (Thomson, 1878, *Eutelus*)
strobicola (Ruschka, 1921, *Eutelus*)

#### Apelioma

Delucchi, 1956

#### Apelioma pteromalinum

(Thomson, 1878)

Dinotus pteromalinus Thomson, 1878

#### Apelioma restrictum

Graham, 1961

#### Apsilocera

Bouček, 1956

#### Apsilocera bramleyi

Graham, 1966

##### Distribution

England, Ireland

#### Arthrolytus

Thomson, 1878

#### Arthrolytus discoideus

(Nees, 1834)

Pteromalus discoideus Nees, 1834
artembares (Walker, 1839, *Pteromalus*)
punctatus (Thomson, 1878, *Pteromalus*)

#### Arthrolytus maculipennis

(Walker, 1835)

Pteromalus maculipennis Walker, 1835
cecidomyiae (Ashmead, 1897, *Holcaeus*)

##### Distribution

England, Wales

#### Arthrolytus ocellus

(Walker, 1834)

Eutelus ocellus Walker, 1834
albiscapus (Thomson, 1878, *Pteromalus*)

#### Atrichomalus

Graham, 1956

#### Atrichomalus trianellatus

Graham, 1956

#### Caenacis

Förster, 1856

#### Caenacis inflexa

(Ratzeburg, 1848)

Pteromalus inflexus Ratzeburg, 1848
punctulata (Thomson, 1878, *Etroxys*)
periclisti (Callan, 1944, *Habrocytus*)

##### Distribution

England

#### Caenacis lauta

(Walker, 1835)

Pteromalus lautus Walker, 1835
divisus (Walker, 1836, *Pteromalus*)
humilis (Förster, 1841, *Pteromalus*)
nervosus (Förster, 1841, *Pteromalus*)
strenuus (Förster, 1841, *Pteromalus*)
grandiclava (Thomson, 1878, *Etroxys*)

##### Distribution

England

#### Calliprymna

Graham, 1966

#### Calliprymna bisetosa

Graham, 1966

#### Callitula

Spinola, 1811

MICROMELUS Walker, 1833
BAEOTOMUS Förster, 1856
APTEROSEMOIDEA Girault, 1913
EURYDINOTELLA Girault, 1913
EURYDINOTELLEUS Girault, 1913
PSEUDOSPHEGIGASTERUS Girault, 1913
PTEROSEMOIDEA Girault, 1913
POLYCYSTOMYIA Dodd, 1915

#### Callitula bicolor

Spinola, 1811

rufomaculatus (Walker, 1833, *Micromelus*)
plagiatus (Nees, 1834, *Pteromalus*)

##### Distribution

England

#### Callitula ferrierei

Bouček, 1964

##### Distribution

England

#### Callitula pyrrhogaster

(Walker, 1833)

Micromelus pyrrhogaster Walker, 1833
mutilus (Förster, 1841, *Pteromalus*)

##### Distribution

England

#### Capellia

Delucchi, 1958

HYLOCOMUS Graham, 1959

#### Capellia cecidomyiae

(Ratzeburg, 1844)

Pteromalus cecidomyiae Ratzeburg, 1844
magnicornis (Thomson, 1878, *Metopon*)
strandi (Masi, 1911, *Pseudocatolaccus*)

#### Capellia orneus

(Walker, 1839)

Pteromalus orneus Walker, 1839
tychon (Walker, 1848, *Pteromalus*)

#### Catolaccus

Thomson, 1878

MERISOIDES Masi, 1911
HORTOBAGYIA Szelényi, 1981

#### Catolaccus ater

(Ratzeburg, 1852)

Pteromalus ater Ratzeburg, 1852
cavigena (Thomson, 1878, *Pteromalus*)

#### Cecidostiba

Thomson, 1878

RHIZOMALUS Bouček, 1972

#### Cecidostiba docimus

(Walker, 1839)

Pteromalus docimus Walker, 1839

##### Notes

R.R. Askew regards *C. jucundus* (Förster, 1841, *Pteromalus*) as a valid species, not occurring in Britain, contra Graham (1969a).

#### Cecidostiba fungosa

(Geoffroy, 1785)

Cynips fungosus Geoffroy, 1785
hilaris (Walker, 1836, *Pteromalus*)
anomalicornis (Förster, 1841, *Pteromalus*)
leucopezus (Ratzeburg, 1844, *Pteromalus*)
naubolus (Walker, 1845, *Pteromalus*)
meconotus (Ratzeburg, 1848, *Pteromalus*)
rugifrons (Thomson, 1878, *Etroxys*)
adana Askew, 1961

##### Distribution

England

#### Cecidostiba geganius

(Walker, 1848)

Gastrancistrus geganius Walker, 1848
cupreus (Bouček, 1972, *Rhizomalus*)

#### Cecidostiba semifascia

(Walker, 1835)

Pteromalus semifascia Walker, 1835
mundus (Walker, 1836, *Pteromalus*)
pronax (Walker, 1839, *Pteromalus*)
perditor (Förster, 1841, *Pteromalus*)
gallicus (Ratzeburg, 1848, *Pteromalus*)
truncata (Thomson, 1878, *Etroxys*)

##### Distribution

England

#### Cheiropachus

Westwood, 1829

PACHYCHIRUS Agassiz, 1848
TROPIDOGASTRA Ashmead, 1904

#### Cheiropachus quadrum

(Fabricius, 1787)

Ichneumon quadrum Fabricius, 1787
bimaculatus (Fabricius, 1793, *Ichneumon*)
bimaculatus (Swederus, 1795, *Pteromalus*)
maculipennis (Curtis, 1827, *Cleonymus*)
bicaliginosus (Ratzeburg, 1844, *Pteromalus*)
binaevius (Ratzeburg, 1844, *Pteromalus*)
binimbatus (Ratzeburg, 1844, *Pteromalus*)
binubeculatus (Ratzeburg, 1844, *Pteromalus*)
fraxini (Ratzeburg, 1844, *Pteromalus*)
intermedia (Förster, 1856, *Pachychirus*)
bimaculatus (Brèthes, 1916, *Habritus*)

##### Distribution

England

#### Chlorocytus

Graham, 1956

LEGOLASIA Hedqvist, 1974

#### Chlorocytus agropyri

Graham, 1965

#### Chlorocytus alticornis

Graham, 1984

##### Notes

Added by Graham (1984)

#### Chlorocytus breviscapus

Graham, 1965

#### Chlorocytus deschampsiae

Graham, 1965

#### Chlorocytus diversus

(Walker, 1836)

Pteromalus diversus Walker, 1836
rhytium (Walker, 1848, *Pteromalus*)
sybritia (Walker, 1848, *Pteromalus*)
laeviusculus (Thomson, 1878, *Etroxys*)

##### Distribution

England

#### Chlorocytus formosus

(Walker, 1835)

Pteromalus formosus Walker, 1835

#### Chlorocytus harmolitae

Bouček, 1957

#### Chlorocytus inchoatus

Graham, 1965

#### Chlorocytus longicauda

(Thomson, 1878)

Etroxys longicauda Thomson, 1878

##### Distribution

England

#### Chlorocytus phalaridis

Graham, 1965

##### Distribution

England

#### Chlorocytus pilosus

Graham, 1965

#### Chlorocytus planus

(Walker, 1834)

Eutelus planus Walker, 1834
pulchripes (Walker, 1836, *Pteromalus*)
aglaope (Walker, 1839, *Pteromalus*)

##### Distribution

England

#### Chlorocytus polichna

(Walker, 1848)

Trigonoderus polichna Walker, 1848
longiscapus Graham, 1965

#### Chlorocytus spicatus

(Walker, 1835)

Pteromalus spicatus Walker, 1835
filicornis (Walker, 1835, *Pteromalus*)
junceus (Walker, 1835, *Pteromalus*)
abila (Walker, 1839, *Pteromalus*)
simulans (Thomson, 1878, *Etroxys*)

#### Chlorocytus terminalis

(Walker, 1836)

Pteromalus terminalis Walker, 1836
laogore (Walker, 1839, *Pteromalus*)

#### Chlorocytus ultonicus

Graham, 1965

#### Coelopisthia

Förster, 1856

KRANOPHORUS Graham, 1956

##### Notes

Distribution data from Askew (1980).

#### Coelopisthia areolata

Askew, 1980

##### Distribution

England

##### Notes

Added by Askew (1980)

#### Coelopisthia caledonica

Askew, 1980

##### Distribution

Scotland

##### Notes

Added by Askew (1980)

#### Coelopisthia extenta

(Walker, 1835)

Pteromalus extentus Walker, 1835
catillus (Walker, 1835, *Pteromalus*)
rotundiventris (Zetterstedt, 1838, *Pteromalus*)
druso (Walker, 1839, *Pteromalus*)
breviramulus (Förster, 1841, *Pteromalus*)
multicarinatus (Förster, 1841, *Pteromalus*)

##### Distribution

England, Scotland, Wales

#### Coelopisthia pachycera

Masi, 1924

##### Distribution

England

#### Conomorium

Masi, 1924

#### Conomorium amplum

(Walker, 1835)

Pteromalus amplus Walker, 1835
eremita (Förster, 1841, *Pteromalus*)
scopas (Walker, 1849, *Pteromalus*)

#### Conomorium patulum

(Walker, 1835)

Pteromalus patulus Walker, 1835
vitripennis (Thomson, 1878, *Pteromalus*)

#### Coruna

Walker, 1833

PACHYCREPIS Förster, 1856

#### Coruna clavata

Walker, 1833

aphidivorus (Förster, 1841, *Pteromalus*)
castigator (Rondani, 1848, *Pteromalus*)
segmentarius (Förster, 1841, *Pteromalus*)
hierocles (Walker, 1848, *Gastrancistrus*)
dubia (Buckton, 1879, *Coryna*)

##### Distribution

England, Wales

#### Cratomus

Dalman, 1820

#### Cratomus megacephalus

(Fabricius, 1793)

Cynips megacephala Fabricius, 1793
nigripes Walker, 1833
macrocephalus (Förster, 1841, *Pteromalus*)
megalocephalus (Schulz, 1906, *Caratomus*)

#### Cryptoprymna

Förster, 1856

PROSODES Walker, 1833
CRYPTOPRYMNUS Thomson, 1878
POLYCYSTELOMORPHA Girault, 1915

#### Cryptoprymna atra

(Walker, 1833)

Prosodes ater Walker, 1833
lugubris (Nees, 1834, *Chrysolampus*)
cavigena Thomson, 1878

##### Distribution

England, Scotland

#### Cryptoprymna paludicola

Askew, 1991

##### Distribution

England

##### Notes

Added by Askew (1991b)Askew (1991a)

#### Cyclogastrella clypealis

Bouček, 1965

##### Distribution

England

#### Cyclogastrella flavius

(Walker, 1839)

Pteromalus flavius Walker, 1839
cepio (Walker, 1848, *Pteromalus*)
heterotomus (Thomson, 1878, *Metopon*)

#### Cyclogastrella simplex

(Walker, 1834)

Ormocerus simplex Walker, 1834
deplanatus (Nees, 1834, *Pteromalus*)
domesticus (Walker, 1835, *Pteromalus*)
artemon (Walker, 1839, *Pteromalus*)
merope (Walker, 1839, *Pteromalus*)
acco (Walker, 1848, *Pteromalus*)
androbius (Walker, 1848, *Pteromalus*)
phasis (Walker, 1848, *Pteromalus*)
quercina Bukovskii, 1938

#### Cyrtogaster

Walker, 1833

POLYCYSTUS Westwood, 1839
DICORMUS Förster, 1841
HATIA Risbec, 1955

##### Notes

Doubtfully placed species of Cyrtogaster:

[*poesos* Walker,1848 nom. Dub.] - type lost; a possible synonym of *vulgaris*.

#### Cyrtogaster britteni

Askew, 1965

#### Cyrtogaster clavicornis

Walker, 1833

obscura Walker, 1833
matthewsii (Westwood, 1839, *Polycystus*)
scapularis (Thomson, 1878, *Polycystus*)

##### Distribution

England

#### Cyrtogaster vulgaris

Walker, 1833

cingulipes Walker, 1833
rufipes Walker, 1833
tenuis Walker, 1833
thoracica Walker, 1833
viridiaeneus (Nees, 1834, *Chrysolampus*)
aquisgranensis (Förster, 1841, *Dicormus*)
acarnas (Walker, 1848, *Lamprotatus*)
biglobus Förster, 1861

##### Distribution

England, Scotland

#### Dibrachoides

Kurdjumov, 1913

#### Dibrachoides cionobius

Graham, 1969

#### Dibrachoides dynastes

(Förster, 1841)

Pteromalus dynastes Förster, 1841
transversus (Förster, 1841, *Pteromalus*)
acutus (Thomson, 1878, *Pteromalus*)

#### Dibrachys

Förster, 1856

COELOPISTHOIDEA Gahan, 1913

##### Notes

Taxonomy and distribution data for some species from Peters and Baur (2011).

#### Dibrachys affinis

Masi, 1907

#### Dibrachys fuscicornis

(Walker, 1836)

Pteromalus fuscicornis Walker, 1836
saltans (Ratzeburg, 1852, *Pteromalus*)
cladiae (Gahan, 1913, *Coelopisthoidea*)

#### Dibrachys lignicola

Graham, 1969

##### Distribution

England, Ireland

#### Dibrachys microgastri

(Bouché, 1834)

Diplolepis microgastri Bouché, 1834
microgasteris (Nees, 1834, *Pteromalus*)
cavus (Walker, 1835, *Pteromalus*)
decedens (Walker, 1835, *Pteromalus*)
perversus (Walker, 1835, *Pteromalus*)
albinervis (Ratzeburg, 1844, *Pteromalus*)
boucheanus (Ratzeburg, 1844, *Pteromalus*)
tenuis (Ratzeburg, 1844, *Pteromalus*)
zelleri (Ratzeburg, 1848, *Pteromalus*)
vesparum (Ratzeburg, 1852, *Pteromalus*)
clisiocampae (Fitch, 1856, *Cleonymus*)
boarmiae (Walker, 1863, *Pteromalus*)
nigrocyaneus (Norton, 1869, *Cheiropachus*)
cereanus (Rondani, 1876, *Eupelmus*)
gelechiae (Webster, 1883, *Pteromalus*)
chionobae (Howard, 1889, *Pteromalus*)
apatelae (Ashmead, 1893, *Arthrolyus*)
pimplae (Ashmead, 1894, *Arthrolyus*)
truyilloi (Blanchard, 1938, *Trichomalus*)
elegans (Szelényi, 1981, *Tritneptis*)

##### Distribution

England

#### Dibrachys verovesparum

Peters & Baur, 2011

##### Distribution

England

##### Notes

Added by Peters and Baur (2011)

#### Diglochis

Förster, 1856

TRICHOGLENUS Thomson, 1878

#### Diglochis sylvicola

(Walker, 1835)

Pteromalus sylvicola Walker, 1835
complanatus (Ratzeburg, 1844, *Pteromalus*)
hybomitri Dzhanokmen, 1979

##### Distribution

England

#### Dimachus

Thomson, 1878

#### Dimachus cingulum

(Nees, 1834)

Pteromalus cingulum Nees, 1834
discolor (Walker, 1836, *Pteromalus*)
emathion (Walker, 1839, *Pteromalus*)
drepanon (Walker, 1848, *Pteromalus*)

#### Dinarmus

Thomson, 1878

BRUCHOBIUS Ashmead, 1904
METASTENOIDES Girault, 1915
OEDAULE Waterston, 1922
SPHAERAKIS Masi, 1924

#### Dinarmus acutus

(Thomson, 1878)

Dimachus acutus Thomson, 1878
robustus (Walker, 1847, *Pteromalus*)
kollari (Dalla Torre, 1898, *Pteromalus*)
mayri (Masi, 1924, *Sphaerakis*)
arachnephaga (Risbec, 1951, *Bruchobius*)
bifoveolatus Delucchi, 1956

#### Dinotiscus

Ghesquière, 1946

DINOTUS Förster, 1856 preocc.

#### Dinotiscus aponius

(Walker, 1848)

Heteroxys aponius Walker, 1848
capitatus (Ratzeburg, 1844, *Pteromalus*)
bidentulus (Thomson, 1878, *Dinotus*)

##### Distribution

England

#### Dinotiscus colon

(Linnaeus, 1758)

Sphex colon Linnaeus, 1758
calcaratus (Thomson, 1878, *Dinotus*)

##### Distribution

Scotland

#### Dinotiscus eupterus

(Walker, 1836)

Pteromalus eupterus Walker, 1836
dimidiatus (Walker, 1836, *Pteromalus*)
capitatus (Förster, 1841, *Pteromalus*)
lanceolatus (Ratzeburg, 1848, *Pteromalus*)
clypealis (Thomson, 1878, *Dinotus*)
acutus (Provancher, 1887, *Dinotus*)
polygraphi (Ashmead, 1894, *Cecidostiba*)
ashmeadi (Crawford, 1912, *Cecidostiba*)
pityogenis (Ishii, 1938, *Uriella*)

##### Distribution

England

#### Dinotoides

Bouček, 1957

#### Dinotoides tenebricus

(Walker, 1834)

Amblymerus tenebricus Walker, 1834
ariovistus (Walker, 1839, *Pteromalus*)
carcinus (Walker, 1839, *Pteromalus*)
antho (Walker, 1845, *Pteromalus*)
bicalcaratus Bouček, 1957

##### Distribution

England

#### Dirhicnus

Thomson, 1878

#### Dirhicnus ramealis

(Nees, 1834)

Pteromalus ramealis Nees, 1834
gonatas (Walker, 1839, *Pteromalus*)
pirus (Walker, 1839, *Pteromalus*)
sisenna (Walker, 1839, *Pteromalus*)
toxicrate (Walker, 1839, *Pteromalus*)
insidiator (Förster, 1841, *Pteromalus*)
separatus (Förster, 1841, *Pteromalus*)
bubaris (Walker, 1845, *Pteromalus*)
cercides (Walker, 1845, *Pteromalus*)
nestocles (Walker, 1845, *Pteromalus*)
gallonius (Walker, 1848, *Pteromalus*)
subcoeruleus (Thomson, 1878, *Metopon*)

##### Distribution

England

#### Endomychobius

Ashmead, 1896

#### Endomychobius endomychi

(Walker, 1836)

Pteromalus endomychi Walker, 1836
mazaces (Walker, 1844, *Pteromalus*)

##### Distribution

England

#### Erdoesia

Bouček, 1957

#### Erdoesia tessellata

Bouček, 1957

#### Erythromalus

Graham, 1956

#### Erythromalus nubilipennis

(Walker, 1835)

Pteromalus nubilipennis Walker, 1835
Erythromalus nubilipennis ?*faustina* (Walker, 1839, *Pteromalus*)

##### Distribution

England

#### Erythromalus rufiventris

(Walker, 1835)

Pteromalus rufiventris Walker, 1835
empoclus (Walker, 1839, *Pteromalus*)

#### Eulonchetron

Graham, 1966

#### Eulonchetron torymoides

(Thomson, 1878)

Etroxys torymoides Thomson, 1878
canadensis (Girault, 1917, *Habrocytus*)
giraulti (Peck, 1951, *Habrocytus*)
scalprum (Askew, 1962, *Lonchetron*)

#### Eumacepolus

Graham, 1957

#### Eumacepolus obscurior

Graham, 1961

##### Distribution

England

#### Eumacepolus pulcher

Graham, 1961

##### Distribution

Ireland

#### Euneura

Walker, 1844

HYPSICAMARA Förster, 1856
GYGAXIA Delucchi, 1955

#### Euneura lachni

(Ashmead, 1887)

Pachycrepis lachni Ashmead, 1887

##### Distribution

England

##### Notes

BMNH, det. Polaszek, added here

#### Euneura sopolis

(Walker, 1840)

Miscogaster sopolis Walker, 1840
augarus Walker, 1844
ratzeburgi (Reinhard, 1859, *Hypsicamara*)

##### Distribution

England

#### Gastracanthus

Westwood, 1833

HETROXYS Westwood, 1833
PHOTISMUS Thomson, 1878
HETEROXYS Dalla Torre, 1898
HEBESTEPHUS Kamijo, 1960
CLEOBLABENA Szelényi, 1981

#### Gastracanthus pulcherrimus

Westwood, 1833

macromerus (Walker, 1836, *Pteromalus*)
elegans (Walker, 1836, *Trigonoderus*)
transversus (Förster, 1841, *Cleonymus*)
nubilosus (Thomson, 1878, *Photismus*)
gracilis (Szelényi, 1981, *Cleoblabena*)

##### Distribution

England

#### Gbelcia

Bouček, 1961

NASONIELLA Szelényi, 1982

#### Gbelcia crassiceps

Bouček, 1961

conspicua (Szelényi, 1982, *Nasoniella*)

#### Gyrinophagus

Ruschka, 1914

#### Gyrinophagus aper

(Walker, 1839)

Pteromalus aper Walker, 1839
marginatus (Thomson, 1878, *Isocyrtus*)

#### Habritys

Thomson, 1878

#### Habritys brevicornis

(Ratzeburg, 1844)

Pteromalus brevicornis Ratzeburg, 1844
pannewitzii (Ratzeburg, 1852, *Pteromalus*)

#### Hemitrichus

Thomson, 1878

URIELLA Ashmead, 1896

#### Hemitrichus oxygaster

Bouček, 1965

##### Distribution

England

##### Notes

BMNH, det. Clarke, added here

#### Hemitrichus seniculus

(Nees, 1834)

Pteromalus seniculus Nees, 1834
phylacis (Walker, 1848, *Pteromalus*)
rufipes (Thomson, 1878, *Dimachus*)
rufipes (Ashmead, 1896, *Uriella*)
assimilis Masi, 1922

##### Distribution

England

#### Heteroprymna

Graham, 1956

#### Heteroprymna longicornis

(Walker, 1835)

Pteromalus longicornis Walker, 1835
camma (Walker, 1848, *Pteromalus*)

#### Hobbya

Delucchi, 1957

#### Hobbya stenonota

(Ratzeburg, 1848)

Pteromalus stenonotus Ratzeburg, 1848
collaris (Thomson, 1878, *Etroxys*)
kollari Askew, 1959

##### Distribution

England

#### Holcaeus

Thomson, 1878

CRICELLIUS Thomson, 1878
DIBRACHELLA Bouček, 1954

#### Holcaeus calligetus

(Walker, 1839)

Pteromalus calligetus Walker, 1839

##### Distribution

England

#### Holcaeus compressus

(Walker, 1836)

Pteromalus compressus Walker, 1836
fuscescens (Walker, 1836, *Pteromalus*)
Holcaeus compressus ?*ection* (Walker, 1845, *Pteromalus*)
hyrtacus (Walker, 1848, *Pteromalus*)
elongatus (Thomson, 1878, *Etroxys*)

##### Distribution

England

#### Holcaeus gorgasus

(Walker, 1839)

Pteromalus gorgasus Walker, 1839
dichrous (Thomson, 1878, *Etroxys*)

##### Distribution

England

#### Holcaeus gracilis

(Walker, 1836)

Pteromalus gracilis Walker, 1836
Holcaeus gracilis ?*alcman* (Walker, 1839, *Pteromalus*)

##### Distribution

Wales

#### Holcaeus repandus

(Graham, 1969)

Cricellius repandus Graham, 1969

#### Holcaeus stenogaster

(Walker, 1836)

Pteromalus stenogaster Walker, 1836
Holcaeus stenogaster ?*cabades* (Walker, 1839, *Pteromalus*)
Holcaeus stenogaster ?*styrus* (Walker, 1839, *Pteromalus*)
Holcaeus stenogaster ?*amnisos* (Walker, 1848, *Gastrancistrus*)
longicauda (Thomson, 1878, *Etroxys*)

##### Distribution

England, Scotland

#### Holcaeus stylatus

Graham, 1969

##### Distribution

England

#### Holcaeus varro

(Walker, 1840)

Pteromalus varro Walker, 1840

#### Homoporus

Thomson, 1878

PHAENACRA Förster, 1878
PARAPTEROMALUS Ashmead, 1904
MERISOPORUS Masi, 1924

#### Homoporus apharetus

(Walker, 1839)

Pteromalus apharetus Walker, 1839
flaviscapus (Thomson, 1878, *Merisus*)

#### Homoporus arestor

(Walker, 1848)

Pteromalus arestor Walker, 1848
chlorogaster (Thomson, 1878, *Merisus*)

#### Homoporus destructor

(Say, 1817)

Ceraphron destructor Say, 1817
intermedius (Lindeman, 1887, *Merisus*)

##### Distribution

England

#### Homoporus febriculosus

(Girault, 1917)

Merisus febriculosus Girault, 1917
filicornis Erdös, 1953
templarius Erdös, 1970

##### Distribution

England, Scotland

#### Homoporus fulviventris

(Walker, 1835)

Pteromalus fulviventris Walker, 1835
bicolor (Förster, 1841, *Pteromalus*) preocc.
bicoloratus (Dalla Torre, 1898, *Pteromalus*)
clavicornis Erdös, 1953

#### Homoporus gibbiscuta

(Thomson, 1878)

Merisus gibbiscuta Thomson, 1878

#### Homoporus luniger

(Nees, 1834)

Pteromalus luniger Nees, 1834
tricolor (Walker, 1835, *Pteromalus*)
zonaras (Walker, 1839, *Pteromalus*)
nubigera (Förster, 1878, *Phaenacra*)

##### Distribution

England, Wales

#### Homoporus nypsius

(Walker, 1839)

Pteromalus nypsius Walker, 1839
chalcidiphagus (Walsh & Riley, 1869, *Semiotellus*)
crassinervis (Thomson, 1878, *Merisus*)

##### Distribution

England

#### Homoporus semiluteus

(Walker, 1872)

Pteromalus semiluteus Walker, 1872
bicolor (Erdös, 1955, *Picroscytus*)
robustus Delucchi, 1957
bicolorus Erdös, 1970

##### Distribution

England

##### Notes

Listed as a British species by Bouček and Graham (1978) presumably on the basis of a specimen in BMNH, det. Z. Bouček.

#### Homoporus subniger

(Walker, 1835)

Pteromalus subniger Walker, 1835
chalcomelas (Walker, 1836, *Pteromalus*)
kurdjumovi Szelényi, 1956
danuvianus Delucchi, 1957

##### Distribution

England, Scotland, Wales

#### Isocyrtus

Walker, 1833

KODYSIA Bouček, 1954

#### Isocyrtus laetus

Walker, 1833

contractus (Nees, 1834, *Chrysolampus*)
tibialis (Bouček, 1954, *Kodysia*)

##### Distribution

England, Scotland, Wales

#### Janssoniella

Kerrich, 1957

#### Janssoniella ambigua

Graham, 1969

#### Janssoniella caudata

Kerrich, 1957

##### Distribution

England

#### Kaleva

Graham, 1957

#### Kaleva corynocera

Graham, 1957

#### Lampoterma

Graham, 1956

#### Lampoterma bianellatum

Graham, 1969

#### Lampoterma viride

(Thomson, 1876)

Metastenus viridis Thomson, 1876

##### Distribution

Scotland

#### Lariophagus

Crawford, 1909

URIELLOMYIA Girault, 1915

##### Notes

Graham (1969a) also lists an apparently undescribed species, collected in England, that remains undescribed.

#### Lariophagus distinguendus

(Förster, 1841)

Pteromalus distinguendus Förster, 1841
calamis (Walker, 1849, *Pteromalus*)
oryzinus (Rondani, 1874, *Pteromalus*)
utibilis (Tucker, 1910, *Meraporus*)
resoluta (Girault, 1915, *Uriellomyia*)
miltoni (Girault, 1929, *Nasonia*)

#### Lariophagus rufipes

Hedqvist, 1978

#### Leptomeraporus

Graham, 1957

#### Leptomeraporus nicaee

(Walker, 1839)

Miscogaster nicaee Walker, 1839
zagreus (Walker, 1848, *Pteromalus*)
tenuicornis (Graham, 1957, *Meraporus*)

##### Distribution

England, Wales

#### Lonchetron

Graham, 1956

#### Lonchetron fennicum

Graham, 1956

##### Distribution

England, Wales

##### Notes

BMNH, det. Bouček, added here

#### Meraporus

Walker, 1834

PARMICROMELUS Girault, 1917

#### Meraporus graminicola

Walker, 1834

hebes (Walker, 1834, *Amblymerus*)
iners (Walker, 1834, *Amblymerus*)
modestus (Walker, 1834, *Amblymerus*)
temperatus (Walker, 1834, *Amblymerus*)
alatus Walker, 1834
tenuiscapus (Förster, 1841, *Pteromalus*)
allutius (Walker, 1848, *Pteromalus*)
gigon (Walker, 1848, *Pteromalus*)
myle (Walker, 1848, *Pteromalus*)
micropterus (Förster, 1861, *Pteromalus*)
pulex (Förster, 1861, *Pteromalus*)

##### Distribution

England, Wales

#### Merisus

Walker, 1834

#### Merisus splendidus

Walker, 1834

spinolae (Förster, 1841, *Pteromalus*)
acutangulus Thomson, 1878

##### Distribution

England

#### Mesopolobus

Westwood, 1833

PLATYMESOPUS Westwood, 1833
AMBLYMERUS Walker, 1834
EUTELUS Walker, 1834
PLATYTERMA Walker, 1834
XENOCREPIS Förster, 1856
PTEROMALODES Dahlbom, 1857
SELITRICHUS Rondani, 1877
ASEMANTUS Förster, 1878
DISEMA Förster, 1878
PLATYTERMUS Thomson, 1878
SYNTOMOCERA Förster, 1878
URIELLOIDES Girault, 1913
ZACALOCHLORA Crawford, 1913
PARANOGMUS Girault & Dodd, 1915
ANOGMOIDEA Girault, 1924
BAEOPONERUS Masi, 1924
EUAMBLYMERUS Hincks, 1944
DISEMISCA Ghesquière, 1946
SYNTOMOCERELLA Ghesquière, 1946
AHLBERGIELLA Rosen, 1955
STUROVIA Bouček, 1961
ISOPTRYNEA Szelényi, 1982

##### Notes

Doubtfully placed species of *Mesopolobus*:

[*comptus* (Walker, 1834, *Platyterma*) nom. dub.]

#### Mesopolobus aequus

(Walker, 1834)

Eutelus aequus Walker, 1834
contractus (Walker, 1835, *Pteromalus*)
purpureus (Walker, 1835, *Pteromalus*)
leogoras (Walker, 1839, *Pteromalus*)
odites (Walker, 1845, *Pteromalus*)
temesa (Walker, 1848, *Pteromalus*)
purus (Walker, 1872, *Metastenus*)
decipiens (Thomson, 1878, *Eutelus*)
oviphaga (Ahlberg, 1925, *Mormoniella*)

##### Distribution

England, Scotland

#### Mesopolobus agropyricola

Rosen, 1960

#### Mesopolobus albitarsus

(Walker, 1834)

Amblymerus albitarsus Walker, 1834
corion (Walker, 1848, *Pteromalus*)
pedunculi (Thomson, 1878, *Eutelus*)

##### Distribution

England

#### Mesopolobus amaenus

(Walker, 1834)

Amblymerus amaenus Walker, 1834
nanus (Walker, 1834, *Amblymerus*)
catenatus (Walker, 1834, *Eutelus*)
dilectus (Walker, 1834, *Eutelus*)
eximius (Walker, 1834, *Eutelus*)
immaculatus (Walker, 1834, *Eutelus*)
remotum (Walker, 1834, *Platyterma*)
Mesopolobus amaenus ?*lebene* (Walker, 1848, *Pteromalus*)
cecidomyinus (Rondani, 1877, *Eupelmus*)
circinantis (Rondani, 1877, *Eupelmus*)
collaris (Thomson, 1878, *Eutelus*)

#### Mesopolobus anogmoides

Graham, 1969

#### Mesopolobus aspilus

(Walker, 1835)

Pteromalus aspilus Walker, 1835
elongatus (Thomson, 1878, *Eutelus*)

##### Distribution

England

#### Mesopolobus citrinus

(Ratzeburg, 1852)

Pteromalus citrinus Ratzeburg, 1852

#### Mesopolobus clavicornis

(Förster, 1878)

Syntomocera clavicornis Förster, 1878

##### Notes

Identification uncertain (Graham 1969a).

#### Mesopolobus diffinis

(Walker, 1834)

Eutelus diffinis Walker, 1834
fulvipes (Walker, 1834, *Amblymerus*)
latus (Walker, 1834, *Amblymerus*)
linearis (Walker, 1834, *Amblymerus*)
pusillus (Walker, 1834, *Amblymerus*)
stenomerus (Walker, 1834, *Amblymerus*)
pygmaeus (Walker, 1834, *Eutelus*)
vagans (Walker, 1834, *Eutelus*)
aenicus (Walker, *Pteromalus*) unavailable
pygmaeus (Walker, 1834, *Pteromalus*)
exilis (Walker, 1836, *Pteromalus*)
leuce (Walker, 1848, *Pteromalus*)

##### Distribution

England, Scotland, Wales, Isle of Man

#### Mesopolobus dubius

(Walker, 1834)

Amblymerus dubius Walker, 1834
fulvipennis (Walker, 1834, *Amblymerus*)
ruralis (Walker, 1834, *Amblymerus*)
trossulus (Walker, 1834, *Amblymerus*)
truncatellus (Walker, 1834, *Amblymerus*)
validus (Walker, 1834, *Amblymerus*)
signatus (Walker, 1834, *Eutelus*)
ovatus (Nees, 1834, *Pteromalus*)
pinguis (Walker, 1835, *Pteromalus*)
luteicornis (Fonscolombe, 1840, *Cinips*)

##### Distribution

England

#### Mesopolobus fasciiventris

Westwood, 1833

flavipes (Walker, 1834, *Eutelus*)
fulvicornis (Walker, 1834, *Eutelus*)
fasciculatus (Förster, 1841, *Pteromalus*)
saxesenii (Ratzeburg, 1844, *Pteromalus*)
trochilus (Ratzeburg, 1844, *Pteromalus*)

##### Distribution

England

#### Mesopolobus fuscipes

(Walker, 1834)

Amblymerus fuscipes Walker, 1834
humilis (Walker, 1834, *Amblymerus*)
erichsonii (Ratzeburg, 1844, *Platymesopus*)

##### Distribution

England

#### Mesopolobus gemellus

Baur & Muller, 2007

##### Distribution

England

##### Notes

Baur et al. (2007)dded by Baur et al. (2007)

#### Mesopolobus graminum

(Hardh, 1950)

Amblymerus graminum Hardh, 1950

#### Mesopolobus incultus

(Walker, 1834)

Platyterma incultum Walker, 1834
stupidus (Walker, 1834, *Amblymerus*)
femorale (Walker, 1834, *Platyterma*)
ergias (Walker, 1839, *Pteromalus*)
leodocus (Walker, 1839, *Pteromalus*)
amyntor (Walker, 1845, *Pteromalus*)
urgo (Walker, 1845, *Pteromalus*)
belesis (Walker, 1848, *Pteromalus*)
berecynthos (Walker, 1848, *Pteromalus*)
lissos (Walker, 1848, *Pteromalus*)
clavicornis (Walker, 1874, *Pteromalus*)
crassicornis (Thomson, 1878, *Eutelus*)

#### Mesopolobus juniperinus

Rosen, 1958

##### Distribution

England, Scotland

##### Notes

Added by Springate and Noyes (1990)

#### Mesopolobus laticornis

(Walker, 1834)

Platyterma laticorne Walker, 1834

#### Mesopolobus longicollis

Graham, 1969

#### Mesopolobus mediterraneus

(Mayr, 1903)

Eutelus mediterraneus Mayr, 1903

#### Mesopolobus mesostenus

Graham, 1969

#### Mesopolobus morys

(Walker, 1848)

Pteromalus morys Walker, 1848
pallipes (Förster, 1878, *Disema*)
ceutorhynchi (Rondani, 1872, *Encyrtus*)
pura (Mayr, 1904, *Xenocrepis*)

##### Distribution

England

#### Mesopolobus nobilis

(Walker, 1834)

Platyterma nobile Walker, 1834
decorum (Walker, 1834, *Platyterma*)

##### Distribution

England

#### Mesopolobus phragmitis

(Erdös, 1957)

Eutelus phragmitis Erdös, 1957

##### Distribution

England

#### Mesopolobus pinus

Hussey, 1960

#### Mesopolobus prasinus

(Walker, 1834)

Platyterma prasinum Walker, 1834
amphibolus (Förster, 1878, *Asemantus*)

#### Mesopolobus pseudofuscipes

Rosen, 1958

##### Distribution

Scotland

#### Mesopolobus pseudolaticornis

Rosen, 1966

#### Mesopolobus rhabdophagae

(Graham, 1957)

Platymesopus rhabdophagae Graham, 1957

##### Distribution

Scotland

#### Mesopolobus semiclavatus

(Ratzeburg, 1848)

Pteromalus semiclavatus Ratzeburg, 1848

##### Distribution

England

#### Mesopolobus sericeus

(Forster, 1770)

Cynips sericeus Forster, 1770
foliaceus (Geoffroy, 1785, *Cynips*)
minuta (Geoffroy, 1785, *Cynips*)
minutus (Geoffroy, 1785, *Cynips*)
jucundus (Walker, 1834, *Eutelus*)
fuscicornis (Fonscolombe, 1840, *Cinips*)
simplex (Thomson, 1878, *Eutelus*)

##### Distribution

England, Scotland

#### Mesopolobus spermotrophus

Hussey, 1960

##### Distribution

Scotland

#### Mesopolobus subfumatus

(Ratzeburg, 1852)

Pteromalus subfumatus Ratzeburg, 1852
punctiger (Thomson, 1878, *Eutelus*)
ecksteini (Wolff, 1916, *Platyterma*)
matsukemushii (Matsumura, 1926, *Pteromalus*)
tabatae (Ishii, 1938, *Eutelus*)

##### Distribution

England

##### Notes

Added by Askew (1992c)

#### Mesopolobus tarsatus

(Nees, 1834)

Pteromalus tarsatus Nees, 1834
squamifer (Thomson, 1878, *Eutelus*)
tenuicornis (Bouček, 1961, *Sturovia*)

#### Mesopolobus teliformis

(Walker, 1834)

Platyterma teliforme Walker, 1834
cincticorne (Walker, 1834, *Platyterma*)
terminale (Walker, 1834, *Platyterma*)
placidus (Förster, 1841, *Pteromalus*)
brevicornis (Thomson, 1878, *Eutelus*)
suavis (Dalla Torre, 1898, *Pteromalus*)

#### Mesopolobus tibialis

(Westwood, 1833)

Platymesopus tibialis Westwood, 1833
bicolor (Walker, 1834, *Eutelus*)
platycerus (Walker, 1834, *Eutelus*)
platynotus (Walker, 1834, *Eutelus*)
sobrinus (Walker, 1834, *Eutelus*)
anticus (Walker, 1836, *Pteromalus*)
rusticus (Förster, 1841, *Pteromalus*)
sodalis (Förster, 1841, *Pteromalus*)
westwoodii (Ratzeburg, 1844, *Platymesopus*)
platymesopus (Reinhard, *Pteromalus*) unavailable name
apicalis (Westwood, 1882, *Platymesopus*)
rusticanus (Dalla Torre, 1898, *Pteromalus*)
caconymus (Schulz, 1906, *Eutelus*)

##### Distribution

England

#### Mesopolobus trasullus

(Walker, 1839)

Ormocerus trasullus Walker, 1839
roseni Graham, 1984

#### Mesopolobus xanthocerus

(Thomson, 1878)

Eutelus xanthocerus Thomson, 1878

##### Distribution

England

#### Metacolus

Förster, 1856

#### Metacolus azureus

(Ratzeburg, 1844)

Pteromalus azureus Ratzeburg, 1844
varicolor (Förster, 1878, *Pterosema*)
aulloi Mercet, 1926

##### Distribution

England

##### Notes

Added by Askew (1992c)

#### Metacolus unifasciatus

Förster, 1856

beesoni (Mani & Kaul, 1973, *Zapachia*)

#### Metastenus

Walker, 1834

SCYMNOPHAGUS Ashmead, 1904
TRIPOLYCYSTUS Dodd, 1915

#### Metastenus concinnus

Walker, 1834

mesnili (Ferrière, 1954, *Scymnophagus*)

#### Mokrzeckia

Mokrzecki, 1934

BEIERINA Delucchi, 1958

#### Mokrzeckia obscura

Graham, 1969

#### Muscidifurax

Girault & Sanders, 1910

SMEAGOLIA Hedqvist, 1973

#### Muscidifurax raptor

Girault & Sanders, 1910

perplexa (Hedqvist, 1973, *Smeagolia*)

##### Distribution

England

#### Nasonia

Ashmead, 1904

MORMONIELLA Ashmead, 1904

#### Nasonia vitripennis

(Walker, 1836)

Pteromalus vitripennis Walker, 1836
muscarum (Hartig, 1838, *Pteromalus*)
abnormis (Boheman, 1858, *Pteromalus*)
insuetus (Walker, 1872, *Stictonotus*)
pallinervosus (Walker, 1872, *Dicyclus*)
pallidinervosus (Dalla Torre, 1898, *Dicyclus*)
brevicornis (Ashmead, 1904, *Mormoniella*)
brevicornis Ashmead, 1904
erausquinii (Brèthes, 1913, *Platymesopus*)

##### Distribution

England

#### Nephelomalus

Graham, 1956

#### Nephelomalus conspersus

(Walker, 1835)

Pteromalus conspersus Walker, 1835

#### Norbanus

Walker, 1843

#### Norbanus scabriculus

(Nees, 1834)

Pteromalus scabriculus Nees, 1834

##### Distribution

England

##### Notes

Added by Rizzo and Mitroiu (2010)

#### Notoglyptus

Masi, 1917

#### Notoglyptus scutellaris

(Dodd & Girault, 1915)

Merismus scutellaris Dodd & Girault, 1915

##### Distribution

England

##### Notes

BMNH, det. Bouček, added here

#### Pachycrepoideus

Ashmead, 1904

#### Pachycrepoideus vindemmiae

(Rondani, 1875)

Pteromalus vindemmiae Rondani, 1875
dubius Ashmead, 1904
dissimilis (Girault & Dodd, 1915, *Toxeumella*)
nigra (Girault, 1915, *Toxeumopsis*)
drosophilae (Dodd, 1917, *Pterosemoidea*)
crassinervis (Bouček, 1954, *Anisopteromalia*)
elongata Delucchi, 1955

#### Pachyneuron

Walker, 1833

PACHYNEVRUM Agassiz, 1848
SERIMUS Brèthes, 1913
NEPACHYNEURON Girault, 1917
PROPACHYNEURONIA Girault, 1917
EUPACHYNEURON Blanchard, 1948
ATRICHOPTILUS Delucchi, 1955

#### Pachyneuron aphidis

(Bouché, 1834)

Diplolepis aphidis Bouché, 1834
minutissimus (Förster, 1841, *Pteromalus*)
pruni Walker, 1850
siphonophorae (Ashmead, 1886, *Encyrtus*)
aphidivorum Ashmead, 1887
maidaphidis Ashmead, 1888
micans Howard, 1890
gifuensis Ashmead, 1904
argentinus (Brèthes, 1913, *Serimus*)
ferrierei Mani, 1939
lali Mani, 1939
bosqi (Blanchard, 1948, *Eupachyneuron*)
triarticulata Mani & Saraswat, 1974

#### Pachyneuron formosum

Walker, 1833

amoenus (Förster, 1841, *Pteromalus*)
incubator (Förster, 1841, *Pteromalus*)

##### Distribution

England

#### Pachyneuron groenlandicum

(Holmgren, 1872)

Pteromalus groenlandicus Holmgren, 1872
mitsukurii Ashmead, 1904
karnalensis Mani, 1939
coeruleum Delucchi, 1955
umbratum Delucchi, 1955
bakrotus Mani & Saraswat, 1974

##### Distribution

England

#### Pachyneuron leucopiscida

Mani, 1939

cremifaniae Delucchi, 1953

#### Pachyneuron muscarum

(Linnaeus, 1758)

Ichneumon muscarum Linnaeus, 1758
coccorum misident.
concolor (Förster, 1841, *Pteromalus*)
psyllaephaga Mani, 1939
siculum Delucchi, 1955

##### Distribution

England

#### Pachyneuron planiscuta

Thomson, 1878

##### Distribution

England, Ireland

#### Pachyneuron vitodurense

Delucchi, 1955

ferrierei Delucchi, 1953

##### Distribution

England

#### Pandelus

Förster, 1856

ZAPACHIA Förster, 1878

#### Pandelus flavipes

(Förster, 1841)

Cleonymus flavipes Förster, 1841
spiloptera (Förster, 1878, *Zapachia*)

##### Distribution

England

##### Notes

Added by Askew (1992c)

#### Panstenon

Walker, 1846

CAUDONIA Walker, 1850

##### Notes

Both species were omitted by Bouček and Graham (1978).

#### Panstenon agylla

(Walker, 1850)

Caudonia agylla Walker, 1850

##### Distribution

Scotland

#### Panstenon oxylus

(Walker, 1839)

Miscogaster oxylus Walker, 1839
assimilis (Nees, 1834, *Pteromalus*)
omissus (Walker, 1841, *Pteromalus*)
pidius Walker, 1850

##### Distribution

England, Wales

#### Pegopus

Förster, 1856

PROSOPON Walker, 1837

#### Pegopus inornatus

(Walker, 1834)

Eutelus inornatus Walker, 1834
sobrius (Walker, 1836, *Pteromalus*)
montanum (Walker, 1837, *Prosopon*)
pyttalus (Walker, 1844, *Pteromalus*)
aollius (Walker, 1845, *Pteromalus*)
rugifrons (Thomson, 1878, *Pteromalus*)

#### Pegopus leptomerus

Graham, 1969

##### Distribution

Scotland

#### Peridesmia

Förster, 1856

#### Peridesmia congrua

(Walker, 1835)

Pteromalus congruus Walker, 1835
Peridesmia congrua ?*lentulus* (Walker, 1839, *Pteromalus*)
lucilla (Walker, 1839, *Pteromalus*)
claripennis (Förster, 1841, *Pteromalus*)
otos (Walker, 1848, *Pteromalus*)
aquisgranensis (Mayr, 1903, *Isocyrtus*)

##### Distribution

Ireland

#### Peridesmia discus

(Walker, 1835)

Pteromalus discus Walker, 1835
subquadratus (Walker, 1836, *Pteromalus*)
phyllus (Walker, 1839, *Pteromalus*)
phytonomi Gahan, 1923

#### Perniphora

Ruschka, 1923

#### Perniphora robusta

Ruschka, 1923

#### Pezilepsis

Delucchi, 1955

#### Pezilepsis dentifera

(Thomson, 1878)

Isocyrtus dentifer Thomson, 1878

##### Distribution

England

##### Notes

BMNH, det. Dale-Skey, added here

#### Phaenocytus

Graham, 1969

#### Phaenocytus glechomae

(Förster, 1841)

Pteromalus glechomae Förster, 1841
heptapotamicus Dzhanokmen, 1990

#### Platneptis

Bouček, 1961

#### Platneptis laeta

(Walker, 1848)

Pteromalus laeta Walker, 1848
maceki Bouček, 1961

#### Platygerrhus

Thomson, 1878

#### Platygerrhus affinis

(Walker, 1836)

Trigonoderus affinis Walker, 1836
amabilis (Walker, 1836, *Trigonoderus*)
gravenhorstii (Ratzeburg, 1852, *Pteromalus*)
gracilis Thomson, 1878

#### Platygerrhus dolosus

(Walker, 1836)

Trigonoderus dolosus Walker, 1836
hirticornis (Walker, 1836, *Trigonoderus*)

##### Distribution

England

#### Platygerrhus ductilis

(Walker, 1836)

Trigonoderus ductilis Walker, 1836
deductor (Walker, 1836, *Trigonoderus*)
figuratus (Walker, 1836, *Trigonoderus*)
linearis (Walker, 1836, *Trigonoderus*)
lappa (Walker, 1848, *Trigonoderus*)

##### Distribution

England

#### Platygerrhus longigena

Graham, 1969

#### Platygerrhus subglaber

Graham, 1969

#### Platygerrhus tarrha

(Walker, 1848)

Trigonoderus tarrha Walker, 1848

#### Platygerrhus unicolor

Graham, 1969

#### Plutothrix

Förster, 1856

ANOGLYPHIS Förster, 1878

#### Plutothrix acuminata

(Thomson, 1878)

Trigonoderus acuminatus Thomson, 1878
cisae Hedqvist, 1966

##### Distribution

England

#### Plutothrix bicolorata

(Spinola, 1808)

Diplolepis bicolorata Spinola, 1808
invenustus (Walker, 1836, *Pteromalus*)
praepileus (Walker, 1836, *Pteromalus*)
scenicus (Walker, 1836, *Pteromalus*)
apicalis (Thomson, 1878, *Trigonoderus*)
vittiger (Thomson, 1878, *Trigonoderus*)

##### Distribution

England, Scotland, Ireland

#### Plutothrix coelius

(Walker, 1839)

Pteromalus coelius Walker, 1839
eleuthera (Walker, 1848, *Pteromalus*)
nubilosa (Förster, 1878, *Anoglyphis*)
britannicus (Morley, 1910, *Pteromalus*)

##### Distribution

England, Ireland

#### Plutothrix nudicoxa

Graham, 1993

##### Notes

Added by Graham (1993)Graham (1993)

#### Plutothrix obtusiclava

Graham, 1993

##### Distribution

England

##### Notes

Added by Graham (1993)

#### Plutothrix trifasciata

(Thomson, 1878)

Trigonoderus trifasciatus Thomson, 1878
foersteri Mayr, 1904

##### Distribution

England

#### Pseudocatolaccus

Masi, 1908

#### Pseudocatolaccus nitescens

(Walker, 1834)

Amblymerus nitescens Walker, 1834
thoracicus (Walker, 1835, *Pteromalus*)
bebryce (Walker, 1839, *Pteromalus*)
elymus (Walker, 1839, *Pteromalus*)
euryops (Förster, 1841, *Pteromalus*)
polyphagus (Förster, 1841, *Pteromalus*)
validus (Förster, 1841, *Pteromalus*)
asphondyliae Masi, 1908
amegallus Dzhanokmen, 1989

##### Distribution

England

#### Psilocera

Walker, 1833

METOPON Walker, 1834
EUPSILOCERA Westwood, 1839
METOPUM Agassiz, 1848
DICHALYSIS Förster, 1856
LOPHOCOMODIA Ashmead, 1888
ACANTHOMETOPON Ashmead, 1904
POLYCYSTOIDES Girault, 1913
PARAPOLYCYSTUS Girault & Dodd, 1915

#### Psilocera crassispina

(Thomson, 1878)

Metopon crassispina Thomson, 1878
curtus (Zetterstedt, 1838, *Pteromalus*) preocc.
curtulus (Dalla Torre, 1898, *Pteromalus*)

#### Psilocera obscura

Walker, 1833

atrum (Walker, 1834, *Metopon*)

##### Distribution

England

#### Psilonotus

Walker, 1834

JANVARTSOVIA Nikol'skaya, 1954

#### Psilonotus achaeus

Walker, 1848

cyamon (Walker, 1848, *Pteromalus*)
viridulus (Thomson, 1878, *Eutelus*)
betulae (Girault, 1917, *Eutelus*)

##### Distribution

England

#### Psilonotus adamas

Walker, 1834

catuli Förster, 1856
aureolus (Thomson, 1878, *Eutelus*)
betulae (Nikol'skaya, 1954, *Janvartsovia*) preocc.

##### Distribution

Wales

#### Psilonotus hortensia

Walker, 1846

alticornis Graham, 1957

#### Psychophagoides

Graham, 1969

#### Psychophagoides crassicornis

Graham, 1969

#### Psychophagus

Mayr, 1904

DIGLOCHIS Thomson, 1878

#### Psychophagus omnivorus

(Walker, 1835)

Pteromalus omnivorus Walker, 1835
processionae (Ratzeburg, 1844, *Pteromalus*)
rotundatus (Ratzeburg, 1844, *Pteromalus*)
antorides (Walker, 1845, *Pteromalus*)
coeruleocephalae (Ratzeburg, 1852, *Pteromalus*)
chrysorrhoeae (Dalla Torre, 1898, *Pteromalus*)

##### Distribution

England

#### Pteromalus

Swederus, 1795

COLAS Curtis, 1827
GNATHO Curtis, 1829
METOPACHIA Westwood, 1839
HABROCYTUS Thomson, 1878
METOPOPACHIA Dalla Torre, 1898
HETEROLACCUS Masi, 1937
GERONTIDELLA Szelényi, 1982

##### Notes

Doubtfully placed species of *Pteromalus*:

[*aeson* Walker, 1848 nom. dub.]

[*mediocris* Walker, 1835 nom. dub.]

[*tiburtus* Walker, 1839 nom. dub.]

#### Pteromalus albipennis

Walker, 1835

coeruleus Dalman, 1820
cingulipes Walker, 1835
plenus Walker, 1835
albipennis Zetterstedt, 1838 preocc.
Pteromalus albipennis ?*hedymeles* Walker, 1839
zelus Walker, 1839
coeno Walker, 1848
diomedon Walker, 1848
Pteromalus albipennis ?*larymna* Walker, 1848
orthagus Walker, 1848
priansos Walker, 1848
Pteromalus albipennis ?*suia* Walker, 1848
beryllinus (Thomson, 1878, *Etroxys*)

##### Distribution

England

#### Pteromalus altus

(Walker, 1834)

Eutelus altus Walker, 1834

#### Pteromalus apum

(Retzius, 1783)

Ichneumon apum Retzius, 1783
venustus Walker, 1835
planiscuta Thomson, 1878

##### Distribution

Scotland

#### Pteromalus aureolus

(Thomson, 1878)

Etroxys aureolus Thomson, 1878
Pteromalus aureolus ?*ortalus* Walker, 1839

#### Pteromalus bedeguaris

(Thomson, 1878)

Etroxys bedeguaris Thomson, 1878

##### Distribution

England

#### Pteromalus berylli

Walker, 1835

ariomedes Walker, 1839

#### Pteromalus bifoveolatus

Förster, 1861

Pteromalus bifoveolatus ?*saturniae* Rudow, 1886
mauritanus (Masi, 1937, *Heterolaccus*)

#### Pteromalus brachygaster

(Graham, 1969)

Habrocytus brachygaster Graham, 1969

##### Distribution

England

#### Pteromalus cardui

(Erdös, 1953)

Cecidostiba cardui Erdös, 1953

#### Pteromalus caudiger

(Graham, 1969)

Habrocytus caudiger Graham, 1969

#### Pteromalus chlorospilus

(Walker, 1834)

Eutelus chlorospilus Walker, 1834
obscuratus Walker, 1836
servulus Walker, 1836

#### Pteromalus chrysos

Walker, 1836

inclusus Walker, 1836
telon Walker, 1839
zipaetes Walker, 1839
eucerus Ratzeburg, 1848
acutigena (Thomson, 1878, *Etroxys*)
poecilopus (Crawford, 1910, *Hypopteromalus*)

##### Distribution

England

#### Pteromalus cioni

(Thomson, 1878)

Etroxys cioni Thomson, 1878

#### Pteromalus conformis

(Graham, 1969)

Habrocytus conformis Graham, 1969

#### Pteromalus crassicornis

Zetterstedt, 1838

##### Distribution

England

##### Notes

BMNH, det. Baur, added here

#### Pteromalus cyniphidis

(Linnaeus, 1758)

Ichneumon cyniphidis Linnaeus, 1758
capreae (Linnaeus, 1761, *Cynips*)

##### Distribution

England

#### Pteromalus decipiens

(Graham, 1969)

Habrocytus decipiens Graham, 1969

##### Distribution

England

#### Pteromalus dispar

(Curtis, 1827)

Colas dispar Curtis, 1827
braconidis (Bouché, 1834, *Diplolepis*)
basalis Walker, 1835
cabarnos Walker, 1839
jaravus Walker, 1846 misspelling
larvarum Nees, 1834
mesochlorus Walker, 1835
saravus Walker, 1845
jouanensis Ratzeburg, 1848
radialis (Thomson, 1878, *Etroxys*)

##### Distribution

England

#### Pteromalus dolichurus

(Thomson, 1878)

Etroxys dolichurus Thomson, 1878
Pteromalus dolichurus ?*albipes* (Zetterstedt, 1838, *Entedon*)
Pteromalus dolichurus ?*excrescentium* Ratzeburg, 1848

#### Pteromalus elevatus

(Walker, 1834)

Eutelus elevatus Walker, 1834
boreus Walker, 1839
ceropasades Walker, 1839
deucetius Walker, 1839
dentifer (Thomson, 1878, *Etroxys*)

##### Distribution

England, Wales

#### Pteromalus fuscipennis

(Walker, 1834)

Eutelus fuscipennis Walker, 1834

##### Distribution

England

#### Pteromalus helenomus

(Graham, 1969)

Habrocytus helenomus Graham, 1969

##### Distribution

England

#### Pteromalus hieracii

(Thomson, 1878)

Etroxys hieracii Thomson, 1878
graciliventris (Szelényi, 1982, *Gerontidella*)

##### Distribution

England

#### Pteromalus intermedius

(Walker, 1834)

Eutelus intermedius Walker, 1834
Pteromalus intermedius ?*impeditus* Walker, 1835
Pteromalus intermedius ?*obscurus* (Thomson, 1878, *Habrocytus*)

##### Distribution

England

#### Pteromalus isarchus

Walker, 1839

##### Distribution

England

#### Pteromalus janssoni

(Graham, 1969)

Habrocytus janssoni Graham, 1969

#### Pteromalus leucanthemi

Janzon, 1980

##### Distribution

England

##### Notes

Added by Jennings (2007a). Polaszek et al. (2004) Polaszek et al. (2004) had recorded *P. leucanthemi* as new to Britain but this was based on a misidentification (H. Baur, pers. comm. to G. Broad).

#### Pteromalus microps

(Graham, 1969)

Habrocytus microps Graham, 1969

##### Distribution

England, Ireland

#### Pteromalus musaeus

Walker, 1844

tarsatus Zetterstedt, 1838
trypetae (Thomson, 1878, *Etroxys*)

#### Pteromalus myopitae

(Graham, 1969)

Habrocytus myopitae Graham, 1969

#### Pteromalus ochrocerus

(Thomson, 1878)

Etroxys ochrocerus Thomson, 1878
ovatus Walker, 1835
Pteromalus ochrocerus ?*bienna* Walker, 1848
ovatulus Dalla Torre, 1898

#### Pteromalus papaveris

Förster, 1841

##### Distribution

England

##### Notes

Added by Jennings (2001). Graham (1969a) had recorded doubtfully identified specimens from Britain.

#### Pteromalus parietinae

(Graham, 1969)

Habrocytus parietinae Graham, 1969

#### Pteromalus patro

Walker, 1848

#### Pteromalus platyphilus

Walker, 1874

pappi (Szelényi, 1982, *Catolaccus*)
amplus (Walker, 1836, *Catolaccus*)

##### Distribution

England

#### Pteromalus procerus

Graham, 1969

#### Pteromalus puparum

(Linnaeus, 1758)

Ichneumon puparum Linnaeus, 1758
antiopae (Scopoli, 1763, *Ichneumon*)
latifrons Walker, 1835
cephalotes Walker, 1836
comes Walker, 1836
ornytus Walker, 1839
brassicae Curtis, 1842
pontiae Curtis, 1842
orinus Walker, 1845
nigricans Walker, 1872
brassicae Packard, 1877 preocc.
pieridis Provancher, 1881
nigritulus Dalla Torre, 1898
australicus Girault & Dodd, 1915

#### Pteromalus puparum

(Newport, 1840)

Eupelmus puparum Newport, 1840

##### Distribution

England

##### Notes

Transferred from *Eupelmus* to *Pteromalus* in Gibson and Fusu (2016), as a junior secondary homonym but not synonym of *P. puparum* (Linnaeus). The only mention of this species is in the original description (Newport 1840); omitted by Bouček and Graham (1978)

#### Pteromalus semotus

(Walker, 1834)

Eutelus semotus Walker, 1834
cupreus Walker, 1835
imbutus Walker, 1835
lugubris Walker, 1835
solutus Walker, 1835
thalassinus Walker, 1836
equestris Walker, 1836
maerens Walker, 1836
Pteromalus semotus ?*mutia* Walker, 1839
pione Walker, 1839
amnisos Walker, 1848
glautias Walker, 1848
parvinucha (Thomson, 1878, *Etroxys*)
maereus Dalla Torre, 1898
variabilis Ratzeburg, 1844
cupreicolor Dalla Torre, 1898
marginicollis (Cameron, 1906, *Etroxys*)
milleri (Delucchi & Verbeke, 1953, *Habrocytus*)

##### Distribution

England

#### Pteromalus sequester

Walker, 1835

infectus Walker, 1835
placidus Walker, 1835
varius Walker, 1835
Pteromalus sequester ?*epimelas* Walker, 1836
simulans Walker, 1836
oroetes Walker, 1839
eulimene Walker, 1848
leguminum Ratzeburg, 1852
insularis Walker, 1872
medicaginis (Gahan, 1914, *Habrocytus*)

##### Distribution

England

#### Pteromalus sonchi

Janzon, 1983

##### Notes

Added by Jennings (2009c)

#### Pteromalus sophax

Walker, 1839

#### Pteromalus squamifer

Thomson, 1878

#### Pteromalus tereus

Walker, 1839

#### Pteromalus tibiellus

Zetterstedt, 1838

#### Pteromalus tripolii

(Graham, 1969)

Habrocytus tripolii Graham, 1969

##### Distribution

England

#### Pteromalus varians

(Spinola, 1808)

Diplolepis varians Spinola, 1808
grandis Walker, 1835
latipennis Walker, 1835
tenuicornis Förster, 1841

##### Distribution

England

#### Pteromalus vibulenus

(Walker, 1839)

Ormocerus vibulenus Walker, 1839
blunckii (Blunck, 1944, *Habrocytus*)

##### Distribution

England

#### Rakosina

Bouček, 1956

BROKKIA Hedqvist, 1977

#### Rakosina deplanata

Bouček, 1956

paradoxa (Hedqvist, 1977, *Brokkia*)

##### Distribution

England

##### Notes

Added by Askew and Shaw (1979a)

#### Rhaphitelus

Walker, 1834

STYLOCERAS Ratzeburg, 1844
RHAPHIDOTELUS Agassiz, 1845
STORTHYGOCERUS Ratzeburg, 1848
EUCERCHYSIUS Brèthes, 1913

#### Rhaphitelus maculatus

Walker, 1834

hecato (Walker, 1839, *Pteromalus*)
subulifer (Förster, 1841, *Pteromalus*)
scolytii (Brèthes, 1913, *Eucerchysius*)

#### Rhopalicus

Förster, 1856

#### Rhopalicus guttatus

(Ratzeburg, 1844)

Ichneumon guttatus Ratzeburg, 1844

#### Rhopalicus quadratus

(Ratzeburg, 1844)

Pteromalus quadratus Ratzeburg, 1844
neostadiensis (Ratzeburg, 1844, *Pteromalus*)
brevicornis Thomson, 1878

##### Distribution

England, Scotland

#### Rhopalicus tutela

(Walker, 1836)

Cheiropachus tutela Walker, 1836
maculifer (Förster, 1841, *Cleonymus*)
immaculatus (Ratzeburg, 1844, *Pteromalus*)
spinolae (Ratzeburg, 1844, *Pteromalus*)
suspensus (Ratzeburg, 1844, *Pteromalus*)
aemulus (Ratzeburg, 1848, *Pteromalus*)
lunulus (Ratzeburg, 1848, *Pteromalus*)
multicolor (Ratzeburg, 1848, *Pteromalus*)
annellus Thomson, 1878

##### Distribution

England, Scotland

#### Rohatina

Bouček, 1954

#### Rohatina denticulata

Graham, 1969

#### Rohatina inermis

Bouček, 1954

#### Roptrocerus

Ratzeburg, 1848

PACHYCERAS Ratzeburg, 1844
ROPTROCEROIDEA Ishii, 1938

#### Roptrocerus brevicornis

Thomson, 1878

##### Distribution

England

##### Notes

Added by Askew (1992c)

#### Roptrocerus mirus

(Walker, 1834)

Amblymerus mirus Walker, 1834
janssoni Hedqvist, 1955

##### Distribution

England, Scotland, Wales

#### Roptrocerus xylophagorum

(Ratzeburg, 1844)

Pachyceras xylophagorum Ratzeburg, 1844
eccoptogastri (Ratzeburg, 1844, *Pachyceras*)
rectus Provancher, 1887
sulcatus Waterston, 1922
ips (Ishii, 1938, *Roptroceroidea*)
karafutoensis (Ishii, 1938, *Roptroceroidea*)

##### Distribution

England, Scotland

#### Sceptrothelys

Graham, 1956

BRIMERIA Hedqvist, 1977
STENETROIDEA Szelényi, 1982

#### Sceptrothelys deione

(Walker, 1839)

Miscogaster deione Walker, 1839
charops (Walker, 1839, *Pteromalus*)
Sceptrothelys deione ?*laricinellae* (Ratzeburg, 1848, *Pteromalus*)
aeacus (Walker, 1848, *Pteromalus*)
aeneiscapus (Thomson, 1878, *Metopon*)
punctatum (Thomson, 1878, *Metopon*)

##### Distribution

England, Wales

#### Sceptrothelys grandiclava

(Walker, 1835)

Pteromalus grandiclava Walker, 1835
claviger (Förster, 1841, *Pteromalus*)
clavata (Hedqvist, 1977, *Brimeria*)

##### Distribution

England, Wales

#### Sceptrothelys intermedia

Graham, 1969

##### Distribution

England

#### Sceptrothelys parviclava

Graham, 1969

#### Schizonotus

Ratzeburg, 1852

#### Schizonotus latus

(Walker, 1835)

Pteromalus latus Walker, 1835
incongruens (Masi, 1907, *Arthrolytus*)
smithii (Gahan, 1913, *Coelopisthia*)

#### Schizonotus sieboldi

(Ratzeburg, 1848)

Pteromalus sieboldi Ratzeburg, 1848

#### Spaniopus

Walker, 1833

#### Spaniopus amoenus

Förster, 1856

##### Distribution

England

##### Notes

BMNH, det. Bouček, added here

#### Spaniopus dissimilis

Walker, 1833

elegans Förster, 1856
modestus (Gahan, 1922, *Polyscelis*)

#### Spaniopus peisonis

(Erdös, 1957)

Gyrinophagus peisonis Erdös, 1957
polyspilus misident.

##### Distribution

England

#### Sphegigaster

Spinola, 1811

TRIGONOGASTRA Ashmead, 1904
PARATRIGONOGASTRA Girault, 1915
BASILEWSKYELLA Risbec, 1957

#### Sphegigaster brevicornis

(Walker, 1833)

Dicyclus brevicornis Walker, 1833

#### Sphegigaster glabrata

Graham, 1969

##### Distribution

England

#### Sphegigaster intersita

Graham, 1969

##### Distribution

England

#### Sphegigaster nigricornis

(Nees, 1834)

Chrysolampus nigricornis Nees, 1834

##### Distribution

England

#### Sphegigaster obliqua

Graham, 1969

##### Distribution

England

#### Sphegigaster pallicornis

(Spinola, 1808)

Diplolepis pallicornis Spinola, 1808
flavicornis (Walker, 1833, *Merismus*)
coronatus (Förster, 1841, *Chrysolampus*)
pallidicornis (Dalla Torre, 1898, *Chrysolampus*)

##### Distribution

England

#### Sphegigaster pedunculiventris

(Spinola, 1808)

Diplolepis pedunculiventris Spinola, 1808
aculeatus (Walker, 1833, *Merismus*)

##### Distribution

England

#### Sphegigaster permagna

Graham, 1984

##### Distribution

England

##### Notes

Added by Graham (1984)

#### Sphegigaster stepicola

Bouček, 1965

melanagromyzae (Mani, 1971, *Acroclisis*)

##### Distribution

England

##### Notes

BMNH, det. Thuroczy, added here

#### Sphegigaster truncata

Thomson, 1878

##### Distribution

England

#### Spilomalus

Graham, 1956

#### Spilomalus quadrinota

(Walker, 1835)

Pteromalus quadrinota Walker, 1835

#### Spintherus

Thomson, 1878

#### Spintherus dubius

(Nees, 1834)

Pteromalus dubius Nees, 1834
nigroaeneus (Walker, 1835, *Pteromalus*)
caligatus (Walker, 1836, *Pteromalus*)
conterminus (Walker, 1836, *Pteromalus*)
orbiculatus (Walker, 1836, *Pteromalus*)
signatus (Walker, 1836, *Pteromalus*)
codrus (Walker, 1839, *Pteromalus*)
flavitarsis (Förster, 1841, *Pteromalus*)
lutescens (Förster, 1841, *Pteromalus*)
triqueter (Förster, 1841, *Pteromalus*)
alimentus (Walker, 1848, *Pteromalus*)
anchinoe (Walker, 1848, *Pteromalus*)
hermachus (Walker, 1848, *Pteromalus*)
opheltes (Walker, 1848, *Pteromalus*)
caligatus (Walker, 1874, *Pteromalus*) preocc.
obscurus (Thomson, 1878)

##### Distribution

England, Wales

#### Staurothyreus

Graham, 1956

#### Staurothyreus cruciger

Graham, 1956

#### Stenomalina

Ghesquière, 1946

STENOMALUS Thomson, 1878 preocc.

#### Stenomalina communis

(Nees, 1834)

Pteromalus communis Nees, 1834
bifrons (Walker, 1836, *Pteromalus*)
continuus (Walker, 1836, *Pteromalus*)
Stenomalina communis ?*dercyllus* (Walker, 1839, *Pteromalus*)
erasippus (Walker, 1839, *Pteromalus*)
mycale (Walker, 1839, *Pteromalus*)
nyctimus (Walker, 1839, *Pteromalus*)
cerycus (Walker, 1848, *Pteromalus*)
rugosus (Thomson, 1878, *Etroxys*)
laetus (Ruschka, 1912, *Stenomalus*)

#### Stenomalina dives

(Walker, 1835)

Pteromalus dives Walker, 1835
mesapos (Walker, 1848, *Pteromalus*)

#### Stenomalina epistena

(Walker, 1835)

Pteromalus epistenus Walker, 1835
linearis (Walker, 1835, *Pteromalus*)
crotus (Walker, 1839, *Pteromalus*)
elyros (Walker, 1848, *Pteromalus*)
themiso (Walker, 1848, *Pteromalus*)
subfumatus (Thomson, 1878, *Etroxys*)

#### Stenomalina favorinus

(Walker, 1839)

Pteromalus favorinus Walker, 1839

#### Stenomalina fervida

Graham, 1965

#### Stenomalina fontanus

(Walker, 1839)

Pteromalus fontanus Walker, 1839
cosingas (Walker, 1839, *Pteromalus*)

#### Stenomalina gracilis

(Walker, 1834)

Eutelus gracilis Walker, 1834
aurifer (Walker, 1835, *Pteromalus*)
thessalus (Walker, 1839, *Pteromalus*)
psittacinus (Förster, 1841, *Pteromalus*)
seladonius (Förster, 1841, *Pteromalus*)

##### Distribution

England, Wales

#### Stenomalina illudens

(Walker, 1836)

Pteromalus illudens Walker, 1836
gaudens (Walker, 1836, *Pteromalus*)
hyloe (Walker, 1848, *Pteromalus*)
crassicornis (Thomson, 1878, *Etroxys*)

#### Stenomalina laticeps

(Walker, 1850)

Pteromalus laticeps Walker, 1850

##### Distribution

England

#### Stenomalina liparae

(Giraud, 1863)

Pteromalus liparae Giraud, 1863

##### Distribution

England

#### Stenomalina micans

(Olivier, 1813)

Pteromalus micans Olivier, 1813
bellus (Walker, 1836, *Pteromalus*)
Stenomalina micans ?*chloris* (Walker, 1836, *Pteromalus*)

##### Distribution

England

#### Stenomalina oxygyne

(Walker, 1835)

Pteromalus oxygyne Walker, 1835
dorsalis (Walker, 1836, *Pteromalus*)

#### Stinoplus

Thomson, 1878

##### Notes

Some distribution data from Askew (2011)

#### Stinoplus etearchus

(Walker, 1848)

Pteromalus etearchus Walker, 1848
aureolus (Thomson, 1878, *Etroxys*)

##### Distribution

England, Scotland, Wales, Isle of Man

#### Stinoplus jenningsi

Askew, 2011

##### Distribution

England

##### Notes

Added by Askew (2011)

#### Stinoplus pervasus

(Walker, 1836)

Pteromalus pervasus Walker, 1836
tedanius (Walker, 1845, *Pteromalus*)

##### Distribution

England

#### Synedrus

Graham, 1956

#### Synedrus transiens

(Walker, 1835)

Pteromalus transiens Walker, 1835
cavigena Graham, 1956

##### Distribution

England

#### Syntomopus

Walker, 1833

MERISMORELLA Girault, 1926

#### Syntomopus agromyzae

Hedqvist, 1973

#### Syntomopus incisus

Thomson, 1878

##### Distribution

England

#### Syntomopus incurvus

Walker, 1833

dirce (Walker, 1839, *Miscogaster*)
phylander (Walker, 1848, *Lamprotatus*)
madizae (Rondani, 1877, *Chrysolampus*)

##### Distribution

England, Scotland

#### Syntomopus oviceps

Thomson, 1878

##### Distribution

England, Scotland

#### Syntomopus thoracicus

Walker, 1833

##### Distribution

England

#### Tomicobia

Ashmead, 1899

IPOCOELIUS Ruschka, 1924
KARPINSKIELLA Bouček, 1955

#### Tomicobia pityophthori

(Bouček, 1955)

Karpinskiella pityophthori Bouček, 1955

##### Distribution

England

##### Notes

Added by Askew (1992c)

#### Tomicobia promulus

(Walker, 1840)

Pteromalus promulus Walker, 1840
acrotatus (Walker, 1845, *Pteromalus*)
sublaevis (Thomson, 1878, *Metopon*)
sublevis (Dalla Torre, 1898, *Dirhicnus*)

#### Toxeuma

Walker, 1833

CIRDANIA Hedqvist, 1974

#### Toxeuma acilius

(Walker, 1848)

Lamprotatus acilius Walker, 1848

##### Distribution

England, Scotland

#### Toxeuma discretum

Graham, 1984

##### Distribution

England

##### Notes

Added by Graham (1984)

#### Toxeuma fuscicorne

Walker, 1833

lugubris (Walker, 1833, *Miscogaster*)
ericae Walker, 1833
accia (Walker, 1848, *Gastrancistrus*)

##### Distribution

England, Scotland, Wales

#### Toxeuma paludum

Graham, 1959

##### Distribution

England, Wales

#### Toxeuma styliclava

(Hedqvist, 1974)

Cirdania styliclava Hedqvist, 1974
mucronatum (Graham, 1984)

##### Distribution

England

##### Notes

BMNH, det. Thuroczy, added here

#### Toxeuma subtruncatum

Graham, 1959

##### Distribution

England

#### Trichomalopsis

Crawford, 1913

EUPTEROMALUS Kurdjumov, 1913
NEMICROMELUS Girault, 1917
METADICYLUS Girault, 1926

#### Trichomalopsis acuminata

(Graham, 1969)

Eupteromalus acuminatus Graham, 1969

#### Trichomalopsis albopilosus

(Graham, 1969)

Eupteromalus albopilosus Graham, 1969

##### Distribution

England

##### Notes

Added by Askew (1992c)

#### Trichomalopsis caricicola

(Graham, 1969)

Eupteromalus caricicola Graham, 1969

#### Trichomalopsis exigua

(Walker, 1834)

Meraporus exiguus Walker, 1834

##### Distribution

England

#### Trichomalopsis fucicola

(Walker, 1835)

Pteromalus fucicola Walker, 1835

##### Distribution

England

#### Trichomalopsis hemiptera

(Walker, 1835)

Pteromalus hemipterus Walker, 1835
apicalis (Walker, 1835, *Pteromalus*)
pedestris (Förster, 1861, *Pteromalus*)
nidulans (Thomson, 1878, *Pteromalus*)

##### Distribution

England

#### Trichomalopsis lasiocampae

(Graham, 1969)

Eupteromalus lasiocampae Graham, 1969

##### Distribution

England

#### Trichomalopsis laticeps

(Graham, 1969)

Eupteromalus laticeps Graham, 1969

#### Trichomalopsis littoralis

(Graham, 1969)

Eupteromalus littoralis Graham, 1969

#### Trichomalopsis maura

(Graham, 1969)

Eupteromalus maurus Graham, 1969

#### Trichomalopsis microptera

(Lindeman, 1887)

Merisus microptera Lindeman, 1887
coxalis (Ashmead, 1897, *Baeotomus*)
arvensis (Kurdjumov, 1914, *Eupteromalus*)

##### Distribution

Scotland

##### Notes

Graham (1969a) was unable to check the identity of the British specimens.

#### Trichomalopsis peregrina

(Graham, 1969)

Eupteromalus peregrinus Graham, 1969

##### Distribution

England

#### Trichomalopsis pompilicola

(Graham, 1969)

Eupteromalus pompilicola Graham, 1969

#### Trichomalopsis potatoriae

(Graham, 1969)

Eupteromalus potatoriae Graham, 1969

#### Trichomalopsis scaposa

(Graham, 1969)

Eupteromalus scaposa Graham, 1969

#### Trichomalopsis subapterus

(Riley, 1885)

Merisus subapterus Riley, 1885
fulvipes (Forbes, 1885, *Pteromalus*)

#### Trichomalopsis tenuicornis

Graham, 1996

##### Distribution

England

##### Notes

Added by Graham (1996)

#### Trichomalopsis tigasis

(Walker, 1839)

Pteromalus tigasis Walker, 1839

#### Trichomalus

Thomson, 1878

#### Trichomalus apertus

(Walker, 1835)

Pteromalus apertus Walker, 1835
Trichomalus apertus ?*alopius* (Walker, 1848, *Pteromalus*)

##### Distribution

England, Wales

#### Trichomalus bracteatus

(Walker, 1835)

Pteromalus bracteatus Walker, 1835
flammiger (Walker, 1835, *Pteromalus*)
herbidus (Walker, 1835, *Pteromalus*)
attenuatus (Walker, 1836, *Pteromalus*)
balux (Walker, 1836, *Pteromalus*)
longulus (Walker, 1836, *Pteromalus*)
Trichomalus bracteatus ?*acraea* (Walker, 1839, *Pteromalus*)
Trichomalus bracteatus ?*automedon* (Walker, 1839, *Pteromalus*)
Trichomalus bracteatus ?*daimenes* (Walker, 1839, *Pteromalus*)
chalcolampus (Förster, 1841, *Pteromalus*)
fasciatus (Förster, 1841, *Pteromalus*)

##### Distribution

England, Scotland, Wales, Ireland

#### Trichomalus campestris

(Walker, 1834)

Amblymerus campestris Walker, 1834
tenuicornis (Walker, 1834, *Amblymerus*)
cyniphis (Nees, 1834, *Pteromalus*)
rufipes (Nees, 1834, *Pteromalus*)
fumipennis (Walker, 1835, *Pteromalus*)
tenuis (Walker, 1835, *Pteromalus*)
redactus (Walker, 1835, *Pteromalus*)
concisus (Walker, 1836, *Pteromalus*)
nubeculosus (Förster, 1841, *Pteromalus*)
coxalis (Thomson, 1878, *Isocyrtus*)

##### Distribution

England, Scotland

#### Trichomalus conifer

(Walker, 1836)

Pteromalus conifer Walker, 1836
laticornis (Walker, 1836, *Pteromalus*)

##### Distribution

England

#### Trichomalus coryphe

(Walker, 1839)

Pteromalus coryphe Walker, 1839

#### Trichomalus curtus

(Walker, 1835)

Pteromalus curtus Walker, 1835

#### Trichomalus elongatus

Delucchi & Graham, 1956

##### Distribution

England

#### Trichomalus flagellaris

Graham, 1969

#### Trichomalus fulvipes

(Walker, 1836)

Pteromalus fulvipes Walker, 1836
Trichomalus fulvipes ?*amphimedon* (Walker, 1839, *Pteromalus*)
operosus (Förster, 1841, *Pteromalus*)

##### Distribution

England

#### Trichomalus germanus

(Dalla Torre, 1898)

Pteromalus germanus Dalla Torre, 1898
exilis (Förster, 1841, *Pteromalus*) preocc.

##### Distribution

England, Wales

##### Notes

BMNH, det. Thuroczy, added here, and removed from possible synonymy under *T. conifer*.

#### Trichomalus gracilicornis

(Zetterstedt, 1838)

Pteromalus gracilicornis Zetterstedt, 1838
punctiger (Thomson, 1878, *Isocyrtus*)

##### Distribution

England, Wales

#### Trichomalus gynetelus

(Walker, 1835)

Pteromalus gynetelus Walker, 1835
stigmatizans (Walker, 1872, *Pteromalus*)

##### Distribution

England

#### Trichomalus helvipes

(Walker, 1834)

Eutelus helvipes Walker, 1834
cuprinus (Walker, 1835, *Pteromalus*)
detritus (Walker, 1835, *Pteromalus*)
famulus (Walker, 1835, *Pteromalus*)
obtusus (Walker, 1835, *Pteromalus*)
perpetuus (Walker, 1835, *Pteromalus*)
futilis (Walker, 1835, *Pteromalus*)
chrysammos (Walker, 1836, *Pteromalus*)
crocale (Walker, 1839, *Pteromalus*)
janira (Walker, 1839, *Pteromalus*)
Trichomalus helvipes ?*saptine* (Walker, 1839, *Pteromalus*)
delectus (Förster, 1841, *Pteromalus*)
lethargicus (Förster, 1841, *Pteromalus*)
peregrinus (Förster, 1841, *Pteromalus*)
quaesitus (Förster, 1841, *Pteromalus*)
carma (Walker, 1848, *Pteromalus*)
hyrtacina (Walker, 1848, *Pteromalus*)
lebadeia (Walker, 1848, *Pteromalus*)
mese (Walker, 1848, *Pteromalus*)
laticeps (Thomson, 1878, *Isocyrtus*)

##### Distribution

England

#### Trichomalus inscitus

(Walker, 1835)

Pteromalus inscitus Walker, 1835
microcerus (Walker, 1835, *Pteromalus*)
tristis (Walker, 1835, *Pteromalus*)
affinis (Walker, 1835, *Pteromalus*)
deiochus (Walker, 1839, *Pteromalus*)
reconditus (Förster, 1841, *Pteromalus*)
diachymatis (Ratzeburg, 1844, *Pteromalus*)
orchestis (Ratzeburg, 1844, *Pteromalus*)
lampe (Walker, 1848, *Pteromalus*)
subnudus (Thomson, 1878, *Isocyrtus*)

##### Distribution

England

#### Trichomalus lepidus

(Förster, 1841)

Pteromalus lepidus Förster, 1841
aeneicoxa (Thomson, 1878, *Isocyrtus*)

##### Distribution

England

#### Trichomalus lonchaeae

Bouček, 1959

#### Trichomalus lucidus

(Walker, 1835)

Pteromalus lucidus Walker, 1835
brevicornis (Walker, 1835, *Pteromalus*)
chalceus (Walker, 1835, *Pteromalus*)
despectus (Walker, 1835, *Pteromalus*)
rusticus (Walker, 1836, *Pteromalus*)
mundus (Förster, 1841, *Pteromalus*)
lyttus (Walker, 1848, *Pteromalus*)
fasciatus (Thomson, 1878, *Isocyrtus*)
purus (Dalla Torre, 1898, *Pteromalus*)

##### Distribution

England

#### Trichomalus nanus

(Walker, 1836)

Pteromalus nanus Walker, 1836
Trichomalus nanus ?*cerpheres* (Walker, 1839, *Pteromalus*)
lucidus (Förster, 1841, *Pteromalus*)
Trichomalus nanus ?*aglaus* (Walker, 1845, *Pteromalus*)
dipoenos (Walker, 1848, *Pteromalus*)
versutus (Förster, 1861, *Pteromalus*)
speciosus (Dalla Torre, 1898, *Pteromalus*)

#### Trichomalus oxygyne

Graham, 1969

#### Trichomalus perfectus

(Walker, 1835)

Pteromalus perfectus Walker, 1835
decisus (Walker, 1835, *Pteromalus*)
decorus (Walker, 1835, *Pteromalus*)
Trichomalus perfectus ?*hippo* (Walker, 1839, *Pteromalus*)
opulentus (Förster, 1841, *Pteromalus*)
laevinucha (Thomson, 1878, *Isocyrtus*)

##### Distribution

England

#### Trichomalus pexatus

(Walker, 1835)

Pteromalus pexatus Walker, 1835
intermedius (Förster, 1841, *Pteromalus*)

##### Distribution

England

#### Trichomalus placidus

(Walker, 1834)

Eutelus placidus Walker, 1834
learchus (Walker, 1845, *Pteromalus*)

##### Distribution

England

#### Trichomalus posticus

(Walker, 1834)

Eutelus posticus Walker, 1834
Trichomalus posticus ?*deudorix* (Walker, 1839, *Pteromalus*)
sunides (Walker, 1845, *Pteromalus*)
Trichomalus posticus ?*xanthe* (Walker, 1845, *Pteromalus*)
punctinucha (Thomson, 1878, *Isocyrtus*)

##### Distribution

England, Scotland

#### Trichomalus repandus

(Walker, 1835)

Pteromalus repandus Walker, 1835
pallicornis (Thomson, 1878, *Isocyrtus*)
cryptophagus (Förster, 1841, *Pteromalus*)
praetermissus (Förster, 1841, *Pteromalus*)
samus (Walker, 1839, *Pteromalus*)
stenotelus (Walker, 1836, *Pteromalus*)
pallidicornis Dalla Torre, 1898

##### Distribution

England

#### Trichomalus robustus

(Walker, 1835)

Pteromalus robustus Walker, 1835
nubilus (Walker, 1835, *Pteromalus*)
vibullius (Walker, 1839, *Pteromalus*)
xanthopterus (Ratzeburg, 1844, *Pteromalus*)
inatos (Walker, 1848, *Pteromalus*)
spiracularis (Thomson, 1878, *Isocyrtus*)

##### Distribution

England, Wales

#### Trichomalus rufinus

(Walker, 1835)

Pteromalus rufinus Walker, 1835
inops (Walker, 1835, *Pteromalus*)
confinis (Walker, 1836, *Pteromalus*)
irus (Walker, 1839, *Pteromalus*)
nitefactus (Förster, 1841, *Pteromalus*)
vagans (Förster, 1841, *Pteromalus*)
pedicellaris (Thomson, 1878, *Isocyrtus*)
rufimanus (Thomson, 1878, *Isocyrtus*)

##### Distribution

England

#### Trichomalus rugosus

Delucchi & Graham, 1956

##### Distribution

England

#### Trichomalus statutus

(Förster, 1841)

Pteromalus statutus Förster, 1841
fertilis (Förster, 1841, *Pteromalus*)

##### Notes

Listed by Dzhanokmen (1978) as occurring in Britain but it is not clear on what basis.

#### Trichomalus tenellus

(Walker, 1834)

Amblymerus tenellus Walker, 1834
saturatus (Walker, 1835, *Pteromalus*)
viridulus (Walker, 1835, *Pteromalus*)
gentilis (Walker, 1836, *Pteromalus*)
axos (Walker, 1848, *Pteromalus*)

##### Distribution

England

#### Trigonoderus

Westwood, 1832

#### Trigonoderus cyanescens

(Förster, 1841)

Cleonymus cyanescens Förster, 1841
quadrum (Nees, 1834, *Cleonymus*)
gribodii (Vollenhoven, 1878, *Hetroxys*)
pedicellaris Thomson, 1878

##### Distribution

England

#### Trigonoderus filatus

Walker, 1836

signatus (Förster, 1841, *Cleonymus*)
brandtii (Ratzeburg, 1844, *Pteromalus*)

##### Distribution

England

#### Trigonoderus princeps

Westwood, 1832

atrovirens Walker, 1836
obscurus Walker, 1836
hirtipes (Zetterstedt, 1838, *Pteromalus*)
lichtensteinii (Ratzeburg, 1844, *Pteromalus*)

##### Distribution

England, Scotland

#### Trigonoderus pulcher

Walker, 1836

contemptus Walker, 1836
tristis Walker, 1836

#### Tritneptis

Girault, 1908

KVASERIA Hedqvist, 1978

#### Tritneptis klugii

(Ratzeburg, 1844)

Pteromalus klugii Ratzeburg, 1844
nematicida (Packard, 1883, *Pteromalus*)

#### Trychnosoma

Graham, 1957

#### Trychnosoma punctipleura

(Thomson, 1878)

Etroxys punctipleura Thomson, 1878

#### Urolepis

Walker, 1846

#### Urolepis maritima

(Walker, 1834)

Hormocerus maritimus Walker, 1834
maritimus (Walker, 1834, *Ormocerus*)
stygne (Walker, 1839, *Miscogaster*)
salinus (Heydon, 1844, *Pteromalus*)
alope (Walker, 1848, *Pteromalus*)

#### Veltrusia

Bouček, 1972

#### Veltrusia rara

Bouček, 1972

##### Distribution

England

##### Notes

Added by Askew (in prep.)

#### Vrestovia

Bouček, 1961

#### Vrestovia fidenas

(Walker, 1848)

Gastrancistrus fidenas Walker, 1848
clypealis Bouček, 1961

#### Xiphydriophagus

Ferrière, 1952

#### Xiphydriophagus meyerinckii

(Ratzeburg, 1848)

Pteromalus meyerinckii Ratzeburg, 1848
xiphydriae (Fahringer, 1935, *Pteromalus*)

##### Distribution

England

### Family Pteromalidae Dalman, 1820: other subfamilies

#### Asaphinae

Ashmead, 1904

#### Asaphes

Walker, 1834

ISOCRATUS Förster, 1856
PARECTROMA Brèthes, 1913

#### Asaphes suspensus

(Nees, 1834)

Chrysolampus suspensus Nees, 1834
altiventris (Nees, 1834, *Chrysolampus*)
petioliventris (Zetterstedt, 1838, *Pteromalus*)
Asaphes suspensus ?*aphidii* (Curtis, 1842, *Colax*)
aphidiphagus (Ratzeburg, 1844, *Chrysolampus*)
aphidicola (Rondani, 1848, *Chrysolampus*)
lucens (Provancher, 1887, *Euplectrus*)
fletcheri (Crawford, 1909, *Megorismus*)
rufipes Brues, 1909
huebrichi (Brèthes, 1913, *Parectroma*)
americana Girault, 1914
bonariensis (Brèthes, 1916, *Pachycrepoideus*)
indicus (Bhatnagar, 1952, *Pachycrepoideus*)
sawraji Sharma & Subba Rao, 1959
uniarticulata (Mani & Saraswat, 1974, *Pachyneuron*)

#### Asaphes vulgaris

Walker, 1834

aeneus (Ratzeburg, 1848, *Chrysolampus*)
aphidophila (Rondani, 1848, *Chrysolampus*)
aenea (Nees, 1834, *Eurytoma*)

##### Distribution

England

##### Notes

**Figure 14.**
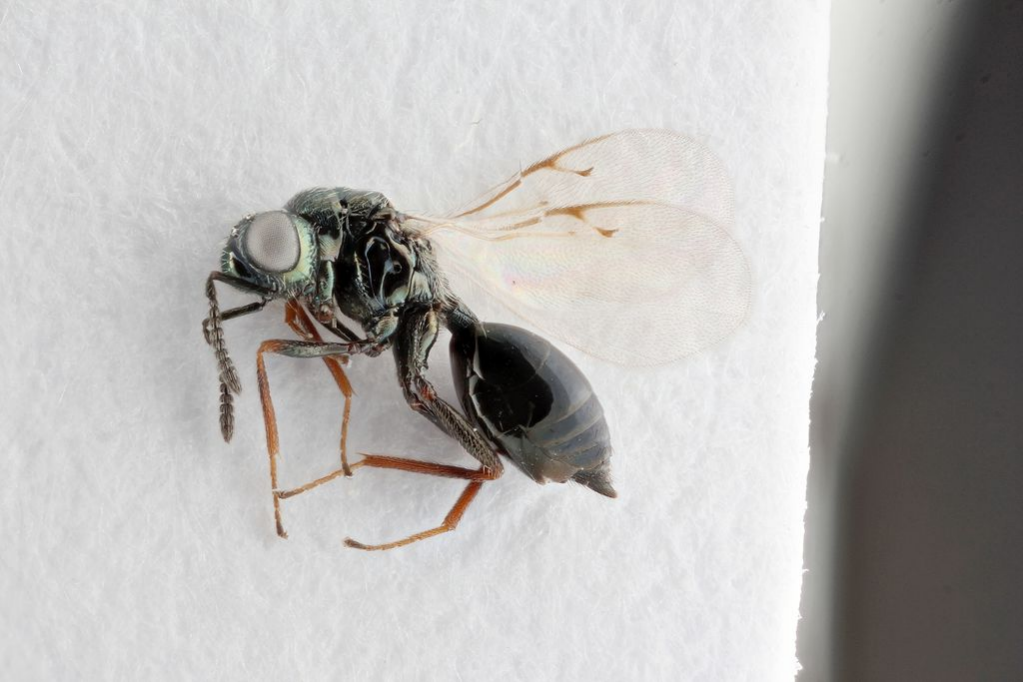
Pteromalidae, Asaphinae: *Asaphes vulgaris* Walker female, BMNH specimen BMNH(E)1414557 [/08/2013, Scilly Isles, Cornwall, England, coll. Dale-Skey] (photo K. McCormack)

See Fig. 14 for habitus

#### Bairamlia

Waterston, 1929

#### Bairamlia fuscipes

Waterston, 1929

nidícola Ferrière, 1934
atrovirens (Bouček, 1955, *Parasaphodes*)

#### Hyperimerus

Girault, 1917

MESPILON Graham, 1957

#### Hyperimerus pusillus

(Walker, 1833)

Cyrtogaster pusilla Walker, 1833
exiguum (Graham, 1957, *Mespilon*)

##### Distribution

England

#### Ceinae

Ashmead, 1904

#### Cea

Walker, 1837

#### Cea pulicaris

Walker, 1837

irene Walker, 1837

##### Distribution

England, Ireland

#### Spalangiopelta

Masi, 1922

#### Spalangiopelta alata

Bouček, 1953

#### Spalangiopelta alboaculeata

Darling, 1995

#### Spalangiopelta procera

Graham, 1966

#### Cerocephalinae

Bouček, 1961

#### Cerocephala

Westwood, 1832

EPIMACRUS Walker, 1833
SCIATHERAS Ratzeburg, 1848
PARASCIATHERAS Masi, 1917
SCIATHERODES Masi, 1917
PROAMOTURA Girault, 1920

#### Cerocephala cornigera

Westwood, 1832

trichotus (Ratzeburg, 1848, *Sciatheras*)

##### Distribution

England

##### Notes

**Figure 15.**
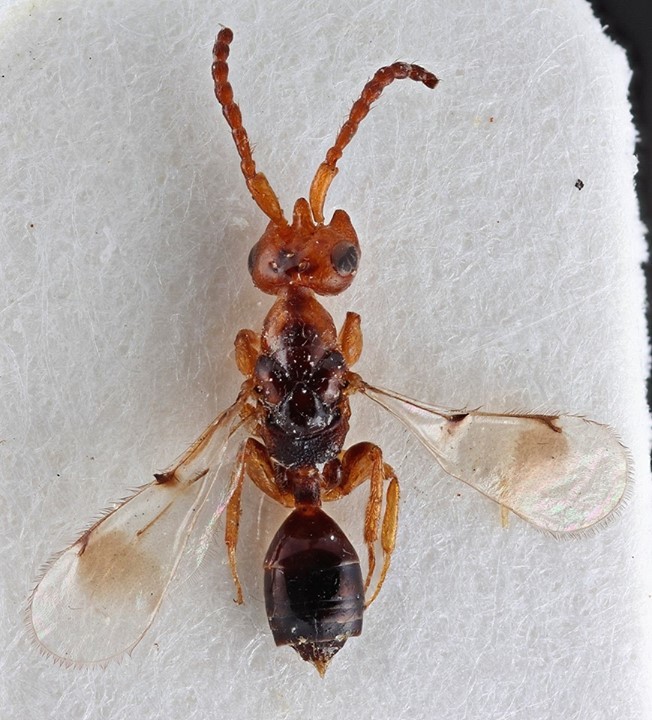
Pteromalidae, Cerocephalinae: *Cerocephala cornigera* Westwood male, BMNH specimen BMNH(E)953670 [1935 to 1936, Ranmore Common, Surrey, England, coll. Richards] (photo K. McCormack)

See Fig. 15 for habitus

#### Cerocephala rufa

(Walker, 1833)

Epimacrus rufus Walker, 1833
dubarae Wallace, 1959

#### Theocolax

Westwood, 1832

LAESTHIA Haliday, 1833
CHOETOSPILA Westwood, 1874
SPALANGIOMORPHA Girault, 1913

#### Theocolax elegans

(Westwood, 1874)

Choetospila elegans Westwood, 1874
metallica (Fullaway, 1913, *Spalangia*)
fasciatipennis (Girault, 1913, *Spalangiomorpha*)
oryzae (Risbec, 1951, *Cerocephala*)
rhizoperthae (Risbec, 1951, *Spalangia*)

##### Distribution

England

#### Theocolax formiciformis

Westwood, 1832

vespertina (Haliday, 1833, *Laesthia*)

##### Distribution

England, Scotland, Wales

#### Cleonyminae

Walker, 1837

#### Cleonymus

Latreille, 1809

PTINOBIUS Ashmead, 1896
APLATYGERRHUS Girault, 1913
SYSTOLOMORPHELLA Girault, 1915
MEGORMYRUS Cockerell, 1926
PARACLEONYMUS Masi, 1927
BEHARELLA Risbec, 1952

#### Cleonymus laticornis

Walker, 1837

thomsoni Erdös, 1957
depressus (Fabricius, 1798, *Ichneumon*) preocc.

##### Distribution

England

##### Notes

**Figure 16.**
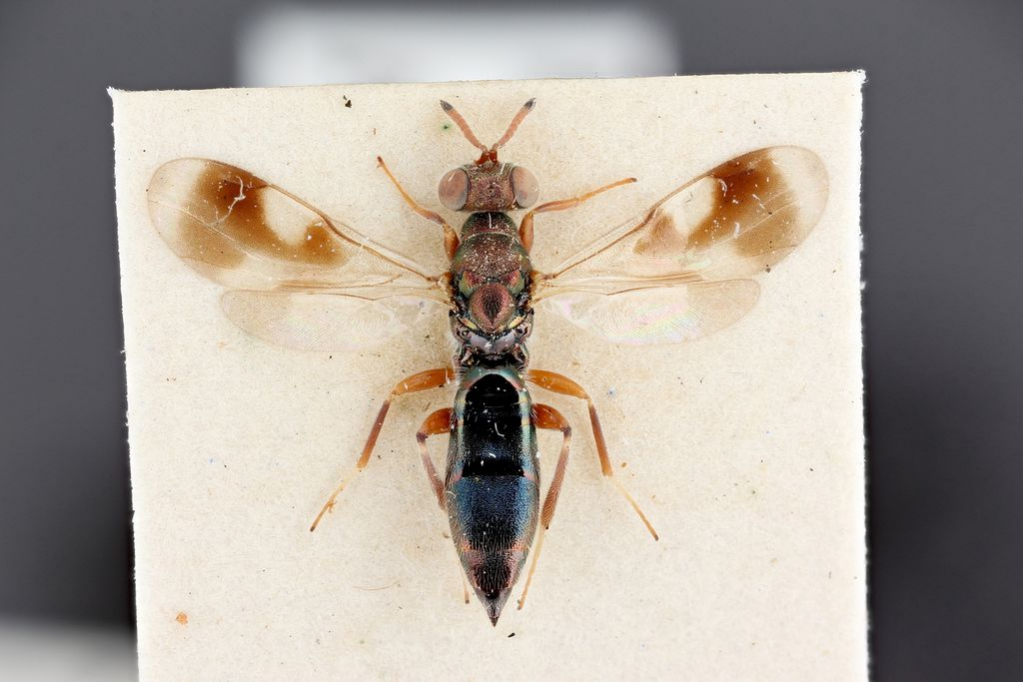
Pteromalidae, Cleonyminae: *Cleonymus laticornis* Walker female, BMNH specimen BMNH(E)953661 [/06/1962, Oxford, Oxfordshire, England, coll. Graham] (photo K. McCormack)

See Fig. 16 for habitus

#### Cleonymus obscurus

Walker, 1837

##### Distribution

England

#### Heydenia

Förster, 1856

#### Heydenia pretiosa

Förster, 1856

excellens Wachtl, 1889
silvestrii (Russo, 1938, *Lycisca*)

##### Notes

Added by Askew (in prep.)

#### Colotrechninae

Thomson, 1876

#### Colotrechnus

Thomson, 1878

ZANONIA Masi, 1921

#### Colotrechnus subcoeruleus

Thomson, 1878

#### Diparinae

Thomson, 1876

#### Dipara

Walker, 1833

TRICORYPHUS Förster, 1856
APTEROLELAPS Ashmead, 1901
EPILELAPS Girault, 1915
APTEROLAELAPS Girault, 1916
HISPANOLELAPS Mercet, 1927
PSEUDIPARELLA Girault, 1927

#### Dipara petiolata

Walker, 1833

cinetoides Walker, 1834
fasciatus (Thomson, 1876, *Tricoryphus*)
coxalis (Mercet, 1927, *Hispanolelaps*)

##### Distribution

England

##### Notes

See Fig. 17 for habitus

#### Eunotinae

Ashmead, 1904

#### Epicopterus

Westwood, 1833

SIMOPTERUS Förster, 1851

#### Epicopterus choreiformis

Westwood, 1833

borges (Walker, 1839, *Ormocerus*)
venustus (Förster, 1851, *Simopterus*)

#### Eunotus

Walker, 1834

MEGAPELTE Förster, 1856

##### Notes

Species of *Eunotus* removed from the British and Irish list:

[?*acutus* Kurdjumov, 1912]

Listed as occurring in Britain by Andriescu and Mitroiu (2003), but presumably in error.

#### Pteromalidae

#### Eunotus cretaceus

Walker, 1834

festucae Masi, 1928

##### Distribution

England

#### Eunotus nigriclavis

(Förster, 1856)

Megapelte nigriclavis Förster, 1856

##### Distribution

England

##### Notes

Identification uncertain (Bouček 1965).

#### Eunotus parvulus

Masi, 1931

aquisgranensis Masi, 1931

#### Macromesinae

Graham, 1959

#### Macromesus

Walker, 1848

METASYSTASIS Girault, 1925
WESENBERGIA Kryger, 1943
CROSSOTOMORIA Delucchi, 1956

#### Macromesus amphiretus

Walker, 1848

occulta (Kryger, 1943, *Wesenbergia*)

#### Miscogastrinae

Walker, 1833

##### Notes

Bouček (1988) restricted the Miscogastrinae to Graham’s (1969) tribe Miscogasterini, with other genera being transferred to the Pireninae and Pteromalinae. Burks (2012) established the correct ending, ‘…trinae’ rather than the frequently used ‘…terini’.

#### Ardilea

Graham, 1959

#### Ardilea convexa

(Walker, 1833)

Miscogaster convexa Walker, 1833
pubicornis (Zetterstedt, 1838, *Pteromalus*)

#### Callimerismus

Graham, 1956

#### Callimerismus fronto

(Walker, 1833)

Merismus fronto Walker, 1833
breviventris (Walker, 1833, *Miscogaster*)

##### Distribution

England, Scotland, Wales

#### Collentis

Heydon, 1992

#### Collentis suecicus

(Graham, 1969)

Callimerismus suecicus Graham, 1969

#### Glyphognathus

Graham, 1956

XESTOGNATHUS Kamijo, 1960

#### Glyphognathus convexus

(Delucchi, 1953)

Stictomischus convexus Delucchi, 1953
umbelliferae Graham, 1956

##### Distribution

England, Scotland

#### Glyphognathus flammeus

(Delucchi, 1953)

Stictomischus flammeus Delucchi, 1953

##### Distribution

England

#### Glyphognathus laevigatus

(Delucchi, 1953)

Stictomischus laevigatus Delucchi, 1953

##### Distribution

England

##### Notes

Although Graham (1969a) had seen only the Hungarian holotype, Bouček and Graham (1978) listed this as a British species; Baur (2005) subsequently listed English material.

#### Glyphognathus laevis

(Delucchi, 1953)

Stictomischus laevis Delucchi, 1953

##### Distribution

England

#### Halticoptera

Spinola, 1811

PACHYLARTHRUS Westwood, 1832
PHAGONIA Curtis, 1832
DICYCLUS Walker, 1833
PHACOSTOMUS Nees, 1834
MEGORISMUS Walker, 1846
TITYROS Walker, 1848
MEGALORISMUS Schulz, 1906
HALTICOPTERINA Erdös, 1946
ABYRSOMELE Dzhanokmen, 1975

##### Notes

Some distribution data from Askew (1972)

#### Halticoptera aenea

(Walker, 1833)

Dicyclus aeneus Walker, 1833
cinctipes (Walker, 1833, *Miscogaster*)
nigroaenea (Walker, 1833, *Miscogaster*)
tristis (Nees, 1834, *Chrysolampus*)
sophron (Walker, 1839, *Pteromalus*)
petiolata Thomson, 1876
liqueatus (Ashmead, 1894, *Cyrtogaster*)
citripes (Ashmead, 1896, *Cyrtogaster*)
occidentalis (Ashmead, 1896, *Cyrtogaster*)
floridanus (Ashmead, 1896, *Polycyrtus*)
foersteri (Crawford, 1913, *Polycystus*)

##### Distribution

England, Scotland, Wales, Ireland

#### Halticoptera aureola

Graham, 1972

#### Halticoptera circulus

(Walker, 1833)

Dicyclus circulus Walker, 1833
fuscicornis (Walker, 1833, *Dicyclus*)
tristis (Walker, 1833, *Dicyclus*)
brevicornis (Zetterstedt, 1838, *Pteromalus*)
palpigerus (Zetterstedt, 1838, *Pteromalus*)
daiphron (Walker, 1839, *Miscogaster*)
suilius (Walker, 1839, *Miscogaster*)
lapponicus (Dalla Torre, 1898, *Pteromalus*)

##### Distribution

England, Scotland, Wales, Ireland

#### Halticoptera collaris

(Walker, 1836)

Pteromalus collaris Walker, 1836
planiscuta Thomson, 1876

##### Distribution

England

#### Halticoptera crius

(Walker, 1839)

Miscogaster crius Walker, 1839
citritibius (Rondani, 1877, *Chrysolampus*)

##### Distribution

England, Wales

#### Halticoptera dimidiata

(Förster, 1841)

Phacostomus dimidiata Förster, 1841
brevicornis Thomson, 1876

##### Distribution

England, Wales

#### Halticoptera elongatula

Graham, 1972

##### Distribution

England

#### Halticoptera flavicornis

(Spinola, 1808)

Diplolepis flavicornis Spinola, 1808
smaragdina (Curtis, 1832, *Phagonia*)
insignis (Westwood, 1832, *Pachylarthrus*)

##### Distribution

England

#### Halticoptera hippeus

(Walker, 1839)

Miscogaster hippeus Walker, 1839
crassipes Thomson, 1876
eurybia (Walker, 1839, *Miscogaster*)
tyrrhaeus (Walker, 1839, *Miscogaster*)

##### Distribution

England, Wales

#### Halticoptera laevigata

Thomson, 1876

##### Distribution

England

#### Halticoptera letitiae

Askew, 1972

##### Distribution

England

#### Halticoptera patellana

(Dalman, 1818)

Diplolepis patellana Dalman, 1818
flavicornis (Curtis, 1832, *Phagonia*)
patellana (Curtis, 1832, *Phagonia*) preocc.
aeratus (Walker, 1839, *Miscogaster*)
pisuthrus (Walker, 1839, *Ormocerus*)
similis (Förster, 1841, *Phacostomus*)

##### Distribution

England, Scotland, Wales

#### Halticoptera polita

(Walker, 1834)

Eutelus politus Walker, 1834
mandrocles (Walker, 1839, *Ormocerus*)
festiva Thomson, 1876

##### Distribution

England, Scotland, Wales, Ireland

#### Halticoptera poreia

(Walker, 1848)

Tityros poreia Walker, 1848
Halticoptera poreia ?*aletes* (Walker, 1848, *Ormocerus*)
cercaphrus (Walker, 1848, *Pteromalus*)

##### Distribution

England, Wales

#### Halticoptera triannulata

(Erdös, 1946)

##### Distribution

England

#### Halticoptera violacea

Askew, 1972

##### Distribution

England

#### Lamprotatus

Westwood 1833

SKELOCERAS Delucchi, 1953
OCTOFUNICULUS Liao, 1982

#### Lamprotatus annularis

(Walker, 1833)

Miscogaster annularis Walker, 1833
nigricornis (Fabricius, 1793, *Ichneumon*)
mandibularis (Zetterstedt, 1838, *Pteromalus*)
petiolaris Thomson, 1876

##### Distribution

England

#### Lamprotatus brevicornis

Thomson, 1876

##### Distribution

England, Ireland

#### Lamprotatus claviger

Thomson, 1876

seiunctum (Delucchi, 1953, *Skeloceras*)

##### Distribution

England

#### Lamprotatus crassipes

Thomson, 1876

flavus Delucchi, 1953

##### Distribution

England

#### Lamprotatus novickyi

(Delucchi, 1953)

Skeloceras novickyi Delucchi, 1953
glaucum (Delucchi, 1955, *Skeloceras*)

##### Distribution

England, Scotland, Ireland

#### Lamprotatus picinervis

Thomson, 1876

montanus Delucchi, 1955

#### Lamprotatus pschorni

Delucchi, 1953

##### Distribution

England, Scotland, Ireland

#### Lamprotatus simillimus

Delucchi, 1953

##### Distribution

England, Scotland

#### Lamprotatus socius

(Zetterstedt, 1838)

Pteromalus socius Zetterstedt, 1838
puncticollis Thomson, 1876

##### Distribution

England

#### Lamprotatus splendens

Westwood, 1833

virens (Nees, 1834, *Pteromalus*)
cupreus Delucchi, 1953
ornatus Delucchi, 1953
rusticus Delucchi, 1953

##### Distribution

England, Scotland

##### Notes

**Figure 18.**
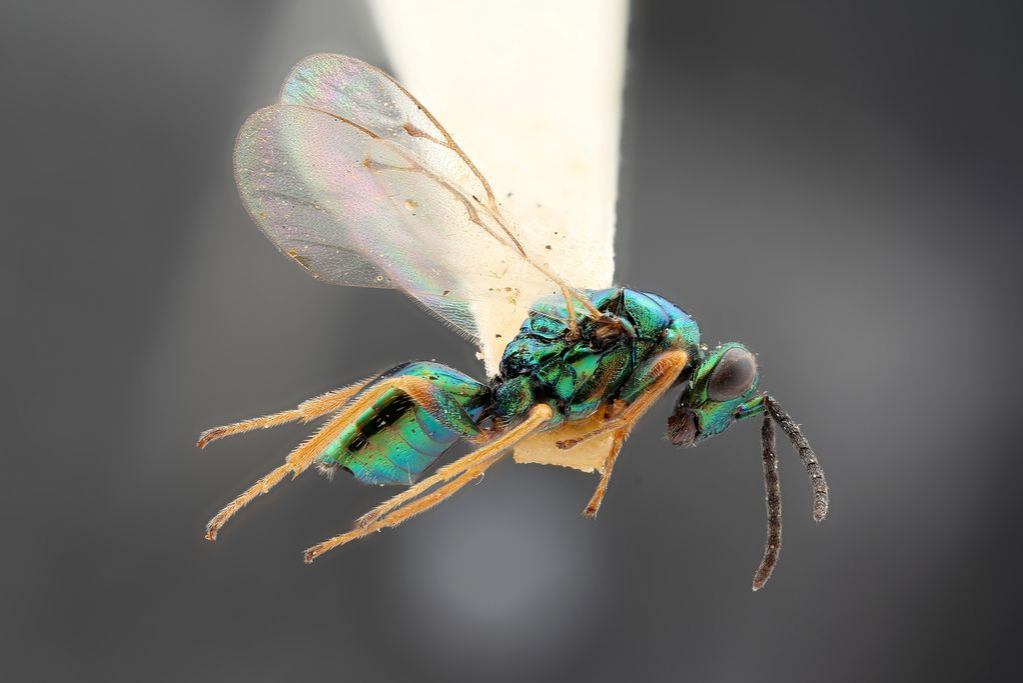
Pteromalidae, Miscogasterinae: *Lamprotatus splendens* Westwood female, BMNH specimen BMNH(E)953746 [/07/1955, Bagley Wood, Berkshire, England] (photo K. McCormack)

See Fig. 18 for habitus

#### Lamprotatus truncatus

(Fonscolombe, 1832)

Cynips truncata Fonscolombe, 1832
cerebrosum (Delucchi, 1955, *Skeloceras*)
mirabile (Delucchi, 1955, *Skeloceras*)

##### Distribution

England

#### Merismus

Walker, 1833

KENTEMA Delucchi, 1953

#### Merismus lasthenes

(Walker, 1848)

Sphegigaster lasthenes Walker, 1848

#### Merismus megapterus

Walker, 1833

clavicornis Walker, 1833
Merismus megapterus ?*tenuicornis* (Walker, 1833, *Miscogaster*)
ovata (Walker, 1833, *Miscogaster*)
agriope (Walker, 1848, *Sphegigaster*)

##### Distribution

England, Scotland, Wales, Ireland

#### Merismus nitidus

(Walker, 1833)

Miscogaster nitida Walker, 1833

##### Distribution

England, Scotland, Wales

#### Merismus rufipes

Walker, 1833

riparius (Nees, 1834, *Chrysolampus*)

##### Distribution

England, Scotland, Wales, Ireland

#### Merismus splendens

Graham, 1969

##### Distribution

England

#### Miscogaster

Walker, 1833

#### Miscogaster elegans

Walker, 1833

punctiger (Nees, 1834, *Chrysolampus*)
helenor (Walker, 1846, *Lamprotatus*)

##### Distribution

England

#### Miscogaster hortensis

Walker, 1833

gracilipes Thomson, 1876
lucens Delucchi, 1953

##### Distribution

England

#### Miscogaster maculata

Walker, 1833

fuscipennis Walker, 1833
Miscogaster maculata ?*maculipes* Walker, 1833
notata Walker, 1833
methymna (Walker, 1848, *Lamprotatus*)
phytomyzae (Ghesquière, 1950, *Stictomischus*)

##### Distribution

England, Scotland, Ireland

#### Miscogaster rufipes

Walker, 1833

fulgens Delucchi, 1953

##### Distribution

England, Scotland, Ireland

#### Nodisoplata

Graham, 1969

#### Nodisoplata diffinis

(Walker, 1874)

Lamprotatus diffinis Walker, 1874
curvus (Thomson, 1876, *Lamprotatus*)
amurensis (Dalla Torre, 1898, *Lamprotatus*)

##### Distribution

England, Scotland, Wales

#### Rhicnocoelia

Graham, 1956

DOGHMIELLA Delucchi, 1962

#### Rhicnocoelia constans

(Walker, 1836)

Pteromalus constans Walker, 1836
cliens (Walker, 1836, *Pteromalus*)
Rhicnocoelia constans ?*archidemus* (Walker, 1839, *Pteromalus*)
Rhicnocoelia constans ?*orsippus* (Walker, 1839, *Pteromalus*)
Rhicnocoelia constans ?*vindalius* (Walker, 1839, *Pteromalus*)
Rhicnocoelia constans ?*labaris* (Walker, 1848, *Lamprotatus*)
Rhicnocoelia constans ?*phalarsarna* (Walker, 1848, *Pteromalus*)
chloris (Thomson, 1876, *Megorismus*)

##### Distribution

England, Scotland

#### Rhicnocoelia coretas

(Walker, 1848)

Lamprotatus coretas Walker, 1848

#### Rhicnocoelia impar

(Walker, 1836)

Pteromalus impar Walker, 1836
Rhicnocoelia impar ?*brevivitta* (Walker, 1836, *Pteromalus*)
crotopus (Walker, 1839, *Pteromalus*)
Rhicnocoelia impar ?*alebion* (Walker, 1848, *Trigonoderus*)
viridis (Delucchi, 1962, *Doghmiella*)

##### Distribution

England, Scotland, Wales, Ireland

#### Schimitschekia

Bouček, 1965

#### Schimitschekia populi

Bouček, 1965

##### Distribution

England

##### Notes

Added by Godfray (1985)

#### Seladerma

Walker, 1834

ISOPLATA Förster, 1856
TELEPSOGOS Delucchi, 1955

#### Seladerma aeneum

(Walker, 1833)

Miscogaster aenea Walker, 1833
nitidipes (Walker, 1833, *Miscogaster*)

##### Distribution

England, Scotland

#### Seladerma annulipes

(Walker, 1833)

Miscogaster annulipes Walker, 1833
Seladerma annulipes ?*dissimile* (Walker, 1833, *Miscogaster*)
Seladerma annulipes ?*venilia* (Walker, 1846, *Lamprotatus*)

#### Seladerma antennatum

(Walker, 1833)

Miscogaster antennata Walker, 1833

##### Distribution

England, Ireland

#### Seladerma berani

(Delucchi, 1953)

Lamprotatus berani Delucchi, 1953

##### Distribution

England

##### Notes

Identification uncertain (Graham 1969a)

#### Seladerma bicolor

Walker, 1834

luteolum Delucchi, 1955

#### Seladerma breve

Walker, 1834

lalage Walker, 1845
Seladerma breve ?*pycnos* (Walker, 1848, *Lamprotatus*)

##### Distribution

England, Scotland, Wales

#### Seladerma caledonicum

Graham, 1969

##### Distribution

Scotland

#### Seladerma convexum

Walker, 1834

agreste Delucchi, 1953

##### Distribution

England

#### Seladerma diffine

(Walker, 1833)

Miscogaster diffinis Walker, 1833
Seladerma diffine ?*lucidum* (Walker, 1833, *Miscogaster*)
viridis (Walker, 1833, *Miscogaster*)
mazoeus (Walker, 1844, *Lamprotatus*)
amulius (Walker, 1848, *Lamprotatus*)

##### Distribution

England, Scotland, Wales, Ireland

#### Seladerma euroto

(Walker, 1839)

Miscogaster euroto Walker, 1839

#### Seladerma gelanor

(Walker, 1840)

Miscogaster gelanor Walker, 1840

#### Seladerma genale

(Thomson, 1876)

Lamprotatus genalis Thomson, 1876

##### Notes

Identification uncertain (Graham (1969a), based on a male specimen

#### Seladerma geniculatum

(Zetterstedt, 1838)

Entedon geniculatus Zetterstedt, 1838
parvulus (Zetterstedt, 1838, *Pteromalus*)
celer (Förster, 1841, *Pteromalus*)
platynotus (Förster, 1841, *Pteromalus*)

##### Distribution

England

#### Seladerma laetum

Walker, 1834

Seladerma laetum ?*dryops* (Walker, 1840, *Miscogaster*)
nobile Delucchi, 1955
violaceum Delucchi, 1955

##### Distribution

England, Scotland, Wales

#### Seladerma parviclava

(Thomson, 1876)

Lamprotatus parviclava Thomson, 1876

#### Seladerma sabbas

(Walker, 1848)

Ormocerus sabbas Walker, 1848
gracilis (Thomson, 1876, *Lamprotatus*)

#### Seladerma saurus

Walker, 1844

#### Seladerma scaea

(Walker, 1844)

Lamprotatus scaea Walker, 1844

#### Seladerma scoticum

(Walker, 1833)

Miscogaster scotica Walker, 1833

##### Distribution

England, Scotland, Ireland

#### Seladerma simplex

(Thomson, 1876)

Lamprotatus simplex Thomson, 1876

##### Distribution

England, Wales

#### Seladerma tarsale

(Walker, 1833)

Miscogaster tarsalis Walker, 1833
scotica (Walker, 1833, *Cyrtogaster*) preocc.
apicalis (Walker, 1833, *Miscogaster*)
brevis (Walker, 1833, *Miscogaster*)
contigua (Walker, 1833, *Miscogaster*)
costalis (Walker, 1833, *Miscogaster*)
cyanea (Walker, 1833, *Miscogaster*)
Seladerma tarsale ?*femoratum* (Walker, 1833, *Miscogaster*)
filicornis (Walker, 1833, *Miscogaster*)
linearis (Walker, 1833, *Miscogaster*)
philochortoides (Walker, 1833, *Miscogaster*)
semiaurata (Walker, 1833, *Miscogaster*)
tristis (Walker, 1833, *Miscogaster*)
brises (Walker, 1844, *Lamprotatus*)
cleta (Walker, 1844, *Lamprotatus*)
leucon (Walker, 1844, *Lamprotatus*)
bolgius (Walker, 1848, *Lamprotatus*)
oebares (Walker, 1848, *Lamprotatus*)
pilicornis (Thomson, 1876, *Lamprotatus*)

##### Distribution

England, Scotland

#### Sphaeripalpus

Förster, 1841

GITOGNATHUS Thomson, 1876

#### Sphaeripalpus fuscipes

(Walker, 1833)

Miscogaster fuscipes Walker, 1833
babilus (Walker, 1846, *Lamprotatus*)
rubrius (Walker, 1846, *Lamprotatus*)

##### Distribution

England

#### Sphaeripalpus microstolus

(Graham, 1969)

Gitognathus microstolus Graham, 1969

##### Distribution

England

#### Sphaeripalpus viridis

Förster, 1841

zipoetes (Walker, 1848, *Lamprotatus*)
grandiclava (Thomson, 1876, *Gitognathus*)
kerrichi (Delucchi, 1953, *Lamprotatus*)
gibberosus (Delucchi, 1955, *Gitognathus*)

##### Distribution

England

#### Stictomischus

Thomson, 1876

#### Stictomischus gibbus

(Walker, 1833)

Miscogaster gibba Walker, 1833
lagenarius (Nees, 1834, *Chrysolampus*)
phyllochlorus (Förster, 1841, *Chrysolampus*)
sublaevis (Förster, 1841, *Chrysolampus*)
pleuralis Thomson, 1876

##### Distribution

England, Scotland, Wales

#### Stictomischus groschkei

Delucchi, 1953

##### Distribution

England, Scotland, Wales

#### Stictomischus nitentis

Delucchi, 1955

lamprosomus Graham, 1969

##### Distribution

Scotland

#### Stictomischus obscurus

(Walker, 1833)

Miscogaster obscura Walker, 1833
chrysochlora (Walker, 1833, *Miscogaster*)
obscuripennis (Walker, 1833, *Miscogaster*)
splendens (Förster, 1841, *Chrysolampus*)
subquadratus (Förster, 1841, *Chrysolampus*)
mallius (Walker, 1848, *Lamprotatus*)

##### Distribution

England, Scotland

#### Stictomischus scaposus

Thomson, 1876

##### Distribution

England, Scotland

#### Stictomischus tumidus

(Walker, 1833)

Miscogaster tumida Walker, 1833
rugicollis Thomson, 1876

##### Distribution

England

#### Telepsogina

Hedqvist, 1958

#### Telepsogina adelognathi

Hedqvist, 1958

##### Distribution

Isle of Man

#### Thinodytes

Graham, 1956

#### Thinodytes cyzicus

(Walker, 1839)

Miscogaster cyzicus Walker, 1839

##### Distribution

England, Wales

#### Tricyclomischus

Graham, 1956

#### Tricyclomischus celticus

Graham, 1956

##### Distribution

England, Ireland

#### Xestomnaster

Delucchi, 1955

#### Xestomnaster chrysochlorus

(Walker, 1846)

Lamprotatus chrysochlorus Walker, 1846
mirificus (Delucchi, 1953, *Lamprotatus*)
parkeri (Delucchi, 1953, *Lamprotatus*)
smaragdus (Delucchi, 1953, *Lamprotatus*)

##### Distribution

England, Scotland

#### Xestomnaster mazares

(Walker, 1844)

Seladerma mazares Walker, 1844

##### Distribution

England

##### Notes

BMNH, det. Bouček, added here

#### Neodiparinae

Bouček, 1961

#### Neodipara

Erdös, 1955

#### Neodipara masneri

Bouček, 1961

#### Ormocerinae

Walker, 1833

#### Bugacia

Erdös, 1946

#### Bugacia arenaria

Erdös, 1946

##### Distribution

England

#### Bugacia classeyi

Bouček, 1965

##### Distribution

England

#### Bugacia submontana

Bouček, 1956

##### Distribution

England

#### Melancistrus

Graham, 1969

#### Melancistrus mucronatus

(Thomson, 1876)

Tridymus mucronatus Thomson, 1876

##### Distribution

England

#### Melancistrus specularis

Graham, 1969

##### Distribution

Scotland

#### Ormocerus

Walker, 1834

HORMOCERUS Förster, 1856
TEROBIA Förster, 1878

#### Ormocerus latus

Walker, 1834

##### Distribution

England, Wales

#### Ormocerus vernalis

Walker, 1834

dispila (Förster, 1878, *Terobia*)

##### Distribution

England, Wales

#### Semiotellus

Westwood, 1839

SEMIOTUS Walker, 1834 preocc.
STICTONOTUS Förster, 1856
CHEIROPACHYSIA Girault, 1915
NEOSYSTASIS Girault, 1915

#### Semiotellus diversus

(Walker, 1834)

Semiotus diversus Walker, 1834

##### Distribution

England, Scotland

#### Semiotellus fumipennis

Thomson, 1876

#### Semiotellus laevicollis

Thomson, 1876

#### Semiotellus mundus

(Walker, 1834)

Semiotus mundus Walker, 1834
clarus (Walker, 1834, *Semiotus*)
maerens (Walker, 1834, *Semiotus*)
praestans (Walker, 1834, *Semiotus*)
Semiotellus mundus ?*quadratus* (Walker, 1834, *Semiotus*)
scoticus (Walker, 1834, *Semiotus*)
tarsalis (Walker, 1834, *Semiotus*)
varians (Walker, 1834, *Semiotus*)
japis (Walker, 1839, *Pteromalus*)
tauriscus (Walker, 1848, *Semiotus*)
puncticollis Thomson, 1876

##### Distribution

England, Scotland

#### Systasis

Walker, 1834

GUIERALIA Risbec, 1951
PARURIELLA Girault, 1913

#### Systasis angustula

Graham, 1969

#### Systasis annulipes

(Walker, 1834)

Gastrancistrus annulipes Walker, 1834
bambyce (Walker, 1839, *Ormocerus*)
clavicornis Bouček, 1956

##### Distribution

England, Wales

#### Systasis encyrtoides

Walker, 1834

geniculatus (Nees, 1834, *Pteromalus*)
punctatus (Ratzeburg, 1852, *Tridymus*)
impletus (Walker, 1872, *Hormocerus*)
longicornis Thomson, 1876

##### Distribution

England

#### Systasis parvula

Thomson, 1876

##### Distribution

England, Ireland

#### Systasis tenuicornis

Walker, 1834

##### Distribution

England

#### Pireninae

Halliday, 1844

#### Ecrizotes

Förster, 1861

HENICETRUS Thomson, 1876

#### Ecrizotes filicornis

(Thomson, 1876)

Henicetrus filicornis Thomson, 1876

##### Distribution

England, Scotland

#### Ecrizotes longicornis

(Walker, 1848)

Gastrancistrus longicornis Walker, 1848

##### Distribution

England, Scotland, Wales

#### Ecrizotes monticola

Förster, 1861

annellus (Thomson, 1876, *Henicetrus*)

#### Gastrancistrus

Westwood, 1833

GLYPHE Walker, 1834
MEROMALUS Walker, 1834
STOMOCTEA Dufour, 1846
TRIDYMUS Ratzeburg, 1848
TRIPEDIAS Förster, 1856
STIGMATOCREPIS Ashmead, 1904
AMUSCIDEA Girault, 1913
ISOPLATA Girault, 1913
ROPTROCEROPSEUS Girault, 1913
EROTOLEPSIOPUS Girault, 1915
MUSCIDEOMYIA Girault, 1915
PAREROTOLEPSIA Girault, 1915
PROPLESIOSTIGMA Girault, 1915
PARECRIZOTES Girault, 1916
PARASYNTOMOCERA Girault, 1917
ISOPLATELLA Gahan & Fagan, 1923
MESECRIZOTES De Santis, 1968

#### Gastrancistrus acontes

Walker, 1840

convergens (Thomson, 1876, *Tridymus*)

#### Gastrancistrus acutus

Walker, 1834

angulus Walker, 1834
panares Walker, 1844
loelianus Walker, 1846

##### Distribution

England, Scotland, Wales

#### Gastrancistrus aequus

Graham, 1969

#### Gastrancistrus affinis

Graham, 1969

##### Distribution

England, Scotland

#### Gastrancistrus alectus

Walker, 1848

##### Distribution

England

#### Gastrancistrus amaboeus

Walker, 1848

#### Gastrancistrus atropurpureus

Walker, 1834

##### Distribution

England

#### Gastrancistrus autumnalis

(Walker, 1834)

Glyphe autumnalis Walker, 1834
productus (Thomson, 1876, *Tridymus*)

##### Distribution

England, Ireland

#### Gastrancistrus clavatus

(Thomson, 1876)

Tridymus clavatus Thomson, 1876

##### Distribution

England

#### Gastrancistrus clavellatus

Graham, 1969

#### Gastrancistrus coactus

Graham, 1969

##### Distribution

England, Scotland

#### Gastrancistrus compressus

Walker, 1834

pallicornis (Thomson, 1876, *Tridymus*) preocc.
thomsonii Dalla Torre, 1898

##### Distribution

England, Wales

#### Gastrancistrus coniferae

Graham, 1969

##### Distribution

England, Scotland

#### Gastrancistrus consors

Graham, 1969

##### Distribution

England, Scotland

#### Gastrancistrus crassus

Walker, 1834

##### Distribution

England, Scotland, Ireland

#### Gastrancistrus cupreus

Graham, 1969

##### Distribution

England

#### Gastrancistrus dispar

Graham, 1969

##### Distribution

England, Wales

#### Gastrancistrus flavicornis

(Walker, 1834)

Meromalus flavicornis Walker, 1834
Gastrancistrus flavicornis ?*drymo* (Walker, 1839, *Ormocerus*)

##### Distribution

England

##### Notes

A species of uncertain status.

#### Gastrancistrus fulvicornis

(Walker, 1874)

Lamprotatus fulvicornis Walker, 1874

##### Distribution

England, Scotland, Wales

#### Gastrancistrus fulvicoxis

Graham, 1969

##### Distribution

England, Ireland

#### Gastrancistrus fumipennis

Walker, 1834

##### Distribution

England

#### Gastrancistrus fuscicornis

Walker, 1834

##### Distribution

England

#### Gastrancistrus glabellus

(Nees, 1834)

Eulophus glabellus Nees, 1834
laeviscuta (Thomson, 1876, *Tridymus*)

##### Distribution

England, Scotland

#### Gastrancistrus hamillus

Walker, 1848

circinantis (Rondani, 1874, *Pteromalus*)
flavipes (Thomson, 1876, *Tridymus*)

##### Distribution

England

#### Gastrancistrus hemigaster

Graham, 1969

##### Distribution

England, Scotland, Ireland

#### Gastrancistrus hirtulus

Graham, 1969

##### Distribution

England

#### Gastrancistrus indivisus

Graham, 1969

##### Distribution

England, Scotland

#### Gastrancistrus laticeps

Graham, 1969

##### Distribution

England

#### Gastrancistrus laticornis

Walker, 1834

tenuicornis Walker, 1834

##### Distribution

England, Scotland, Wales

#### Gastrancistrus latifrons

(Thomson, 1876)

Tridymus latifrons Thomson, 1876

##### Distribution

England

#### Gastrancistrus lativentris

Graham, 1969

##### Distribution

Scotland

#### Gastrancistrus longigena

Graham, 1969

##### Distribution

England

#### Gastrancistrus obscurellus

Walker, 1834

##### Distribution

England

#### Gastrancistrus oporinus

Graham, 1969

##### Distribution

England, Ireland

#### Gastrancistrus picipes

(Nees, 1834)

Pteromalus picipes Nees, 1834
walkeri Graham, 1969

##### Distribution

England

#### Gastrancistrus piricola

(Marchal, 1907)

Tridymus piricola Marchal, 1907

##### Distribution

England, Wales

#### Gastrancistrus praecox

Graham, 1969

fulvicornis (Walker, 1874, *Semiotus*)

##### Distribution

England

#### Gastrancistrus puncticollis

(Thomson, 1876)

Tridymus puncticollis Thomson, 1876

##### Distribution

England

#### Gastrancistrus pusztensis

(Erdös, 1946)

Meromalus pusztensis Erdös, 1946
tripedias Bouček, 1964

##### Distribution

Scotland

#### Gastrancistrus salicis

(Nees, 1834)

Pteromalus salicis Nees, 1834

##### Distribution

England, Ireland

#### Gastrancistrus terminalis

Walker, 1834

##### Distribution

England

#### Gastrancistrus torymiformis

(Ratzeburg, 1852)

Tridymus torymiformis Ratzeburg, 1852

##### Distribution

England

#### Gastrancistrus triandrae

Graham, 1969

##### Distribution

England

#### Gastrancistrus unicolor

Walker, 1834

frenalis (Thomson, 1876, *Tridymus*)

#### Gastrancistrus vagans

Westwood, 1833

tenebricosus Walker, 1834
pacilus Walker, 1848

#### Gastrancistrus venustus

Graham, 1969

##### Distribution

England

#### Gastrancistrus vernalis

Graham, 1969

##### Distribution

England

#### Gastrancistrus viridis

Walker, 1834

dryas Walker, 1839

##### Distribution

England

#### Gastrancistrus vulgaris

Walker, 1834

##### Distribution

England, Scotland

#### Macroglenes

Westwood, 1832

PIRENE Haliday, 1833
CORYNOCERE Nees, 1834
STENOPHRUS Förster, 1841
CALYPSO Haliday, 1844
EURYOPHRYS Förster, 1856
PHOCION Girault, 1925
PYRENISCA Ghesquière, 1946

#### Macroglenes bouceki

(Graham, 1969)

Pirene bouceki Graham, 1969

##### Distribution

England

#### Macroglenes chalybeus

(Haliday, 1833)

Pirene chalybea Haliday, 1833
rubi (Haliday, 1844, *Pirene*)
scylax (Walker, 1848, *Pirene*)

##### Distribution

England, Wales

#### Macroglenes compressus

Förster, 1841

umbellatarum (Haliday, 1844, *Macroglenes*)
serratulae (Haliday, 1844, *Calypso*)
occultus (Thomson, 1876, *Macroglenes*)

##### Distribution

Ireland

#### Macroglenes conjungens

(Graham, 1969)

Pirene conjungens Graham, 1969

##### Distribution

England, Wales

#### Macroglenes eximius

(Haliday, 1833)

Pirene eximia Haliday, 1833

##### Distribution

England

#### Macroglenes gramineus

(Haliday, 1833)

Pirene graminea Haliday, 1833

##### Distribution

England, Scotland, Wales, Ireland

#### Macroglenes herbaceus

(Graham, 1969)

Pirene herbacea Graham, 1969

##### Distribution

England

#### Macroglenes microcerus

Haliday, 1844

##### Distribution

England

#### Macroglenes paludum

(Graham, 1969)

Pirene paludum Graham, 1969

##### Distribution

England, Scotland

#### Macroglenes penetrans

(Kirby, 1800)

Ichneumon penetrans Kirby, 1800
oculatus Westwood, 1832
brevicornis Thomson, 1876
decipiens Graham, 1969 (*Pirene*)

##### Distribution

England

#### Macroglenes varicornis

(Haliday, 1833)

Pirene varicornis Haliday, 1833
deplana (Nees, 1834, *Corynocere*)

##### Distribution

England, Scotland

#### Micradelus

Walker, 1834

##### Notes

Included here in Pireninae as the tribe Micradelini was placed in the Tridymine group (Graham 1969a); most of Tridyminae were transferred to the Pireninae by Bouček (1988).

#### Micradelus acutus

Graham, 1969

##### Distribution

England

#### Micradelus rotundus

Walker, 1834

##### Distribution

England, Scotland, Wales

#### Spathopus

Ashmead, 1904

#### Spathopus nasalis

Springate & Noyes, 1990

##### Distribution

Wales

##### Notes

Added by Springate and Noyes (1990)

#### Termolampa

Bouček, 1961

HABRITOIDES Szelényi, 1981

##### Notes

Included here in Pireninae as the tribe Termolampini was described in the Tridyminae (Bouček 1961), most of which were transferred to the Pireninae by Bouček (1988).

#### Termolampa pinicola

Bouček, 1961

perardens (Szelényi, 1981, *Habritoides*)

##### Distribution

England

##### Notes

Added by Askew (1992c)

#### Spalangiinae

Halliday, 1833

#### Spalangia

Latreille, 1805

PROSPALANGIA Brèthes, 1915

#### Spalangia cameroni

Perkins, 1910

philippinensis Fullaway, 1917
melanogastra Masi, 1940
atherigonae Risbec, 1951

##### Distribution

England

#### Spalangia crassicornis

Bouček, 1963

#### Spalangia erythromera

Förster, 1850

spuria Förster, 1850
umbellatarum Förster, 1850

##### Distribution

England

#### Spalangia nigra

Latreille, 1805

hirta Haliday, 1833
rugosicollis Ashmead, 1894

##### Distribution

England

##### Notes

**Figure 25.**
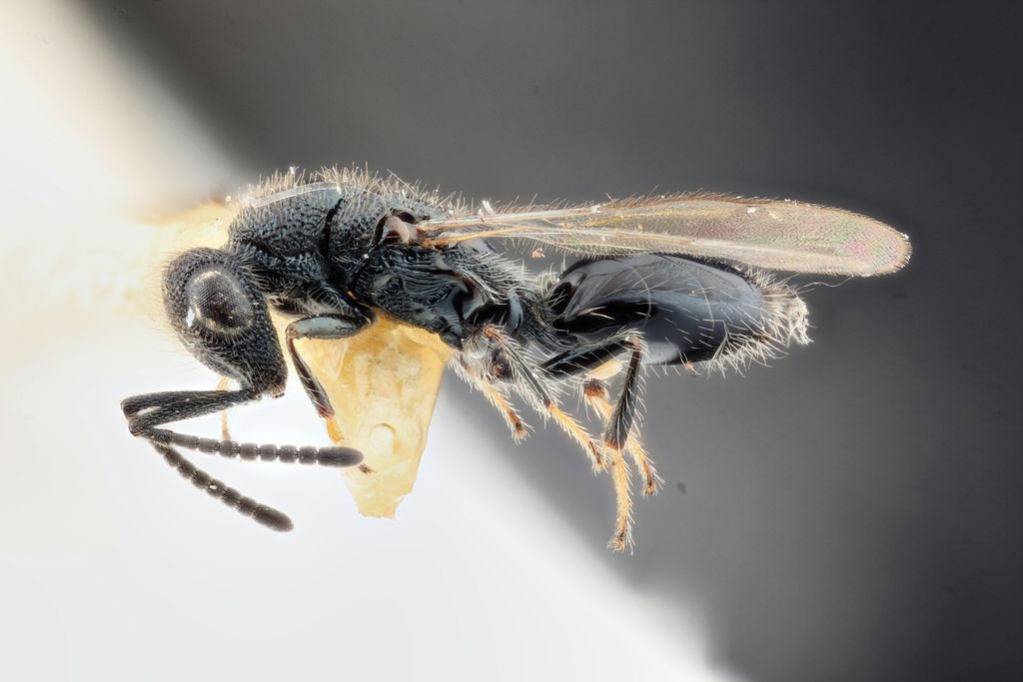
Pteromalidae, Spalangiinae: *Spalangia nigra* Latreille female, BMNH specimen BMNH(E)1414553 [/07/1935, Storey's Way, Cambridge, Cambridgeshire, England, coll. Varley] (photo K. McCormack)

See Fig. 25 for habitus

#### Spalangia nigripes

Curtis, 1839

formicaria Kieffer, 1905
muscarum Girault, 1920
hyaloptera Förster, 1850

#### Spalangia nigroaenea

Curtis, 1839

homalaspis Förster, 1850
astuta Förster, 1851
muscidarum Richardson, 1913
platensis (Brèthes, 1915, *Prospalangia*)
abenabooi Girault, 1932
sundaica Graham, 1932
mors Girault, 1933

#### Spalangia rugulosa

Förster, 1850

##### Distribution

England

#### Spalangia subpunctata

Förster, 1850

leptogramma Förster, 1850

### Family Signiphoridae Howard, 1894

#### Chartocerus

Motschulsky, 1859

MATRITIA Mercet, 1916
XANA Kurdjumov, 1917
SIGNIPHORINA Nikol'skaya, 1950
NEOCALES Risbec, 1957

#### Chartocerus subaeneus

(Förster, 1878)

Chartocerus subaeneus Förster, 1878
mala (Nikol'skaya, 1950, *Signiphorina*)

#### Thysanus

Walker, 1840

NEOSIGNIPHORA Rust, 1913
PLASTOCHARIS Förster, 1856
TRIPHASIUS Förster, 1856

#### Thysanus ater

Walker, 1840

##### Distribution

England

### Family Tetracampidae Förster, 1856

#### Platynocheilinae

Förster, 1856

#### Platynocheilus

Westwood, 1837

PTERONCOMA Förster, 1841

#### Platynocheilus cuprifrons

(Nees, 1834)

Pteromalus cuprifrons Nees, 1834
erichsonii Westwood, 1837
derceto (Walker, 1839, Stenocera)
linearis (Förster, 1841, Pteroncoma)

##### Distribution

England, Scotland, Ireland

##### Notes

**Figure 26.**
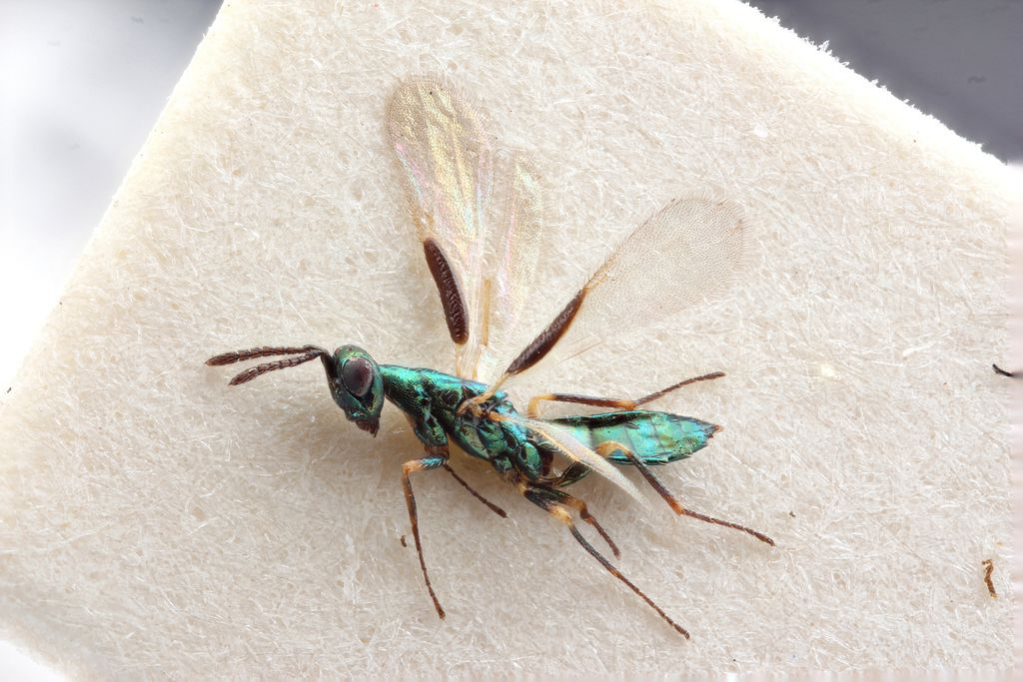
Tetracampidae, Platynocheilinae: *Platynocheilus cuprifrons* (Nees) male, BMNH specimen BMNH(E)953747 [/07/1969, Southgate, London, England, coll. Graham] (photo K. McCormack)

See Fig. 26 for habitus

#### Tetracampinae

Bouček, 1958

#### Dipriocampe

Bouček, 1957

#### Dipriocampe diprioni

(Ferrière, 1935)

Tetracampe diprioni Ferrière, 1935

##### Distribution

England

#### Epiclerus

Haliday, 1844

DIPARELLOMYIA Girault, 1913

#### Epiclerus nomocerus

(Masi, 1934)

Tetracampe nomocera Masi, 1934

##### Distribution

England

##### Notes

Askew coll., det. Askew, added here

#### Epiclerus panyas

(Walker, 1839)

Entedon panyas Walker, 1839
viridulus (Rondani, 1874, *Heptomerus*)

##### Distribution

England

#### Epiclerus temenus

(Walker, 1839)

Entedon temenus Walker, 1839

##### Distribution

England, Ireland

#### Foersterella

Dalla Torre, 1897

Hyperbius Förster, 1878 preocc.

#### Foersterella erdoesi

Bouček, 1958

##### Distribution

England

#### Foersterella reptans

(Nees, 1834)

Pteromalus reptans Nees, 1834
flavipes (Förster, 1841, *Tetracampe*)

##### Distribution

England

#### Tetracampe

Förster, 1841

#### Tetracampe impressa

Förster, 1841

##### Distribution

England

### Family Torymidae Walker, 1833

#### Megastiminae

Thomson, 1876

#### Bootanomyia

Girault, 1915

EPIBOOTANIA Girault, 1937

##### Notes

These species were transferred from *Megastigmus* by Doğanlar (2011)

#### Bootanomyia dorsalis

(Fabricius 1798)

Ichneumon dorsalis Fabricius, 1798
bohemanii Ratzeburg, 1848
xanthopygus Förster, 1859

##### Distribution

England

#### Bootanomyia stigmatizans

(Fabricius 1798)

Ichneumon stigmatizans Fabricius, 1798
stigma (Fabricius, 1804)
puparum (Schmidt, 1851)
giganteus Walker, 1852

##### Distribution

England

#### Megastigmus

Dalman, 1820

CYCLONEURON Dahlbom, 1857
TROGOCARPUS Rondani, 1877
MEGALOSTIGMUS Schulz, 1906
XANTHOSOMOIDES Girault, 1913
EUMEGASTIGMUS Hussey, 1956

#### Megastigmus aculeatus

(Swederus 1795)

Pteromalus aculeatus Swederus, 1795
transversus Walker, 1833
collaris (Boheman, 1834, *Torymus*)
punctum (Förster, 1841, *Torymus*)
vexillum Ratzeburg, 1848
flavus Förster, 1859
cynorrhodi Perris, 1876

##### Distribution

England

##### Notes

**Figure 27.**
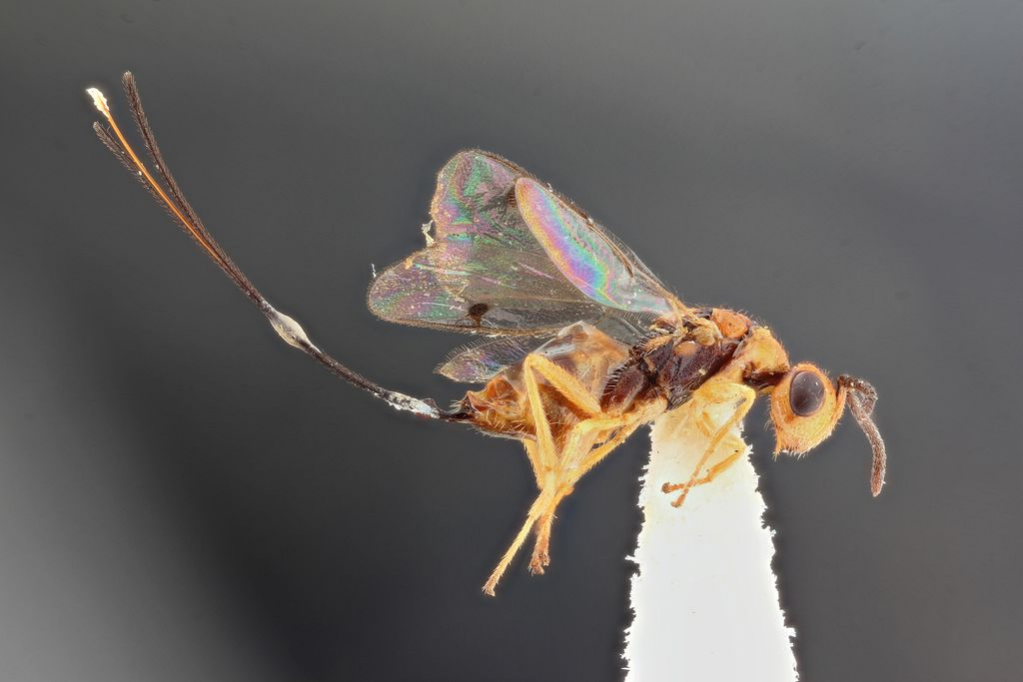
Torymidae, Megastigminae: *Megastigmus aculeatus* (Swederus) female, BMNH specimen BMNH(E)953641 [/07/1953, Northwood, Middlesex, England, coll. Quinlan] (photo K. McCormack)

See Fig. 27 for habitus

#### Megastigmus atedius

Walker, 1851

piceae Rohwer, 1915
zwoelferi Schefer-Immel, 1957

##### Distribution

England

##### Notes

Accidental introduction from North America and apparently established, developing in seeds of *Pinus* and *Picea* conifers (Roques and Skrzypczyńska 2003).

#### Megastigmus bipunctatus

(Swederus 1795)

Pteromalus bipunctatus Swederus, 1795
erythrothorax (Nees, 1834)
microspilus Thomson, 1876
kuntzei Kapuscinski, 1946

#### Megastigmus brevicaudis

Ratzeburg, 1852

##### Distribution

England

##### Notes

Added by Askew and Shaw (1979b)

#### Megastigmus milleri

Milliron, 1949

##### Notes

Accidental introduction from North America; has been confused under *pinus* (Roques and Skrzypczyńska 2003).

#### Megastigmus pictus

(Förster, 1841)

Torymus pictus Förster, 1841
seitneri Hoffmeyer, 1929

##### Distribution

England, Ireland

##### Notes

An established accidental introduction from Eurasia, developing in the seeds of non-native *Larix* (Roques and Skrzypczyńska 2003).

#### Megastigmus pinus

Parfitt, 1857

##### Distribution

Ireland

#### Megastigmus rafni

Hoffmeyer, 1929

##### Notes

An accidental introduction from North America, developing in the seeds of *Abies* (Roques and Skrzypczyńska 2003).

#### Megastigmus spermotrophus

Wachtl, 1893

##### Distribution

England, Scotland, Ireland

##### Notes

An accidental introduction from North America, developing in the seeds of *Pseudotsuga* (Roques and Skrzypczyńska 2003).

#### Megastigmus strobilobius

Ratzeburg, 1848

abietis Seitner, 1916

##### Notes

An accidental introduction from Eurasia, developing in the seeds of *Picea* (Roques and Skrzypczyńska 2003).

#### Megastigmus suspectus

Borries, 1895

bornmuellerianus Hussey, 1957
piceae Seitner, 1916

##### Notes

An accidental introduction from Eurasia, developing in the seeds of *Abies* (Roques and Skrzypczyńska 2003).

#### Toryminae

Walker, 1833

#### Monodontomerini

Ashmead, 1899

#### Monodontomerus

Westwood, 1833

PAROLIGOSTHENUS Cameron, 1913

#### Monodontomerus aeneus

(Fonscolombe 1832)

Cynips aenea Fonscolombe, 1832
punctatus (Geoffroy, 1785, *Cynips*)
obsoletus (Fabricius, 1798, *Ichneumon*)
nitidus Newport, 1849
retusa Newport, 1850
vacillans Förster, 1860
punctatus Thomson, 1876

##### Distribution

England

#### Monodontomerus aereus

Walker, 1834

anephelus (Ratzeburg, 1844, *Torymus*)
cupreus Fabre, 1886
kashmiricus Narendran, 1994

#### Monodontomerus obscurus

Westwood, 1833

pubescens (Walker, 1833, *Callimome*)
dresdensis (Ratzeburg, 1844, *Torymus*)
metallicus (Ratzeburg, 1844, *Torymus*)
anthophorae Walker, 1852
intermedius Förster, 1860
trichiopthalmus (Cameron, 1913, *Paroligosthenus*)
masii Hoffmeyer, 1929

##### Distribution

England

#### Monodontomerus rugulosus

Thomson, 1876

gladiatus Steffan, 1962

##### Distribution

England

#### Monodontomerus vicicellae

(Walker 1847)

Torymus vicicellae Walker, 1847
nubecula Rondani, 1877

##### Distribution

England

#### Torymini

Walker, 1833

#### Torymus

Dalman, 1820

CALLIMOME Spinola, 1811
MISOCAMPE Latreille, 1818
MISOCAMPUS Stephens, 1829
DIOMORUS Walker, 1834
SYNTOMASPIS Förster, 1856
LIOTERPHUS Thomson, 1876
CALLIMOMUS Thomson, 1876
NANNOCERUS Mayr, 1885
HEMITORYMUS Ashmead, 1904
DIHOMERUS Schulz, 1906
PARASYMPIESIS Brèthes, 1927

##### Notes

Doubtfully placed species of *Torymus*:

[*vallisnierii* Cameron, 1901 nom. dub., from England, Scotland]

Species of *Torymus* excluded from the British and Irish list:

[*socius* Mayr, 1874] - Recorded as new to Britain by Graham (1969b), identified by reference to the original description, but not listed as occurring in Britain in Graham and Gijswijt (1998).

#### Torymus aceris

Bouček, 1994

##### Distribution

England

##### Notes

Added by Bouček (1994)

#### Torymus affinis

(Fonscolombe 1832)

Cynips affinis Fonscolombe, 1832
apicalis (Walker, 1833, *Callimome*)
fuscipennis (Walker, 1833, *Callimome*)
littoralis (Walker, 1833, *Callimome*)
tarsalis (Walker, 1833, *Callimome*)
caudatus Nees, 1834
saphirinus Boheman, 1834
admirabilis Förster, 1841
crinicaudis Ratzeburg, 1844
sapphyrina (Dalla Torre, 1898, *Syntomaspis*)

##### Distribution

England

#### Torymus amurensis

(Walker 1874)

Callimome amurensis Walker, 1874

##### Distribution

England, Ireland

#### Torymus angelicae

(Walker 1836)

Callimome angelicae Walker, 1836
abdominalis Boheman, 1834 preocc.

##### Distribution

Ireland

##### Notes

Listed as a synonym of *T. cingulatus* by Bouček and Graham (1978)

#### Torymus armatus

Boheman, 1834

nobilis (Walker, 1834)

##### Distribution

England, Ireland

#### Torymus arundinis

(Walker 1833)

Callimome arundinis Walker, 1833
compactus (Walker, 1834, *Callimome*)
lasiopterae (Giraud, 1863, *Callimome*)
bohemani Thomson, 1876
bohemanii Dalla Torre, 1898
antipai Andriescu, 1971

##### Distribution

England

#### Torymus auratus

(Müller 1764)

Cynips aurata Müller, 1764
nigricornutus (Christ, 1791, *Cynipsichneumon*)
rubicornutus (Christ, 1791, *Cynipsichneumon*)
nigricornutus (Christ, 1791, *Diplolepis*)
nitens (Walker, 1833, *Callimome*)
inconstans (Walker, 1834, *Callimome*)
lateralis (Walker, 1834, *Callimome*)
regius Nees, 1834
incertus Förster, 1841
longicaudis Ratzeburg, 1844
amyrius (Walker, 1846, *Callimome*)
devoniensis (Parfitt, 1856, *Callimome*)
flavipes (Parfitt, 1856, *Callimome*)

##### Distribution

England, Scotland

#### Torymus azureus

Boheman, 1834

chalybaeus Ratzeburg, 1844
erdoesi (Györfi, 1945, *Callimome*)

##### Distribution

England, Scotland

##### Notes

**Figure 28.**
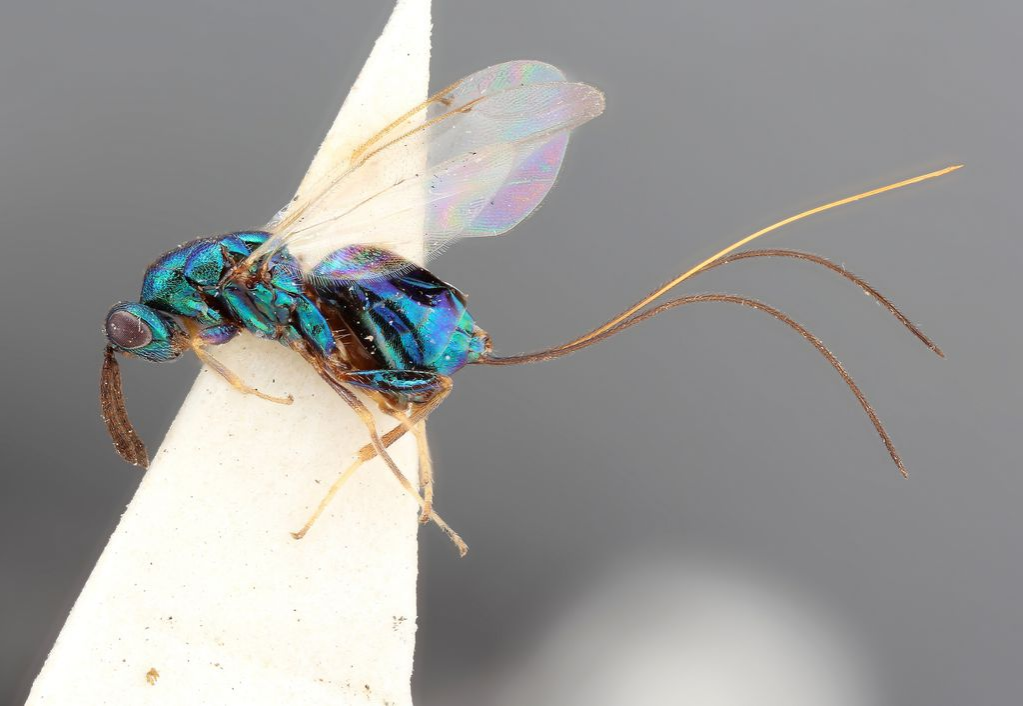
Torymidae, Toryminae: *Torymus azureus* Boheman female, BMNH specimen BMNH(E)953644 [/07/1955, Ballater, Aberdeenshire, Scotland] (photo K. McCormack)

See Fig. 28 for habitus

#### Torymus basalis

(Walker 1833)

Callimome basalis Walker, 1833
viridiaeneus (Walker, 1833, *Callimome*)

##### Distribution

England, Ireland

#### Torymus baudysi

Bouček, 1954

##### Distribution

England

#### Torymus bedeguaris

(Linnaeus 1758)

Ichneumon bedeguaris Linnaeus, 1758
viridis (Geoffroy, 1785, *Cynips*)
rosaeaurata (Christ, 1791, *Cynips*)
Torymus bedeguaris ?*pretiosus* (Walker, 1833, *Callimome*)
elegans Boheman, 1834
foersteri Ratzeburg, 1844
divisus (Walker, 1871, *Callimome*)
rosarum (Hoffmeyer, 1929, *Callimome*)

##### Distribution

England

#### Torymus boops

Graham, 1994

##### Notes

Added by Jennings (2004)

#### Torymus brachyurus

Boheman, 1834

##### Notes

Added by Graham and Gijswijt (1998)

#### Torymus breviscapus

Graham & Gijswijt, 1998

##### Distribution

England

##### Notes

Added by Graham and Gijswijt (1998)

#### Torymus caledonicus

Graham & Gijswijt, 1998

##### Distribution

Scotland

##### Notes

Added by Graham and Gijswijt (1998)

#### Torymus caudatulus

Graham & Gijswijt, 1998

##### Distribution

England

##### Notes

Added by Graham and Gijswijt (1998)

#### Torymus caudatus

Boheman, 1834

distinctus Förster, 1841

#### Torymus centor

Graham & Gijswijt, 1998

##### Distribution

England

##### Notes

Added by Graham and Gijswijt (1998)

#### Torymus chlorocopes

Boheman, 1834

#### Torymus chloromerus

(Walker 1833)

Callimome chloromerus Walker, 1833
abdominalis (Walker, 1833, *Callimome*)
euphorbiae (Walker, 1833, *Callimome*)
micropterus (Walker, 1833, *Callimome*)
abbreviatus Boheman, 1834
cyanimus Boheman, 1834
chlorinus Förster, 1841
hieracii Mayr, 1874
campanulae Cameron, 1880
britannicus Dalla Torre, 1898
euphorbiae Ruschka, 1921
tilicola Ruschka, 1921
centaureae (Hoffmeyer, 1930, *Callimome*)

##### Distribution

England

#### Torymus chrysocephalus

Boheman, 1834

##### Distribution

Scotland, Wales

#### Torymus cingulatus

Nees, 1834

aeneus Nees, 1834
medius Förster, 1841
glechomae Mayr, 1874

##### Distribution

England

#### Torymus confinis

(Walker 1833)

Callimome confinis Walker, 1833
curtus (Walker, 1833, *Callimome*)
inconspectus (Walker, 1833, *Callimome*)
urticae (Perris, 1841, *Cynips*)
difficilis Ratzeburg, 1844

##### Distribution

England

#### Torymus corni

Mayr, 1874

##### Notes

Added by Graham and Gijswijt (1998)

#### Torymus cultriventris

Ratzeburg, 1844

##### Notes

Added by Graham and Gijswijt (1998)

#### Torymus cupratus

Boheman, 1834

##### Notes

Added by Graham and Gijswijt (1998)

#### Torymus curticauda

Graham & Gijswijt, 1998

##### Distribution

England, Wales

##### Notes

Added by Graham and Gijswijt (1998)

#### Torymus curtisi

Graham & Gijswijt, 1998

##### Distribution

England

##### Notes

Added by Graham and Gijswijt (1998)

#### Torymus cyaneus

Walker, 1847

dubius Ratzeburg, 1848
eurynotus (Walker, 1850, *Callimome*)
eurynotus (Förster, 1859, *Syntomaspis*)
lazulinus (Förster, 1859, *Syntomaspis*)

##### Distribution

England

#### Torymus druparum

Boheman, 1834

##### Distribution

England

#### Torymus eadyi

Boheman, 1834

##### Distribution

England

##### Notes

Added by Graham and Gijswijt (1998)

#### Torymus erucarum

(Schrank, 1781)

Ichneumon erucarum Schrank, 1781
fulgens Fabricius, 1798 (*Ichneumon*)
purpurascens (Olivier, 1791, *Cynips*)
fuliginosa (Spinola, 1808, *Diplolepis*)
aurulentus Nees, 1834
fulgidus Boheman, 1834
rasaces (Walker, 1844, *Callimome*)
rubripes Ratzeburg, 1844

##### Distribution

England

#### Torymus fagineus

Graham, 1994

##### Distribution

England

##### Notes

Added by Graham (1994)

#### Torymus fastuosus

Boheman, 1834

robustus Ratzeburg, 1852

##### Distribution

England

#### Torymus filipendulae

Graham & Gijswijt, 1998

##### Distribution

England

##### Notes

Added by Graham and Gijswijt (1998)

#### Torymus flavipes

(Walker 1833)

Callimome flavipes Walker, 1833
auratus (Geoffroy, 1785, *Cynips*) preocc.
aequalis (Walker, 1833, *Callimome*)
ater (Walker, 1833, *Callimome*)
autumnalis (Walker, 1833, *Callimome*)
bicolor (Walker, 1833, *Callimome*)
chlorinus (Walker, 1833, *Callimome*)
dauci (Walker, 1833, *Callimome*)
exilis (Walker, 1833, *Callimome*)
gracilis (Walker, 1833, *Callimome*)
latus (Walker, 1833, *Callimome*)
leptocerus (Walker, 1833, *Callimome*)
leucopterus (Walker, 1833, *Callimome*)
meridionalis (Walker, 1833, *Callimome*)
minutus (Walker, 1833, *Callimome*)
mutabilis (Walker, 1833, *Callimome*)
stramineitarsus (Walker, 1833, *Callimome*)
terminalis (Walker, 1833, *Callimome*)
euchlorus Boheman, 1834
viridissimus Boheman, 1834
nanus Förster, 1841
propinquus Förster, 1841
appropinquans Ratzeburg, 1844
noerdlingeri Ratzeburg, 1844
contractus Ratzeburg, 1848
gallarum Ratzeburg, 1852
hibernans Mayr, 1874
sodalis Mayr, 1874

##### Distribution

England

#### Torymus formosus

(Walker 1833)

Callimome formosus Walker, 1833
amoenus Boheman, 1834
compressus Förster, 1841

##### Distribution

England

#### Torymus fuscicornis

(Walker 1833)

Callimome fuscicornis Walker, 1833
posticus (Walker, 1833, *Callimome*)
moelleri (Thomson, 1876, *Lioterphus*)

##### Distribution

England

#### Torymus fuscipes

Boheman, 1834

#### Torymus galeobdolonis

Graham & Gijswijt, 1998

##### Distribution

England

##### Notes

Added by Graham and Gijswijt (1998)

#### Torymus galii

Boheman, 1834

##### Distribution

England

#### Torymus geranii

(Walker 1833)

Callimome geranii Walker, 1833
cyniphidum Ratzeburg, 1844
lusitanicus Tavares, 1901

##### Distribution

England

#### Torymus gloriosus

Graham & Gijswijt, 1998

##### Distribution

England

##### Notes

Added by Graham and Gijswijt (1998)

#### Torymus grahami

Bouček, 1994

##### Distribution

England, Wales

##### Notes

Added by Bouček (1994)

#### Torymus hederae

(Walker 1833)

Callimome hederae Walker, 1833

#### Torymus heterobiae

Graham & Gijswijt, 1998

##### Distribution

England

##### Notes

Added by Graham and Gijswijt (1998)

#### Torymus heyeri

Wachtl, 1883

##### Distribution

England

#### Torymus hylesini

Graham, 1994

##### Distribution

England

##### Notes

Added by Graham (1994)

#### Torymus igniceps

Mayr, 1874

##### Distribution

England

#### Torymus impar

Rondani, 1877

bakkendorfi (Hoffmeyer, 1933, *Callimome*)
drewseni Zavada, 2001

##### Distribution

England

#### Torymus juniperi

(Linnaeus 1758)

Ichneumon juniperi Linnaeus, 1758
maestus (Walker, 1833, *Callimome*)
amethystinus Boheman, 1834
solinus (Walker, 1848, *Callimome*)
budensis Erdös, 1955

#### Torymus laetus

(Walker 1833)

Callimome laetus Walker, 1833
congruens Förster, 1841
rufipes Förster, 1841
hormomyiae Kieffer, 1899

##### Distribution

England, Ireland

#### Torymus lampros

Graham, 1994

##### Distribution

England

##### Notes

Added by Graham (1994)

#### Torymus lathyri

Graham & Gijswijt, 1998

##### Distribution

England

##### Notes

Added by Graham and Gijswijt (1998)

#### Torymus microcerus

(Walker 1833)

Callimome microcerus Walker, 1833
aerope (Walker, 1844, *Callimome*)
insolitus (Walker, 1874, *Callimome*)
liogaster Thomson, 1876
saliciperdae Ruschka, 1921
henrikseni (Hoffmeyer, 1930, *Callimome*)

##### Distribution

England, Scotland, Wales

#### Torymus microstigma

(Walker 1833)

Callimome microstigma Walker, 1833
brevicauda (Walker, 1833, *Callimome*)
viridis Förster, 1841
Torymus microstigma ?*strenuus* (Walker, 1871, *Callimome*)
pruni Cameron, 1883

##### Distribution

England

#### Torymus nigritarsus

(Walker 1833)

Callimome nigritarsus Walker, 1833
alpinus Thomson, 1876
taxi Ruschka, 1921

##### Distribution

England

#### Torymus nitidulus

(Walker 1833)

Callimome nitidulus Walker, 1833
pallidicornis Boheman, 1834
nanulus (Walker, 1874, *Callimome*)

##### Distribution

England

#### Torymus nobilis

Boheman, 1834

conjunctus Nees, 1834
subterraneus (Curtis, 1835, *Callimome*)

##### Distribution

England, Ireland

#### Torymus notatus

(Walker 1833)

Callimome notatus Walker, 1833
incrassata (Thomson, 1876, *Syntomaspis*)

##### Distribution

England

#### Torymus paludum

Graham & Gijswijt, 1998

##### Distribution

England, Scotland, Ireland

#### Torymus pascuorum

Bouček, 1994

##### Distribution

England

##### Notes

Added by Graham and Gijswijt (1998)

#### Torymus persicariae

Mayr, 1874

##### Notes

Added by Bouček (1994)

#### Torymus phillyreae

Ruschka, 1921

schiodtei (Hoffmeyer, 1930, *Callimome*)
scoparii (Hoffmeyer, 1930, *Callimome*)
tripudians Graham, 1993

##### Distribution

England

##### Notes

Added by Graham and Gijswijt (1998)

#### Torymus problematicus

Graham & Gijswijt, 1998

##### Distribution

England

##### Notes

Added by Graham and Gijswijt (1998)

#### Torymus pulchellus

Thomson, 1876

##### Distribution

Ireland

#### Torymus quadriceps

Graham & Gijswijt, 1998

##### Distribution

Wales

##### Notes

Added by Graham and Gijswijt (1998)

#### Torymus quercinus

Boheman, 1834

macrocentrus Ratzeburg, 1852

#### Torymus regalis

(Walker 1833)

Callimome regalis Walker, 1833

#### Torymus roboris

(Walker 1833)

Callimome roboris Walker, 1833
nitidulus Nees, 1834 preocc

##### Distribution

England

#### Torymus rubi

(Schrank 1781)

Cynips rubi Schrank, 1781
macropterus (Walker, 1833, *Callimome*)
splendidus Förster, 1841

##### Distribution

England

#### Torymus salicis

Graham, 1994

##### Distribution

England

##### Notes

Added by Graham (1994)

#### Torymus scutellaris

(Walker 1833)

Callimome scutellaris Walker, 1833
auronitens Förster, 1841
pleuralis Thomson, 1876

##### Distribution

England

#### Torymus spilopterus

Boheman, 1834

##### Notes

Added by Graham and Gijswijt (1998)

#### Torymus stenus

Graham, 1994

##### Distribution

England

##### Notes

Added by Graham (1994)

#### Torymus tanaceticola

Ruschka, 1921

##### Notes

Added by Jennings (2009d)

#### Torymus tipulariarum

Zetterstedt, 1838

pumilus Ratzeburg, 1844

##### Distribution

England

#### Torymus ulmariae

Ruschka, 1921

##### Distribution

England

#### Torymus varians

(Walker 1833)

Callimome varians Walker, 1833
annellus (Thomson, 1876, *Syntomaspsis*)
pubescens Förster, 1841

##### Distribution

England

#### Torymus ventralis

(Fonscolombe 1832)

Cinips ventralis Fonscolombe, 1832
antennatus (Walker, 1833, *Callimome*)
quadricolor (Walker, 1833, *Callimome*)
versicolor (Walker, 1833, *Callimome*)
confusus (Walker, 1834, *Callimome*)
rudis (Walker, 1836, *Callimome*)
affinis Förster, 1841
modestus Förster, 1841
obscuripes Förster, 1841
discolor (Thomson, 1876, *Callimomus*)

##### Distribution

England

#### Torymus veronicae

Ruschka, 1921

##### Notes

Added by Graham and Gijswijt (1998)

#### Torymoidini

Grissell, 1995

#### Pseudotorymus

Masi, 1921

HOLASPIS 1874 preocc.
SENEGALELLA Risbec, 1951
THIESIA Risbec, 1951

#### Pseudotorymus arvernicus

(Walker 1833)

Callimome arvernicus Walker, 1833
dubius (Nees, 1834, *Torymus*)
congener (Förster, 1841, *Torymus*)
apionis (Mayr, 1874, *Holaspis*)

##### Distribution

England

#### Pseudotorymus bollinensis

Askew, 2002

##### Notes

Added by Askew (2002)

#### Pseudotorymus frontinus

(Walker 1851)

Callimome frontinus Walker, 1851
carinata (Mayr, 1874, *Holaspis*)

##### Distribution

Wales

#### Pseudotorymus leguminus

Ruschka, 1923

##### Notes

Added by Askew (2002)

#### Pseudotorymus militaris

(Boheman 1834)

Torymus militaris Boheman, 1834
parellinus (Boheman, 1834, *Torymus*)

#### Pseudotorymus nephthys

(Walker 1848)

Callimome nephthys Walker, 1848

##### Distribution

England

#### Pseudotorymus salicis

Ruschka, 1923

medicaginis misident.

##### Notes

*P. medicaginis* (Mayr, 1874, *Torymus*) was listed as doubtfully British by Bouček and Graham (1978); Askew (2002) demonstrated that British specimens were misidentified *P. salicis*.

#### Torymoides

Walker, 1871

DIMEROMICRUS Crawford, 1910
MACRODONTOMERUS Girault, 1913
DIDACTYLIOCERUS Masi, 1916
AMEROMICRUS Nikol'skaya, 1954
PONDOTORYMUS Bou?ek, 1978

#### Torymoides kiesenwetteri

(Mayr 1874)

Holaspis kiesenwetteri Mayr, 1874
longicauda (Masi, 1916, *Dimeromicrus*)

##### Distribution

England

##### Notes

Added by Askew (1999)

#### Cryptopristus

Förster, 1856

WEBSTERELLUS Ashmead, 1893

#### Cryptopristus caliginosus

(Walker 1833)

Torymus caliginosus Walker, 1833
fulvocinctus Förster, 1859
intermedius Förster, 1859
macromerus Förster, 1859

#### Glyphomerus

Förster, 1856

OLIGOSTHENUS Förster, 1856

#### Glyphomerus stigma

(Fabricius 1793)

Ichneumon stigma Fabricius, 1793
ater (Nees, 1834, *Torymus*)
bimaculatus (Provancher, 1887, *Oligosthenus*)

##### Distribution

England

#### Glyphomerus tibialis

Förster, 1856

##### Distribution

England

#### Idiomacromerus

Crawford, 1914

LOCHITES Förster, 1856 preocc
LIODONTOMERUS Gahan, 1914
LOCHITISCA Ghesquière, 1946
LOCHIMERUS Szelényi, 1957
LIOTORYMUS Steffan, 1962

#### Idiomacromerus papaveris

(Förster 1856)

Lochites papaveris Förster, 1856

##### Distribution

England

##### Notes

Added by Jennings (2007c)

### Family Trichogrammatidae Haliday, 1851

#### Oligositinae

Ashmead, 1904

#### Chaetostrichini

Girault, 1912

#### Aphelinoidea

Girault, 1911

LATHROMEROIDES Girault, 1913
DIACLAVA Blood & Kryger, 1928
KRYGERIOLA Novicki, 1934
THALESANNA Girault, 1938
LENGERKENIOLA Novicky, 1946
ENCYRTOGRAMMA De Santis, 1957
TANYGRAMMA De Santis, 1957

#### Aphelinoidea waterhousei

(Blood & Kryger, 1928)

Diaclava waterhousei Blood & Kryger, 1928

##### Distribution

England

#### Chaetostricha

Walker, 1851

CENTROBIA Förster, 1856
CENTROBIELLA Girault, 1912
RATZEBURGALLA Girault, 1938

#### Chaetostricha dimidiata

Walker, 1851

##### Distribution

Ireland

#### Chaetostricha doricha

(Walker, 1839)

Pteroptrix doricha Walker, 1839
errata (Nowicki, 1935, *Centrobia*)

##### Distribution

England

#### Chaetostricha silvestrii

(Kryger, 1920)

Centrobia silvestrii Kryger, 1920

#### Chaetostricha walkeri

(Förster, 1851)

Trichogramma walkeri Förster, 1851

#### Lathromeris

Förster, 1856

LATHROMERELLA Girault, 1912
GAROUELLA Risbec, 1956

#### Lathromeris scutellaris

Förster, 1856

italica (Nowicki, 1927, *Lathromerella*)
austriaca Soyka, 1934

#### Monorthochaeta

Blood, 1923

#### Monorthochaeta nigra

Blood, 1923

##### Distribution

England

#### Tumidiclava

Girault, 1911

ORTHONEURA Blood, 1923 preocc.
ORTHONEURELLA Blood & Kryger, 1929

#### Tumidiclava bimaculata

(Blood, 1923)

Orthoneura bimaculata Blood, 1923

##### Distribution

England

#### Ufensia

Girault, 1913

NEOCENTROBIA Blood, 1923
STEPHANOTHEISA Soyka, 1931
GRANTANNA Girault, 1939

#### Ufensia foersteri

(Kryger, 1919)

Centrobia foersteri Kryger, 1919
hirticornis (Blood, 1923, *Neocentrobia*)
vitoldi (Soyka, 1931, *Stephanotheisa*)

##### Distribution

England

#### Uscana

Girault, 1911

BRUCHOCTONUS Grese, 1923

#### Uscana fumipennis

(Blood, 1923)

Centrobia fumipennis Blood, 1923
princeps Steffan, 1954

##### Distribution

England

#### Xiphogramma

Nowicki, 1940

#### Xiphogramma holorhoptra

Nowicki, 1940

#### Oligositini

Ashmead, 1904

#### Chaetostrichella

Girault, 1914

#### Chaetostrichella pungens

(Mayr, 1904)

Brachystira pungens Mayr, 1904
platoni Girault, 1914
nigra (Kryger, 1919, *Brachista*)

#### Chaetostrichella rufina

(Nowicki, 1936)

Brachista rufina Nowicki, 1936

#### Epoligosita

Girault, 1916

PAROLIGOSITA Girault & Dodd, 1915 preocc.

#### Epoligosita nudipennis

(Kryger, 1919)

Oligosita nudipennis Kryger, 1919

#### Megaphragma

Timberlake, 1924

#### Megaphragma sp. indet.

##### Distribution

England

##### Notes

BMNH, det. Noyes & Polaszek, added here. Two unidentified species of *Megaphragma* have been found in England (J. Noyes, A. Polaszek, pers. comm.); listed here as a record of this genus from Britain and Ireland.

#### Oligosita

Walker, 1851

WESTWOODELLA Ashmead, 1904
PAROLIGOSITA Kurdjumov, 1911

#### Oligosita acestes

(Walker, 1839)

Pteroptrix acestes Walker, 1839
werneri (Kryger, 1919, *Chaetostricha*)

##### Distribution

England

#### Oligosita collina

Walker, 1851

##### Distribution

Ireland

#### Oligosita engelharti

Kryger, 1919

#### Oligosita pallida

Kryger, 1919

nigromaculata Soyka, 1931
tominici Bakkendorf, 1971

#### Oligosita subfasciata

Westwood, 1879

germanica Girault, 1914
incrassata Kryger, 1919
bella (Kurdjumov, 1911, *Paroligosita*)

#### Prestwichia

Lubbock, 1864

AUSTROMICRON Tillyard, 1926

#### Prestwichia aquatica

Lubbock, 1864

##### Distribution

England

#### Pseudoligosita

Girault, 1913

ZORONTOGRAMMA Silvestri, 1915

#### Pseudoligosita krygeri

(Girault, 1929)

Oligosita krygeri Girault, 1929
pulchra (Kryger, 1919, *Chaetostricha*)
formosa (Nowicki, 1935, *Oligosita*)
aurulenta (Doutt, 1961, *Chaetostricha*)

#### Paracentrobiini

Viggiani, 1971

#### Paracentrobia

Howard, 1897

ABBELLA Girault, 1911
BRACHISTELLA Girault, 1911
JASSIDOPHTHORA Perkins, 1912
ABBELLISCA Ghesquière, 1946

#### Paracentrobia pulchella

(Claridge, 1959)

Monorthochaeta pulchella Claridge, 1959

##### Distribution

England

#### Trichogrammatinae

Haliday, 1851

#### Trichogrammatini

Haliday, 1851

#### Mirufens

Girault, 1915

TRACHOCERA Blood & Kryger, 1928

#### Mirufens longicauda

(Blood, 1923)

Asynacta longicauda Blood, 1923
longicauda (Blood & Kryger, 1928, *Trachocera*) preocc.

##### Distribution

England

#### Ophioneurus

Ratzeburg, 1852

MOOA Girault, 1930

#### Ophioneurus signatus

Ratzeburg, 1852

#### Poropoea

Förster, 1851

#### Poropoea stollwerckii

Förster, 1851

simplex (Ratzeburg, 1852, *Ophioneurus*)
grandis (Thomson, 1878, *Ophioneurus*)

#### Pterandrophysalis

Nowicki, 1935

#### Pterandrophysalis levantina

Nowicki, 1935

##### Distribution

England

##### Notes

Added by Polaszek et al. (2009)

#### Trichogramma

Westwood, 1833

CALLEPTILES Haliday, 1833
PENTARTHRON Riley, 1872
APROBOSCA Westwood, 1879
OOPHTHORA Aurivillius, 1898
XANTHOATOMUS Ashmead, 1904
NEOTRICHOGRAMMA Girault, 1911
NUNIELLA Kostadinov, 1988

#### Trichogramma cacaeciae

Marchal, 1927

cacoeciae misspelling,
flavum Marchal, 1936

##### Distribution

England

##### Notes

Added by Fursov and Pintureau (1999)

#### Trichogramma danubiense

Birova & Kazimirova, 1997

##### Distribution

England

##### Notes

Added by Fursov (2000)

#### Trichogramma daumalae

Dugast & Voegel‚ 1984

##### Distribution

England

##### Notes

Added by Fursov (2000)

#### Trichogramma dendrolimi

Matsumura, 1926

pallida Meyer, 1940

##### Distribution

England

##### Notes

BMNH, det. Stouthamer, added here

#### Trichogramma evanescens

Westwood, 1833

latipennis (Haliday, 1833, *Calleptiles*)
vitripenne Walker, 1851
carpocapsae (Schreiner, 1907, *Pentarthron*)
piniperdae Wolff, 1915
barathrae Skriptshinsky, 1928
pini Meyer, 1940
rhenana Voegel‚ & Russo, 1982

##### Distribution

England, Ireland

#### Trichogramma lacustre

Sorokina, 1978

##### Distribution

England

##### Notes

Added by Fursov (2000)

#### Trichogramma minutum

Riley, 1871

minutissimum Packard, 1881
odontotae Howard, 1885
intermedium Howard, 1889
albipes (Ashmead, 1904, *Xanthoatomus*)
helocharae Perkins, 1907
nagarkattii Voegel‚ & Pintureau, 1982

##### Distribution

England

#### Trichogramma niveiscapus

(Morley, 1950)

Anagrus niveiscapus Morley, 1950

##### Distribution

England

#### Trichogramma semblidis

(Aurivillius, 1898)

Oophthora semblidis Aurivillius, 1898
schuberti Voegel‚ & Russo, 1982

##### Distribution

England

#### Trichogramma talitzkii

Dyurich, 1987

##### Distribution

England

##### Notes

Added by Fursov (2000)

#### Trichogrammatoidea

Girault, 1911

#### Trichogrammatoidea stammeri

(Novicky, 1946)

Trichogramma stammeri Novicky, 1946

##### Distribution

England

##### Notes

BMNH, det. Fursov, added here

### Superfamily Mymarommatoidea Debauche, 1948

#### Mymarommatoidea

Debauche, 1948

##### Notes

Sometimes included in the Serphitoidea, with the extinct family Serphitidae, but there is very little justification for this other than the two-segmented petiole, the families being otherwise dissimilar (Gibson et al. 2007). The single British species was transferred from *Palaeomymar* Meunier, 1901, by Gibson et al. (2007). The only published British distribution records are those of Blood and Kryger (1922), Askew (1990) and George (1991).

#### Mymarommatidae

Debauche, 1948

#### Mymaromma

Girault, 1920

PETIOLARIA Blood & Kryger, 1922

#### Mymaromma anomalum

(Blood & Kryger, 1922)

Petiolaria anomala Blood & Kryger, 1922

##### Distribution

England

## Supplementary Material

Supplementary material 1 British and Irish Chalcidoidea checklist 2016 data Data type: names
Brief description: Names of species included in the 2016 checklist, with notes and synonymy
File: oo_86736.xlsx Natalie Dale-Skey

Supplementary material 2 Comparison of 1978 checklist species with 2016 checklist Data type: names
Brief description: This document lists the valid species included in the 1978 checklist with their corresponding entry in the 2016 checklist, indicating name changes and exclusions. It includes lists of the species lost and gained through taxonomic changes.
File: oo_86644.xlsx Natalie Dale-Skey

Supplementary material 3 Taxa added from collections surveys Data type: names
Brief description: A list of new (unpublished) records in the British and Irish Chalcidoidea checklist based on reliably identified specimens in collections
File: oo_86606.xlsx Natalie Dale-Skey

## Figures and Tables

**Figure 7a.**
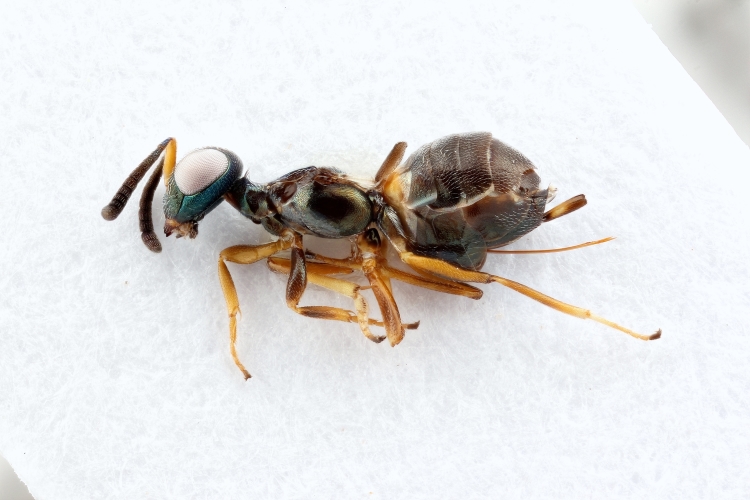
Female, BMNH specimen BMNH(E)953745 [/08/2013, Scilly Isles, Cornwall, England, coll. Dale-Skey,] (photo K. McCormack)

**Figure 7b.**
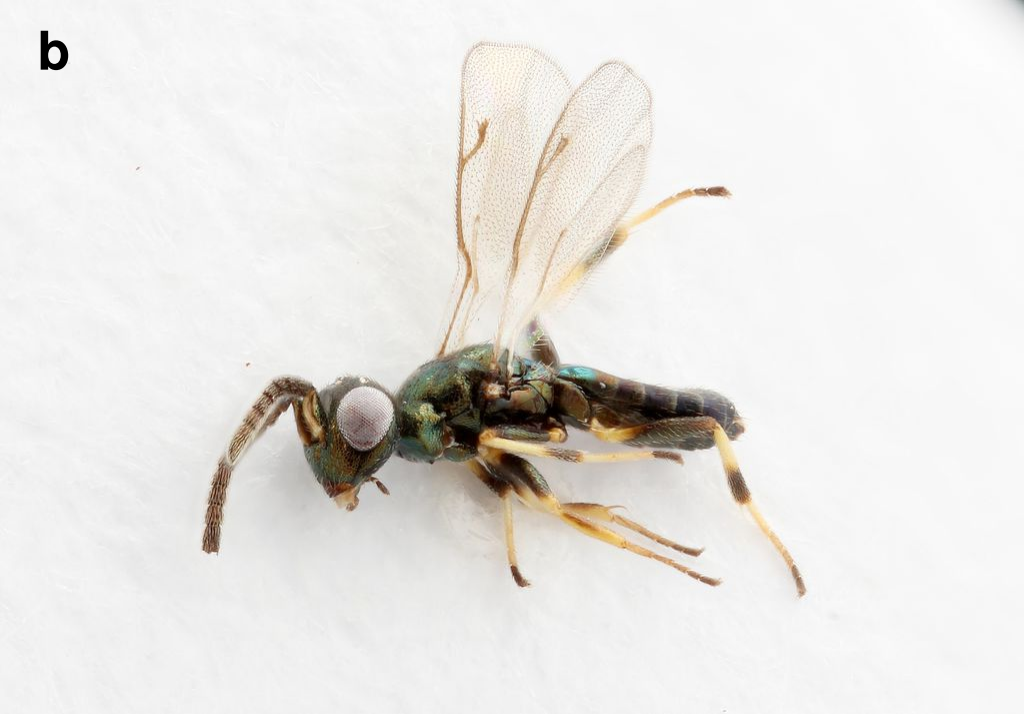
Male, BMNH specimen BMNH(E)953744 [/08/2013, Scilly Isles, Cornwall, England, coll. Dale-Skey] (photo K. McCormack)

**Figure 17a.**
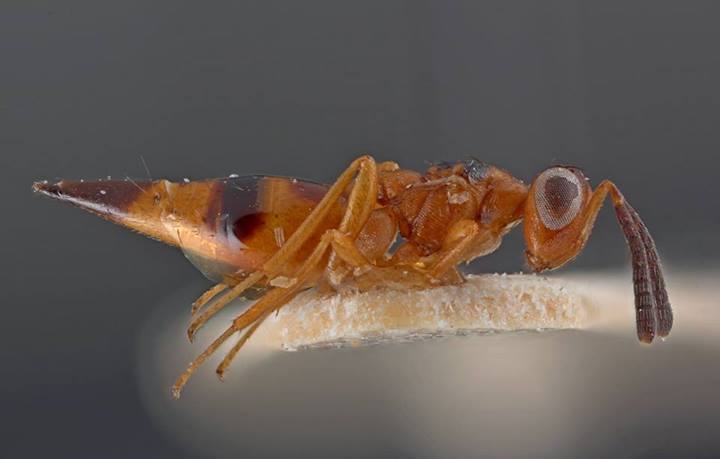
Female, BMNH specimen BMNH(E)953671 [/07/1947, Umgebung Linz, Austria, coll. Priesner] (photo K. McCormack)

**Figure 17b.**
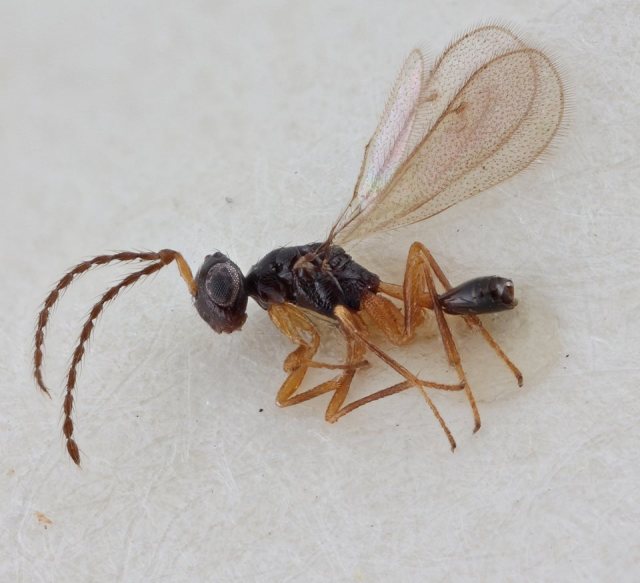
Male, BMNH specimen BMNH(E)953672 [/08/1970, Chobham Common, Surrey, England, coll. Bouček] (photo K. McCormack)

**Figure 19.**
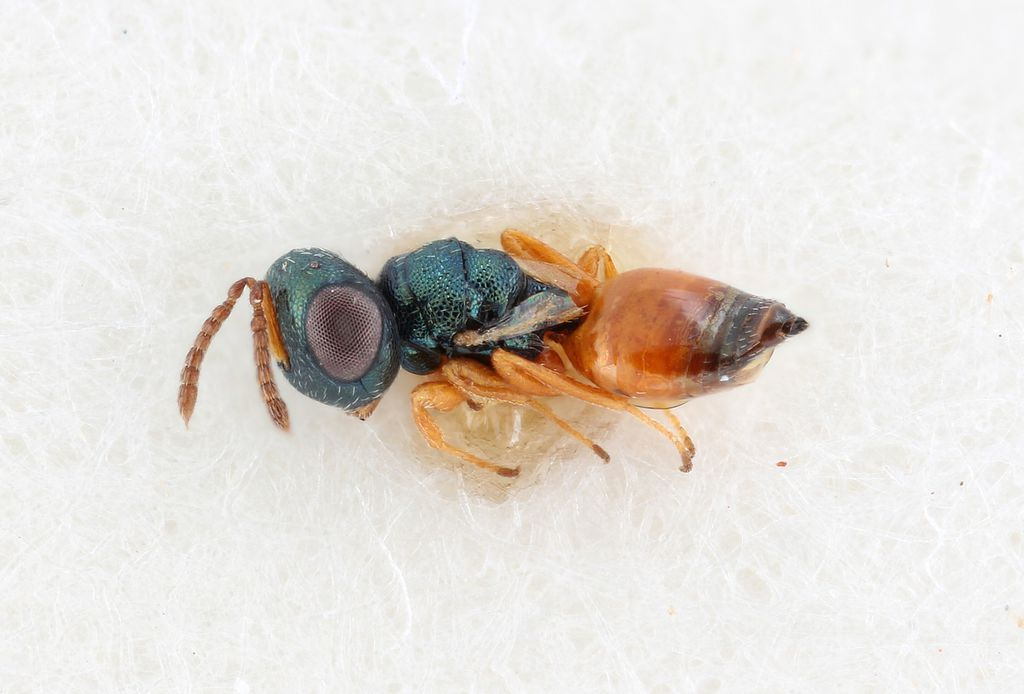
Pteromalidae, Pteromalinae: *Callitula pyrrhogaster* (Walker) female, BMNH specimen BMNH(E)1414549 [/08/1975, Box Hill, Surrey, England, coll. Bouček] (photo K. McCormack)

**Figure 20.**
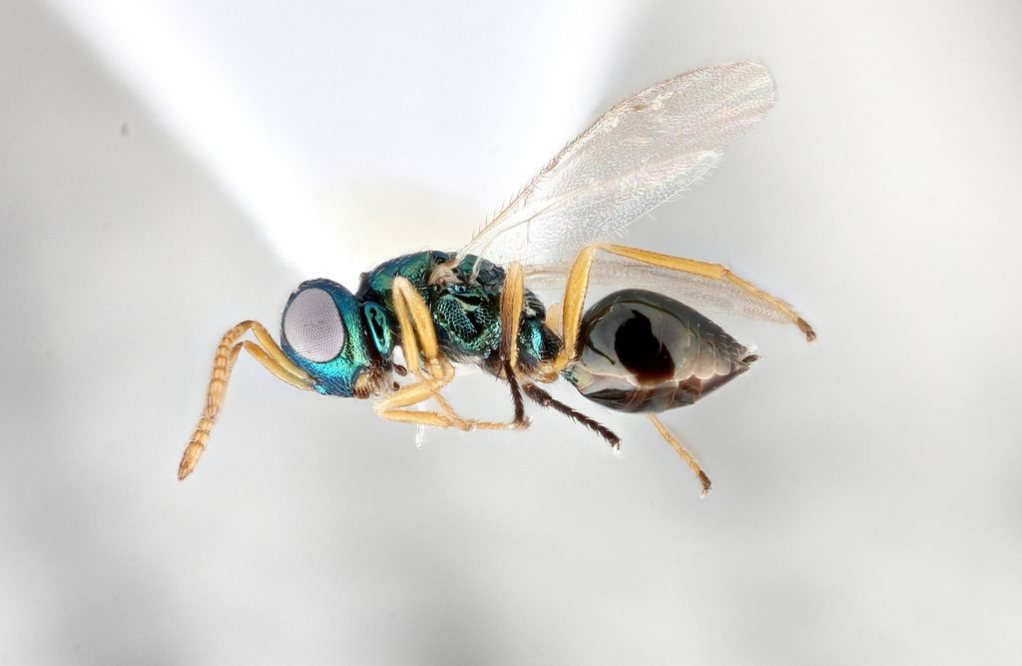
Pteromalidae, Pteromalinae: *Cyrtogaster vulgaris* Walker male, BMNH specimen BMNH(E)953675 [/08/2013, Scilly Isles, Cornwall, England, coll. Dale-Skey] (photo K. McCormack)

**Figure 21a.**
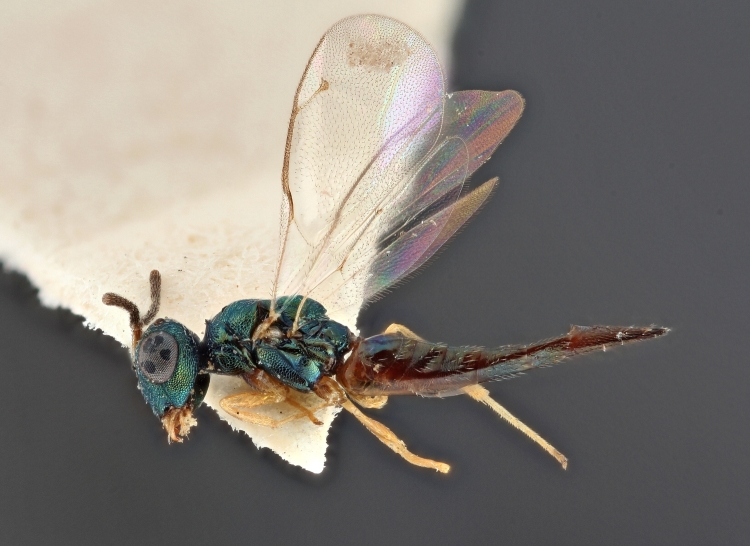
Female, BMNH specimen BMNH(E)1414504 [/1968, Surbiton, Surrey, England, coll. Danks] (photo K. McCormack)

**Figure 21b.**
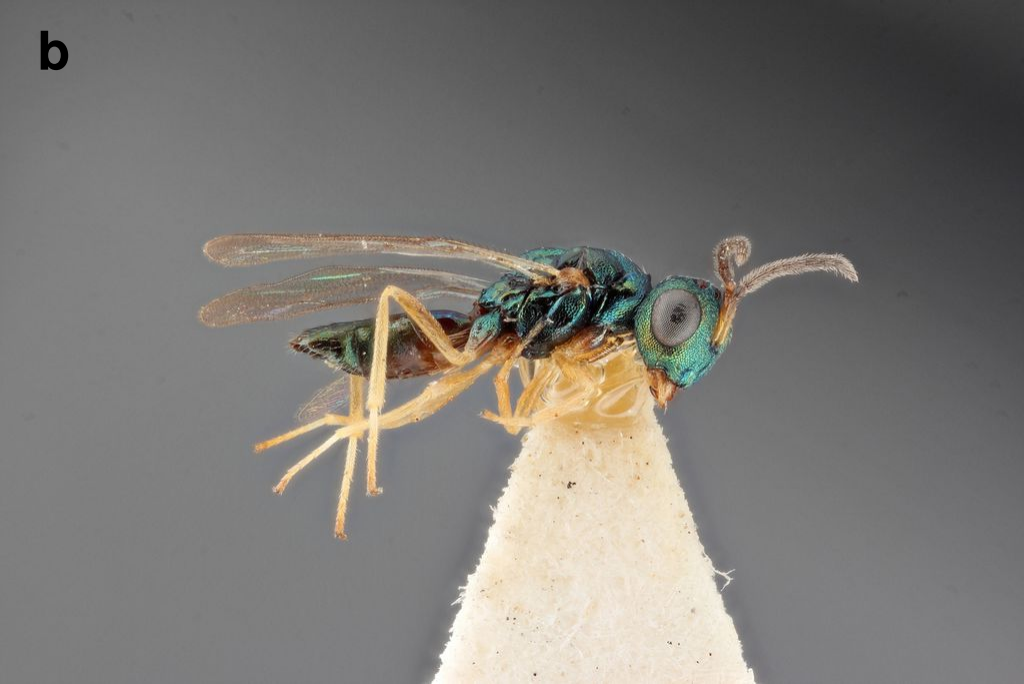
Male, BMNH specimen BMNH(E)1414505 [/1968, Surbiton, Surrey, England, coll. Danks] (photo K. McCormack)

**Figure 22.**
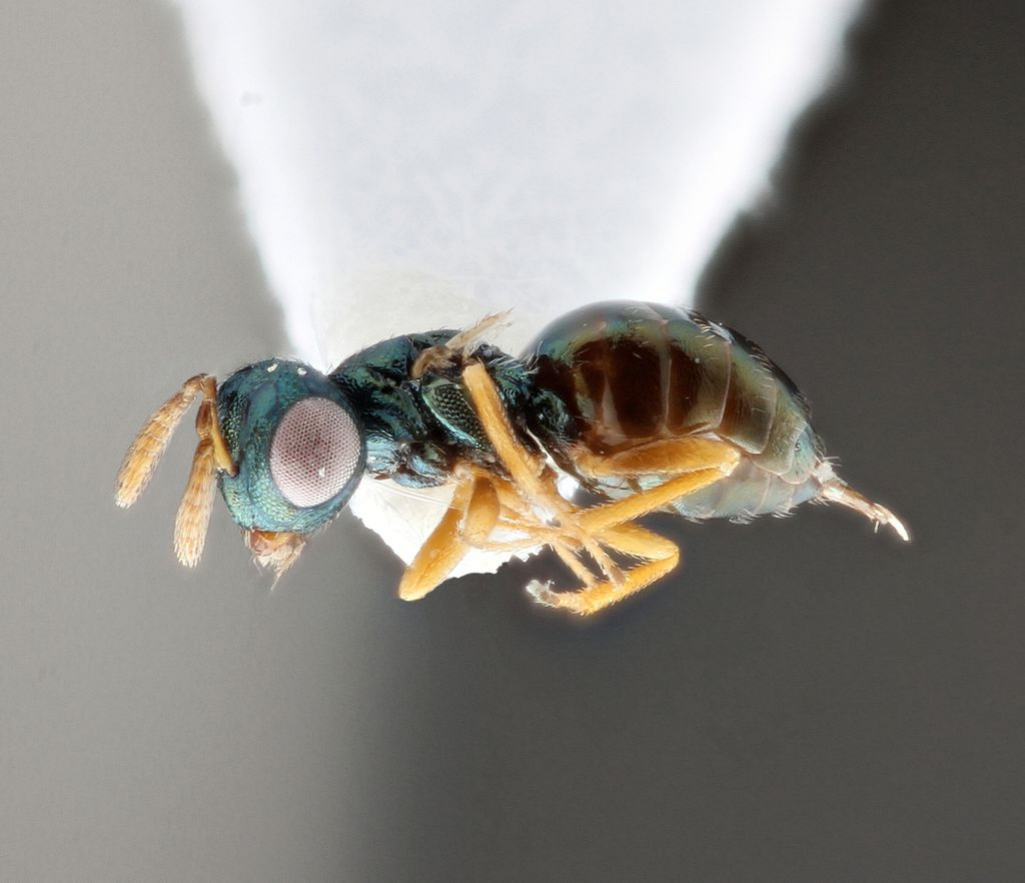
Pteromalidae, Pteromalinae: *Meraporus graminicola* Walker male, BMNH specimen BMNH(E)1414556 [/08/2013, Scilly Isles, Cornwall, England, coll. Dale-Skey] (photo K. McCormack)

**Figure 23.**
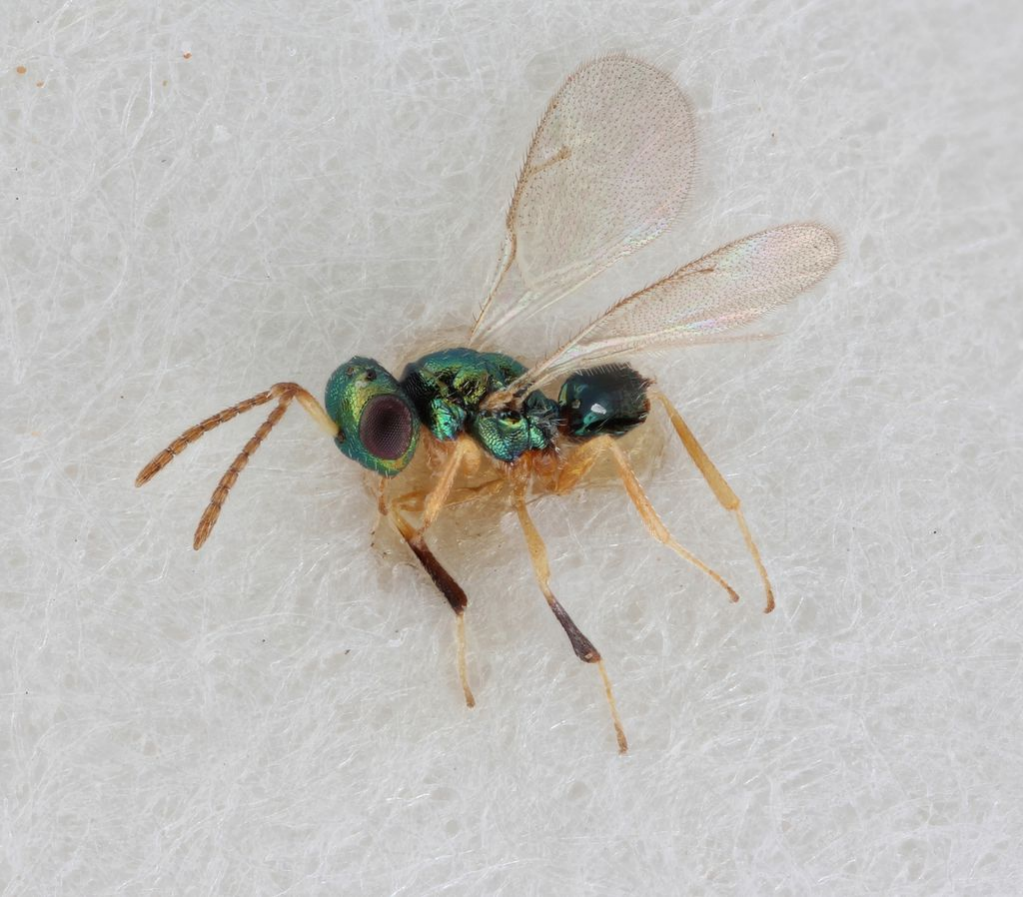
Pteromalidae, Pteromalinae: *Spaniopus amoenus* Förster male, BMNH specimen BMNH(E)1414513 [/08/1975, Burnham Beeches, Buckinghamshire, England, coll. Bouček] (photo K. McCormack)

**Figure 24a.**
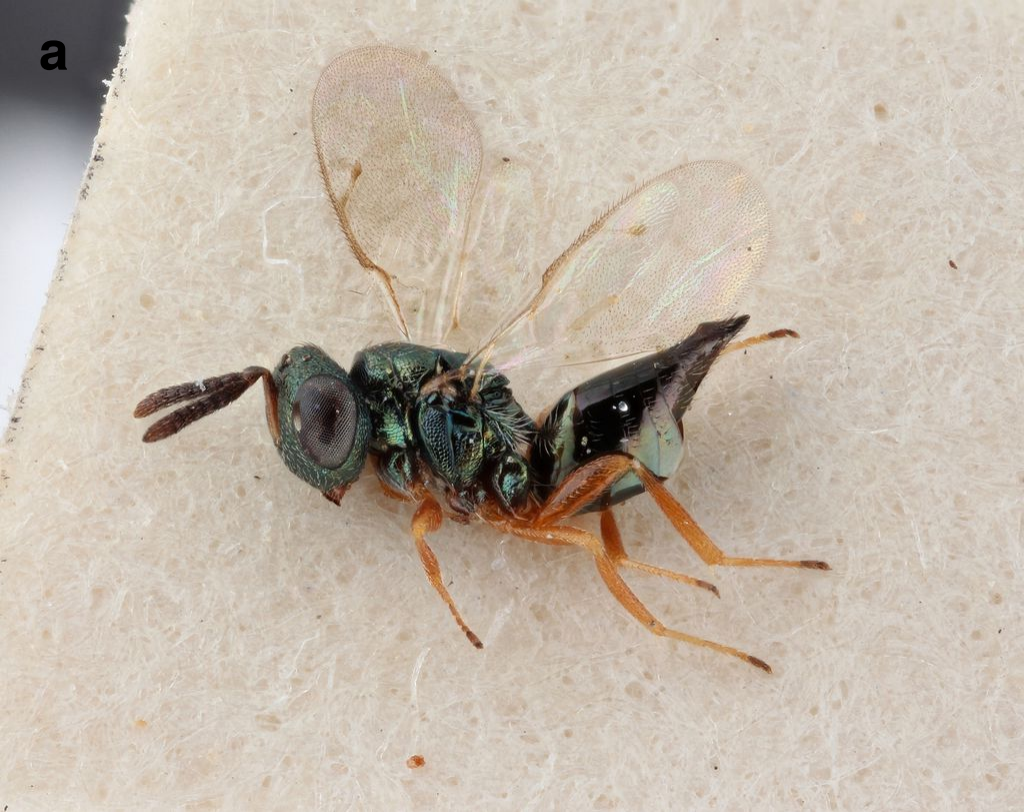
Female, BMNH specimen BMNH(E)1414511 [/07/1973, Windsor Forest, Berkshire, England, coll. Graham] (photo K. McCormack)

**Figure 24b.**
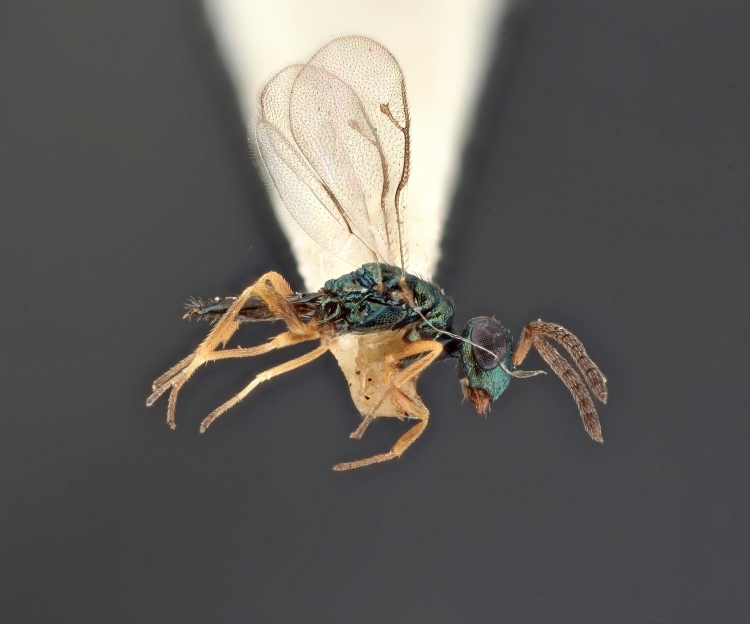
Male, BMNH specimen BMNH(E)1414510 [/08/1956, Oxon, Oxfordshire, England, coll. Graham] (photo K. McCormack)

**Table 1.** Numbers of conﬁrmed British and Irish Chalcidoidea species in the 1978 and current checklists (not including known introductions that have failed to establish in the wild or nomina dubia). The species numbers indicated in Broad (2014) have been updated to include new published records and records resulting from work done on the BMNH collections.

**Family**	**1978**	**2016**
Aphelinidae	34	40
Azotidae	1	1
Chalcididae	6	10
Encyrtidae	185	229
Eucharitidae	1	1
Eulophidae	385	509
Eupelmidae	13	20
Eurytomidae	90	97
Mymaridae	85	104
Ormyridae	3	4
Perilampidae	7	9
Pteromalidae	519	570
Signiphoridae	2	2
Tetracampidae	7	8
Torymidae	72	111
Trichogrammatidae	29	39
**Total**	**1439**	**1754**

**Table 2.** Number of species lost and gained from the 1978 checklist through taxonomic changes [*Losses* = species included as valid in 1978 and subsequently synonymised under another entry in the checklist; *Gains* = species included as junior synyonyms in 1978 whose status has been revived since] The taxa included are listed in Suppl. material 2

**Family**	**Losses**	**Gains**
Aphelinidae	2	
Encyrtidae	9	7
Eulophidae	29	11
Eupelmidae	2	
Eurytomidae	4	2
Mymaridae	11	
Pteromalidae	6	4
Torymidae	4	
Trichogrammatidae	2	
**Total**	**69**	**24**
